# Short Tryptamine-Based Peptoids as Potential Therapeutics for Microbial Keratitis: Structure-Function Correlation Studies

**DOI:** 10.3390/antibiotics11081074

**Published:** 2022-08-08

**Authors:** Ghayah Bahatheg, Rajesh Kuppusamy, Muhammad Yasir, David StC. Black, Mark Willcox, Naresh Kumar

**Affiliations:** 1 School of Chemistry, The University of New South Wales (UNSW), Sydney, NSW 2052, Australia; 2 Department of Chemistry, Faculty of Science, University of Jeddah, Jeddah 21589, Saudi Arabia; 3 School of Optometry and Vision Science, The University of New South Wales (UNSW), Sydney, NSW 2052, Australia

**Keywords:** antimicrobial peptide, peptidomimetics, peptoids, antimicrobial resistance, bacterial keratitis, *Staphylococcus aureus*, membrane disruption

## Abstract

Peptoids are peptidomimetics that have attracted considerable interest as a promising class of antimicrobials against multi-drug-resistant bacteria due to their resistance to proteolysis, bioavailability, and thermal stability compared to their corresponding peptides. *Staphylococcus aureus* is a significant contributor to infections worldwide and is a major pathogen in ocular infections (keratitis). *S. aureus* infections can be challenging to control and treat due to the development of multiple antibiotic resistance. This work describes short cationic peptoids with activity against *S. aureus* strains from keratitis. The peptoids were synthesized via acid amine-coupling between naphthyl-indole amine or naphthyl-phenyl amine with different amino acids to produce primary amines (series **I**), mono-guanidines (series **II**), tertiary amine salts (series **III**), quaternary ammonium salts (series **IV**), and di-guanidine (series **V**) peptoids. The antimicrobial activity of the peptoids was compared with ciprofloxacin, an antibiotic that is commonly used to treat keratitis. All new compounds were active against *Staphylococcus aureus S.aureus* 38. The most active compounds against S.aur38 were **20a** and **22** with MIC = 3.9 μg mL^−1^ and 5.5 μg mL^−1^, respectively. The potency of these two active molecules was investigated against 12 *S. aureus* strains that were isolated from microbial keratitis. Compounds **20a** and **22** were active against 12 strains with MIC = 3.2 μg mL^−1^ and 2.1 μg mL^−1^, respectively. There were two strains that were resistant to ciprofloxacin (*Sa.*111 and *Sa.*112) with MIC = 128 μg mL^−1^ and 256 μg mL^−1^, respectively. Compounds **12c** and **13c** were the most active against *E. coli*, with MIC > 12 μg mL^−1^. Cytoplasmic membrane permeability studies suggested that depolarization and disruption of the bacterial cell membrane could be a possible mechanism for antibacterial activity and the hemolysis studies toward horse red blood cells showed that the potent compounds are non-toxic at up to 50 μg mL^−1^.

## 1. Introduction 

Antimicrobial resistance of pathogenic bacteria poses a significant threat to global public health [1]. Microbes, especially bacteria, began to show clinically significant resistance to antibiotics such as penillicin almost as soon as the antibiotics became widely available. For example, most strains of *Staphylococcus aureus* had become resistant to penicillin in the 1950s [2]. *S. aureus* is one of the so-called ESKAPE (*Enterococcus faecium*, *Staphylococcus aureus*, *Klebsiella pneumoniae*, *Acinetobacter baumannii*, *Pseudomonas aeruginosa*, and *Enterobacter* species) pathogens that have been given the highest priority by the World Health Organisation as they are some of the most common bacterial pathogens to have acquired antibiotic resistance [3]. *S. aureus* causes a variety of infections in humans including bacteremia, infective endocarditis, osteoarticular, skin pleuropulmonary, and device-related infections [4]. *S. aureus* is also a major cause of bacterial keratitits, infection of the corea, the clear membrane overlying the pupil and colored iris. If keratitis is not rapidly treated with appropriate antibiotics. it can lead to blindness.

Microbial keratitis is usually monobacterial, with only 2.4% of cases in Sydney Australia being polymicrobial [5], and 3.7% being polybacterial in Florida, USA [6]. A longitudinal ongoing nationwide USA study reported that *S. aureus* keratitis isolates had a resistance rate to fluoroquinolones of between 25% (for moxifloxacin) and 32% (for ciprofloxacin), 15% to tobramycin, and 35% of strains were methicillin-resistant (MRSA) [7]. MRSA is increasingly being isolated from ocular infections in many countries [8,9,10]. The percentage of *S. aureus* that was isolated from microbial keratitis that are MRSA in Taiwan or India has increased by four times from 2007 to 2016 [11,12]. MRSA strains have high levels of resistance to most fluoroquinolones (75% ofloxacin, 74% ciprofloxacin) [8]. Moxifloxacin at 5000 μg/mL (0.5%) when applied to the cornea only penetrates the cornea to levels less than 0.2 μg/mL [13], which is less than the concentration of fluoroquinolones that are needed to kill resistant or non-resistant isolates. Infection by a strain of bacterium with a high minimum inhibitory concentration to antibiotics is associated with a slower time to healing [14]. There are significant linear associations between clinical outcomes and MIC for ulcers that are caused by *S. aureus* (higher MIC, worse outcome) [15]. Therefore, there is a clear need to develop improved therapeutics to treat *S. aureus*-associated keratitis.

Antimicrobial peptides (AMPs) are produced by most living organisms [16,17] and are involved in the first line of immune defense against various pathogens [17,18]. The amphipathic structure of AMPs allows the formation of pores on negatively charged bacterial cell membranes leading to cell lysis. AMPs’ efficiency and selectivity are derived from their chemical and physical properties. Their cationicity is commonly attributed to the abundance of lysine and arginine residues [19,20]. Their hydrophobicity increases the penetration of AMPs into microbial cytoplasmic membranes [21,22]. The most common hydrophobic residues that are attached to the AMPs backbone are phenylalanine, valine, alanine, and tryptophan. Although many conventional AMPs have shown antimicrobial activity, proteolytic degradation and systemic toxicity limit the clinical utilization of AMPs to topical applications [23,24,25]. For these reasons, attempts have been made to improve AMPs’ activity and alleviate these issues [26]. Peptidomimetics including *N*-substituted glycine oligomers (peptoids) [27,28], β-peptides [27,29], γ-peptides [30,31], and peptide nucleic acids [32], can enhance activity and stability [26,33].

**Figure 1:**
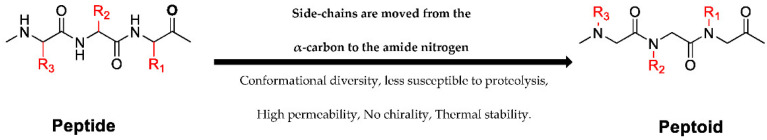
The difference between peptide and peptoid in terms of configuration and features.

**Figure 2:**
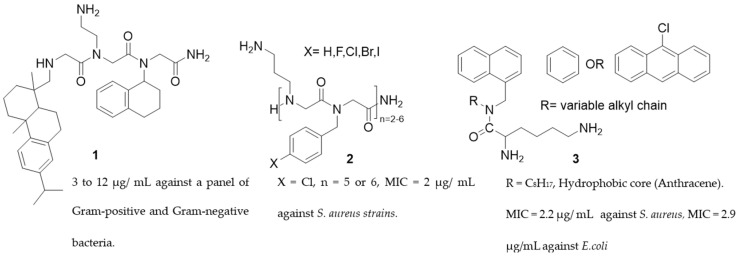
Peptoid mimics that were synthesized by various research groups with the activity of the most active molecules.

**Figure 3:**
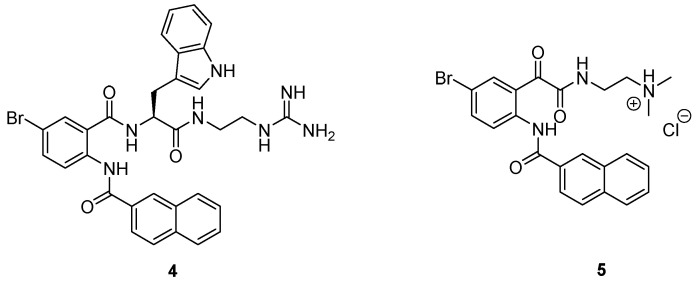
Synthetic antimicrobial peptide mimics that were reported by our group.

Peptoids (poly-N-substituted glycines) represent a new class of oligomeric compounds that mimic the natural composition of peptides (Figure 1). There is a consensus that peptoids also work via membrane disruption, but the exact nature of this mechanism is unclear [34]. Peptoids have significant biological activity; proteolytic stability against proteases such as trypsin, elastase, and chemotrypsin [35,36,37]; and have excellent cellular permeability compared with their corresponding peptides [38,39]. Peptoids also have a broad antibacterial spectrum of activity [26,40,41,42,43,44]. The first report on antibacterial peptoids was described by in 1998, among a combinatorial library of approximately 840 compounds, where CHIR29498 **1** showed the highest antimicrobial activity (Figure 2) [45]. A range of cationic peptoids of variable lengths have been synthesized utilizing lysine and halogen-substituted phenylalanine amino acids **2** (Figure 2). These had good antibacterial activity against *S. aureus*, *E. coli*, and *P. aeruginosa* [43]. The conversion of peptide K6L2W3 into its peptoid k6l2w3 enhanced its protease stability and antimicrobial selectivity [46]. Short cationic antimicrobial peptoids have also been synthesized using aromatic scaffolds, including naphthyl, phenyl, and anthracene as hydrophobic centers in addition to lysine chain as the cationic group **3** (Figure 2) [47]. Our research group has synthesized peptide mimics using indole and naphthyl scaffolds **4**, **5** with excellent MIC values (Figure 3). The current study generated peptoid molecules using tryptophan and naphthyl cores and investigated their activity against keratitis isolates of *S. aureus*.

## 2. Results and Discussion

### 2.1. Synthesis of Antimicrobial Peptoids

The peptoids were prepared by reductive amination of 2-naphthyldehyde with tryptamine or phenylethylamine to produce naphthyl, phenyl, and indole secondary amines and the subsequent acid-amine coupling followed by the removal of boc groups. In order to understand the contribution of the hydrophilic/hydrophobic balance to activity, different chain length of amines were incorporated. For cationicity, primary amine (series **I**), guanidine (series **II**), tertiary amine salt (series **III**), and quaternary ammonium salts (series **IV**) were synthesied. Another series was produced using naphthyl indole as the primary scaffold to produce diamine and diguanidine peptoids (series **V**). The antibacterial potency of these molecules was tested against strains of *Staphylococcus aureus*, as well as the Gram-negative bacterium *Escherichia coli* K12 (ATCC 10798).

**Scheme 1:**
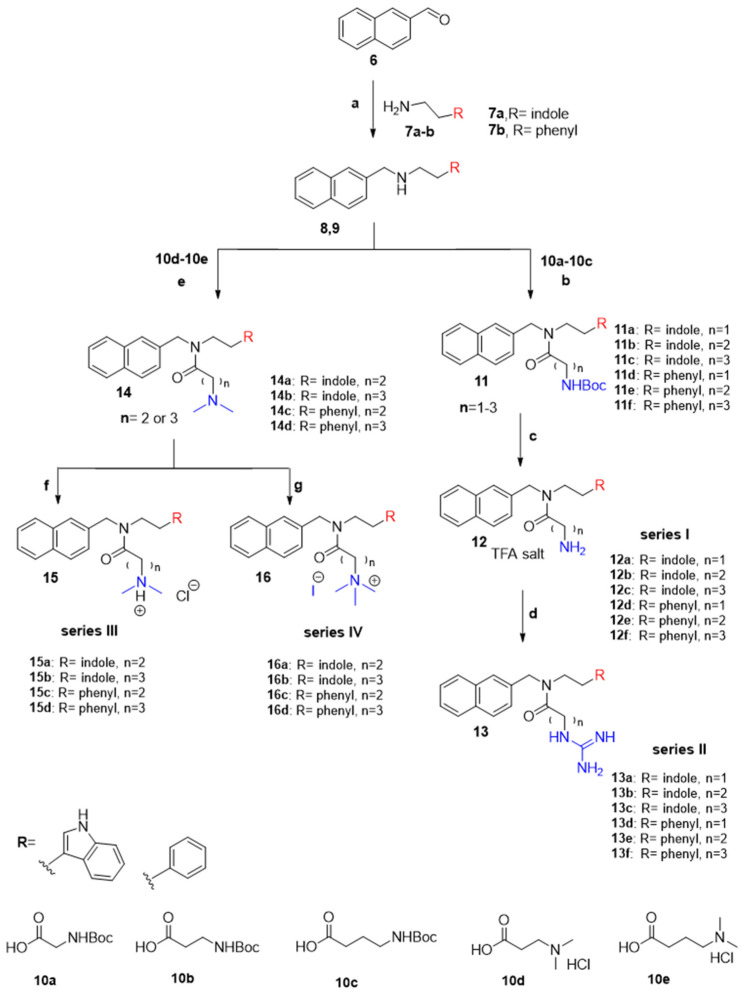
General synthetic scheme to synthesize naphthyl-based peptoids. Reaction conditions (**a**) **7a** or **7b** (1 equiv.), TMOF, rt, 1.5 h, AcOH, NaCNBH_3_ (0.3 equiv.) 20 min rt. (**b**) **10a**–**10c** (1.0–2.0 equiv.), coupling reagent (HATU 1.1 equiv.), (EDC 3.0 equiv.) or (HBTU 2.0 equiv.), DIPEA (3.0 equiv.) or Et_3_N (3.0 equiv.), DMF, rt, 2–3 h. (**c**) DCM (1–3 mL), (0.1–0.3 mL) of thioanisole (TA) and 1,2-ethanedithiol (EDT), TFA (1–3 mL). rt, 1–3 h. (**d**) pyrazole-1*H*-carboxamidine. HCl (1.0 equiv.), DIPEA (3.0 equiv.) and DMF stir at rt overnight. (**e**) 10d or 10e (1.1 equiv.), HATU (1.1 equiv.) and DMF rt, overnight. (**f**) 1 N HCl, rt, 30 min. (**g**) CH_3_I (2.0 equiv.), CH_3_CN, rt, overnight.

The initial core scaffolds were synthesized via a reductive amination reaction between aldehyde **6** bearing a naphthyl group and amine **7a** bearing an indole group, to produce the indole-naphthyl secondary amine scaffold (Scheme 1) [48]. The effect of substituting phenyl rings in place of naphthyl or indole resulting in scaffolds **8, 9** (Supplementary Materials) on biological activity was assessed. The synthesis of compounds **11a**–**11f** was achieved via a coupling reaction of **8** and **9** with different amino acids **10a**–**10c** to give boc-peptoids in good yields. This reaction was followed by a boc-deprotection reaction utilizing trifluoroacetic acid (TFA) and dichloromethane as a solvent to yield the desired compounds (series **I**) **12a**–**12f** (Scheme 1). To generate guanidinium peptoids series **II** (**13a**–**13f**), compounds **12a**–**12f** were reacted with pyrazole-1*H*-carboxamidine hydrochloride using DIPEA and DMF. The coupling reaction of 3-(dimethylamino) propionic acid hydrochloride or 4-(Dimethyl-amino) butyric acid hydrochloride with **8** and **9** resulted in compounds **(14a**–**14d)**. The reaction of scaffolds **8** and **9** with *N*,*N*-dimethylglycine hydrochloride using different methods, reagents, and conditions was unsuccessful (Supplementary Materials). The *N*-dimethyl peptoids **14a**–**14d** were reacted with 1 N HCl at room temperature to afford the tertiary ammonium hydrochloride salts **15a**–**15d** (series **III**) (Scheme 1). Also, the reaction of compounds **14a**–**14d** and methyl iodide in acetonitrile gave the quaternary ammonium iodide salts **16a**–**16d** (series **IV**).

**Scheme 2:**
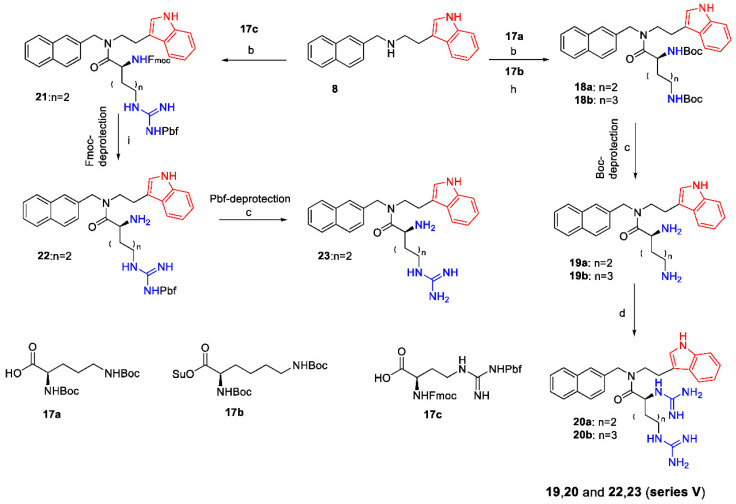
General synthetic scheme to synthesize naphthyl-indole based diamino and guanidine peptoids (series **V**). Reaction conditions: (**b**) **17a** and **17c** (1.0 equiv.), coupling reagent (HATU 1.1 equiv.), DIPEA (3.0 equiv.) and DMF, rt, 2–3 h. (**c**) DCM (1–2 m), (0.1–0.2 mL) of thioanisole (TA) and 1,2-ethanedithiol (EDT), TFA (1–2 mL). rt, 1-3 hrs. (**d**) pyrazole-1*H*-carboxamidine. HCl (1.0 equiv.), DIPEA (3.0 equiv.) and DMF stir at rt overnight. (**h**) **17b** (1.0 equiv.), Et_3_N (3.0 equiv.), DMF 5 mL. (**i**) 1.1 mL of piperidine and 4 mL of DMF overnight at rt.

To create potentially more active peptoids, the cationicity and the length of the side chain were increased. Compound **8** was treated with *tert*-butoxycarbonyl-L-ornithine under HATU coupling conditions to afford boc-compounds **18a** in 82% yield; meanwhile, Compound **8** was reacted with di-boc-L-lysine hydroxysuccinimide ester in the presence of triethylamine to give product **18b**. The removal of the boc groups from compounds **18a** and **18b** yielded the corresponding primary di-ammonium TFA salts **19a** and **19b,** respectively (Scheme 2). This was followed by the formation of guanidinium peptoids **20a** and **20b** which was achieved by the reaction of 19a and 19b with pyrazole-1H-carboxamidine hydrochloride. In an attempt to investigate the role of the two guanidinium groups in compounds **19**–**20,** compound **8** was reacted with Fmoc-Arg(pbf)-OH (**17c**) to generate compound **21**. Then the Fmoc-group was removed to give compound **22**. Eventually, the Pbf group was eliminated to yield the mono-amine guanidinium peptoid **23**.

### 2.2. ^1^H NMR Variable Temperature (VT) Study

Many studies of short cationic peptoids have utilized modelling [49,50] and NMR analysis [51,52] to demonstrate that the tertiary amides in the peptoid backbone can exist as both cis and trans isomers, as a result of restricted rotation about the partial C-N double-bond [53]. The presence of isomers could be observed by ^1^H NMR and ^13^C NMR spectroscopy at specific temperatures due to the energy barrier changes between rotamers [54]. It is possible to separate the isomers when the energy barrier is higher than 24 kcal/mol and the half-life time of the interconversion is higher than 1000 s [55]. Rotational isomerism has been observed during the ^1^H NMR and ^13^C NMR spectroscopic characterization of the majority of the peptoids in DMSO-d_6_ at room temperature [52] (Supplementary Materials). The peptoid ^1^HNMR data showed double signals for the methylene protons, some aromatic protons, and the indole-NH of peptoids containing an indole ring, as well as double signals in the ^13^C-NMR spectra for all carbons that are connected to these protons. Variable temperature (VT) ^1^H NMR was used to prove the existence of rotational isomers in this work and investigate the coalescence temperatures (Tc) when the signals of specific protons are fused into one peak. The coalescence temperature is one of the Eyring equation variables that allows the calculation of the energy barrier for coalescence [54]. Compounds **12a**, **13a**, **14a**, and **15a** were subjected to the VT ^1^H NMR experiments and the results proved that there were two isomers in the solution at room temperature. The investigation mainly concentrated on the CH_2_-naphthyl and NH- indole signals. (VT ^1^H NMR for **13a**, **14a**, and **15a** are available in the Supplementary Materials).

**Figure 4:**
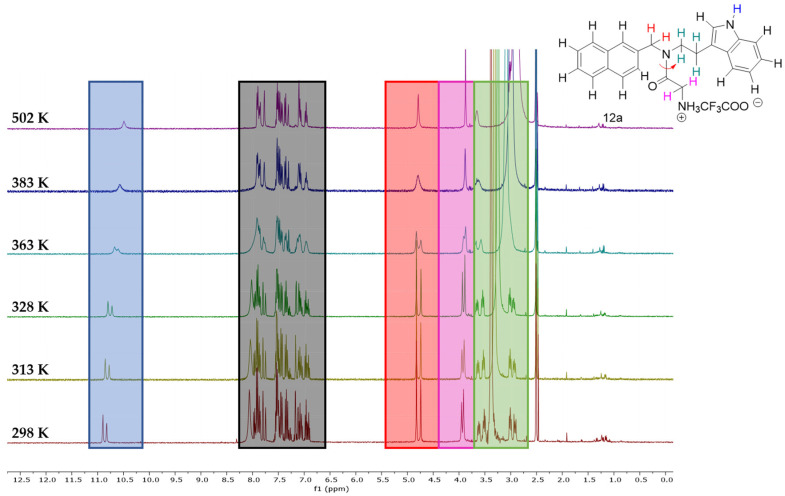
The ^1^H NMR spectra of compound **12a** at variable temperatures (298–502 K) in DMSO-d_6_.

**Figure 5:**
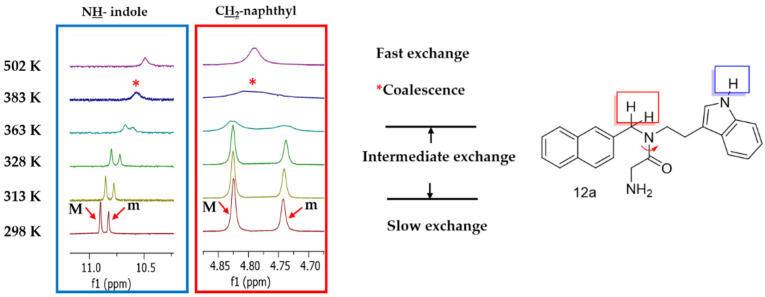
Expanded ^1^HNMR spectra of the major (M) and the minor (m) isomers for compound **12a** over 298–502 K in DMSO-d_6_, CH_2_-naphthyl region on right and NH-indole on left. (*) the coalescence temperature.

**Figure 6:**
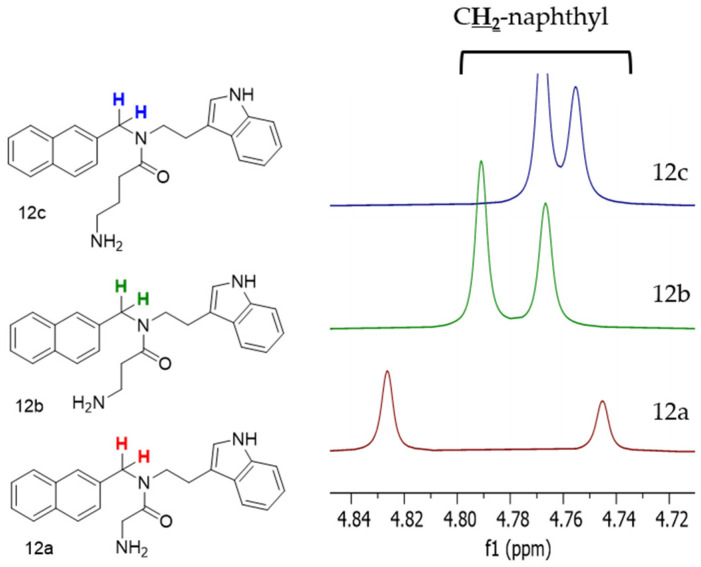
The chemical shift between CH_2_- naphthyl doublet signals in ^1^HNMRspectra in DMSO-d_6_ at 298 K compounds **12a**–**12c**.

Due to the slow interconversion, compound **12a** in DMSO-d_6_ at 298 K showed major and minor rotamers with different ratios (Figure 4). By raising the temperature, the signals of the two isomers moved closer together but were still detected. At 383 K, each of the two signals dd or dt fused into one broad peak. The signals of the rotamers coalesced to single peaks at the coalescence temperature and moved to a more shielded region, as can be observed with respect to the indole-NH peak in (Figure 5). Figure 6 shows naphthyl-CH_2_ doublet signals of compounds **12a–12c** at 298 K. Compounds **12a**–**12c** contain indole rings and have the same structure except for the length of the side chain between the amide carbonyl carbon and the NH_2_ group. Compound **12a** contains one carbon that is attached to the amide carbonyl, and compounds **12b** and **12c** have two and three carbons that are attached to the amide carbon, respectively. It is noticeable that the energy barriers between the two signals for Nph-CH_2_-N-CH_2_-CH_2_-Ind in compound **12a** is higher than that for **12b** and **12c** due to the frequency difference between the signals in compound **12a**. The same phenomenon was observed for compounds **12d**, **12e**, and **12f** when the indole ring was replaced with phenyl (^1^H NMR spectra in Supplementary Materials).

The energy barriers between two unequal isomers in compound **12a** were calculated using Eyring’s equations as modified by Shanan-Atidi and Bar-Eli [56,57] (Supplementary Materials).

### 2.3. Antimicrobial Activity and Structure Activity Relationship (SAR) Study

The determination of the minimal inhibitory concentration (MIC) was used to evaluate the antimicrobial activity of the new peptoids (series **I** to **V**) initially against the Gram-positive bacteria *Staphylococcus aureus* strain *S.aureus* 38 and Gram-negative bacteria *Escherichia coli* strain K12. The MIC values of the peptoids in these series are summarized in Table 1.

The MICs provided information on the role of the indole ring, the side chain length, and the cationicty in series **I** compounds (**12a**–**12f**). Against *Staphylococcus aureus* 38, indole derivatives **12a** and **12b** had approximately the same MIC when the side chain contained one or two carbon atoms (MIC = 21.8 μg mL^−1^ and 22.7 μg mL^−1^). The longest side chain indole derivative **12c** was found to be the most active molecule in series **I**, with MICs of 12 μg mL^−1^. The replacement of the indole moiety by a phenyl ring in series **I** compounds **12d**–**12f** decreased the activity by four-fold or more. Unlike compounds **12a**–**12b**, the side chain length played a reverse role in compounds **12d**–**12f** and led to a decline in the antibacterial activity from compound **12d** to **12f**. In general, compounds **12a**–**12f** had better antibacterial activity against *S. aureus* than *E. coli*. For *E. coli* K12, compound **12a** containing the shortest side chain length showed the highest MIC (44.65 μg mL^−1^) compared to **12b** and **12c** There was a two-fold decrease in MIC by increasing of the side chain length (**12b** MIC = 22.7 μg mL^−1^; **12c** MIC = 12 μg mL^−1^). In the absence of the indole ring, compounds **12d**–**12f** were inactive (MIC > 80 μg mL^−1^) against *E. coli.*

The idea of increasing the cationicity emerged as a result of the MICs in series **I,** with guanidinium peptoids of series **II** being generated. Overall, an increase of the net cationic charge decreased the MIC of the guanidine compounds (**13a**–**13f**), especially against *E. coli*. The MIC of series **II** compounds **13a**–**13f** was greater with *E. coli* K12 compared to *S. aureus* 38. The indole derivatives **13a**–**13c** had equivalent activity (MIC = 6.2–6.6 μg mL^−1^) against *S. aureus* 38. Of the guanidinium phenyl peptoids (**13d**–**13f**), **13d** and **13e** had equivalent MICs against *S. aureus* 38 (MIC = 11.2–11.6 μg mL^−1^), whereas **13f**, with a side chain containing three carbons, had the same MIC as the indole derivatives **13a**–**13c** (MIC = 6.1 μg mL^−1^). The phenyl guanidine peptoids **13d**–**13f** had minimal activity against *E. coli* K12 (MIC = 45–48.5 μg mL^−1^). The compounds **13a, 13b,** and **13c** showed better activity with MICs ≤ 25.3 μg mL^−1^. The series **II** compounds demonstrated that the side chain length played an important role in the activity of the indole guanidinium peptoids **13a–13c** against *E. coli*, while increases in the side chain length has improved the activity of phenyl guanidinium peptoids **9d**–**9f** against *S. aureus*.

To examine the effect of other related structures and compositions, series **III** and **IV** compounds were synthesized. However, the tertiary ammonium hydrochloride salts and quaternary ammonium iodide salts peptoids did not show much activity against either bacteria, with the lowest MIC value in this series being 50 μg mL^−1^ for **15a** against *S. aureus* 38.

The results that were obtained from series **I** showed that the indole-core and cationic character were important factors to boost antimicrobial activity. The next step was to generate more guanidine indole peptoids in series **V**. Series **V** contains six compounds. Compounds **19a** and **19b**, which are ornithine and lysine diamine indole-based peptoids, did not show improved activity against *S. aureus* 38. However, in line with our hypothesis, the lysine guanidinium peptoid **20b** was very active against *S. aureus* 38 with an MIC of 3.9 μg mL^−1^. On the other hand, compound **20a** did not show a good activity against *S. aureus* 38 (MIC = 15.5 μg mL^−1^). The Pbf-protected arginine peptoid **22** had good activity (MIC = 5.5 μg mL^−1^) against *S. aureus* 38. The MIC result of compound **23**, 7.1 μg mL^−1^, was similar to that of the simple guanidinium peptoids **13a**–**13c** and lower than the Pbf-protected peptoid **22**. Compounds **19**, **20**, **22,** and **23** did not show high antibacterial activity against *E. coli* K12, MIC ≥ 51.7 μg mL^−1^.

The most active compounds, **20b** and **22,** were tested against different clinical isolates of *S. aureus* from cases of keratitis [58] (Table 2) and compared with ciprofloxacin, a commonly used antibiotic to treat keratitis [59]. Most of the keratitis strains were resistant to ciprofloxacin (Table 2) but they were highly susceptible to compounds **20b** and **22**.

**Figure 7:**
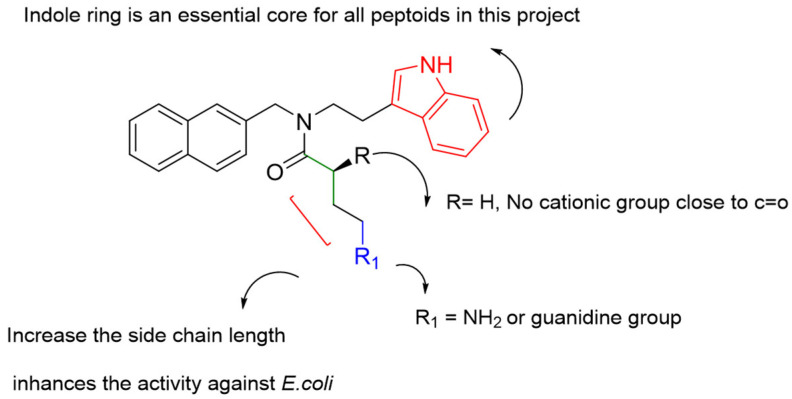
SAR of the most active compounds against *E. coli* K12.

**Figure 8:**
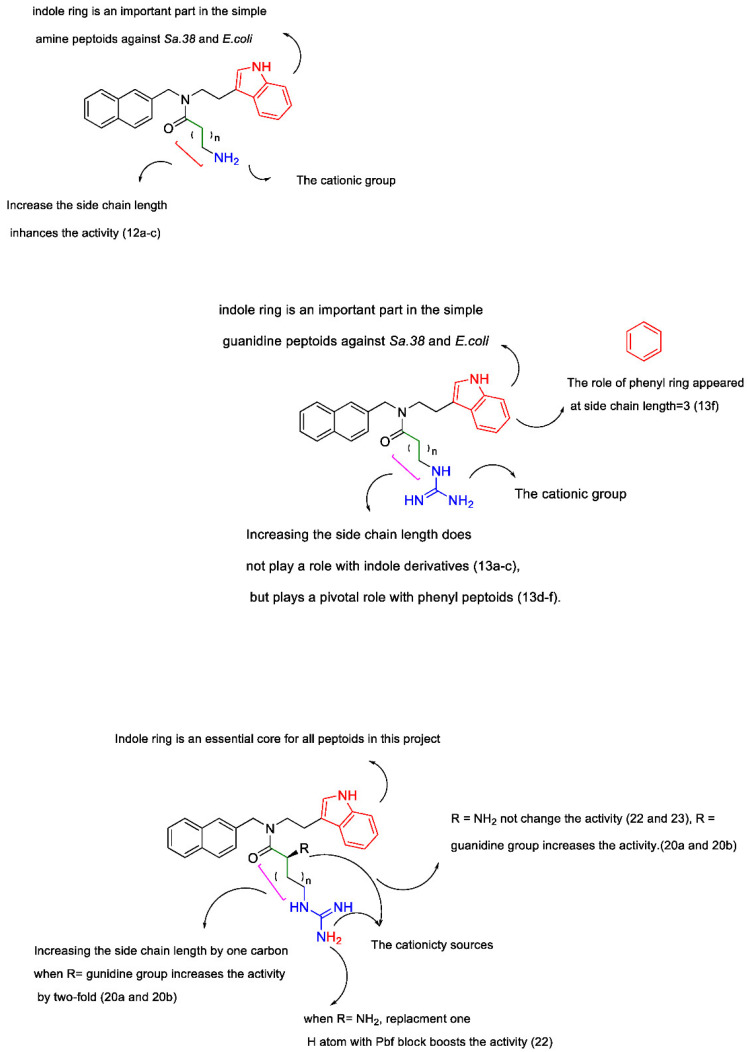
Antibacterial SAR of compounds that showed potent and good activity against *S. aureus* 38.

The structure-activity relationship of naphthyl-indole and naphthyl-phenyl backbone peptoid derivatives against *S. aureus* and *E. coli* can be sumamrised in the following. In series **I**, the replacement of the phenyl ring by an indole core yielded molecules that were active against *S. aureus* 38 and *E. coli* K12. In addition to this, increasing the net cationic charge enhanced the activity of series **II** in both the phenyl and indole peptoids against *S. aureus*. Although the side chain length did not play a role with indole derivatives (series **II**) against *S. aureus* 38, it made a notable difference with the phenyl peptoids (series **II**) activity against the same bacteria. The antibacterial activity of indole-peptoids **II** against *E. coli* was similar to their simpler amine indole peptoids, even when the cationicity was increased. In the same series, phenyl guanidinium peptoids did not show noticeable activity against *E. coli*. The experiments showed that the activity of compounds **13a**–**13c**, and **23** (mono-guanidinium peptoids) against *S. aerus* 38 did not depend on the side chain length or the presence of amino group attached to α-carbon in compound **23**. The increase of the side-chain size in di-guanidinium molecules from four carbons in peptoid **20a** to five carbons in peptoid **20b** increased the activity. The tertiary ammonium hydrochloride salts and quaternary ammonium iodide salt peptoids, regardless of the indole or phenyl backbone, were not active. The compounds with a net cationic charge that were attached to the α-carbon atom or close to it lost their activity against *E. coli* but had good activity against *S. aureus*. So, the most active compounds against *S. aureus* 38, **20b** and **22**, had the lowest activity against *E. coli* because they contain a guanidine or amine group that is attached to the α-carbon atom. In another example, compounds **12c** and **13c** were the most active compounds against *E. coli* with MIC of 12 μg mL^−1^ and 13.3 μg mL^−1^, respectively, due to the fact that their cationic groups were further away from the carbonyl carbon atom (attached to the γ-carbon atom). These data indicate that the most effective way to increase the activity of the current peptoids against Gram-negative bacteria would be to increase their side chain length that was connected with the amino groups. The SAR of active peptoids is also outlined in Figure 7 and Figure 8.

### 2.4. Cytoplasmic Permeability

**Figure 9:**
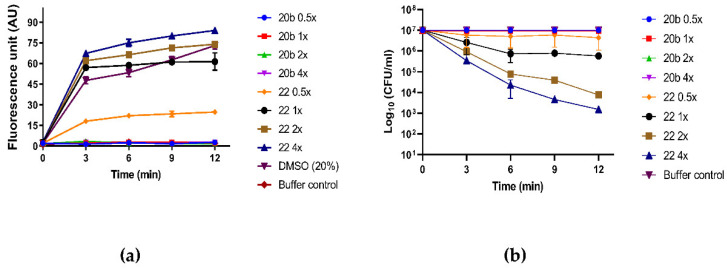
(**a**) *S. aureus* cytoplasmic membrane disruption that was promoted by **20b** and **22** (3.9 µM, 7.8 µM, 15.6 µM, 62.4 µM) at different concentrations. 20% DMSO was used as a positive control. (**b**) *S. aureus* cell viability count in the presence of **20b** and **22** at same concentrations that were used for cytoplasmic membrane disruption.

To investigate whether the peptoids affect the bacterial cytoplasmic membrane the membrane potential-sensitive dye diSC3–5 (3,3′-dipropylthiadicarbocyanine iodide) was used. The dye readily partitions into the bacterial cell membrane and aggregates within the membrane, leading to self-quenching of fluorescence [60]. However, when the bacterial cell membrane is affected or damaged via membrane destabilization or pore formation, an increase in the fluorescence due to release of the dye is observed. The Lys-diguanidine and amino-pbf-guanidine peptoids (**20b**, **22**) that showed excellent MIC values against *S. aureus* were selected to examine their mode of action. As shown in Figure 9a, compounds **22** (added at 0.5×, 1×, 2× and 4× MIC) caused the release of the dye from *S. aureus* in a time- and concentration-dependent manner, while compound **20b** (added at 0.5×, 1×, 2× and 4× MIC) did not cause a noticeable increase in the fluorescence during the experiment time. In particular, compound **22** showed an increase in the fluorescence at 0.5× MIC levels within 3 min.

To further explore the mechanism that is responsible for cell killing, we analyzed the effect of the active peptoids on bacterial cell viability as shown in Figure 9b The cell viability of compounds **20b** and **22** against S. aureus generally resembled the results that were observed in membrane depolarization studies. The compound **22** at 4× MIC showed almost 4 log reductions in bacterial numbers within 6 min and this result coincides with the dye release assay as well. Compound **20b** did not show a reduction in the bacterial numbers at all concentrations during the experiment duration.

These results indicated that membrane permeabilization may be one mechanism of action of these peptides over short time points but there must be other longer-term effects that lead to cell death, especially for **20b**. These effects might include the release of autolysins, as has been shown with the antimicrobial peptides melimine and Mel4 [61].

### 2.5. Hemolysis Assay

Hemolytic activity of the most active compounds against *S. aureus* 38 with MIC ≤ 26 μg mL^−1^ (15 peptoids) was evaluated by their ability to lyse horse red blood cells and represented as their HC_50_ values. The most active peptoids **20b** and **22** displayed haemolysis <40% at 50 ug/mL concentration. This shows the peptoids have good therapeutic index.

## 3. Experimental Section

### 3.1. General Notes

All chemical reagents were purchased from commercial sources (Combi-Blocks, San Diego, CA, USA; Chem-Impex, Wood Dale, IL, USA; Sigma Aldrich, Saint Louis, MO, USA) and used without further purification. The solvents were commercial and used as obtained. The reactions were performed using oven-dried glassware under an atmosphere of nitrogen and in anhydrous conditions (as required). Room temperature refers to the ambient temperature. Yields refer to chromatographically and spectroscopically pure compounds unless otherwise stated. The reactions were monitored by thin layer chromatography (TLC) plates that were pre-coated with Merck silica gel 60 F254. Visualization was accomplished with UV light, and a ninhydrin staining solution in n-butanol. Flash chromatography and silica pipette plugs were performed under positive air pressure using Silica Gel 60 of 230–400 mesh (40–63 μm) and also using Grace Davison LC60A 6-μm for reverse phase chromatography. Infrared spectra were recorded using a Cary 630 ATR spectrophotometer. High-resolution mass spectrometry was performed by the Bioanalytical Mass Spectrometry facility, UNSW. Proton and Carbon NMR spectra were recorded in the solvents that were specified using a Bruker DPX 300 or a Bruker Avance 400 or 600 MHz spectrometer as designated. Chemical shifts (δ) are quoted in parts per million (ppm), to the nearest 0.01 ppm and internally referenced relative to the solvent nuclei. ^1^HNMR spectroscopic data are reported as follows [chemical shift in ppm; multiplicity in br, broad; s, singlet; d, doublet; t, triplet; q, quartet; quint, quintet; sext, sextet; sept, septet; m, multiplet; or as a combination of these (e.g., dd, dt etc.)]; coupling constant (J) in hertz, integration, proton count, and assignment.

### 3.2. General Methods

#### 3.2.1. General Procedure A for the Synthesis of Compounds (**8–9–24**) via Reductive Amination

Tryptamine or phenylethylamine (1 equiv.) and 2-naphthaldehyde or benzaldehyde (1 equiv.) in trimethyl orthoformate (40 mL) was stirred for 1h at room temperature (rt) under an argon atmosphere. After 1 h, AcOH (1.6 mL) and NaCNBH_3_ (0.3 equiv.) was added to the reaction mixture, and stirring was continued for 20 min. After the completion of the reaction, 1 N NaOH was added to the mixture. The reaction mixture was extracted with ethyl acetate (350 mL) and the extract was dried over NaSO_4_, filtered, and the solvent evaporated in vaccuo. The crude product was purified by flash chromatography using 10% CHCl_3_/MeOH. The pure compound was dried under vacuum to give a solid product.

#### 3.2.2. General Procedure B for the Synthesis of Compounds (**11b–c**) via Acid Amine Coupling Reaction

The amine compound (1 equiv.) and acid (2.0 equiv.) were dissolved in DMF (7–20 mL) by stirring at rt. Then, Et_3_N (3.0 equiv.) was added to the reaction mixture, and EDC (3.0 equiv.) was added portion-wise at 0 °C. The reaction was stirred at rt between 1 to 3 h under an argon atmosphere. After the reaction completion, EtOAc was added to the reaction mixture which was then washed with water, NaHCO_3_, and brine. The extracted organic layer was concentrated under vacuum and subjected to flash chromatography (5% MeOH/CHCl_3_ as the eluent). The pure compound was dried under vacuum to give a solid product.

#### 3.2.3. General Procedure C for the Synthesis of Compounds (**11d–f**, **18a**, and **21**) via Acid Amine Coupling Reaction

The amine compound (1 equiv.), acid (1.0–2.0 equiv.), and HATU (1.1 equiv.) were dissolved in DMF (7–20 mL). Then DIPEA (3.0 equiv.) was added to the reaction portion-wise. The reaction was stirred at rt between 1 to 5 hrs under an argon atmosphere. After the reaction completion, EtOAc was added to the reaction mixture and washed with water, NaHCO_3_, and brine. The organic layer was concentrated under vacuum and subjected to flash chromatography (5% MeOH/CHCl_3_ as the eluent). The pure compound was dried under vacuum to give the product.

#### 3.2.4. General Procedure D for the Synthesis of Compounds (**11a**) via Acid Amine Coupling Reaction

The amine compound (1 equiv.), acid (2.0 equiv.), and HBTU (2.0 equiv.) were dissolved in DMF (15 mL). Then, DIPEA (3.0 equiv.) was added to the reaction portion-wise. The reaction mixtue was stirred at rt between 1 to 3 hrs under an argon atmosphere. After the reaction completion, EtOAc was added to the reaction mixture and washed with water, NaHCO_3_, and brine. The organic layer was concentrated under vacuum and subjected to flash chromatography (5% MeOH/CHCl_3_ as the eluent). The pure compound was dried under vacuum to give a solid product.

#### 3.2.5. General Procedure E for the Synthesis of Compounds (**18b**)

The amine compound (1.0 equiv.) and Boc-Lys(Boc)-OSu (1.0 equiv.) were dissolved in DMF (5 mL). Then, Et_3_N (2.0 equiv.) was added to the reaction portion-wise. The reaction was stirred at rt under an argon atmosphere. After the reaction completion, EtOAc was added to the reaction mixture and washed with water, NaHCO_3_, and brine. The organic layer was concentrated under vacuum and subjected to flash chromatography (5% MeOH/DCM as the eluent). The pure compound was dried under vacuum to give a solid product.

#### 3.2.6. General Procedure F for the Synthesis of Compounds (**12a–c, 19a–b, 12d–f,** and **23**) (N-Boc Deprotection)

To a stirred solution of the Boc-protected peptoid in DCM (1–3 mL) Thioansiole (TA) and 1,2-Ethanedithiol (EDT) (0.1–0.3 mL) was added. Then, at 0 °C TFA (1–3 mL) was added gradually to the reaction mixture, which was stirred at room temperature for 1–3 hrs before the solvent was evaporated under vacuum. After triturating with diethyl ether, the residue was concentrated to dryness, and the product was purified by reverse phase HPLC using 0.1% trifluoroacetic acid (TFA) in water/acetonitrile (0–100%), then freeze-dried. The same protocol was followed to prepare compounds **12d–f** without using TA and EDT reagents.

#### 3.2.7. General Procedure G for the Synthesis of Compounds (**13a–f** and **20a–b**)

Peptoids **12a**–**f** or **20a**–**b** (1.0 equiv.) were dissolved in 0.4 mL DMF, then (3.0 equiv.) of DIPEA was added to the reaction mixture. The reaction mixture was stirred for 5 min at 0 °C before the addition of pyrazole-1*H*-carboxamidine HCl (1.0 equiv.) to the reaction vessel. After the addition of all the reaction components, the reaction mixture was stirred at room temperature overnight. The crude product was concentrated under reduced pressure, purified by reverse phase HPLC using 0.1% trifluoroacetic acid (TFA) in water/acetonitrile (0−100%), and freeze dried to afford the desired compounds.

#### 3.2.8. General Procedure H for the Synthesis of Compounds (**14a–d**)

The amine compound (1 equiv.), acid (1.1 equiv.), and HATU (1.1 equiv.) were dissolved in DMF (15 mL) by stirring at rt. Then, DIPEA (3.0 equiv.) was added to the reaction portion-wise. The reaction was stirred overnight at rt under an argon atmosphere. After the reaction completion, EtOAc was added and the reaction mixture was washed with water, NaHCO_3_, and brine. The organic layer was concentrated under vacuum and subjected to flash chromatography (5% MeOH/CHCl_3_ as the eluent). The pure compound was dried under vacuum to give a solid product.

#### 3.2.9. General Procedure I for the Synthesis of Compounds (**15a–d**)

To N-dimethyl peptoids (1.0 equiv.) was added 1ml of HCl (1 N) to form the HCl salt. The gummy liquid was concentrated under reduced pressure to yield a gummy product which was dissolved in the minimum amount of ACN/H_2_O and freeze-dried to afford the desired compound.

#### 3.2.10. General Procedure J for the Synthesis of Compounds (**16a–d**)

To a solution of **14a–14d** (0.1 mmol) in CH_3_CN (1.0 mL) was added CH_3_I (0.1 mmol). The reaction mixture was stirred at room temperature for 8 h. After completion of the reaction, the solvent was removed under reduced pressure and treated with diethyl ether and the solution was dried under high vacuum to yield the product.

### 3.3. Preparation of Derivatives

**2-*(*1*H-*indol-3-yl)-*N*-(naphthalen-2-ylmethyl) ethan-1-amine *(8)***. The compound **8** was prepared from tryptamine (12 mmol, 2 g) and 2-naphthaldhyde (12 mmol, 1.87 g) according to the general procedure **A** to yield a yellowish solid (1.838 g, 50%); mp 97.7–99.8 °C; **^1^H NMR** (400 MHz, DMSO) δ 10.76 (s, 1H), 7.90–7.76 (m, 3H), 7.80–7.77 (m, 1H), 7.55–7.41 (m, 4H), 7.32 (dt, *J* = 8.1, 1.0 Hz, 1H), 7.13 (d, *J* = 2.3 Hz, 1H), 7.04 (ddd, *J* = 8.1, 6.9, 1.2 Hz, 1H), 6.93 (ddd, *J* = 7.9, 7.0, 1.0 Hz, 1H), 3.91 (s, 2H), 2.93–2.79 (m, 4H), 2.26 (s, 1H); **^13^C NMR** (101 MHz, DMSO) δ 138.7, 136.2, 132.9, 132.1, 127.5, 127.4, 127.2, 126.7, 125.9, 125.8, 125.3, 122.5, 120.7, 118.3, 118.1, 112.6, 111.3, 52.9, 49.5, 25.5.; **IR (ATR): νmax** 3414, 3295, 3049, 2897, 2753, 1425, 1338, 1226, 1096, 997, 813, 737; **HRMS (ESI):** *m/z* calcd for C_21_H_20_N_2_ [M]^+^: 301.1699; found: 301.1697.

***N*-(naphthalen-2-ylmethyl)-2-phenylethan-1-amine (9)**. The compound **9** was obtained from phenylethylamine (28 mmol, 3.54 mL) and 2-naphthaldhyde (20 mmol, 4 g) according to the general procedure **A**. The product **9** was obtained as a yellowish solid product. (2.37 g, 35%); mp 41.3–42.7 °C; **^1^H NMR** (400 MHz, DMSO) δ 7.91–7.80 (m, 3H), 7.78 (d, *J* = 1.6 Hz, 1H), 7.53–7.42 (m, 3H), 7.27 7.18 (m, 5H), 3.89 (d, *J* = 0.9 Hz, 2H), 2.77 (s, 4H).**^13^C NMR** (101 MHz, DMSO) δ 140.5, 138.5, 132.9, 132.1, 128.5, 128.4, 128.3, 128.2, 127.5, 127.4, 126.7, 125.9, 125.8, 125.7, 125.3, 52.9, 50.4, 35.8; **IR (ATR): νmax** 3022, 2823, 2401, 2115, 1939, 1598, 1442, 1327, 1105, 1013, 897, 821, 739; **HRMS (ESI):** *m/z* calcd for C_19_H_19_N [M]^+^: 262.1591; found: 262.1589.

***Tert-*butyl(2-((2-(1*H*-indol-3-yl)ethyl)(naphthalen-2-ylmethyl)amino)-2-oxoethyl)carbamate (11a)**. The title compound **11a** was prepared via procedure **D**, using **8** (0.5 g, 1.6 mmol) and (Boc-Gly-OH) (0.28 g, 1.6 mmol) as a white solid compound (0.521 g, 68%); mp 146.5–148.7 °C; **^1^H NMR** (400 MHz, DMSO) δ 10.86-10.79 (s, NH-indole, 1H), 7.95–7.84 (m, ArH-naphthyl, 3H), 7.75–7.74 (m, ArH-naphthyl, 1H), 7.55–7.47 (m, ArH-naphthyl, 3H), 7.43–7.38 (m, ArH-indole, 1H), 7.34–7.31 (m, ArH-indole, 1H), 7.18–7.11 (s, ArH-indole,1H), 7.07–7.04 (m, ArH-indole, 1H), 6.96–6.91 (m, ArH-indole, 1H), 6.87–6.85 (t, NH**-**Boc, 1H), 4.76–4.72 (sAr-CH^1^H^2^*-*N-2CH_2_-(indole)Boc-Gly, 2H), 3.91–3.89 (s, α-CH^1^H^2^ of Boc-Gly, 2H), 3.56–3.49 (t, Ar-CH_2_-N-CH^1^H^2^-CH_2_-indole, 2H), 3.00–2.89 (t, Ar-CH_2_-N-CH_2_-CH^1^H^2^-indole, 2H), 1.41–1.33 (s, (CH_3_)_3_ of Boc-Gly, 9H); **^13^C NMR** (101 MHz, DMSO) δ 169.1, 168.9, 155.9, 155.8, 136.2, 136.1, 135.5, 135.1, 132.9, 132.8, 132.3, 132.2, 128.3, 128.1, 127.5, 127.5, 127.4, 127.1, 126.9, 126.3, 126.2, 126.08, 126.02, 125.9, 125.7, 125.2, 125.1, 123.1, 122.6, 121.1, 120.9, 118.3, 118.2, 118.1, 111.4, 111.3, 110.7, 77.9, 77.9, 50.1, 48.2, 47.1, 46.9, 42.1, 41.4, 28.2, 28.1, 24.1, 23.1; **IR (ATR):** vmax 3439, 3258, 3056, 2980, 2931, 2321, 2107, 1698, 1630, 1502, 1457, 1366, 1252, 1158, 1046, 969, 865, 738; **HRMS (ESI):** *m/z* calcd for C_28_H_31_N_3_O_3_ [M + Na]^+^: 480.2257; found: 480.2255.

***Tert*-butyl(3-((2-(1*H*-indol-3-yl)ethyl)(naphthalen-2-ylmethyl)amino)-3 oxopropyl) carbamate (11b)**. The title compound **11b** was synthesized from compound **8** (0.4 g, 1.3 mmol) and Boc-β-Ala-OH (0.4 g, 2.6 mmol) according to the protocol **B**. The product **11b** was obtained as an off-white solid (0.32 g, 51%); mp 57.4–58.7 °C; **^1^H NMR** (600 MHz, DMSO) δ 10.85–10.78 (s, NH-indole, 1H), 7.93–7.87 (m, ArH-naphthyl, 3H), 7.73–7.69 (m, ArH-naphthyl, 1H), 7.52–7.45 (m, ArH-naphthyl, 3H), 7.40–7.38 (m, ArH-indole, 1H), 7.33–7.31 (m, ArH-indole, 1H), 7.14–7.10 (s, ArH-indole, 1H), 7.06–7.04 (m, ArH-indole, 1H), 6.94–6.90 (m, ArH-indole, 1H), 6.80–6.71 (t, NH**-**Boc, 1H), 4.75–4.73 (s, Ar-CH^1^H^2^*-*N-2CH_2_-(indole)Boc-β-Ala, 2H), 3.56–3.48 (t, Ar-CH_2_-N-CH_2_-CH_2_-indole, 2H), 3.23–3.20 (m, β-CH_2_ of Boc-β-Ala, 2H), 2.95–2.89 (m, Ar-CH_2_-N-CH_2_-CH_2_-indole, 2H), 2.62–2.54 (t, α-CH^1^H^2^ of Boc-β-Ala, 2H), 1.37–1.34 (s, (CH_3_)_3_ of Boc- β-Ala, 9H); **^13^C NMR** (151 MHz, DMSO) δ 170.7, 155.5, 155.4, 136.2, 136.1, 135.8, 135.3, 132.9, 132.8, 132.2, 132.1, 128.3, 128.1, 127.6, 127.55, 127.5, 127.1, 126.9, 126.3, 126.1, 126.05, 126.0, 125.8, 125.7, 125.1, 124.9, 123.1, 122.6, 120.99, 120.9, 118.3, 118.2, 118.17, 118.1, 111.48, 111.4, 111.3, 110.7, 77.6, 77.5, 50.8, 47.7, 47.7, 46.6, 36.8, 36.6, 33.1, 32.2, 28.23, 28.2, 24.2, 23.2; **IR (ATR):** vmax 3295, 3050, 2921, 2320, 2102, 1687, 1621, 1499, 1453, 1364, 1245, 1162, 1080, 963, 856, 813, 740; **HRMS (ESI):** *m/z* calcd for C_29_H_33_N_3_O_3_ [M + Na]^+^: 494.2414; found: 494.2416.

***Tert*-butyl(4-((2-(1*H*-indol-3-yl) ethyl) (naphthalen-2-ylmethyl) amino)-4-oxobutyl) carbamate (11c)**. The title compound **11c** was synthesized from compound **8** (1.2 g, 4.0 mmol) and Boc-γ-Abu-OH (1.63 g, 8.0 mmol) according to the protocol **B**. The product **11c** was obtained as a white solid (1.12 g, 58%); mp 116.4–117.2 °C; **^1^H NMR** (400 MHz, DMSO) δ 10.85–10.77 (s, NH-indole, 1H), 7.93–7.87 (m, ArH-naphthyl, 3H), 7.73–7.68 (d, ArH-naphthyl, 1H), 7.54–7.44 (m, ArH-naphthyl, 3H), 7.40–7.36 (m, ArH-indole, 1H), 7.34–7.30 (m, ArH-indole, 1H), 7.14–7.09 (s, ArH-indole, 1H), 7.06–7.04 (m, ArH-indole, 1H), 6.95–6.91 (m, ArH-indole, 1H), 6.84–6.79 (t, NH**-**Boc, 1H), 4.75–4.72 (s, Ar-CH^1^H^2^*-*N-2CH_2_ (indole)Boc-γ-Abu, 2H), 3.55–3.48 (m, Ar-CH_2_-N-CH_2_-CH_2_-indole, 2H), 2.96–2.88 (m, γ -CH_2_ of Boc-γ-Abu and Ar-CH_2_-N-CH_2_-CH_2_-indole, 4H), 2.43–2.38 (m, α -CH_2_ of Boc- γ-Abu, 2H), 1.69–1.63 (p, β -CH^1^H^2^ of Boc-γ-Abu, 2H), 1.38–1.33 (s, (CH_3_)_3_ of Boc-γ-Abu, 9H); **^13^C NMR** (101 MHz, DMSO) δ 171.97, 171.9, 155.6, 136.2, 136.17, 136.0, 135.4, 132.99, 132.9, 132.2, 132.1, 128.3, 128.1, 127.6, 127.55, 127.5, 127.1, 126.9, 126.3, 126.1, 126.0, 125.9, 125.8, 125.6, 125.1, 124.9, 123.1, 122.6, 121.0, 120.9, 118.3, 118.2, 118.17, 118.1, 111.5, 111.4, 111.3, 110.8, 77.3, 50.9, 47.7, 47.6, 46.7, 29.9, 29.1, 28.24, 28.2, 25.3, 24.1, 23.2; **IR (ATR):** vmax 3357, 3292, 3052, 2938, 2712, 2287, 1912, 1681,1636, 1520, 1443, 1353, 1246, 1169, 1018, 950, 863, 817, 742; **HRMS (ESI):** *m/z* calcd for C_30_H_35_N_3_O_3_ [M + Na]^+^: 508.2570; found: 508.2572.

***Tert*-butyl (2-((naphthalen-2-ylmethyl) (phenethyl)amino)-2-oxoethyl) carbamate (11d)**. The title compound **11d** was synthesized from compound **9** (1.0 g, 4.0 mmol) and Boc-Gly-OH (0.7 g, 4.0 mmol) according to the protocol **C**. The product **11d** was obtained as a colourless gum (0.863 g, 54%); **^1^H NMR** (600 MHz, DMSO) δ 7.95–7.85 (m, ArH-naphthyl, 3H), 7.76–7.74 (d, ArH-naphthyl, 1H), 7.53–7.48 (m, ArH-naphthyl, 2H), 7.41–7.38 (m, ArH-naphthyl, 1H), 7.31–7.18 (m, ArH-phenyl, 5H), 6.86–6.82 (t, NH**-**Boc, 1H), 4.71–4.69 (s, Ar-CH^1^H^2^*-*N-2CH_2_(phenyl)Boc-Gly, 2H), 3.86–3.84 (s, α-CH^1^H^2^ of Boc-Gly, 2H), 3.51–3.46 (m, Ar-CH_2_-N-CH_2_-CH_2_-phenyl, 2H), 2.87–2.78 (m, Ar-CH_2_-N-CH_2_-CH_2_-phenyl, 2H), 1.40–1.35 (s, (CH_3_)_3_ of Boc- Gly, 9H); **^13^C NMR** (151 MHz, DMSO) δ 169.1, 169.0, 155.85, 155.8, 139.1, 138.4, 135.5, 134.9, 132.9, 132.8, 132.3, 132.1, 128.86, 128.8, 128.6, 128.59, 128.5, 128.46, 128.4, 128.39, 128.3, 128.2, 128.1, 127.6, 127.58, 127.5, 127.4, 126.43, 126.4, 126.38, 126.3, 126.2, 126.17, 126.1, 126.0, 125.9, 125.78, 125.7, 125.1, 125.0, 77.9, 77.8, 49.9, 48.1, 47.9, 47.7, 41.9, 41.4, 40.1, 34.1, 34.0, 33.2, 32.6, 28.2, 28.2, 28.1, 27.9; **IR (ATR):** vmax 3415, 3327, 2974, 2929, 2320, 2104, 1705, 1648, 1454, 1364, 1247, 1021, 948, 858, 813; **HRMS (ESI):** *m/z* calcd for C_26_H_30_N_2_O_3_ [M ]^+^: 419.2329; found: 419.2321.

***Tert*-butyl (3-((naphthalen-2-ylmethyl) (phenethyl)amino)-3-oxopropyl) carbamate (11e)**. The title compound **11e** resulted from the reaction between compound **9** (0.5 g, 2.0 mmol) and Boc-β-Ala-OH (0.72 g, 4.0 mmol) according to the protocol **C**. The product **11e** was obtained as a yellow gum (0.512 g, 62%); **^1^H NMR** (600 MHz, DMSO) δ 7.93–7.87 (m, ArH-naphthyl, 3H), 7.74–7.69 (m, ArH-naphthyl, 1H), 7.52–7.48 (m, ArH-naphthyl, 2H), 7.40–7.36 (m, ArH-naphthyl, 1H), 7.30–7.18 (m, ArH-phenyl, 5H), 6.78–6.67 (t, NH**-**Boc, 1H), 4.71–4.68 (s, Ar-CH^1^H^2^*-*N-2CH_2_-(phenyl)Boc- β-Ala Hz, 2H), 3.52–3.45 (t, Ar-CH_2_-N-CH^1^H^2^-CH_2_-phenyl, 2H), 3.21–3.17 (m, β-CH_2_ of Boc- β-Ala, 2H), 2.84–2.77 (m, Ar-CH_2_-N-CH_2_-CH_2_-phenyl 2H), 2.53–2.51 (m, α-CH_2_ of Boc- β-Ala, 2H), 1.37–1.33 (s, (CH_3_)_3_ of Boc- β-Ala, 9H); **^13^C NMR** (151 MHz, DMSO) δ 170.8, 155.5, 155.4, 139.2, 138.5, 135.7, 135.2, 132.99, 132.9, 132.2, 132.1, 128.8, 128.6, 128.4, 128.36, 128.3, 128.1, 127.6, 127.59, 127.55, 127.5, 126.38, 126.3, 126.1, 126.0, 125.89, 125.8, 125.7, 125.0, 124.7, 77.6, 77.5, 50.7, 48.5, 47.6, 47.5, 36.8, 36.5, 34.3, 33.3, 33.1, 32.2, 28.2, 28.1; **IR (ATR):** vmax 3432, 3337, 2967, 2929, 2113, 1702, 1630, 1494, 1364, 1246, 1163, 963, 813, 746; **HRMS (ESI):** *m/z* calcd for C_27_H_32_N_2_O_3_ [M]^+^: 433.2485; found: 433.2481.

***Tert*-butyl (4-((naphthalen-2-ylmethyl) (phenethyl)amino)-4-oxobutyl) carbamate (11f)**. The title compound **11f** was prepared from compound **9** (1.0 g, 4.0 mmol) and Boc-γ-Abu-OH (1.63 g, 8.0 mmol) according to the protocol **C**. The product **11f** was obtained as a white solid (0.604 g, 71%); mp 90.1–91.4 °C; **^1^H NMR** (600 MHz, DMSO) δ 7.93–7.87 (m, ArH-naphthyl, 3H), 7.75–7.67 (m, ArH-naphthyl, 1H), 7.52–7.48 (m, ArH-naphthyl, 2H), 7.40–7.35 (m, ArH-naphthyl, 1H), 7.30–7.17 (m, ArH-phenyl, 5H), 6.84–6.76 (t, NH**-**Boc, 1H), 4.72–4.67 (s, Ar-CH^1^H^2^*-*N-2CH_2_ (phenyl)Boc-γ-Abu, 2H), 3.48–3.45 (m, Ar-CH_2_-N-CH_2_-CH_2_-phenyl, 2H), 2.95–2.91 (q, γ-CH^1^H^2^ of Boc-γ-Abu, 2H), 2.85–2.74 (m, Ar-CH_2_-N-CH_2_-CH_2_-phenyl, 2H), 2.36–2.32 (t, α-CH^1^H^2^ of Boc-γ-Abu, 2H), 1.65–1.62 (p, β-CH^1^H^2^ of Boc-γ-Abu, 2H), 1.38–1.32 (s, (CH_3_)_3_ of Boc-γ-Abu, 9H); **^13^C NMR** (151 MHz, DMSO) δ 172.0, 171.9, 155.6, 155.5, 139.2, 138.6, 135.9, 135.4, 132.99, 132.9, 132.2, 132.1, 128.8, 128.6, 128.4, 128.39, 128.3, 128.0, 127.6, 127.56, 127.5, 126.36, 126.3, 126.16, 126.1, 126.0, 125.8, 125.79, 125.7, 125.0, 124.7, 77.4, 77.3, 50.8, 48.4, 47.6, 47.5, 35.7, 34.2, 33.3, 29.9, 29.1, 28.25, 28.2, 25.33, 25.3; **IR (ATR):** vmax 3354, 2939, 2881, 2320, 2113, 1644, 1523, 1439, 1361, 1247, 1168, 1018, 959, 859, 822, 748; **HRMS (ESI):** *m/z* calcd for C_28_H_34_N_2_O_3_ [M + Na]^+^: 469.2461; found: 469.2463.

***N*-(2-(1*H*-indol-3-yl) ethyl)-2-amino-*N*-(naphthalen-2-ylmethyl) acetamide(TFA salt)(12a)**. The title compound **12a** was synthesized from compound **11a** (0.1 g, 0.21 mmol) according to the protocol **F**. The product **12a** was obtained as an off-white solid (0.072 g, 90%); mp 161.1–162.8 °C; **^1^H NMR** (600 MHz, DMSO) δ 10.91–10.90 (s, NH-indole, 1H), 8.10–8.05 (t, **^+^**NH_3_ CF_3_COO^−^, 3H), 7.98–7.86 (m, ArH-naphthyl, 3H), 7.80–7.75 (s, ArH-naphthyl, 1H), 7.55–7.50 (m, ArH-naphthyl, 3H), 7.47–7.43 (m, ArH-indole, 1H), 7.36–7.32 (m, ArH-indole, 1H), 7.18–7.12 (s, ArH-indole, 1H), 7.08–7.05 (m, ArH-indole, 1H), 6.98–6.91 (m, ArH-indole, 1H), 4.83–4.75 (s, Ar-CH^1^H^2^*-*N-2CH_2_-indole (Gly), 2H), 3.97–3.91 (m, α-CH^1^H^2^ of Gly, 2H), 3.63–3.50 (m, Ar-CH_2_-N-CH^1^H^2^-CH_2_- indole (Gly), 2H), 3.03–2.91 (t, Ar-CH_2_-N-CH_2_-CH^1^H^2^-indole, 2H); **^13^C NMR** (151 MHz, DMSO) δ 166.3, 166.2, 157.9, 157.7, 136.2, 136.1, 134.8, 134.2, 132.9, 132.8, 132.4, 132.3, 128.5, 128.1, 127.65, 127.64, 127.6, 127.4, 127.0, 126.8, 126.5, 126.3, 126.2, 126.1, 126.0, 125.9, 125.5, 125.3, 123.3, 122.8, 121.1, 121.0, 118.4, 118.2, 118.17, 118.1, 111.5, 111.4, 110.9, 110.5, 50.1, 48.0, 47.0, 46.9, 23.5, 23.1; **IR (ATR):** vmax 3384, 3048, 2898, 2632, 2321, 2075, 1651, 1477, 1422, 1370, 1349, 1242, 1202, 1122, 1024, 927, 811, 720; **HRMS (ESI):** *m/z* calcd for C_23_H_23_N_3_O [M]^+^: 358.1913; found: 358.1914.

***N*-(2-(1*H*-indol-3-yl) ethyl)-3-amino-*N*-(naphthalen-2-ylmethyl) propenamide (TFA salt) (12b)**. The title compound **12b** was synthesized from compound **11b** (0.15 g, 0.31 mmol) according to the protocol **F**. The product **12a** was obtained as a brown gummy compound (0.1 g, 83%); **^1^H NMR** (600 MHz, DMSO) δ 10.90–10.82 (s, NH-indole, 1H), 7.96–7.89 (m, **^+^**NH_3_ CF_3_COO^−^, 3H), 7.77–7.74 (m, ArH-naphthyl, 4H), 7.53–7.48 (m, ArH-naphthyl, 3H), 7.44–7.42 (m, ArH-indole, 1H), 7.35–7.32 (m, ArH-indole, 1H), 7.16–7.11 (s, ArH-indole, 1H), 7.07–7.05 (m, ArH-indole, 1H), 6.96–6.93 (m, ArH-indole, 1H), 4.79–4.77 (s, Ar-CH^1^H^2^*-*N-2CH_2_-(indole)-β-Ala, 2H), 3.63–3.50 (m, Ar-CH_2_-N-CH_2_-CH_2_-indole-β-Ala, 2H), 3.09–3.05 (m, β-CH_2_ of β-Ala, 2H), 2.99–2.92 (m, Ar-CH_2_-N-CH_2_-CH_2_-indole- β-Ala, 2H), 2.87–2-77 (t, α-CH^1^H^2^ of β-Ala, 2H); **^13^C NMR** (151 MHz, DMSO) δ 169.9, 169.8, 158.0, 157.8, 136.2, 136.1, 135.4, 134.8, 133.0, 132.8, 132.3, 132.2, 128.4, 128.1, 127.6, 127.58, 127.56, 127.5, 127.0, 126.8, 126.4, 126.28, 126.2, 126.0, 125.9, 125.8, 125.1, 124.9, 123.1, 122.7, 121.0, 120.9, 118.3, 118.2, 118.1, 117.8, 115.8, 111.5, 111.4, 111.2, 110.6, 50.7, 47.8, 47.5, 47.0, 35.4, 35.3, 30.1, 29.6, 23.9, 23.2; **IR (ATR):** vmax 3392, 2934, 2727, 2343, 2107, 1750, 1674, 1610, 1455, 1370, 1122, 812, 740; **HRMS (ESI):** *m/z* calcd for C_24_H_25_N_3_O [M]^+^: 372.2071; found: 372.2068.

***N*-(2-(1*H*-indol-3-yl) ethyl)-4-amino-*N*-(naphthalen-2-ylmethyl) butanamide (TFA salt) (12c)**. The title compound **12c** was synthesized from compound **11c** (0.15 g, 0.31 mmol) according to the protocol **F**. The product **12c** was obtained as a beige gum (0.1 g, 83%); **^1^H NMR** (600 MHz, DMSO) δ 10.88–10.80 (s, NH-indole, 1H), 7.95–7.86 (m, **^+^**NH_3_ CF_3_COO^−^, 3H), 7.75–7.71 (m, ArH-naphthyl, 4H), 7.52–7.46 (m, ArH-naphthyl, 3H), 7.41–7.39 (m, ArH-indole, 1H), 7.35–7.31 (m, ArH-indole, 1H), 7.16–7.10 (s, ArH-indole, 1H), 7.07–7.05 (m, ArH-indole, 1H), 6.95–6.91(t, ArH-indole, 1H), 4.77–4.76 (s, Ar-CH^1^H^2^*-*N-2CH_2_ (indole)- γ-Abu, 2H), 3.58–3.51 (m, Ar-CH_2_-N-CH_2_-CH_2_-indole- γ-Abu, 2H), 2.98–2.88 (m, γ -CH_2_ of Boc- γ-Abu, 2H), 2.86–2.72 (m, Ar-CH_2_-N-CH_2_-CH_2_-indole-γ-Abu 2H), 2.56–2.57 (t, α -CH^1^H^2^ of Boc- γ-Abu, 2H), 1.88–1.74 (p, β-CH^1^H^2^ of Boc- γ-Abu, 2H); **^13^C NMR** (151 MHz, DMSO) δ 171.3, 158.2, 158.0, 157.8, 157.6, 136.2, 136.1, 135.8, 135.2, 132.9, 132.8, 132.3, 132.2, 128.3, 128.1, 127.6, 127.58, 127.55, 127.5, 127.1, 126.9, 126.3, 126.2, 126.1, 126.0, 125.9, 125.7, 125.2, 124.9, 123.2, 122.6, 121.0, 120.9, 118.3, 118.2, 118.18, 118.1, 118.0, 116.0, 111.47, 111.4, 111.3, 110.8, 50.8, 47.7, 47.4, 46.7, 38.6, 38.5, 29.5, 28.8, 23.9, 23.2, 22.9, 22.8; **IR (ATR):** vmax 3404, 3259, 3051, 2926, 2342, 2106, 1673, 1613, 1424, 1366, 1175, 1126, 1010, 813, 742; **HRMS (ESI):** *m/z* calcd for C_25_H_27_N_3_O [M]^+^: 386.2226; found: 386.2227.

**2-Amino-*N*-(naphthalen-2-ylmethyl)-*N*-phenethylacetamide (TFA salt) (12d)**. The title compound **12d** was synthesized from compound **11d** (0.176 g, 0.42 mmol) according to the protocol **F**. The product **12d** was obtained as a brown gum (0.1 g, 77%); **^1^H NMR** (600 MHz, DMSO) δ 8.10 (s, **^+^**NH_3_ CF_3_COO^−^, 3H), 7.97–7.87 (m, ArH-naphthyl, 3H), 7.81–7.74 (s, ArH-naphthyl, 1H), 7.55–7.49 (m, ArH-naphthyl, 2H), 7.44–7.41 (m, ArH-naphthyl, 1H), 7.33–7.19 (m, ArH-phenyl, 5H), 4.79–4.71 (s, Ar-CH^1^H^2^*-*N-2CH_2_-phenyl-Gly, 2H), 3.92–3.87 (s, α-CH^1^H^2^ of Gly, 2H), 3.59–3.47 (t, Ar-CH_2_-N-CH^1^H^2^-CH_2_-phenyl-Gly, 2H), 2.91–2.80 (t, Ar-CH_2_-N-CH_2_-CH^1^H^2^-phenyl-Gly, 2H); **^13^C NMR** (151 MHz, DMSO) δ 166.3, 166.2, 158.1, 157.9, 157.7, 157.5, 138.7, 138.3, 134.8, 134.1, 132.9, 132.8, 132.3, 132.2, 128.8, 128.6, 128.53, 128.5, 128.4, 128.1, 127.6, 127.5, 127.4, 126.56, 126.5, 126.3, 126.1, 126.0, 125.9, 125.29, 125.2, 118.2, 116.3, 49.9, 48.0, 47.7, 33.5, 33.0; **IR (ATR):** vmax 3401, 3025, 2928, 2321, 2077, 1656, 1479, 1427, 1364, 1175, 1125, 1018, 913, 816, 746; **HRMS (ESI):** *m/z* calcd for C_21_H_22_N_2_O [M]^+^: 319.1804; found: 319.1807.

**3-Amino-*N*-(naphthalen-2-ylmethyl)-*N*-phenethylpropanamide (TFA salt) (12e)**

The title compound **12e** was synthesized from compound **11e** (0.4 g, 0.92 mmol) according to the protocol **F**. The product **12e** was obtained as a brown gum (0.241 g, 78%); **^1^H NMR** (400 MHz, DMSO) δ 7.96–7.87 (m, **^+^**NH_3_ CF_3_COO^−^, 3H), 7.78–7.70 (m, ArH-naphthyl, 4H), 7.56–7.48 (m, ArH-naphthyl, 2H), 7.44–7.39 (m, ArH-naphthyl, 1H), 7.33–7.19 (m, ArH-phenyl, 5H), 4.75–4.72 (s, Ar-CH^1^H^2^*-*N-2CH_2_-(phenyl)- β-Ala, 2H), 3.59–3.46 (m, Ar-CH_2_-N-CH_2_-CH_2_-phenyl 2H), 3.08 -3.01 (m, β-CH_2_ of β-Ala, 2H), 2.89–2.73 (m, Ar-CH_2_-N-CH_2_-CH_2_-phenyl- β-Ala and α-CH_2_ of β-Ala, 4H); **^13^C NMR** (101 MHz, DMSO) δ 169.97, 169.9, 158.1, 157.8,139.0, 138.4, 135.3, 134.8, 133.0, 132.8, 132.3, 132.2, 128.7, 128.6, 128.5, 128.4, 128.1, 127.6, 127.5, 127. 4, 126.5, 126.4, 126.2, 126.1, 126.07, 126.0, 125.8, 125.1, 124.8, 50.7, 48.4, 47.7, 35.4, 35.3, 33.9, 33.2, 30.1, 29.6; **IR (ATR):** vmax 3022, 2567, 2489, 2096, 1674, 1612, 1532, 1464, 1355, 1193, 1120, 972, 911, 821, 758; **HRMS (ESI):** *m/z* calcd for C_22_H_24_N_2_O [M]^+^: 333.1961; found: 333.1961.

**4-Amino-*N*-(naphthalen-2-ylmethyl)-*N*-phenethyl- butanamide (TFA salt) (12f)**

The title compound **12f** was synthesized from compound **11f** (0.189 g, 0.42 mmol) according to the protocol **F**. The product **12f** was obtained as a light brown gum (0.09 g, 60%); **^1^H NMR** (400 MHz, DMSO) δ 7.95–7.87 (m, **^+^**NH_3_ CF_3_COO^−^, 3H), 7.75–7.69 (m, ArH-naphthyl, 4H), 7.53–7.49 (m, ArH-naphthyl, 2H), 7.41–7.38 (m, ArH-naphthyl, 1H), 7.32–7.16 (m, ArH-phenyl, 5H), 4.73–4.71 (s, Ar-CH^1^H^2^*-*N-2CH_2_-(phenyl)-γ-Abu, 2H), 3.55–3.46 (m, Ar-CH_2_-N-CH_2_-CH_2_-phenyl-γ-Abu, 2H), 2.88–2.75 (m, Ar-CH_2_-N-CH_2_-CH^1^H^2^-phenyl γ-Abu and γ -CH^1^H^2^ of Boc-γ-Abu, 4H), 2.49–2.41 (m, α-CH_2_ of Boc- γ-Abu 2H), 1.82–1.74 (m, β-CH^1^H^2^ of Boc-γ-Abu, 2H); **^13^C NMR** (101 MHz, DMSO) δ 171.4, 171.3, 158.2, 157.9, 139.1, 138.6, 135.7, 135.2, 132.9, 132.8, 132.2, 132.1, 128.9, 128.6, 128.4, 128.3, 128.1, 127.6, 127.58, 127.5, 126.4, 126.2, 126.1, 126.0, 125.99, 125.9, 125.7, 125.1, 124.8, 50.6, 48.2, 47.6, 47.4, 38.65, 38.6, 33.9, 33.2, 29.4, 28.8, 22.8, 22.8; **IR (ATR):** vmax 3405, 3025, 2931, 2105, 1674, 1619, 1423, 1366, 1174, 1125, 1032, 815, 748; **HRMS (ESI):** *m/z* calcd for C_23_H_26_N_2_O [M]^+^: 347.2117; found: 347.2118.

***N*-(2-(1*H*-indol-3-yl) ethyl)-2-guanidino-*N*-(naphthalen-2-ylmethyl) acetamide (TFA salt) (13a)**. The title compound **13a** was synthesized from compound **12a** (0.1 g, 0.3 mmol) according to the protocol **G**. The product **13a** was obtained as an off-white solid (0.07 g, 63%); mp 152.2–153.9 °C; **^1^H NMR** (600 MHz, DMSO) δ 10.91–10.81 (s, NH-indole, 1H), 7.97–7.87 (m, ArH-naphthyl, 3H), 7.79 (s, ArH-naphthyl, 1H), 7.57–7.47 (m, ArH-naphthyl and NH-guanidine, 5H), 7.45–7.42 (m, ArH-indole, 1H), 7.35–7.30 (m, ArH-indole, 1H), 7.19–7.10 (s, ArH-indole, 1H), 7.07–7.04 (m, ArH-indole, 1H), 6.94–6.89 (m, ArH-indole, 1H), 4.80–4.69 (s, Ar-CH^1^H^2^*-*N-2CH_2_-(indole), 2H), 4.27–4.23 (s, α-CH^1^H^2^- of guanidine, 2H), 3.57–3.48 (m, Ar-CH_2_-N-CH_2_-CH_2_-indole, 2H), 3.03–2.88 (m, Ar-CH_2_-N-CH_2_-CH_2_-indole, 2H); **^13^C NMR** (151 MHz, DMSO) δ 166.9, 166.6, 158.3, 158.1, 157.9, 157.7, 156.9, 156.7, 136.2, 136.1, 135.0, 134.2, 132.9, 132.8, 132.4, 132.2, 128.4, 128.1, 127.68, 127.6, 127.56, 127.5, 127.0, 126.9, 126.4, 126.28, 126.2, 126.09, 126.0, 125.8, 125.6, 125.5, 123.2, 122.7, 121.0, 120.9, 118.3, 118.26, 118.21, 118.2, 118.1, 116.2, 114.2, 111.45, 111.4, 111.1, 110.6, 49.8, 48.1, 46.56, 46.5, 43.0, 42.5, 23.5, 22.9; **IR (ATR):** vmax 3388, 3186, 3059, 2321, 2113, 1640, 1423, 1361, 1344, 1171, 1124, 1038, 952, 811; **HRMS (ESI):** *m/z* calcd for C_24_H_25_N_5_O [M]^+^: 400.2131; found: 400.2134.

***N*-(2-(1*H*-indol-3-yl) ethyl)-3-guanidino-*N*-(naphthalen-2-ylmethyl) propenamide (TFA salt) (13b)**. The title compound **13b** was synthesized from compound **12b** (0.1 g, 0.26 mmol) according to the protocol **G**. The product **13b** was obtained as an off-white solid (0.079 g, 74%); mp 55.3–57.1 °C; **^1^H NMR** (400 MHz, DMSO) δ 10.88–10.81 (s, NH-indole, 1H), 7.95–7.86 (m, 3H), 7.76–7.71 (s, ArH-naphthyl, 1H), 7.54–7.44 (m, ArH-naphthyl and NH-guanidine, 5H), 7.43–7.40 (m, ArH-indole, 1H), 7.35–7.31 (m, ArH-indole, 1H), 7.15–7.10 (s, ArH-indole, 1H), 7.09–7.01 (t, ArH-indole, 1H), 6.97–6.89 (t, ArH-indole, 1H), 4.79–4.76 (s, Ar-CH^1^H^2^*-*N-2CH_2_-(indole), 2H), 3.61–3.51 (m, β-CH^1^H^2^- of guanidine, 1H), 3.44–3.38 (t, Ar-CH_2_-N-CH^1^H^2^-CH_2_-indole, 2H), 2.99–2.90 (m, Ar-CH_2_-N-CH_2_-CH_2_-indole, 2H), 2.74–2.68 (t, α-CH^1^H^2^- of guanidine, 2H); ^**13**^**C NMR** (151 MHz, DMSO) δ 170.57, 170.5, 158.5, 158.3, 158.1, 157.9, 156.82, 156.8, 136.2, 136.1, 135.6, 135.0, 133.0, 132.9, 132.3, 132.2, 128.4, 128.1, 127.64, 127.6, 127.57, 127. 5, 127.1, 126.9, 126.4, 126.2, 126.1, 126.0, 125.9, 125.8, 125.1, 124.9, 123.1, 122.7, 121.0, 120.9, 118.4, 118.2, 118.1, 117.7, 115.7, 111.5, 111.4, 111.3, 110.7, 50.8, 47.8, 47.6, 46.9, 37.2, 37.1, 32.3, 31.7, 23.9, 23.3; **IR (ATR):** vmax 3274, 3174, 3057, 2320, 2112, 1618, 1424, 1365, 1175, 1129, 950, 813, 742; **HRMS (ESI):** *m/z* calcd for C_25_H_27_N_5_O [M]^+^: 414.2288; found: 414.2286.

***N*-(2-(1*H*-indol-3-yl) ethyl)-4-guanidino-*N*-(naphthalen-2-ylmethyl) butanamide (TFA salt) (13c)**. The title compound **13c** was synthesized from compound **12c** (0.1 g, 0.26 mmol) according to the protocol **G**. The product **13c** was obtained as a brown gum (0.091 g, 82%); **^1^H NMR** (600 MHz, DMSO) δ 10.88–10.80 (s, NH-indole, 1H), 7.95–7.86 (m, ArH-naphthyl, 3H), 7.74 (m, ArH-naphthyl, 1H), 7.70–7.69 (m, NH-guanidine, 1H), 7.53–7.45 (m, ArH-naphthyl and NH-guanidine, 4H), 7.40–7.38 (m, ArH-indole, 2H), 7.35–7.31 (m, ArH-indole, 1H), 7.07–7.05 (m, ArH-indole, 1H), 6.97–6.91 (m, ArH-indole, 1H), 4.78–4.75 (s, Ar-CH^1^H^2^*-*N-2CH_2_-(indole), 2H), 3.60–3.51 (t, γ-CH^1^H^2^- of guanidine 2H), 3.13–3.02 (m, Ar-CH_2_-N-CH_2_-CH_2_-indole, 2H), 2.98–2.89 (t, Ar-CH_2_-N-CH_2_-CH^1^H^2^-indole, 2H), 2.49–2.42 (t, α-CH^1^H^2^- of guanidine, 2H), 1.78–1.70 (p, β-CH^1^H^2^- of guanidine, 2H); **^13^C NMR** (151 MHz, DMSO) δ 171.8, 171.7, 158.6, 158.4, 158.2, 157.9, 156.9, 156.8, 136.2, 136.1, 135.8, 135.2, 132.9, 132.8, 132.3, 132.2, 128.4, 128.1, 127.6, 127.57, 127.54, 127.5, 127.1, 126.9, 126.4, 126.2, 126.09, 126.0, 125.9, 125.7, 125.1, 124.9, 123.1, 122.6, 121.0, 120.9, 118.4, 118.35, 118.3, 118.2, 118.1, 117.7, 115.7, 111.47, 111.4, 111.3, 110.8, 50.8, 47.7, 47.5, 46.8, 40.3, 40.2, 29.3, 28.7, 24.3, 24.2, 23.9, 23.2; **IR (ATR):** vmax 3342, 3180, 3067, 2320, 2112, 1663, 1616, 1424, 1365, 1173, 1129, 956, 811, 742; **HRMS (ESI):** *m/z* calcd for C_26_H_29_N_5_O [M]^+^: 428.2444; found: 428.2441.

**2-Guanidino-*N*-(naphthalen-2-ylmethyl)-*N*-phenethyl acetamide (TFA salt) (13d)**. The title compound **13d** was synthesized from compound **12d** (0.1 g, 0.31 mmol) according to the protocol **G**. The product **13d** was obtained as a brown gum (0.082 g, 72%); **^1^H NMR** (400 MHz, DMSO) δ 7.97–7.88 (m, ArH-naphthyl, 3H), 7.81–7.78 (m, ArH-naphthyl, 1H), 7.55–7.42 (m, ArH-naphthyl and NH-guanidine, 4H), 7.33–7.15 (m, ArH-phenyl and NH-guanidine, 6H), 4.78–4.65 (s, Ar-CH^1^H^2^*-*N-2CH_2_-(phenyl), 2H), 4.22–4.16 (s, α-CH^1^H^2^- of guanidine, 2H), 3.54–3.44 (m, Ar-CH_2_-N-CH_2_-CH_2_-phenyl, 2H), 2.93–2.76 (t Ar-CH_2_-N-CH_2_-CH^1^H^2^-phenyl, 2H); **^13^C NMR** (101 MHz, DMSO) δ 166.9, 166.7, 158.3, 158.0, 156.8, 138.9, 138.3, 134.9, 134.1, 132.9, 132.8, 132.4, 132.2, 128.9, 128.5, 128.43, 128.4, 128.1, 127.69, 127.6, 127.5, 126.47, 126.4, 126.2, 126.1, 126.0, 125.8, 125.5, 125.4, 49.7, 48.0, 47.37, 47.3, 43.0, 42.6, 33.5, 32.9; **IR (ATR):** vmax 3379, 3155, 2923, 2321, 2110, 1635, 1478, 1424, 1374, 1184, 1131, 1010, 799, 744, 699; **HRMS (ESI):** *m/z* calcd for C_22_H_24_N_4_O [M]^+^: 361.2022; found: 361.2023.

**3-Guanidino-*N*-(naphthalen-2-ylmethyl)-*N*-phenethyl- propanamide (TFA salt) (13e)**. The title compound **13e** was synthesized from compound **12e** (0.12 g, 0.36 mmol) according to the protocol **G**. The product **13d** was obtained as a beige gum (0.088 g, 65%); **^1^H NMR** (600 MHz, DMSO) δ 7.95–7.87 (m, ArH-naphthyl, 3H), 7.77–7.70 (d, ArH-naphthyl, 1H), 7.53–7.45 (m, ArH-naphthyl, 3H), 7.42–7.38 (m, NH-guanidine 1H), 7.31–7.28 (m, ArH-phenyl and NH-guanidine, 2H), 7.27–7.18 (m, ArH-phenyl, 4H), 4.76–4.72 (s, Ar-CH^1^H^2^*-*N-2CH_2_-(phenyl), 2H), 3.56–3.48 (m, β-CH_2_- of guanidine, 2H), 3.40–3.34 (m, Ar-CH_2_-N-CH_2_-CH_2_-phenyl, 2H), 2.87–2.79 (t, Ar-CH_2_-N-CH_2_-CH^1^H^2^-phenyl, 2H), 2.66–2.59 (t, α-CH^1^H^2^- of guanidine, 2H); **^13^C NMR** (151 MHz, DMSO) δ 170.6, 170.5, 158.5, 158.3, 158.1, 157.9, 156.8, 139.0, 138.5, 135.5, 135.0, 133.0, 132.9, 132.3, 132.2, 128.8, 128.5, 128.47, 128.4, 128.1, 127.65, 127.6, 127.5, 126.48, 126.4, 126.26, 126.2, 126.05, 126.0, 125.9, 125.8, 125.0, 124.8, 50.6, 48.3, 47.7, 47.6, 37.09, 37.0, 33.9, 33.3, 32.2, 31.7; **HRMS (ESI):** *m/z* calcd for C_23_H_26_N_4_O [M]^+^: 374.2107; found: 374.2178; **IR (ATR):** vmax 3329, 3159, 2341, 2119, 1619, 1452, 1365, 1172, 1128, 1031, 950, 813, 745; **HRMS (ESI):** *m/z* calcd for C_23_H_26_N_4_O [M]^+^: 375.2179; found: 375.2178.

**4-Guanidino-*N*-(naphthalen-2-ylmethyl)-*N*-phenethyl- butanamide (TFA salt) (13f)**. The title compound **13f** was synthesized from compound **12f** (0.1 g, 0.28 mmol) according to the protocol **G**. The product **13f** was obtained as a light brown gum (0.066 g, 61%); **^1^H NMR** (600 MHz, DMSO) δ 7.94–7.87 (m, ArH-naphthyl and NH-guanidine, 5H), 7.75–2.69 (s, ArH-naphthyl, 1H), 7.52–7.45 (m, ArH-naphthyl and NH-guanidine, 2H), 7.40–7.37 (m, NH-guanidine, 1H), 7.31–7.18 (m, ArH-phenyl and NH-guanidine, 6H), 4.75–4.71 (s, Ar-CH^1^H^2^*-*N-2CH_2_-(phenyl), 2H), 3.55–3.47 (t, γ-CH^1^H^2^- of guanidine 2H), 3.11–3.07 (m, Ar-CH_2_-N-CH_2_-CH_2_-phenyl, 2H), 2.86–2.78 (t, Ar-CH_2_-N-CH_2_-CH^1^H^2^-phenyl, 2H), 2.45–2.37 (t, α-CH^1^H^2^- of guanidine, 2H), 1.75–1.70 (m, β-CH_2_- of guanidine 2H); **^13^C NMR** (151 MHz, DMSO) δ 171.8, 159.1, 158.9, 158.7, 158.5, 156.99, 156.9, 139.1, 138.5, 135.7, 135.2, 133.0, 132.9, 132.8, 132.3, 132.2, 128.8, 128.69, 128.6, 128.45, 128.42, 128.4, 128.3, 128.1, 127.8, 127.7, 127.6, 127.58, 127.55, 127.5, 126.4, 126.2, 126.1, 126.0, 125.9, 125.7, 125.0, 124.8, 50.7, 50.2, 48.3, 47.67, 47.6, 47.5, 40.3, 40.2, 34.0, 33.2, 31.5, 29.3, 28.7, 24.3, 24.2; **IR (ATR):** vmax 3342, 3157, 2320, 2126, 1619, 1422, 1365, 1173,1125, 815, 748, 719; **HRMS (ESI):** *m/z* calcd for C_24_H_28_N_4_O [M]^+^: 389.2336; found:389.2338.

***N*-(2-(1*H*-indol-3-yl)ethyl)-3-(dimethylamino)-*N*-(naphthalen-2-ylmethyl) propenamide (14a)**. The title compound **14a** was synthesized from compound **8** (0.1 g, 0.33 mmol) according to the protocol **H**. The product **14a** was obtained as a white solid (0.095 g, 71%); mp 231.9–233.4 °C; **^1^H NMR** (600 MHz, DMSO) δ 10.89–10.80 (s, NH-indole, 1H), 7.94–7.86 (m, ArH-naphthyl, 3H), 7.76–7.72 (m, ArH-naphthyl, 1H), 7.52–7.47 (m, ArH-naphthyl, 3H), 7.42–7.40 (m, ArH-indole, 1H), 7.35–7.31 (m, ArH-indole, 1H), 7.16–7.10 (s, ArH-indole, 1H), 7.08–7.03 (m, ArH-indole, 1H), 6.97–6.91 (t, ArH-indole, 1H), 4.78–4.77 (s, Ar-CH^1^H^2^*-*N-2CH_2_-(indole)N,N- dimethylpropane, 2H), 3.59–3.53 (m, β-CH_2_ of N,N- dimethylpropane, 2H), 2.99–2.87 (m, Ar-CH_2_-N-CH_2_-CH_2_-(indole) N,N- dimethylpropane, 2H), 2.89–2.52 (m, Ar-CH_2_-N-CH_2_-CH_2_-indole- N,N- dimethylpropane and α-CH_2_ of N,N- dimethylpropane, 4H), 2.31–2.25 (s, N-CH_3_CH_3_ of N,N- dimethylpropane, 6H); **^13^C NMR** (151 MHz, DMSO) δ 170.7, 170.6, 136.2, 135.7, 135.3, 132.9, 132.8, 132.2, 132.1, 128.3, 128.0, 127.6, 127.57, 127.54, 127.5, 127.1, 126.9, 126.3, 126.2, 126.09, 126.0, 125.9, 125.7, 125.1, 125.0, 123.3, 122.6, 121.0, 120.9, 118.3, 118.2, 118.1, 118.0, 111.48, 111.4, 111.3, 110.8, 54.4, 50.9, 47.4, 46.8, 44.2, 44.0, 29.8, 29.1, 23.8, 23.2; **IR (ATR):** vmax 3237, 3050, 2921, 2455, 2114, 1921, 1674, 1627, 1455, 1365, 1197, 1123, 1009, 963, 816, 741; **HRMS (ESI):** *m/z* calcd for C_26_H_29_N_3_O [M]^+^: 400.2383; found: 400.2380.

***N*-(2-(1*H*-indol-3-yl)ethyl)-4-(dimethylamino)-*N*-(naphthalen-2-ylmethyl) butanamide (14b)**. The title compound **14b** was synthesized from compound **8** (0.4 g, 1.33 mmol) according to the protocol **H**. The product **14b** was obtained as a white solid (0.27 g, 49%); mp 108.6–109.2 °C; **^1^H NMR** (400 MHz, DMSO) δ 10.87–10.79 (s, NH-indole, 1H), 7.94–7.86 (m, ArH-naphthyl, 3H), 7.74–7.68 (m, ArH-naphthyl, 1H), 7.54–7.46 (m, ArH-naphthyl, 3H), 7.41–7.37 (m, ArH-indole, 1H), 7.35–7.31 (t, ArH-indole, 1H), 7.14–7.10 (s, ArH-indole, 1H), 7.09–7.02 (m, ArH-indole, 1H), 6.98–6.90 (m, ArH-indole, 1H), 4.77–4.74 (s, Ar-CH^1^H^2^*-*N-2CH_2_-(indole)N,N- dimethylbutan, 2H), 3.58–3.51 (m, Ar-CH_2_-N-CH_2_-CH_2_-(indole) N,N- dimethylbutane, 2H), 2.97–2.90 (m, γ-CH_2_ of N,N- dimethylbutane, 2H), 2.45–2.09 (m, Ar-CH_2_-N-CH_2_-CH_2_-indole- N,N- dimethylbutane, α-CH_2_ of N,N- dimethylbutane and N-CH_3_CH_3_ of N,N- dimethylbutane, 10H), 1.75–1.63 (p, β-CH_2_ of N,N- dimethylbutane, 2H); **^13^C NMR** (151 MHz, DMSO) δ 171.9, 136.2, 136.1, 136.0, 135.5, 132.9, 132.8, 132.2, 132.1, 128.3, 128.0, 127.6, 127.56, 127.5, 127.4, 127.1, 126.9, 126.3, 126.2, 126.08, 126.0, 125.8, 125.7, 125.1, 124.8, 123.2, 122.6, 121.0, 120.9, 118.3, 118.2, 118.17, 118.1, 111.5, 111.4, 111.3, 110.8, 57.8, 50.9, 47.6, 47.4, 46.8, 44.38, 44.3, 30.7, 29.9, 29.3, 24.0, 23.2, 22.0; **IR (ATR):** vmax 3109, 3057, 2774, 2455, 2343, 2114, 1799, 1635, 1455, 1413, 1338, 1218, 1137, 1017, 973, 818, 733; **HRMS (ESI):** *m/z* calcd for C_27_H_31_N_3_O [M]^+^: 414.2539; found: 414.2535.

**4-(Dimethylamino)-*N*-(naphthalen-2-ylmethyl)-*N*-phenethyl-butanamide (14c)**. The title compound **14c** was synthesized from compound **9** (0.4 g, 1.5 mmol) according to the protocol **H**. The product **14c** was obtained as a white solid (0.447 g, 78%); mp 46.6–48.2 °C; **^1^H NMR** (600 MHz, DMSO) δ 7.96–7.87 (m, ArH-naphthyl, 3H), 7.78–7.72 (s, ArH-naphthyl, 1H), 7.54–7.49 (m, ArH-naphthyl, 2H), 7.42–7.40 (m, ArH-naphthyl, 1H), 7.33–7.18 (m, ArH-phenyl, 5H), 4.75 (s, Ar-CH^1^H^2^*-*N-2CH_2_(phenyl)N,N- dimethylpropane, 2H), 3.56–3.51 (m, β-CH_2_ of N,N- dimethylpropane, 2H), 3.04–2.98 (t, Ar-CH_2_-N-CH^1^H^2^-CH_2_-(phenyl) N,N- dimethylpropane, 2H), 2.89–2.79 (m, Ar-CH_2_-N-CH_2_-CH_2_-phenyl- N,N- dimethylpropane, 2H), 2.76–2.65 (t, α-CH^1^H^2^ of N,N- dimethylpropane, 2H), 2.54–2.52 (s, N-CH_3_CH_3_ of N,N- dimethyl-propane, 6H); **^13^C NMR** (151 MHz, DMSO) δ 170.3, 170.2, 139.0, 138.6, 135.4, 135.0, 132.9, 132.8, 132.3, 132.2, 128.9, 128.6, 128.5, 128.4, 128.1, 127.64, 127.6, 127.56, 127.5, 126.47, 126.4, 126.3, 126.2, 126.0, 125.9, 125.8, 125.1, 124.8, 53.9, 53.8, 50.7, 48.3, 47.6, 47.5, 43.4, 33.9, 33.2, 28.8, 28.1; **IR (ATR):** vmax 3055, 2933, 2110, 1624, 1454, 1363, 1149, 1018, 957, 829, 747; **HRMS (ESI):** *m/z* calcd for C_24_H_28_N_2_O [M]^+^: 361.2274; found: 361.2271.

**3-(Dimethylamino)-*N*-(naphthalen-2-ylmethyl)-*N*-phenethyl-propanamide (14d)**

The title compound **14d** was synthesized from compound **9** (0.4 g, 1.5 mmol) according to the protocol **H**. The product **14d** was obtained as a white solid (0.447 g, 49%), mp 144.9–145.3 °C; **^1^H NMR** (600 MHz, DMSO) δ 7.95–7.87 (m, ArH-naphthyl, 3H), 7.76–7.69 (s, ArH-naphthyl, 1H), 7.53–7.48 (m, ArH-naphthyl, 2H), 7.41–7.38 (m, ArH-naphthyl, 1H), 7.33–7.18 (m, ArH-phenyl, 5H), 4.75–4.72 (s, Ar-CH^1^H^2^*-*N-2CH_2_-(phenyl)N,N- dimethylbutan, 2H), 3.56–3.48 (t, Ar-CH_2_-N-CH^1^H^2^-CH_2_-(phenyl) N,N- dimethylbutane 2H), 2.96–2.79 (m, γ-CH_2_ of N,N-dimethylbutane and Ar-CH_2_-N-CH_2_-CH_2_-phenyl-N,N-dimethylbutane, 4H), 2.74–2.70 (s, N-CH_3_CH_3_ of N,N- dimethylbutane, 6H), 2.47–2.38 (t, α-CH^1^H^2^ of N,N-dimethylbutane, 2H), 1.86–1.79 (m, β-CH_2_ of N,N- dimethylbutane, 2H); **^13^C NMR** (151 MHz, DMSO) δ 171.2, 171.1, 139.1, 138.6, 135.7, 135.2, 132.9, 132.8, 132.2, 132.1, 128.9, 128.6, 128.47, 128.4, 128.3, 128.1, 127.7, 127.6, 127.55, 127.5, 126.44, 126.4, 126.2, 126.1, 126.06, 126.0, 125.9, 125.7, 125.0, 124.7, 56.7, 56.6, 50.6, 48.2, 47.7, 47.6, 42.5, 42.4, 33.9, 33.3, 29.4, 28.8, 19.96, 19.9; **IR (ATR):** vmax 3026, 2934, 2776, 2486, 2113, 1602, 1475, 1369, 1281, 1204, 1174, 828, 752; **HRMS (ESI):** *m/z* calcd for C_25_H_30_N_2_O [M]^+^: 375.2431; found: 375.2431.

***N*-(2-(1*H*-indol-3-yl)ethyl)-3-(dimethylamino)-*N*-(naphthalen-2-ylmethyl) propenamide (15a)**. The title compound **15a** was prepared from compound **14a** (0.1 g, 0.25 mmol) according to the general procedure **I**. The product **15a** was obtained as a white gum (0.091 g, 91%); **^1^H NMR** (400 MHz, DMSO) δ 10.96–10.85 (s, NH-indole, 1H), 10.22–10.15 (s, H-N- 2CH_3_, 1H), 7.96–7.87 (m, ArH-naphthyl, 3H), 7.79–7.77 (m, ArH-naphthyl, 1H), 7.54–7.41 (m, ArH-naphthyl and ArH-indole, 4H), 7.36–7.31 (m, ArH-indole, 1H), 7.21–7.10 (s, ArH-indole, 1H), 7.07–7.02 (m, ArH-indole, 1H), 6.98–6.91 (m, ArH-indole, 1H), 4.80–4.79 (s, Ar-CH^1^H^2^*-*N-2CH_2_-(indole)N,N- dimethylpropane, 2H), 3.59–3.53 (m, β-CH^1^H^2^ of N,N- dimethylpropane, 2H), 3.37–3.18 (m, Ar-CH_2_-N-CH_2_-CH_2_-(indole) N,N-dimethylpropane, 2H), 3.03–2.98 (m, Ar-CH_2_-N-CH_2_-CH_2_-indole- N,N- dimethylpropane, 2H), 2.91–2.85 (m, α-CH_2_ of N,N- dimethylpropane, 2H), 2.77–2.63 (s, HN-CH_3_CH_3_ of N,N-dimethylpropane, 6H); **^13^C NMR** (151 MHz, DMSO) δ 169.4, 169.3, 136.2, 136.1, 135.4, 134.8, 133.0, 132.9, 132.3, 132.2, 128.4, 128.1, 127.68, 127.6, 127.57, 127.5, 127.0, 126.9, 126.4, 126.3, 126.2, 126.1, 126.0, 125.8, 125.44, 125.4, 123.5, 122.7, 121.0, 120.9, 118.4, 118.3, 118.2, 111.5, 111.4, 111.2, 110.7, 53.1, 52.9, 50.7, 47.6, 47.5, 46.6, 42.4, 42.3, 42.1, 42.0, 27.7, 26.9, 23.7, 23.1; **IR (ATR):** vmax 3393, 3219, 3049, 2917, 2848, 2672, 2467, 1620, 1456, 1363, 1228, 1144, 1007, 962, 816, 741; **HRMS (ESI):** *m/z* calcd for C_26_H_29_N_3_O [M]^+^: 400.2383; found: 400.2380.

***N*-(2-(1*H-*indol-3-yl)ethyl)-4-(dimethylamino)-*N*-(naphthalen-2-ylmethyl) butanamide (15b)**. The title compound **15b** was prepared from compound **14b** (0.1 g, 0.24 mmol) according to the general procedure **I**. The product **15b** was obtained as a colorless gum (0.1 g, 100%); **^1^H NMR** (600 MHz, DMSO) δ 10.95–10.84 (s, NH-indole, 1H), 10.56–10.50 (m, H-N-2CH_3_, 1H), 7.94–7.88 (m, ArH-naphthyl, 3H), 7.76–7.71 (s, ArH-naphthyl, 1H), 7.52–7.46 (m, ArH-naphthyl, 3H), 7.42–7.40 (m, ArH-indole, 1H), 7.36–7.31 (m, ArH-indole, 1H), 7.18–7.10 (s, ArH-indole, 1H), 7.08–7.03 (m, ArH-indole, 1H), 6.97–6.90 (t, ArH-indole, 1H), 4.78–4.76 (s, Ar-CH^1^H^2^*-*N-2CH_2_-(indole)N,N-dimethylbutan, 2H), 3.58–3.51 (t, Ar-CH_2_-N-CH^1^H^2^-CH_2_-(indole)-N,N- dimethylbutane, 2H), 3.04–2.96 (m, γ-CH_2_ of N,N- dimethylbutane, 2H), 2.91–2.81 (m, Ar-CH_2_-N-CH_2_-CH_2_-indole-N,N-dimethylbutane, 2H), 2.72–2.70 (s, HN-CH_3_CH_3_ of N,N- dimethylbutane, 6H), 2.55–2.42 (t, α-CH^1^H^2^ of N,N-dimethylbutane, 2H), 1.95–1.81 (m, β-CH_2_ of N,N-dimethylbutane, 2H); **^13^C NMR** (151 MHz, DMSO) δ 171.0, 136.2, 136.1, 135.8, 135.3, 133.0, 132.9, 132.3, 132.2, 128.3, 128.1, 127.6, 127.58, 127.5, 127.1, 127.0, 126.3, 126.2, 126.1, 125.9, 125.7, 125.2, 125.0, 123.3, 122.7, 121.0, 120.9, 118.4, 118.2, 118.1, 111.5, 111.42, 111.4, 110.8, 56.2, 56.1, 50.8, 47.6, 47.4, 46.8, 41.97, 41.9, 29.6, 28.9, 23.7, 23.2, 19.6, 19.4; **IR (ATR):** vmax 3249, 3049, 2964, 2691, 2343, 2109, 1612, 1456, 1363, 1228, 1009, 965, 815, 742; **HRMS (ESI):** *m/z* calcd for C_27_H_31_N_3_O [M]^+^: 414.2539; found: 414.2537.

**3-(Dimethylamino)-*N*-(naphthalen-2-ylmethyl)-*N*-phenethyl-propanamide(15c)**. The title compound **15c** was prepared from compound **14c** (0.1 g, 0.27 mmol) according to the general procedure **I**. The product **15c** was obtained as a white gum (0.1 g, 100%); **^1^H NMR** (600 MHz, DMSO) δ 9.54–9.47 (s, H-N-2CH_3_, 1H), 7.96–7.88 (m, ArH-naphthyl, 3H), 7.79–7.75 (s, ArH-naphthyl, 1H), 7.54–7.50 (m, ArH-naphthyl, 2H), 7.43–7.41 (m, ArH-naphthyl, 1H), 7.33–7.18 (m, ArH-phenyl, 5H), 4.77–4.76 (s, Ar-CH^1^H^2^*-*N-2CH_2_(phenyl)N,N- dimethylpropane, 2H), 3.55–3.51 (m, Ar-CH_2_-N-CH^1^H^2^-CH_2_-(phenyl) N,N- dimethylpropane, 2H), 3.33–3.26 (m, β-CH_2_ of N,N- dimethylpropane, 2H), 2.94–2.79 (m, Ar-CH_2_-N-CH_2_-CH_2_-phenyl-N,N-dimethylpropane and α-CH_2_ of N,N- dimethylpropane, 4H), 2.76–2.75 (s, HN-CH_3_CH_3_ of N,N- dimethyl-propane, 6H); **^13^C NMR** (151 MHz, DMSO) δ 169.6, 169.5, 138.9, 138.5, 135.3, 134.7, 133.0, 132.8, 132.3, 132.2, 128.9, 128.59, 128.5, 128.4, 128.1, 127.67, 127.6, 127.56, 127.5, 126.49, 126.4, 126.29, 126.2, 126.1, 126.0, 125.8, 125.2, 125.0, 53.18, 53.1, 50.6, 48.3, 47.6, 47.5, 42.5, 42.4, 33.8, 33.1, 27.6, 27.0; **IR (ATR):** vmax 3394, 3059, 2930, 2105, 1613, 1471, 1364, 1273, 1240, 1161, 1061, 948, 829, 754; **HRMS (ESI):** *m/z* calcd for C_24_H_28_N_2_O [M]^+^: 361.2274; found: 361.2274.

**4-(Dimethylamino)-*N*-(naphthalen-2-ylmethyl)-*N*-phenethyl-butanamide (15d)**

The title compound **15d** was prepared from compound **14d** (0.08 g, 0.2 mmol) according to the general procedure **I**. The product **15d** was obtained as a white solid (0.08 g, 100%); mp 131.7–132.9 °C; **^1^H NMR** (600 MHz, DMSO) δ 9.43–9.38 (s, H-N-2CH_3_, 1H), 7.95–7.88 (m, ArH-naphthyl, 3H), 7.77–7.70 (s, ArH-naphthyl, 1H), 7.53–7.49 (m, ArH-naphthyl, 2H), 7.41–7.38 (m, ArH-naphthyl, 1H), 7.33–7.18 (m, ArH-phenyl, 5H), 4.75–4.72 (s, Ar-CH^1^H^2^*-*N-2CH_2_-(phenyl)N,N-dimethylbutan, 2H), 3.56–3.48 (t, Ar-CH_2_-N-CH^1^H^2^-CH_2_-(phenyl) N,N- dimethylbutane, 2H), 3.03–2.96 (m, γ-CH_2_ of N,N-dimethylbutane, 2H), 2.88–2.80 (m, Ar-CH_2_-N-CH_2_-CH_2_-phenyl- N,N- dimethylbutane, 2H), 2.79–2.75 (s, HN-CH_3_CH_3_ of N,N- dimethylbutane, 6H), 2.49–2.40 (t, α-CH^1^H^2^ of N,N- dimethylbutane, 2H), 1.89–1.81 (m, β-CH_2_ of N,N-dimethylbutane, 2H); **^13^C NMR** (151 MHz, DMSO) δ 171.1, 171.0, 139.1, 138.6, 135.7, 135.2, 132.9, 132.8, 132.2, 132.1, 128.9, 128.6, 128.47, 128.4, 128.3, 128.1, 127.6, 127.59, 127.5, 126.44, 126.4, 126.2, 126.1, 126.06, 126.0, 125.9, 125.7, 125.0, 124.7, 56.5, 56.4, 50.6, 48.2, 47.7, 47.6, 42.3, 42.2, 33.9, 33.3, 29.3, 28.7, 19.7, 19.6; **IR (ATR):** vmax 3021, 2776, 2342, 2106, 1620, 1459, 1413, 1360, 1280, 1195, 1160, 827, 756; **HRMS (ESI):** *m/z* calcd for C_25_H_30_N_2_O [M]^+^: 375.2431; found: 375.2424.

**3-((2-(1*H-*indol-3-yl) ethyl) (naphthalen-2-ylmethyl) amino)-*N,N,N*-trimethyl-3-oxopropan-1-aminium (16a)**. The title compound **16a** was prepared from compound **14a** (0.05 g, 0.13 mmol) according to the general procedure **J**. The product **16a** was obtained as a yellow solid (0.043g, 82%) mp 98.3–99.5 °C; **^1^H NMR** (600 MHz, DMSO) δ 10.91–10.79 (s, NH-indole, 1H), 7.97–7.87 (m, ArH-naphthyl, 3H), 7.79–7.75 (m, ArH-naphthyl, 1H), 7.56–7.50 (m, ArH-naphthyl, 3H), 7.43–7.41 (m, ArH-indole, 1H), 7.37–7.31 (m, ArH-indole, 1H), 7.18–7.11 (m, ArH-indole,1H), 7.09–7.03 (m, ArH-indole, 1H), 6.99–6.88 (m, ArH-indole, 1H), 4.83–4.80 (m, Ar-CH_2_*-*N-2CH_2_-(indole)N,N,N- trimethylpropan, 2H), 3.70–3.53 (m, β-CH_2_ of N,N,N- trimethylpropane, 2H), 3.44–3.21 (m, Ar-CH_2_-N-CH_2_-CH_2_-(indole) N,N,N- trimethylpropane, 2H), 3.08–2.66 (m, N-3CH_3_, Ar-CH_2_-N-CH_2_-CH_2_-indole- N,N,N- trimethylpropane and α-CH_2_ of N,N,N- trimethylpropane, 13H); **^13^C NMR** (151 MHz, DMSO) δ 169.6, 169.5, 168.5, 136.2, 136.1, 135.4, 135.3, 134.9, 134.8, 132.99, 132.9, 132.8, 132.4, 132.3, 132.2, 128.4, 128.3, 128.2, 128.1, 127.64, 127.6, 127.57, 127.5, 127.1, 127.0, 126.9, 126.5, 126.4, 126.3, 126.29, 126.2, 126.1, 126.06, 126.0, 125.8, 125.5, 125.5, 125.2, 125.1, 123.7, 123.4, 122.72, 122.7, 121.1, 120.9, 118.5, 118.4, 118.3, 118.2, 111.6, 111.5, 111.4, 111.3, 111.2, 110.8, 110.7, 61.9, 61.6, 53.3, 53.2, 52.4, 52.2, 52.16, 52.1, 50.7, 47.6, 47.4, 47.3, 47.0, 46.8, 46.4, 42.6, 42.3, 27.6, 26.8, 26.6, 25.9, 23.6, 23.4, 23.1, 23.0; **IR (ATR):** vmax 3386, 3248, 2919, 2704, 2321, 1622, 1452, 1363, 1230, 1158, 1092, 920, 820, 741; **HRMS (ESI):** *m/z* calcd for C_27_H_32_N_3_O [M] ^+^: 414.2539; found: 414.2533.

**4-((2-(1*H*-indol-3-yl) ethyl) (naphthalen-2-ylmethyl) amino)-*N,N,N*-trimethyl-4-oxobutan-1-aminium (16b)**. The title compound **16b** was prepared from compound **14b** (0.06 g, 0.14 mmol) according to the general procedure **J**. The product **16b** was obtained as a white solid (0.06 g, 97%); mp 92.3–93.1 °C; **^1^H NMR** (600 MHz, DMSO) δ 10.88–10.79 (s, NH-indole, 1H), 7.95–7.87 (m, ArH-naphthyl, 3H), 7.77–7.70 (m, ArH-naphthyl, 1H), 7.53–7.48 (m, ArH-naphthyl, 3H), 7.43–7.40 (m, ArH-indole, 1H), 7.37–7.32 (m, ArH-indole, 1H), 7.16–7.11 (s, ArH-indole, 1H), 7.10–7.04 (m, ArH-indole, 1H), 6.99–6.91 (m, ArH-indole, 1H), 4.80–4.77 (s, Ar-CH^1^H^2^*-*N-2CH_2_-(indole)N,N,N- trimethylbutan, 2H), 3.62–3.52 (m, Ar-CH_2_-N-CH_2_-CH_2_-(indole) N,N,N- trimethylbutane, 2H), 3.40–3.23 (m, γ-CH_2_ of N,N,N- trimethylbutane, 2H), 3.06–2.75 (m, N-3CH_3_ and Ar-CH_2_-N-CH_2_-CH_2_-indole- N,N,N- trimethyl butane, 11H), 2.53–2.39 (m, α-CH_2_ of N,N,N- trimethylbutane, 2H), 2.00–1.75 (m, β-CH_2_ of N,N,N- trimethylbutane 2H); **^13^C NMR** (151 MHz, DMSO) δ 171.0, 170.8, 170.7, 136.2, 136.1, 135.7, 135.2, 132.9, 132.8, 132.3, 132.2, 128.4, 128.1, 127.6, 127.58, 127.54, 127.5, 127.1, 126.9, 126.4, 126.2, 126.1, 126.0, 125.9, 125.7, 125.0, 124.8, 124.7, 123.3, 123.2, 122.6, 121.0, 120.9, 118.4, 118.2, 118.1, 111.5, 111.3, 110.9, 110.8, 64.9, 56.5, 52.24, 52.2, 52.1, 50.8, 47.6, 47.3, 47.0, 42.4, 42.3, 29.1, 28.4, 23.7, 23.2, 18.2, 18.0; **IR (ATR):** vmax 3426, 3241, 3048, 2933, 2704, 2303, 1622, 1455, 1364, 1227, 1159, 1020, 954, 819, 746; **HRMS (ESI):** *m/z* calcd for C_28_H_34_N_3_O [M] ^+^: 428.2696; found: 428.2689.

***N,N,N*-Trimethyl-3-((naphthalen-2-ylmethyl)(phenethyl)amino)-3-oxopropan-1-aminium (16c)**. The title compound **16c** was prepared from compound **14c** (0.1 g, 0.27 mmol) according to the general procedure **J**. The product **16c** was obtained as a yellowish solid (0.1 g, 98%); mp 50.2–52.1 °C; **^1^H NMR** (400 MHz, DMSO) 7.97–7.87 (m, ArH-naphthyl, 3H), 7.79–7.74 (m, ArH-naphthyl, 1H), 7.55–7.40 (m, ArH-naphthyl, 3H), 7.34–7.17 (m, ArH-phenyl, 5H), 4.80–4.76 (m, Ar-CH_2_*-*N-2CH_2_-(phenyl)N,N,N- trimethylpropan, 2H), 3.67–3.26 (m, Ar-CH_2_-N-CH_2_-CH_2_-(phenyl)N,N,N-trimethylpropane and β-CH_2_ of N,N,N- trimethylpropane, 4H), 3.06–2.76 (m, N-3CH_3_, Ar-CH_2_-N-CH_2_-CH_2_-indole-N,N,N-trimethylpropane and α-CH_2_ of N,N,N- trimethylpropane, 13H); **^13^C NMR** (151 MHz, DMSO) δ 169.7, 169.6, 168.5, 139.0, 138.9, 138.6, 138.5, 135.3, 135.2, 134.8, 134.7, 132.9, 132.8, 132.3, 132.3, 132.2, 129.0, 128.9, 128.58, 128.5, 128.43, 128.4, 128.1, 128.0, 127.64, 127.6, 127.55, 127.52, 126.5, 126.48, 126.4, 126.3, 126.25, 126.2, 126.15, 126.1, 126.08, 126.0, 125.8, 125.4, 125.3, 125.1, 124.9, 61.7, 53.28, 53.25, 52.46, 52.44, 52.4, 50.59, 50.5, 48.2, 47.9, 47.6, 47.58, 47.5, 47.0, 42.6, 42.5, 33.75, 33.7, 33.1, 33.0, 27.6, 26.9, 26.6, 26.0; **IR (ATR):** vmax 3053, 2922, 2682, 2320, 1625, 1454, 1363, 1236, 1150, 950, 829, 743; **HRMS (ESI):** *m/z* calcd for C_25_H_31_N_2_O [M] ^+^: 375.2431; found: 375.2428.

***N,N,N*-Trimethyl-4-((naphthalen-2-ylmethyl)(phenethyl)amino)-4-oxobutan-1-aminium (16d)**. The title compound **16d** was prepared from compound **14d** (0.1 g, 0.26 mmol) according to the general procedure **J**. The product 2–19 was obtained as a yellowish gum (0.097 g, 96%); mp 46.7–48.1 °C; **^1^H NMR** (600 MHz, DMSO) δ 7.95–7.87 (m, ArH-naphthyl, 3H), 7.77–7.70 (s, ArH-naphthyl, 1H), 7.54–7.50 (m, ArH-naphthyl, 2H), 7.41–7.38 (m, ArH-naphthyl, 1H), 7.33–7.18 (m, ArH-phenyl, 5H), 4.75–4.73 (s, Ar-CH^1^H^2^*-*N-2CH_2_-(phenyl)N,N,N- trimethylbutan, 2H), 3.57–3.48 (t, Ar-CH_2_-N-CH^1^H^2^-CH_2_-(phenyl)N,N,N-trimethylbutane, 2H), 3.06–2.74 (m, N-3CH_3_, γ-CH_2_ of N,N,N- trimethylbutane and Ar-CH_2_-N-CH_2_-CH_2_-phenyl- N,N,N- trimethylbutane, 13H), 2.48–2.39 (t, α-CH^1^H^2^ of N,N,N- trimethylbutane, 2H), 1.87–1.80 (m, β-CH_2_ of N,N,N- trimethylpropane, 2H); **^13^C NMR** (151 MHz, DMSO) δ 171.1, 171.0, 139.1, 138.6, 135.7, 135.2, 132.9, 132.8, 132.2, 132.1, 128.9, 128.6, 128.47, 128.4, 128.3, 128.1, 127.6, 127.58, 127.54, 127.53, 126.4, 126.4, 126.2, 126.1, 126.06, 126.0, 125.9, 125.8, 125.0, 124.7, 56.6, 56.5, 52.2, 50.6, 48.2, 47.69, 47.6, 42.3, 42.2, 33.9, 33.3, 29.3, 28.7, 19.7, 19.6, 18.0; **IR (ATR):** vmax 3435, 3026, 2928, 2703, 2460, 1601, 1474, 1367, 1291, 1228, 1173, 1031, 964, 828, 751; **HRMS (ESI):** *m/z* calcd for C_26_H_33_N_2_O [M] ^+^: 389.2587; found: 389.2579.

**Di-*tert*-butyl(5-((2-(1*H*-indol-3-yl)ethyl)(naphthalen-2-ylmethyl)amino)-5-oxopentane-1,4-diyl) *(S*)-dicarbamate (18a)**. The title compound **18a** was synthesized from compound **8** (0.5 g, 1.6 mmol) and Boc-Orn(Boc)-OH (0.55 g, 1.6 mmol) according to the protocol **C**. The product **18a** was obtained as a white solid (0.84 g, 82%); mp 68.1–70.2 °C; **^1^H NMR** (600 MHz, DMSO) δ 10.87–10.77 (s, NH-indole, 1H), 7.91–7.82 (m, ArH-naphthyl, 3H), 7.77–7.73 (s, ArH-naphthyl, 1H), 7.52–7.46 (m, ArH-naphthyl, 3H), 7.34–7.33 (d, α-CH-NH-Boc, 1H), 7.31–7.30 (m, ArH-indole, 1H), 7.17–7.04 (m, ArH-indole, 3H), 6.96–6.88 (m, ArH-indole, 1H), 6.65–6.72 (s, δ-CH_2_-NH-Boc, 1H), 4.86–4.60 (s, Ar-CH_2_*-*N-2CH_2_-(indole)- Boc-Orn(Boc), 2H), 4.52–4.38 (m, α-CH of Boc-Orn(Boc), 1H), 3.78–3.38 (m, Ar-CH_2_-N-CH_2_-CH_2_-indole, 2H), 3.05–2.79 (m, δ-CH_2_- of Boc-Orn(Boc) and Ar-CH_2_-N-CH_2_-CH_2_-indole 4H), 1.64–1.18 (m, β-CH_2_, γ-CH_2_, of Boc-Orn(Boc) and 2(CH_3_)_3_ of Boc-Orn(Boc), 22H); **^13^C NMR** (151 MHz, DMSO) δ 172.7, 171.8, 155.6, 155.5, 155.4, 136.2, 135.6, 135.0, 132.9, 132.8, 132.3, 132.1, 128.1, 128.0, 127.58, 127.5, 127.4, 127.0, 126.9, 126.2, 126.1, 125.8, 125.77, 125.74, 125.7, 125.5, 123.1, 122.6, 120.99, 120.9, 118.3, 118.2, 118.15, 118.1, 111.4, 111.39, 111.3, 110.8, 79.1, 78.0, 77.9, 77.3, 50.4, 50.3, 50.2, 48.1, 47.5, 46.2, 29.1, 28.9, 28.2, 28.1, 25.9, 25.8, 24.4, 22.8; **IR (ATR):** vmax 3297, 2972, 2927, 2320, 2101, 1688, 1627, 1502, 1451, 1363, 1245, 1159, 1011, 857, 813, 739; **HRMS (ESI):** *m/z* calcd for C_36_H_46_N_4_O_5_ [M + Na]^+^: 637.3361; found: 637.3363.

**Di-*tert*-butyl(6-((2-(1*H*-indol-3-yl) ethyl) (naphthalen-2-ylmethyl) amino)-6-oxohexane-1,5-diyl) *(S)-*dicarbamate (18b)**. The title compound **18b** was synthesized from compound **8** (0.2 g, 0.66 mmol) and Boc-Lys(Boc)-OSu (0.29 g, 0.66 mmol) according to the protocol **E**. The product **18b** was obtained as a white solid (0.127 g, 63%); mp 51.2–53.6 °C; **^1^H NMR** (600 MHz, DMSO) δ 10.86–10.76 (s, NH-indole, 1H), 7.92–7.82 (m, ArH-naphthyl, 3H), 7.77–7.69 (d, ArH-naphthyl, 1H), 7.52–7.44 (m, ArH-naphthyl, 3H), 7.35–7.33 (m, α-CH-NH-Boc, 1H), 7.32–7.30 (m, ArH-indole, 1H), 7.15–7.03 (m, ArH-indole, 3H), 6.97–6.88 (m, ArH-indole, 1H), 6.76–6.70 (t, ε-CH_2_-NH-Boc, 1H), 4.84–4.69 (m, Ar-CH_2_*-*N-2CH_2_-(indole)- Boc-Lys(Boc), 2H), 4.46–4.35 (t, α-CH of Boc-Lys(Boc), 1H), 3.80–3.35 (m, Ar-CH_2_-N-CH_2_-CH_2_-indole, 2H), 3.05–2.81 (m, ε-CH_2_- of Boc-Lys(Boc) and Ar-CH_2_-N-CH_2_-CH_2_-indole, 4H), 1.54–1.24 (m, β-CH_2_, γ-CH_2_, δ-CH_2_ of Boc-Lys(Boc) and 2(CH_3_)_3_ of Boc-Lys(Boc), 24H); **^13^C NMR** (151 MHz, DMSO) δ 172.8, 172.0, 155.7, 155.5, 155.4, 136.1, 135.7, 135.1, 132.9, 132.8, 132.3, 132.1, 128.1, 127.9, 127.56, 127.54, 127.4, 127.0, 126.9, 126.2, 126.1, 125.8, 125.75, 125.7, 125.6, 125.5, 123.2, 122.6, 120.99, 120.9, 118.3, 118.2, 118.1, 118.0, 111.4, 111.39, 111.3, 110.8, 78.0, 77.9, 50.6, 50.5, 50.3, 48.0, 47.5, 46.3, 33.3, 31.3, 31.0, 29.3, 29.2, 28.26, 28.2, 28.1, 24.4, 22.8, 22.6; **IR (ATR):** vmax 3308, 2929, 1688, 1631, 1506, 1453, 1364, 1245, 1161, 1011, 859, 814, 740; **HRMS (ESI):** *m/z* calcd for C_37_H_48_N_4_O_5_ [M + Na]^+^: 651.3516; found: 651.3512.

***(S)*-*N*-(2-(1*H*-indol-3-yl)ethyl)-2,5-diamino-*N*-(naphthalen-2-ylmethyl) pentanamide (TFA salt) (19a)**. The title compound **19a** was synthesized from compound **18a** (0.2 g, 0.32 mmol) according to the protocol **F**. The product **19a** was obtained as a yellowish gum (0.076 g, 57%); **^1^H NMR** (400 MHz, DMSO) δ 10.93–10.83 (s, NH-indole, 1H), 8.39 -8.36 (t, α-CH-**^+^**NH_3_ CF_3_COO^−^, 3H), 7.99–7.78 (m, δ-CH_2_-**^+^**NH_3_ CF_3_COO^−^ and ArH-naphthyl, 7H), 7.57–7.53 (m, ArH-naphthyl, 2H), 7.48–7.42 (m, ArH-naphthyl and ArH-indole, 2H), 7.36–7.31 (m, ArH-indole, 1H), 7.21–7.09 (m, ArH-indole, 1H), 7.07–7.03 (m, ArH-indole, 1H), 6.96–6.88 (m, ArH-indole, 1H), 5.00–4.92 (s, Ar-CH^1^H^2^*-*N-2CH_2_-(indole)- Orn, 1H), 4.79–4.62 (s, Ar-CH^1^H^2^*-*N-2CH_2_-(indole)- Orn, 1H), 4.59–4.43 (m, α-CH of Orn, 1H), 3.81–3.24 (m, Ar-CH_2_-N-CH_2_-CH_2_-indole-Orn, 2H), 3.04–2.74 (m, Ar-CH_2_-N-CH_2_-CH_2_-indole-Orn and δ-CH_2_- of Orn, 4H), 1.81–1.61 (m, β-CH_2_,γ-CH_2_, of Orn, 4H); **^13^C NMR** (151 MHz, DMSO) δ 167.8, 167.5, 157.5, 157.3, 157.1, 156.8, 135.2, 135.1, 133.6, 133.1, 131.9, 131.8, 131.4, 131.3, 127.39, 127.3, 126.65, 126.6, 126.5, 125.9, 125.7, 125.5, 125.3, 125.2, 125.0, 124.98, 124.9, 124.5, 122.3, 121.8, 120.1, 120.0, 118.5, 117.4, 117.2, 117.1, 117.0, 116.6, 114.6, 112.6, 110.5, 110.4, 109.8, 109.3, 49.4, 48.5, 48.4, 47.0, 46.3, 45.3, 38.3, 38.2, 38.1, 37.4, 37.3, 27.0, 26.8, 23.2, 21.8, 21.6, 21.2; **IR (ATR):** vmax 3405, 3043, 2931, 2082, 1655, 1524, 1430, 1367, 1176, 1126, 797, 743; **HRMS (ESI):** *m/z* calcd for C_26_H_30_N_4_O [M]+: 415.2492; found: 415.2488. 

***(S)*-*N*-(2-(1*H*-indol-3-yl) ethyl)-2,6-diamino-*N*-(naphthalen-2-ylmethyl) hexanamide (TFA salt) (19b)**. The title compound **19b** was synthesized from compound **18b** (0.1 g, 0.15 mmol) according to the protocol **F**. The product **19b** was obtained as a brown gum (0.06 g, 89%); **^1^H NMR** (600 MHz, DMSO) δ 10.94–10.86 (s, NH-indole, 1H), 8.34–8.32 (s, α-CH-**^+^**NH_3_ CF_3_COO^−^, 3H), 7.98–7.85 (m, ε-CH_2_-**^+^**NH_3_ CF_3_COO^−^ and ArH-naphthyl, 6H), 7.80–7.97 (d, ArH-naphthyl, 1H), 7.56–7.51 (m, ArH-naphthyl 2H), 7.48–7.42 (m, ArH-naphthyl and ArH-indole, 2H), 7.37–7.32 (m, ArH-indole, 1H), 7.21–7.11 (dd, ArH-indole, 1H), 7.08–7.05 (m, ArH-indole, 1H), 6.95–6.89 (dt, ArH-indole, 1H), 4.95–4.92 (s, Ar-CH^1^H^2^*-*N-2CH_2_-(indole)- Lys, 1H), 4.76–4.62 (s, Ar-CH^1^H^2^*-*N-2CH_2_-(indole)- Lys, 1H), 4.47–4.33 (m, α-CH of Lys, 1H), 3.84–3.27 (m, Ar-CH_2_-N-CH_2_-CH_2_-indole-Lys, 2H), 3.07–3.82 (m, Ar-CH_2_-N-CH_2_-CH_2_-indole-Lys, 2H), 2.74–2.68 (m, ε-CH2-Lys, 2H), 1.76–1.62 (m, β-CH_2_ of Lys, 2H), 1.56–1.44 (m, δ-CH_2_ of Lys, 2H), 1.41–1.23 (m, γ-CH_2_ of Lys, 2H); **^13^C NMR** (151 MHz, DMSO) δ 168.9, 168.7, 158.4, 158.2, 158.0, 157.8, 136.2, 136.1, 134.8, 134.1, 132.9, 132.8, 132.4, 132.3, 128.3, 128.2, 127.6, 127.59, 127.5, 127.0, 126.8, 126.5, 126.4, 126.3, 126.1, 126.0, 125.8, 125.4, 123.3, 122.9, 121.1, 121.0, 119.7, 118.4, 118.2, 118.0, 117.7, 115.8, 113.8, 111.5, 111.4, 110.8, 110.3, 50.2, 49.7, 49.5, 48.0, 47.4, 46.0, 39.9, 39.8, 39.6, 39.5, 39.3, 39.2, 39.1, 38.4, 38.3, 30.3, 30.2, 26.6, 26.5, 24.1, 22.6, 21.0, 20.7; **IR (ATR):** vmax 3399, 3049, 2935, 2088, 1774, 1654, 1523, 1430, 1367, 1127, 798, 743; **HRMS (ESI):** *m/z* calcd for C_27_H_32_N_4_O [M]+: 429.2648; found: 429.2644.

***N*-(2-(1*H*-indol-3-yl) ethyl)-2,5-diguanidino-*N*-(naphthalen-2-ylmethyl) pentanamide (TFA salt) (20a)**. The title compound **20a** was synthesized from compound **19a** (0.1 g, 0.24 mmol) according to the protocol **G**. The product **20a** was obtained as a white gum (0.045 g, 38%); **^1^H NMR** (600 MHz, DMSO) δ 10.93–10.79 (s, NH-indole, 1H), 8.32–8.28 (dd, **^+^**NH_3_ CF_3_COO^−^, 1H), 7.98–7.86 (m, ArH-Nph, 3H), 7.81–7.70 (m, ArH-naphthyl NH-guanidine, 3H), 7.57–7.50 (m, ArH-naphthyl, 2H), 7.46–7.31 (m, ArH-naphthyl NH-guanidine, 3H), 7.20–7.02 (m, NH-guanidine and ArH-indole, 5H), 6.95–6.87 (m, ArH-indole, 1H), 5.03–4.42 (m, Ar-CH_2_*-*N-2CH_2_-(indole), and α-CH of diguanidine, 3H), 3.82–2.78 (m, δ-CH_2_- of digunidine, Ar-CH_2_-N-CH_2_-CH_2_-indole and Ar-CH_2_-N-CH_2_-CH_2_-indole, 6H), 1.78–1.42 (m, β-CH_2_,γ-CH_2_, of diguanidine, 4H); **^13^C NMR** (151 MHz, DMSO) δ 169.8, 169.6, 158.5, 158.3, 158.1, 157.9, 156.7, 156.09, 156.0, 136.2, 136.1, 134.9, 134.1, 132.9, 132.8, 132.4, 132.2, 128.5, 128.3, 127.6, 127.58, 127.5, 126.9, 126.8, 126.6, 126.42, 126.4, 126.2, 126.0, 125.7, 125.7, 125.2, 123.2, 122.7, 121.1, 121.0, 118.3, 118.2, 118.1, 116.2, 111.5, 111.4, 110.9, 110.3, 50.5, 50.2, 50.1, 47.9, 47.1, 46.2, 30.0, 29.8, 24.2, 24.1, 23.8, 22.8; **IR (ATR):** vmax 3344, 3183, 2321, 1620, 1427, 1365, 1128, 799, 743, 720; **HRMS (ESI):** *m/z* calcd for C_28_H_34_N_8_O [M]^+^: 499.2928; found: 499.2921.

***(S)*-*N*-(2-(1*H*-indol-3-yl)ethyl)-2,6-diguanidino-*N*-(naphthalen-2-ylmethyl)hexanamide (TFA salt) (20b)**. The title compound **20b** was synthesized from compound **19b** (0.2 g, 0.46 mmol) according to the protocol **G**. The product **20b** was obtained as a yellowish solid (0.09 g, 39%); mp 48.2–50.6 °C; **^1^H NMR** (600 MHz, DMSO) δ 10.93–10.82 (s, NH-indole, 1H), 7.97–7.86 (m, ArH-naphthyl, 3H), 7.79–7.65 (m, ArH-naphthyl and NH-guanidine, 4H), 7.56–7.50 (m, ArH-naphthyl and NH-guanidine, 3H), 7.46–7.31 (m, ArH-naphthyl and ArH-indole, 4H), 7.18–7.03 (m, ArH-indole, 3H), 6.95–6.89 (m, ArH-indole, 1H), 4.97–4.69 (m, Ar-CH_2_*-*N-2CH_2_-(indole), and α-CH of diguanidine, 3H), 3.61–3.42 (m, ε-CH_2_- of digunidine, 2H), 3.08–2.82 (m, Ar-CH_2_-N-CH_2_-CH_2_-indole and Ar-CH_2_-N-CH_2_-CH_2_-indole, 4H), 1.71–1.24 (m, β-CH_2_, δ-CH_2_ and γ-CH_2_ of diguanidine_,_ 6H); **^13^C NMR** (151 MHz, DMSO) δ 170.0, 169.8, 158.7, 158.4, 158.2, 158.0, 156.7, 156.1, 156.0, 136.2, 136.1, 134.9, 134.2, 132.89, 132.8, 132.3, 132.2, 128.4, 128.2, 127.63, 127.6, 127.57, 127.5, 127.0, 126.8, 126.5, 126.37, 126.3, 126.1, 125.9, 125.8, 125.5, 125.1, 123.2, 122.8, 121.0, 120.9, 119.7, 118.3, 118.2, 118.1, 117.7, 115.7, 113.8, 111.48, 111.4, 111.0, 110.3, 50.5, 50.45, 50.4, 47.8, 47.0, 46.3, 40.7, 40.6, 32.3, 32.2, 28.48, 28.4, 24.1, 22.7, 21.4, 21.1; **IR (ATR):** vmax 3342, 3186, 2947, 2323, 2112, 1619, 1427, 1365, 1177, 1130, 800, 720; **HRMS (ESI):** *m/z* calcd for C_29_H_36_N_8_O [M]^+^: 512.3084; found: 512.3077.

***Tert*-butyl(S)-(1-((2-(1*H*-indol-3-yl) ethyl) (naphthalen-2-ylmethyl) amino)-1-oxo-5-(3-((2,2,4,6,7-pentamethyl-2,3-dihydrobenzofuran-5-yl) sulfonyl)guanidino) pentan-2-yl) carbamate (21)**. The title compound 21 was synthesized from compound 8 (0.2 g, 0.66 mmol) and Fmoc-Arg (Pbf)-OH (0.43 g, 0.66 mmol) according to the protocol C. The product 21 was obtained as a beige solid (0.84 g, 82%); mp 117.5–119.2 °C; ^1^H NMR (600 MHz, DMSO) δ 10.82–10.76 (s, NH-indole, 1H), 7.90–7.69 (m, ArH and α-CH-NH-Fmoc, 9H), 7.49–7.40 (m, ArH, 5H), 7.35–7.24 (m, ArH and NH-guanidine, 4H), 7.12–7.02 (m, ArH-indole, 2H), 6.93–6.88 (m, ArH-indole, 1H), 6.63 (s, NH-guanidine, 1H), 6.39 (s, NH-guanidine, 1H), 4.84–4.64 (m, Ar-CH_2_-N-2CH_2_-(indole)-Fmoc-Arg(Pbf), 2H), 4.54–4.45 (m, α-CH of Fmoc-Arg(Pbf),1H), 4.33–4.15 (m, NH-(C=O)-O-CH_2_-fluorenyl, 2H), 3.71–3.32 (m, CH_2_ of fluorenyl five ring and Ar-CH_2_-N-CH_2_-CH_2_-indole, 4H), 3.02–2.79 (m, CH_2_ of furan, δ-CH_2_- of Fmoc-Arg(Pbf) and Ar-CH_2_-N-CH_2_-CH_2_-indole, 6H), 2.48–2.42 (s, 2CH_3_ of benzofuran benzene ring, 6H), 1.96 (s, CH_3_ of benzofuran benzene ring, 3H), 1.62–1.21 (m, β-CH_2_, γ-CH_2_ of Fmoc-Arg(Pbf) and 2CH_3_ of furan, 10H); ^13^C NMR (151 MHz, DMSO) δ 172.0, 171.5, 157.4, 156.1, 156.0, 143.8, 143.77, 143.7, 140.7, 137.2, 136.2, 136.1, 135.5, 132.8, 132.3, 132.1, 131.4, 128.9, 128.2, 128.1, 127.6, 127.5, 127.4, 127.3, 127.0, 126.9, 126.3, 126.1, 125.9, 125.8, 125.7, 125.3, 124.3, 123.2, 122.7, 121.3, 121.0, 120.9, 120.1, 120.0, 118.3, 118.2, 118.1, 118.0, 116.2, 111.4, 111.3, 110.7, 86.2, 65.8, 50.5, 48.0, 47.4, 46.66, 46.6, 42.3, 29.0, 28.2, 24.3, 22.9, 18.9, 17.5, 12.2; **IR (ATR):** vmax 3330, 2928, 2321, 2112, 1712, 1621, 1545, 1449, 1241, 1089, 812, 728; **HRMS (ESI):** *m/z* calcd for C_55_H_58_N_6_O_6_S [M]^+^: 931.4211; found: 931.4207.

***(S)-N*-(2-(1*H*-indol-3-yl) ethyl)-2-amino-*N*-(naphthalen-2-ylmethyl)-5-(3-((2,2,4,6,7-pentamethyl-2,3-dihydrobenzofuran-5-yl) sulfonyl) guanidino)pentanamide (22)**. Compound **21** (0.25 g, 0.27 mmol) was added to a solution of 1.1 mL of piperidine in 4 mL of DMF and the reaction mixture was left to stir overnight. It was then extracted with ethyl acetate, H_2_O, and brine and the extract was dried over NaSO_4_. Filtration and evaporation of the solvent in vacuo gave the crude product which was purified by flash chromatography using DCM: MeOH 2–7%. The pure compound was dried under vacuum to give a white solid (0.14 g, 74%); mp 112.3–113.1 °C; **^1^H NMR** (600 MHz, DMSO) δ 10.85 -10-77 (s, NH-indole, 1H), 7.92–7.83 (m, ArH-naphthyl, 3H), 7.72 -7.70 (m, ArH-naphthyl, 1H), 7.52–7.47 (m, ArH-naphthyl, 2H), 7.47–7.43 (m, ArH-naphthyl, 1H), 7.41–7.35 (m, ArH-indole, 1H), 7.34–7.31 (m, ArH-indole, 1H), 7.14–7.08 (s, ArH-indole, 1H), 7.07–7.04 (m, ArH-indole, 1H), 6.95–6.89 (t, ArH-indole, 1H), 6.67–6.54 (d, NH-guanidine, 2H), 4.90–4.88 (s, Ar-CH^1^H^2^*-*N-2CH_2_-(indole)-Arg(Pbf), 1H), 4.83–4.80 (s, Ar-CH^1^H^2^*-*N-2CH_2_-(indole)-Arg(Pbf), 1H), 3.76–3.66 (m, α-CH of Arg(Pbf), 1H), 3.54–3.29 (m, Ar-CH_2_-N-CH^1^H^2^-CH_2_-indole-Arg(Pbf), 2H), 3.06–2.80 (m, δ-CH_2_- of Arg(Pbf) Ar-CH_2_-N-CH_2_-CH_2_-indole-Arg(Pbf) and CH_2_ of furan, 6H), 2.47 (s, CH_3_ of benzofuran benzene ring, 3H), 2.42 (s, CH_3_ of benzofuran benzene ring, 3H), 1.97 (s, CH_3_ of benzofuran benzene ring, 3H), 1.58–1.23 (m, β-CH_2_,γ-CH_2_ of Arg(Pbf) and 2CH_3_ of furan, 10H); **^13^C NMR** (151 MHz, DMSO) δ 157.4, 156.0, 137.2, 136.2, 136.1, 135.7, 135.2, 132.9, 132.8, 132.3, 132.1, 131.4, 128.3, 128.1, 127.6, 127.57, 127.5, 127.0, 126.9, 126.3, 126.2, 126.0, 125.96, 125.9, 125.7, 125.3, 125.2, 124.3, 123.2, 122.7, 121.0, 120.9, 118.4, 118.3, 118.1, 118.0, 116.2, 111.4, 111.37, 111.3, 110.7, 86.26, 86.2, 50.4, 50.3, 50.0, 47.6, 47.1, 46.5, 42.4, 28.26, 28.2, 24.4, 23.0, 18.9, 17.5, 12.26, 12.2; **IR (ATR):** vmax 3325, 2926, 2343, 2115, 1922, 1618, 1544, 1454, 1367, 1242, 1089, 992, 901, 813, 741; **HRMS (ESI):** *m/z* calcd for C_40_H_48_N_6_O_4_S [M]^+^: 709.3531; found: 709.3523.

***(S)-N*-(2-(1*H*-indol-3-yl)ethyl)-2-amino-5-guanidino-*N*-(naphthalen-2-ylmethyl) pentanamide (TFA salt) (23)**. The title compound **23** was synthesized from compound **22** (0.09 g, 0.127 mmol) according to the protocol **F**. The product **23** was obtained as a white gum (0.046 g, 80%); **^1^H NMR** (600 MHz, DMSO) δ 10.92–10.81 (s, NH-indole, 1H), 8.33–8.29 (dd, α-CH**^+^**NH_3_ CF_3_COO^−^, 3H), 7.97–7.84 (m, ArH-naphthyl and NH-guanidine, 4H), 7.80–7.77 (m, ArH-naphthyl and NH-guanidine, 2H), 7.59–7.48 (m, ArH-naphthyl, 2H), 7.45 (s, NH-guanidine, 1H), 7.41–7.39 (m, ArH-indole, 1H), 7.36–7.31 (m, ArH-indole, 1H), 7.20–7.08 (dd, ArH-indole, 1H), 7.07–7.03 (m, ArH-indole, 1H), 6.95–6.88 (t, ArH-indole, 1H), 5.00–4.97 (s, Ar-CH^1^H^2^*-*N-2CH_2_-(indole)-Arg, 1H), 4.70–4.59 (s, Ar-CH^1^H^2^*-*N-2CH_2_-(indole)-Arg, 1H), 4.55–4.39 (q, α-CH of Arg, 1H), 3.82–3.22 (m, Ar-CH_2_-N-CH_2_-CH_2_-indole-Arg, 2H), 3.14–2.78 (m, δ-CH_2_- of Arg and Ar-CH_2_-N-CH_2_-CH_2_-indole-Arg, 4H), 1.79–1.49 (m, β-CH_2_,γ-CH_2_ of Arg, 4H); **^13^C NMR** (151 MHz, DMSO) δ 168.9, 168.6, 158.7, 158.5, 158.2, 158.0, 156.8, 156.7, 136.2, 136.1, 134.7, 134.0, 132.88, 132.8, 132.4, 132.3, 128.4, 128.3, 127.65, 127.6, 127.59, 127.5, 126.9, 126.7, 126.55, 126.5, 126.4, 126.2, 126.06, 126.0, 125.8, 125.5, 123.3, 122.8, 121.1, 121.0, 119.7, 118.4, 118.2, 118.1, 118.0, 117.7, 115.7, 113.7, 111.5, 111.4, 110.8, 110.3, 50.4, 49.6, 49.5, 48.0, 47.3, 46.1, 28.2, 28.0, 24.2, 24.1, 23.7, 22.7; **IR (ATR):** vmax 3353, 3183, 2933, 2343, 2093, 1650, 1430, 1366, 1176, 1128, 798, 744; **HRMS (ESI):** *m/z* calcd for C_27_H_32_N_6_O [M]^+^: 457.2711; found: 457.2709.

### 3.4. Minimum Inhibitory Concentration (MIC)

The antimicrobial activity of the compounds was evaluated by a broth microdilution assay using the procedure that was described by Clinical and Laboratory Standards Institute (CLSI) [62]. Briefly, bacteria were grown to the mid-log phase in Muller Hinton broth (MHB) with shaking at 120 rpm and incubated at 37 °C for 12–16 h. Following incubation, the bacteria were washed three times in PBS pH 7.4 at 3500 g for 10 min. After washing, the bacteria were diluted with fresh MHB. The turbidity of the bacterial suspensions was adjusted so that OD_660_ nm was 0.1, which gave 1 × 10^8^ CFU mL^−1^, and then further diluted to achieve 5 × 10^5^ CFU mL^−1^ as a final bacterial concentration. Each compound was diluted (250–3.9 µM) through two-fold dilution. The wells in the microtiter plates were loaded with 100 µL of inoculum containing 5 × 10^5^ CFU mL^−1^ bacteria. The wells without any compound and containing only bacteria were used as negative controls (i.e., no inhibition of growth). The wells with media only were set as blank. The microtiter plate was wrapped with paraffin to prevent evaporation and incubated with shaking at 120 rpm at 37 °C for 18–24 h. After incubation, a spectrophotometric reading was taken. The well at the lowest concentration without any bacterial growth and showing zero spectrophotometric reading was regarded as the MIC of the compounds. The MIC data of all the compounds were compared with that of ciprofloxacin (brand names Ciproxin, Ciloxan, and Cetraxal), which is a fluoroquinolone antibiotic. Each experiment was performed in triplicate and was repeated in three independent experiments.

### 3.5. Cytoplasmic Membrane Permeability Assay

The method was adopted from Wu et al. [63] with slight modification. The bacterial cytoplasmic membrane permeability was determined using membrane potential sensitive dye diSC3–5 (3,3′-dipropylthiadicarbocyanine iodide) which penetrates inside the bacterial cells depending on the membrane potential gradient of the cytoplasmic membrane. Bacteria were grown in MHB to the mid-log phase by incubating with shaking at 37 °C for 18–24 h. Following incubation, the bacteria were washed with 5 mM HEPES containing 20 mM glucose pH 7.2 and resuspended in the same buffer to an OD_600_ 0.05–0.06 which gave 1 × 10^7^ CFU ml^−1^. The dye diSC3–5 was added at 4 µM to the bacterial suspension. The suspensions were incubated at room temperature for 1 h in the dark for maximum dye uptake by the bacterial cells. Then, 100 mM KCl was added to balance the K^+^ outside and inside the bacterial cell to prevent further uptake or outflow of the dye. A total of 100 µL of bacterial suspension was added in a 96-well microtiter plate and with an equal volume of antimicrobial compounds. DMSO (20%) was set as a positive control while dye and only bacterial cells were set as negative control. Fluorescence was measured with a luminescence spectrophotometer at 3 min intervals at an excitation wavelength of 621 m and an emission wavelength of 670 nm.

### 3.6. Viable Cell Count Assay

The number of viable cells was confirmed by serially diluting aliquots of bacteria in D/E neutralizing broth (Remel, Lenexa, KS, USA) and plating these onto Tryptic Soy Agar (Oxoid, Basingstoke, UK) containing phosphatidylcholine (0.7 g L^−1^) and Tween 80 (5 mL L^−1^). The plates were incubated at 37 °C overnight and numbers of live bacteria were enumerated and expressed as CFU mL^−1^. The experiment was performed in triplicate.

### 3.7. Lysis of Horse Red Blood Cells

The haemolytic activities of the compounds that showed MIC ≤ 26 μg mL^−1^ (15 compounds) were determined using horse red blood cells (HRBCs; Sigma) as described previously [60]. The HRBCs were washed three times with PBS at 470× *g* for 5 min. The compounds (100 µM, 50 µM, and 25 µM, in PBS) were added to the washed HRBCs and incubated at 37 °C for 4 h. After incubation, the cells were pelleted at 1057× *g* for 5 min, and the supernatant was removed to assess the release of haemoglobin by measuring OD_540nm_. HRBCs in PBS and HRBCs in distilled water were used as negative (diluent) and positive controls to achieve 0% and 100% lysis, respectively. The relative OD of HRBCs that were treated with the 15 compounds were compared to those that were treated with distilled water and were used to determine the relative percentage of haemolysis. There were two separate experiments that were carried out in triplicate.

% haemolysis = (absorbance of test compound) − (absorbance of diluent)/(absorbance of positive control) − (absorbance of diluent) × 100

## 4. Conclusions

In conclusion, new short peptoids based on a tryptamine structural scaffold have been developed. The systematic tuning of hydrophobicity and cationic charge of the peptoids resulted in moderate to excellent antibacterial activities. Compounds **20b** and **22** showed excellent antibacterial activity against *S. aureus* (3.2 µg/mL and 2.1 µg/mL) without cytotoxicity against horse red blood cells. Based on the results of the cytoplasmic membrane permeability assay, the compounds may exhibit membrane damage mechanisms that are similar to most AMPs. These peptoids showed very good antibacterial activity in microbial keratitis bacterial strains which are resistant to ciprofloxacin. These short peptoids are worthy of further development in order to understand their mechanism of action on Gram-positive and Gram-negative bacterial strains.

## Figures and Tables

**Table 1:** Antibacterial activity (MIC) and hemolytic activity of the compounds.

Series No.	ID	R	n	MIC (μg mL^−1^)				HC_50_ (μg mL^−1^)
				*S. aureus* 38	*E. coli* K12	*S. aureus* 111	*S. aureus* 112	
**(I)**	**12a**	Indole	1	21.8	44.65	NT	NT	>50
	**12b**	Indole	2	22.7	22.7	NT	NT	19
	**12c**	Indole	3	12	**12**	NT	NT	10
	**12d**	Phenyl	1	39.7	>79.5	NT	NT	NT
	**12e**	Phenyl	2	>41.5	>83	NT	NT	NT
	**12f**	Phenyl	3	86.55	>86.5	NT	NT	NT
**(II)**	**13a**	Indole	1	**6.2**	24.4	**2.4**	**4.7**	>50
	**13b**	Indole	2	**6.4**	25.3	**2.5**	**4.9**	21
	**13c**	Indole	3	**6.6**	**13.3**	**2.6**	**5.1**	21
	**13d**	Phenyl	1	11.2	45	NT	NT	>50
	**13e**	Phenyl	2	11.6	46.7	NT	NT	>50
	**13f**	Phenyl	3	**6.1**	48.5	**4.6**	9.7	>50
**(III)**	**15a**	Indole	2	50	>100	NT	NT	NT
	**15b**	Indole	3	51.6	>103.3	NT	NT	NT
	**15c**	Phenyl	2	90	90	NT	NT	NT
	**15d**	Phenyl	3	93.5	>93.5	NT	NT	NT
**(IV)**	**16a**	Indole	2	51.7	103.5	NT	NT	NT
	**16b**	Indole	3	53.5	107	NT	NT	NT
	**16c**	Phenyl	2	93.8	>93.8	NT	NT	NT
	**16d**	Phenyl	3	>97.3	>97.3	NT	NT	NT
**(V)**	**19a**	Indole	2	51.7	51.7	NT	NT	>50
	**19b**	Indole	3	26.2	53.5	NT	NT	>50
	**20a**	Indole	2	15.5	62.3	NT	NT	>50
	**20b**	Indole	3	** 3.9 **	64	**3.2**	**3.2**	>50
	**22**	Indole	2	** 5.5 **	>177	**2.1**	**2.1**	>50
	**23**	Indole	2	**7.1**	114	11.4	11.4	>50
**Ciprofloxacin** *				0.1	0.02	**128**	**256**	NT

*, data from Afzal et al. [58], NT (not tested), MICs = most active peptoids against *E. coli* K, MICs = Most active peptoids against *S. aureus* strains compared to ciprofloxacin, **MICs** = compounds with a good activity against *S. aureus* strains. HC_50_ = concentration causing 50% lysis of horse red blood cells.

**Table 2:** MIC values of compounds **20b** and **22** against 12 *S. aureus* strains that were isolated from keratitis.

*S. aureus* Strain	Antimicrobial Compound		
	20b (μg mL^−1^)	22 (μg mL^−1^)	Ciprofloxacin (≤1, 2, ≥4) * (μg mL^−1^)
106	3.2	2.1	128
107	3.2	2.1	64
108	3.2	2.1	1
109	3.2	2.1	128
110	3.2	2.1	128
111	3.2	2.1	128
112	3.2	2.1	256
113	3.2	2.1	128
114	6.4	2.1	8
M5.01	6.4	2.1	64
M43.01	3.2	2.1	128
M71.01	3.2	2.1	4

* break points for ciprofloxacin form CLSI; data from Afzal et al. [58].

## Data Availability

Data is contained within the article and Supplementary Materials.

## Supplementary Materials

The following Supplementary Materials can be downloaded at: https://www.mdpi.com/article/10.3390/antibiotics11081074/s1, Supplementary Files: Supporting Data.
