# Supplementary material for: Short Tryptamine-Based Peptoids as Potential Therapeutics for Microbial Keratitis: Structure-Function Correlation Studies
Source: Antibiotics (Basel). 2022 Aug 8;11(8):1074. doi: 10.3390/antibiotics11081074 (PMC9404767; doi:10.3390/antibiotics11081074)

The Supplementary Data for

Short tryptamine based peptoids as potential therapeutics for microbial keratitis: Structure-function correlation studies

Ghayah Bahatheg [1,2], Rajesh Kuppusamy [1,3,*], Muhammad Yasir [3], David StC Black [1], Mark Willcox [3] and Naresh Kumar [1,*]

[1] School of Chemistry, The University of New South Wales, , Australia;

2 Department of Chemistry, Faculty of Science, University of Jeddah, Jeddah, 21589, Saudi Arabia

[3] School of Optometry and Vision Science, The University of New South Wales, UNSW Sydney, NSW 2052, Australia

Table of Contents

1. Design and synthesis of the main scaffolds and their ^{1}HNMR and ^{13}C NMR data……. ….S2
2. Attempted reaction *N,N*-Dimethylglycine hydrochloride with compound 8 and 9…..…..S4
3. TFA salts peaks……………………………………………………………………….... S6
4. VT ^{1}HNMR spectra of compounds 12a,13a,14a and 15a……………………...…..................… S7
5. The calculations of the free energy barriers between the two unequal populated isomers of CH_2-naphthyl (12a) ………...…………………………………………………………….......S11
6. ^{1}H and ^{13}CNMR spectra of the main scaffolds naphthyl, phenyl, and indole secondary amines………………………………………………………………………...……S12
7. ^{1}H, and ^{13}CNMR of Boc-naphthyl-indole and naphthyl-phenyl based peptoids……….....S18
8. ^{1}H, and ^{13}CNMR of mono-amine naphthyl-indole and naphthyl-phenyl based peptoids. S38
9. ^{1}H, and ^{13}CNMR of diamino-naphthyl- indole based peptoids……………………..........S52
10. ^{1}H, and ^{13}CNMR of mono-guanidine naphthyl-indole and naphthyl-phenyl based peptoids…………………………………………………………………………….... S58
11. ^{1}H, and ^{13}CNMR of di-guanidine-naphthyl- indole based peptoids……………………….. S70
12. ^{1}H, and ^{13}CNMR of dimethyl- naphthyl-indole and naphthyl-phenyl based peptoids…..... S76
13. ^{1}H, and ^{13}CNMR of tertiary ammonium hydrochloride salts peptoids…………..............S84
14. ^{1}H, and ^{13}CNMR of quaternary ammonium iodide salts peptoids…………………...…...…S92
15. Expanded ^{1}HNMR spectra for some regions not clear in the full ^{1}HNMR spectra………. S100
16. HRMS data……………………………………………………………………………...S112

1- Design and synthesis of the main scaffolds:

The indole ring in tryptophan is known to prefer the interfacial regions of the cell lipid membrane, hence the indole motif is incorporated into the peptides to increase their cell permeability [16]. Thus, the core basic scaffolds were synthesized contain both naphthyl and indole rings. The naphthyl ring increases the hydrophobicity of the compounds, while the indole ring promotes membrane permeability. Furthermore, cationic amino acids were used to increase the electrostatic interaction between antibacterial peptoids and cell membranes. The initial core scaffolds were synthesized via a reductive amination reaction between aldehyde **6** bearing a naphthyl group and amine **7** bearing an indole or phenyl group, to produce the aromatic secondary amine scaffolds **8** and **9**, using an established procedure [48]. Phenyl rings were also investigated in place of naphthyl to investigate the importance of these groups on biological activity, giving scaffolds **12a**, **12d** and **26 (Scheme S1)**. α-Gly-peptoids **12a**,**12b** and **26** were tested against Gram-positive and Gram-negative bacteria. Compound **12a** which contains indole and naphthyl has shown the best activity against sa.38 and E. coli k12 among the three initial peptoids (*see Table S1 for MIC values*). These results encouraged us to focus on scaffold **12a** to create most active peptoids.

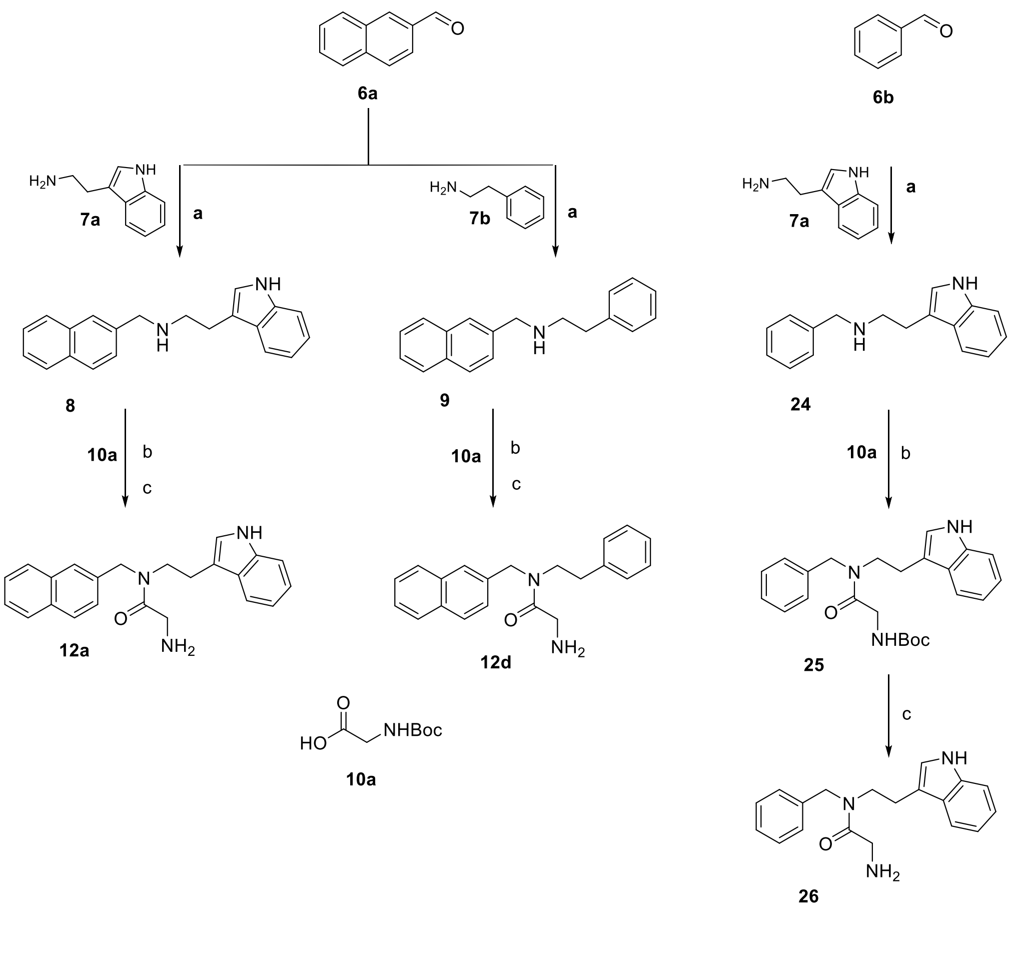

Scheme S1. General procedure for the synthesis of main scaffolds via reductive amination. a) 7a or 7b (1.0 equiv), TMOF, rt, 1.5h, AcOH, $NaCNBH_3$ (0.3 equiv) 20 mins rt. **b)** 10a (1.0-2.0 equiv), coupling reagent (HATU 1.1equiv), or (HBTU 2.0 equiv), DIPEA (3.0 equiv), DMF, rt, 2-3 hrs. **c)** DCM (1-3 mL), TFA (1-3 mL). rt, 1-3 hrs.

Table S1. The initial MIC results of the first compounds.

ID	MIC of SA38 (μM)	MIC of E. coli k12 (μM)
12a	21.8	125
12d	125	> 125
26	> 125	> 250

General procedure A for the synthesis of compounds (8-9-24) via reductive amination.

Tryptamine or phenylethylamine (1 equiv) and 2-naphthladhyde or benzaldehyde (1 equiv) in trimethyl orthoformate (40 mL) was stirred for 1h at rt under an argon atmosphere. After 1h AcOH (1.6 ml) and $NaCNBH_3$ (0.3 equiv) was added to the reaction mixture, stirring was continued for 20 mins. After completion of the reaction NaOH 1N was added to the mixture. The reaction mixture was extracted with ethyl acetate (350ml) and then it was dried over $NaSO_4$. Then it was filtered and evaporated solvent in vacuo. The crude products were purified by flash chromatography using $CHCl_3$/MeOH 10 % or EtOAc/Hexane. The pure compound was dried under vacuum to give a solid product.

^{1}H NMR and ^{13}C NMR spectra of 2-(*1H*-indol-3-yl)-*N*-(naphthalen-2-ylmethyl)ethan-1-amine (8).The compound (8) was prepared from tryptamine (12 mmol, 2g) and 2-naphthaldhyde (12 mmol,1.87 g) according to the general procedure **A**. The product 8 was obtained as yellowish solid product(1.838 g, 50%); mp 97.7-99.8 °C; **^{1}H NMR** (400 MHz, DMSO) δ 10.76 (s, 1H), 7.90 – 7.76 (m, 3H), 7.80 – 7.77 (m, 1H), 7.55 – 7.41 (m, 4H), 7.32 (dt, *J* = 8.1, 1.0 Hz, 1H), 7.13 (d, *J* = 2.3 Hz, 1H), 7.04 (ddd, *J* = 8.1, 6.9, 1.2 Hz, 1H), 6.93 (ddd, *J* = 7.9, 7.0, 1.0 Hz, 1H), 3.91 (s, 2H), 2.93 – 2.79 (m, 4H), 2.26 (s, 1H); **^{13}C NMR** (101 MHz, DMSO) δ 138.7, 136.2, 132.9, 132.0, 127.5, 127.4, 127.2, 126.7, 125.9, 125.8, 125.3, 122.5, 120.7, 118.3, 118.0, 112.6, 111.3, 52.9, 49.5, 25.5. ; **IR (ATR): νmax** 3414, 3295, 3049, 2897, 2753, 1425, 1338, 1226, 1096, 997, 813, 737; **HRMS (ESI):** m/z calcd for $C_{21}H_{20}N_2$ $[M]^+$: 301.1699; found: 301.1697

^{1}H NMR and ^{13}C NMR spectra of *N*-(naphthalen-2-ylmethyl)-2-phenylethan-1-amine (9). The compound **(9)** was obtained from phenylethylamine (28 mmol, 3.54mL) of and 2-naphthaldhyde (20 mmol,4g) according to the general procedure **A**. The product **9** was obtained as a yellowish solid. (2.37 g, 35%); mp 41.3- 42.7 °C; **^{1}H NMR** (400 MHz, DMSO) δ 7.91 – 7.80 (m, 3H), 7.78 (d, *J* = 1.6 Hz, 1H), 7.53 – 7.42 (m, 3H), 7.27 7.18 (m, 5H), 3.89 (d, *J* = 0.9 Hz, 2H), 2.77 (s, 4H); **^{13}C NMR** (101 MHz, DMSO) δ 140.5, 138.5, 132.9, 132.0, 128.5, 128.4, 128.3, 128.2, 127.5, 127.4, 126.7, 125.93, 125.8, 125.7, 125.3, 52.9, 50.4, 35.8; **IR (ATR): νmax** 3022, 2823, 2401, 2115, 1939, 1598, 1442, 1327, 1105, 1013, 897, 821, 739; **HRMS (ESI):** m/z calcd for $C_{19}H_{19}N$ $[M]^+$: 262.1590; found: 262.1589.

^{1}H NMR and ^{13}C NMR spectra of *N*-benzyl-2-(1H-indol-3-yl) ethan-1-amine (24). The compound (**24**) was obtained from Tryptamine (12 mmol, 2g) of and benzaldehyde (12 mmol,1.3mL) according to the general procedure **A**. The product **24** was obtained as a light brown gum, (0.27g, 84%); **^{1}H NMR** (600 MHz, DMSO) δ 10.77 (s, 1H), 7.49 (d, *J* = 7.9 Hz, 1H), 7.36 – 7.28 (m, 5H), 7.27 – 7.18 (m, 1H), 7.13 (d, *J* = 2.3 Hz, 1H), 7.09 – 7.02 (m, 1H), 6.96 (td, *J* = 7.4, 6.9, 1.0 Hz, 1H), 4.11 (s, 1H), 3.77 (s, 2H), 2.91 – 2.78 (m, 4H); **^{13}C NMR** (151 MHz, DMSO) δ 140.4, 136.2, 128.12, 128.1, 128.0, 127.2, 126.6, 122.5, 120.8, 118.2, 118.1, 112.4, 111.3, 52.7, 49.4, 25.2; **IR (ATR): νmax** 3409, 3054, 2915, 2837, 2102, 1600, 1452, 1338, 1227, 1092, 803; **HRMS (ESI):** m/z calcd for $C_{17}H_{18}N_2$ $[M]^+$: 251.1542; found: 251.1541.

^{1}H NMR and ^{13}C NMR spectra of *Tert*-butyl(2-((2-(*1H*-indol-3-yl)ethyl)(benzyl)amino)-2-oxoethyl)carbamate (25). The title compound **25** was prepared via protocol **C** using compound **24** (0.22 g, 0.1 mmol) and Boc-Gly-OH (0.175 g, 0.1 mmol) to give a brown solid (0.309 g, 76%); mp 57.6-58.9 °C; **^{1}H NMR** (600 MHz, DMSO) δ 10.83 (d, NH-indole, 1H), 7.49 (t, ArH, 1H), 7.41 – 7.23 (m, ArH 6H), 7.14 (dd, ArH, 1H), 7.10 – 7.03 (m, ArH 1H), 7.01 – 6.92 (m, ArH, 1H), 6.82 (q, ArH,1H), 4.57 (d, Ar-CH^{1}H^2-N-2CH$_2$-(indole)Boc-Gly, 2H), 3.85 (dd, α-CH^{1}H^2 of Boc-Gly, 2H), 3.51 – 3.44 (m, Ar-CH$_2$-N-CH$_2$-CH$_2$-indole, 2H), 2.98 – 2.82 (m, Ar-CH$_2$-N-CH$_2$-CH$_2$-indole, 2H), 1.40 (d, (CH$_3$)$_3$ of Boc- Gly, 9H).; **^{13}C NMR** (151 MHz, DMSO) δ 169.0, 168.8, 155.9, 155.8, 138.0, 137.4, 136.2, 136.1, 128.6, 128.3, 127.5, 127.2, 127.0, 126.99, 126.9, 126.7, 126.6, 123.1, 122.6, 121.0, 120.9, 118.3, 118.2, 118.0, 111.4, 111.37, 111.3, 110.6, 78.1, 77.9, 77.8, 60.2, 49.7, 48.0, 47.0, 46.9, 41.9, 41.3, 38.2, 28.2, 27.9, 23.9, 23.1; **IR (ATR): νmax** 3297, 3056, 2973, 2339, 2105, 1694, 1641, 1529, 1451,1364, 1159, 1051, 947, 864, 736; **HRMS (ESI):** m/z calcd for $C_{24}H_{29}N_3O_3$ $[M + Na]^+$: 430.2101; found: 430.2102.

^{1}HNMR and ^{13}CNMR spectra of *N*-(2-(*1H*-indol-3-yl) ethyl)-2-amino-*N*-benzylacetamide (26) (TFA salt). The title compound **26** was synthesized from compound **25** (0.189 g, 0.42 mmol) according to the protocol **E**. The product **26** was obtained as light brown solid (0.09 g, 60%) mp 149.1-150.8 °C; **^{1}H NMR** (600 MHz, DMSO) δ 11.17 – 10.68 (m, NH-indole, 1H), 7.91 (s, $^+$NH$_3$ CF$_3$COO$^-$, 3H), 7.52 (dd, ArH, 1H), 7.41 (t, ArH, 1H), 7.38 – 7.26 (m, ArH, 5H), 7.14 (dd, ArH, 1H), 7.11 – 7.04 (m, ArH, 1H), 6.98 (m, ArH, 1H), 4.62 (d, Ar-CH^{1}H^2-N-2CH$_2$-indole (Gly), 2H), 3.86 (d, α-CH^{1}H^2 of Gly, 2H), 3.71 – 3.42 (m, Ar-CH$_2$-N-CH^{1}H^2-CH$_2$- indole (Gly), 2H), 2.93 (m, Ar-CH$_2$-N-CH$_2$-CH^{1}H^2-indole, 2H);**^{13}C NMR** (151 MHz, DMSO) δ 166.3, 137.3, 136.6, 136.2, 136.1, 128.7, 128.4, 127.7, 127.6, 127.2, 127.0, 126.8, 123.3, 122.7, 121.1, 121.0, 118.4, 118.3, 118.14, 118.1, 111.5, 111.4, 110.9, 110.4, 49.8, 47.8, 46.9, 46.9, 23.4, 23.0; **IR (ATR):** vmax 3313, 3057, 2961, 2321, 2106, 1778, 1638, 1576, 1492, 1359, 1188, 1119, 1016, 915, 799, 737; **HRMS (ESI):** m/z calcd for $C_{19}H_{21}N_3O$ $[M]^+$: 308.1757; found: 308.1757.

2- Attempted reaction *N,N*-Dimethylglycine hydrochloride with 2-(*1H*-indol-3-yl)-N-(naphthalen-2-ylmethyl)ethan-1-amine or *N*-(naphthalen-2-ylmethyl)-2-phenylethan-1-amine.

The reaction of *N,N*-Dimethylglycine hydrochloride with naphthyl-indole α-amin and naphthyl-phenyl α-amine as shown in Scheme S2 protocol **a** was used a synthetic route to produce dimethyl amino-propane peptoids. Unlike the reaction with 3-(Dimethyl- amino) propionic acid hydrochloride

or 4-(Dimethyl -amino)butyric acid hydro- chloride, the reaction with *N,N*-Dimethylglycine hydrochloride was unsuccessful.

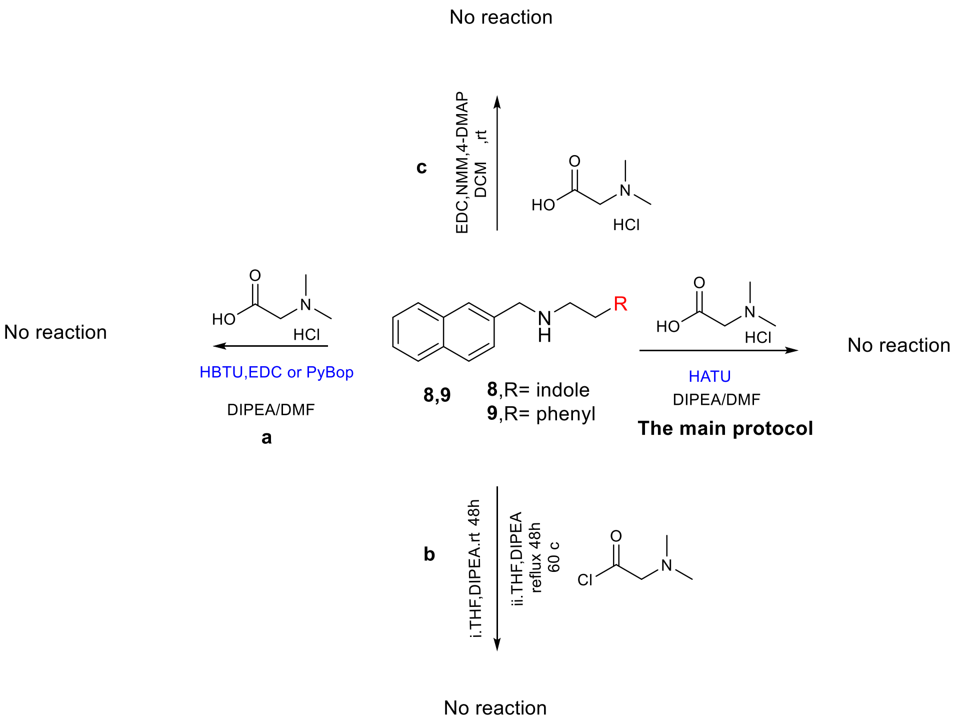

Scheme S2. Attempted reactions to prepare (dimethylamino)propane peptoids.

As a result of this negative reaction according to protocol a, several routes and conditions were investigated.

a- The utilization of varying coupling reagents such as HBTU, EDC, and ByBop Scheme S2

<u>The general procedure:</u>

The amine compound A,B (1 equiv), *N,N*-Dimethylglycine hydrochloride (1.1 equiv), and coupling reagent (1.1 equiv) (EDC 2.5 equiv) were dissolved in DMF (15mL) by stirring at rt. Then DIPEA (3.0 equiv) was added to the reaction portion-wise. The reaction was stirred at rt under an argon atmosphere for 48h. The reaction was under TLC monitoring; after 48h, there was no new spot on the TLC plate except the starting material's spot.

b- The conversion of *N,N*-dimethylglycine hydrochloride into *N,N*-dimethylglycinol chloride, In an attempt to activate the reaction, *N,N*-dimethylglycinol chloride was reacted with compound A or B as shown in in Scheme S2 rout **b.** There was no reaction under these conditions. For this reason, reaction b in Scheme S2 was subjected to reflux under 60 C° for 48 hrs, but no result was obtained.

c- The reaction between primary scaffold **8** or **9** and *N,N*-dimethylglycine hydrochloride as shown in Scheme S2 rout c was also not working.

3- TFA salts peaks in ^{13}CNMR

Amino and guanidinium peptoids NMR spectra show the TFA salt peaks ($^{+}NH_nCF_3COO^{-}$) For amino peptoids **12a-d**, **20a-b** and **22** ($^{+}N\underline{H_3}\,CF_3COO^{-}$) peaks was clear in ^{1}HNMR, they appeared as a single or multiple peaks after ArH-peaks and in ^{13}CNMR between 157 and 158 ppm. For guanidinium peptoids also we noticed TFA salt peaks at 157 and 158 in ^{13}C NMR.

To prove if the peaks in NMR belong to ($^{+}NH_nCF_3COO^{-}$) or not we used Lysine and dissolved it in TFA for 10 mins then dry it after that we subjected the product to ^{13}CNMR. ^{13}CNMR for Lysine TFA salt shows the same peak at 157-158 similar to the ^{13}CNMR spectra of amine and guanidine's peptoids in this work. Figure S1 and S2.

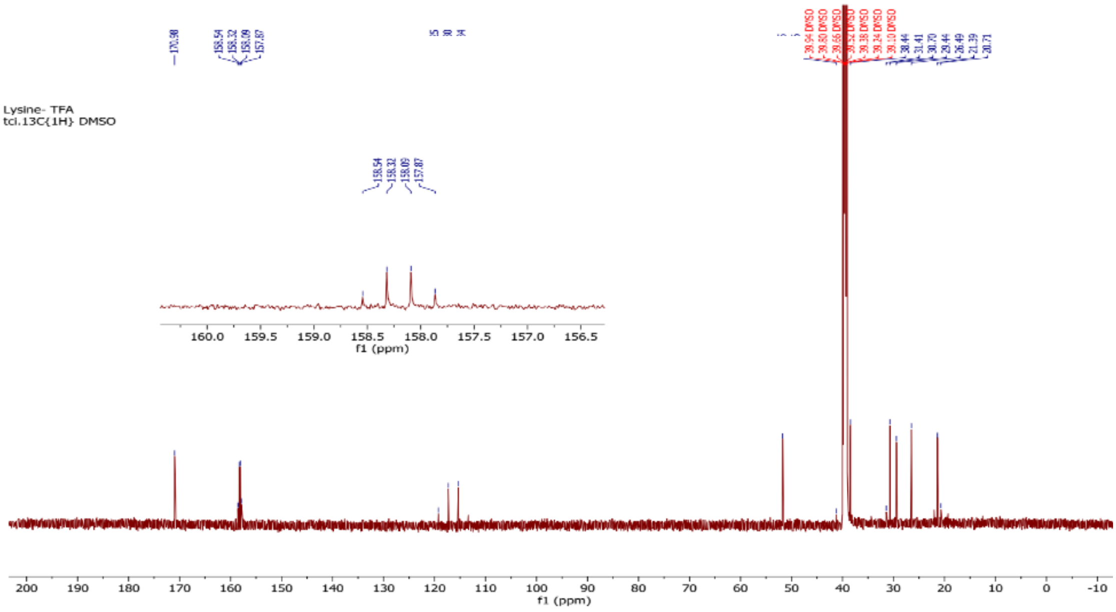

Figure S1. ^{13}C NMR spectra of Lysine TFA salt.

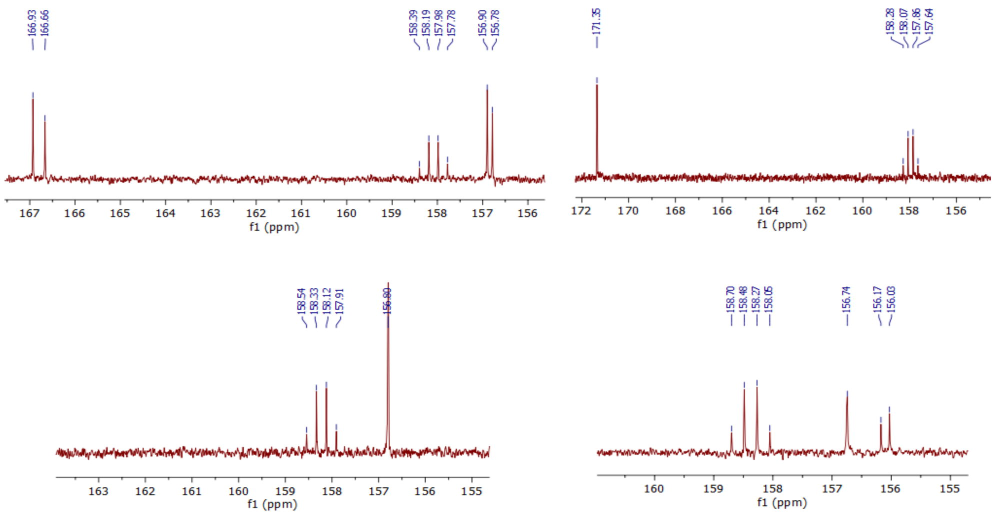

Figure S2. Expanded spectra for TFA salt region in ^{13}C NMR of some of amine and guanidine peptoids in this work.

4- VT ^{1}HNMR spectra of compounds 12a,13a,14a and 15a:

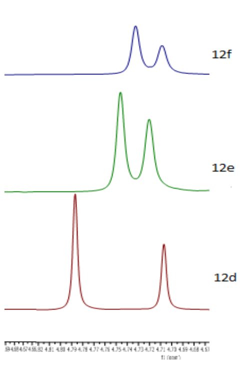

Figure S3. The chemical shift between CH̲$_2$-naphthyl doublet signals in ^{1}HNMR spectra in DMSO-d$_6$ at 289 k compounds 12d-12f

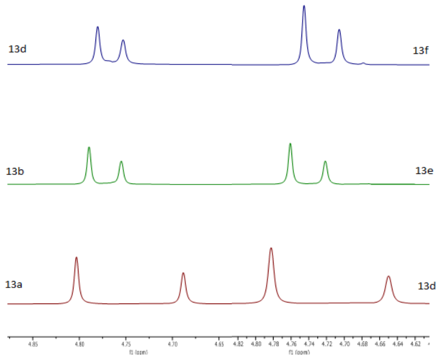

Figure S4. The chemical shift between CH̲$_2$-naphthyl doublet signals in ^{1}HNMR spectra in DMSO-d$_6$ at 289 K compounds 13a-13f

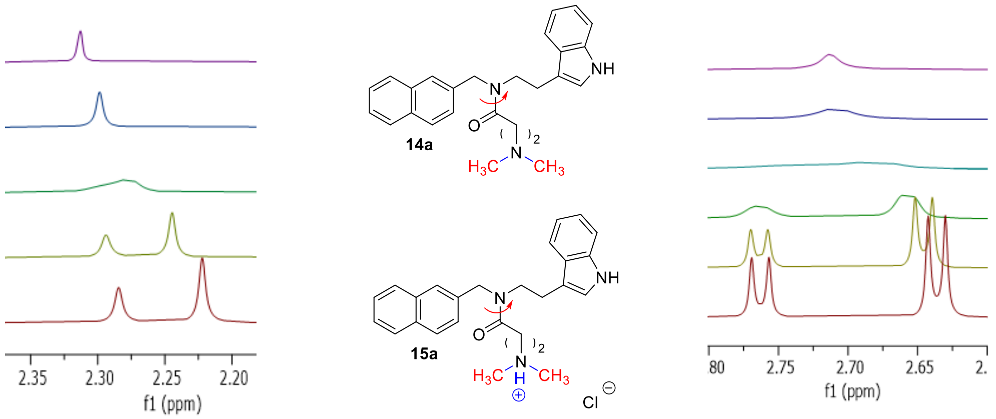

Figure S5. ^{1}HNMR spectra of 14a (left) and 15a (right) over 298-383 k in DMSO-d$_6$, (-N-dimethyl region)

The reaction between compound **14a** and HCl produced the tertiary ammonium hydrochloride salt compound **15a** and its VT ^{1}HNMR is shown in Figure S5. In DMSO-d_6 at 298k, the two CH_3 groups of the tertiary amide **15a** showed dd signals. In contrast, the same two CH_3 groups in compound **14a** before the reaction with HCl showed double signals. These outcomes can be explained based on the HCl proton attached to the tertiary amide nitrogen atom (compound **15a**) which affected the splitting of each CH_3 group in the two isomers to appear as two double peaks, as shown in (Figure S5).

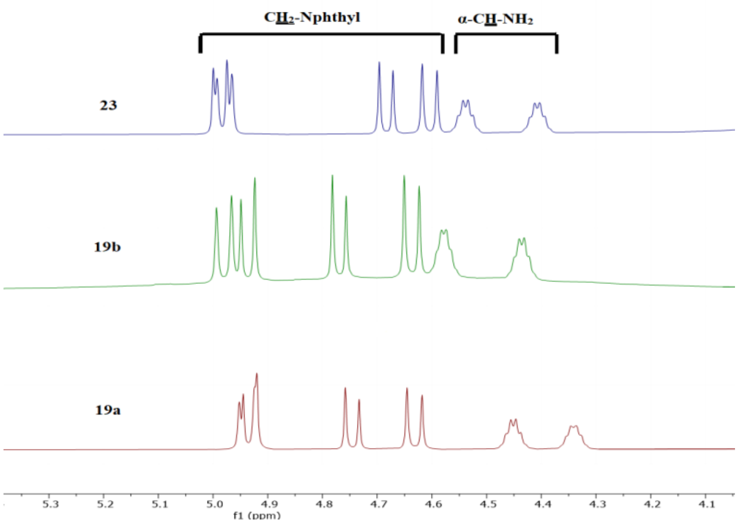

Figure S6. C<u>H</u>$_2$-Nphthyl and α-C<u>H</u>-NH$_2$ region in compounds 19a,19b and 23 in DMSO-d$_6$ at 289 K compounds.

Figure S6. shows the splitting of C<u>H</u>$_2$-Nph peaks of compounds **19a**,**19b**, and **23** which contain NH_2 group attached to α-carbon and how these peaks are affected by the isomerization and the presence of amino group on alpha position. It is clear that the first proton from the left appears as dd and the chemical shift between dd signals is not huge while the second proton spiltting in dd peaks with a huge chemical shift difference between the two double signals.

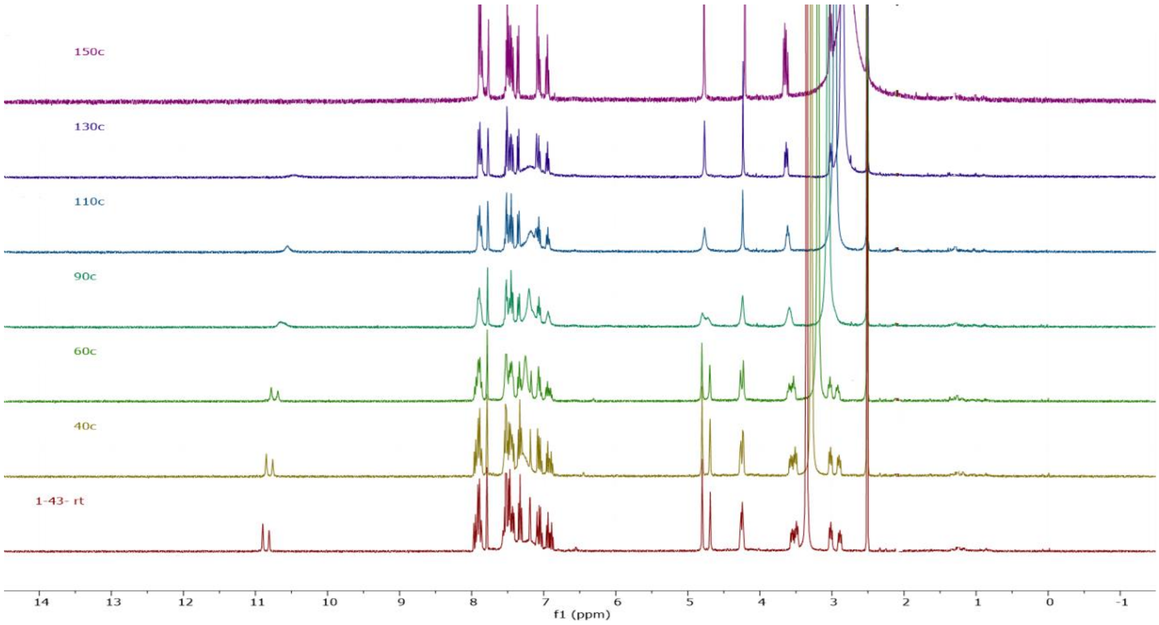

Figure S7. The ^{1}H NMR spectra of compounds 13a at variable temperatures in DMSO-d_6.

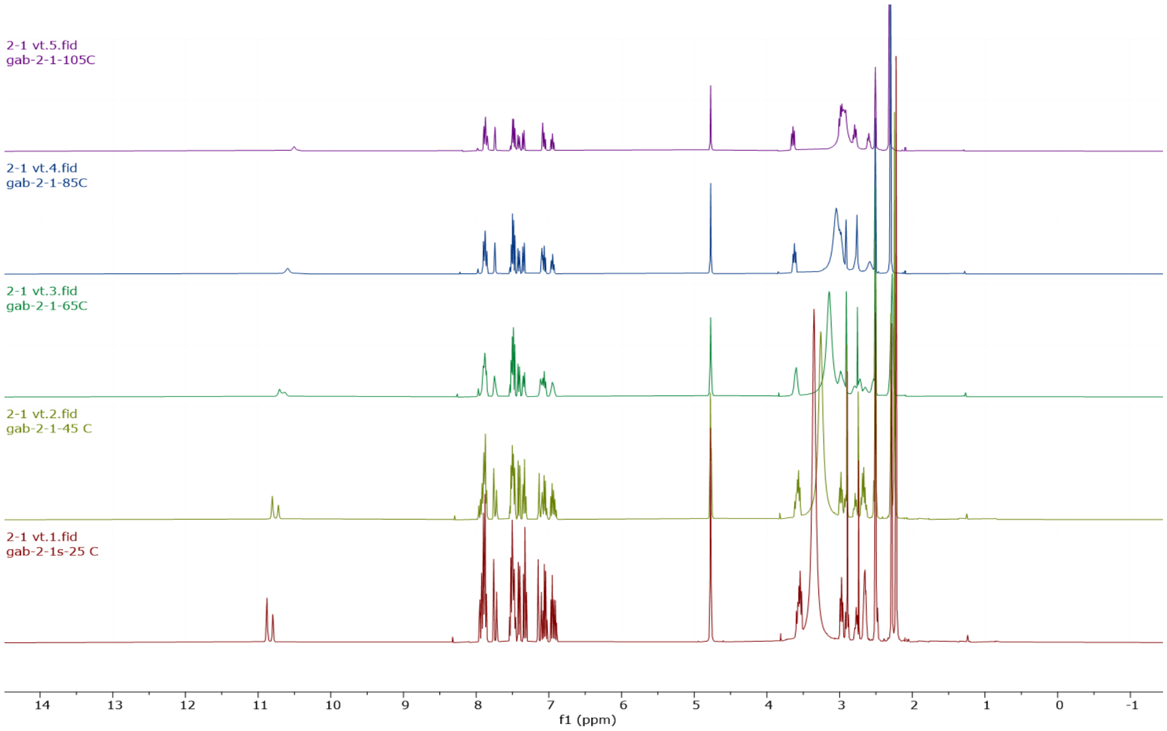

Figure S8. The ^{1}H NMR spectra of compounds 13a at variable temperatures in DMSO-d_6.

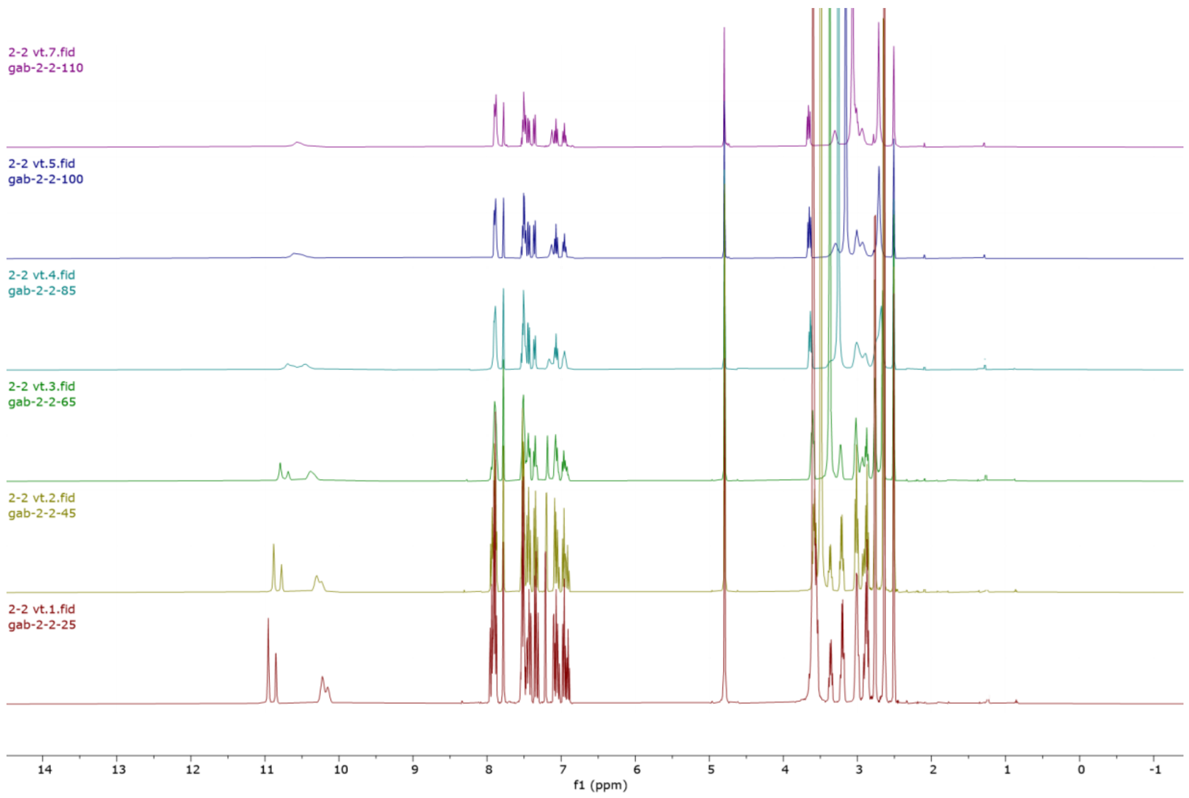

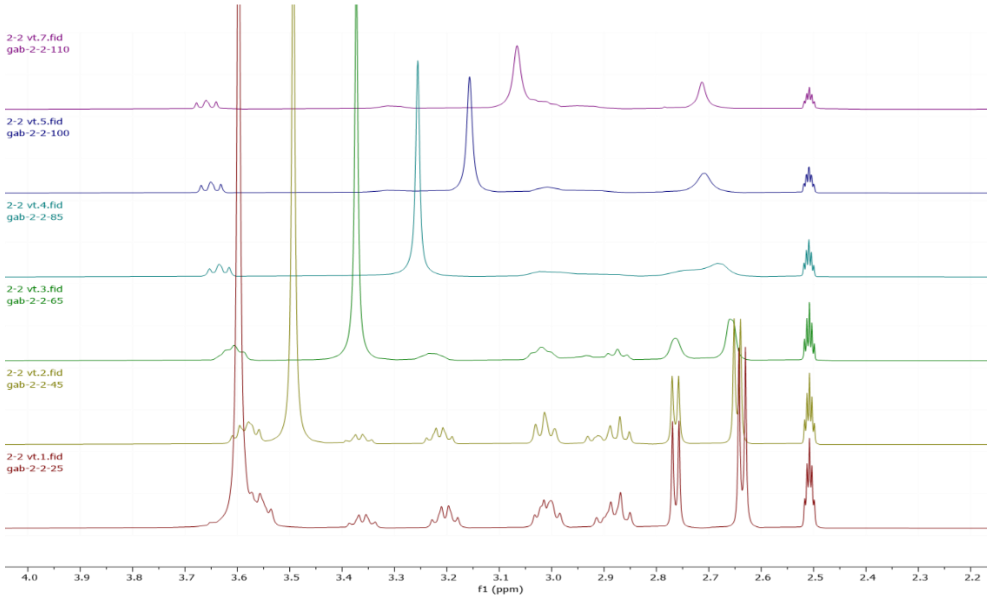

Figure S9. The ^{1}H NMR spectra of compounds 15a at variable temperatures in DMSO-d_6.

5- The calculations of the free energy barriers between the two unequal populated isomers of CH_2-naphthyl

The energy barriers between two unequal isomers in compound **12a** depending on the CH_2-naphthyl double signals can be obtained from Eyring's equations (1 and 2) as modified by Shanan-Atidi and Bar-Eli [56,57].

$\Delta G^{\ddagger}$= 4.57Tc {10.62 + log [X/2π (1 - ΔP)] + log (Tc/ Δν)} (1)

$\Delta G^{\ddagger}$= 4.57Tc {10.62 + log [X/2π (1 + ΔP)] + log (Tc/ Δν)} (2)

Tc refers to the coalescence temperature, and Δv is the frequency difference between isomers M and m. $\Delta v = V_M - V_m$ is obtained using equation (3).
$\Delta_m = V_m - V_{TMS} / V_{NMR} * 10^6{}_{ppm}$ (3)

P_M and P_m mean the ratio of the rotamers; M and m (PA > PB, PA + PB = 1). X is calculated using equation (4) depending on the ΔP value.

PA − PB = ΔP = [(X2 − 2)/3]3/2*1/X (4).

$\Delta P = (1.3 - 1)/2.3 = [(X^2 - 2)/3]^{3/2} * 1/X$, X=1.551

The ^{1}HNMR experiment at variable temperature found that the coalescence temperature, Tc, was 383 K. The frequency difference (Δv) of the major and minor isomers was obtained from ^{1}HNMR spectra at 298 K, and it was 32 Hz.

By the substitution of values of the variables in equation 1 and 2 we calculated the free energy of activation of the two isomers.

$\Delta G^{\ddagger}_A$ = 4.57 *383* {10.62 + log [1.551/2π (1 – 0.1304)] + log (383/ 32)} = 19157 cal/mol

($1 \text{ cal mol}^{-1} = 4.184 \text{ J mol}^{-1}$)

$\Delta G^{\ddagger}_A$ = 80.152 KJ/mol

$\Delta G^{\ddagger}_B$ = 4.57 Tc {10.62 + log [1.551/2π (1 + 0.1304)] + log (383/ 32)} = 19318 cal/mol

$\Delta G^{\ddagger}_B$ = 80.826 KJ/mol = ~ 81 KJ/mol

6- ^{1}H and ^{13}CNMR spectra of the main scaffolds naphthyl, phenyl and indole secondary amines

*1. (Compound **8**- ^{1}HNMR)*

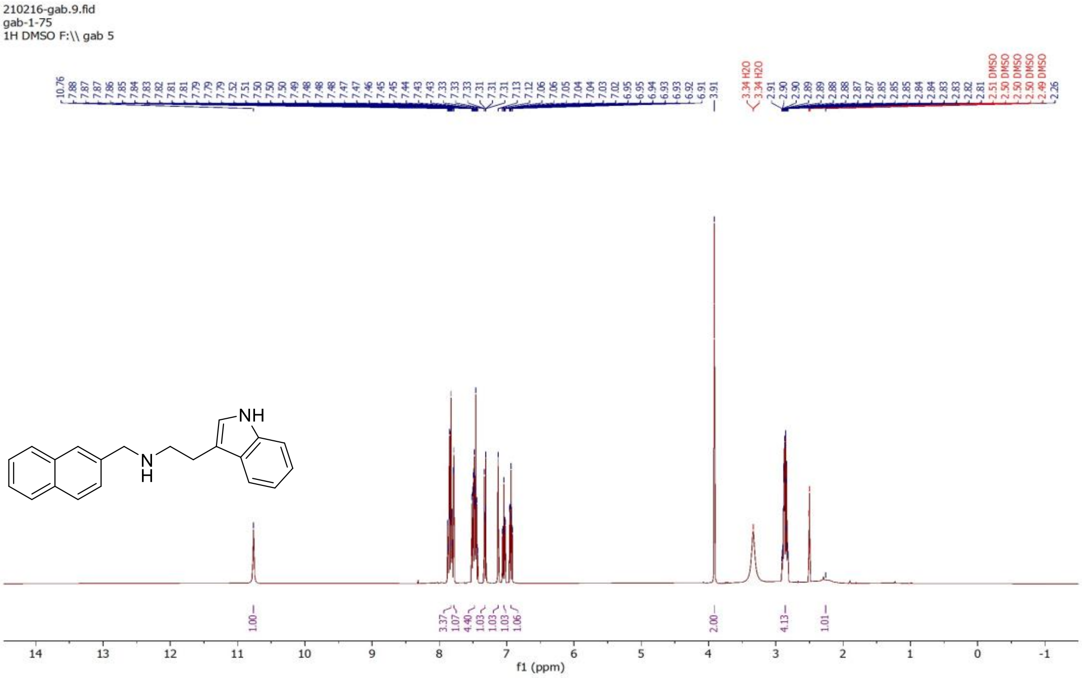

*2. (Compound **8**-^{13}CNMR)*

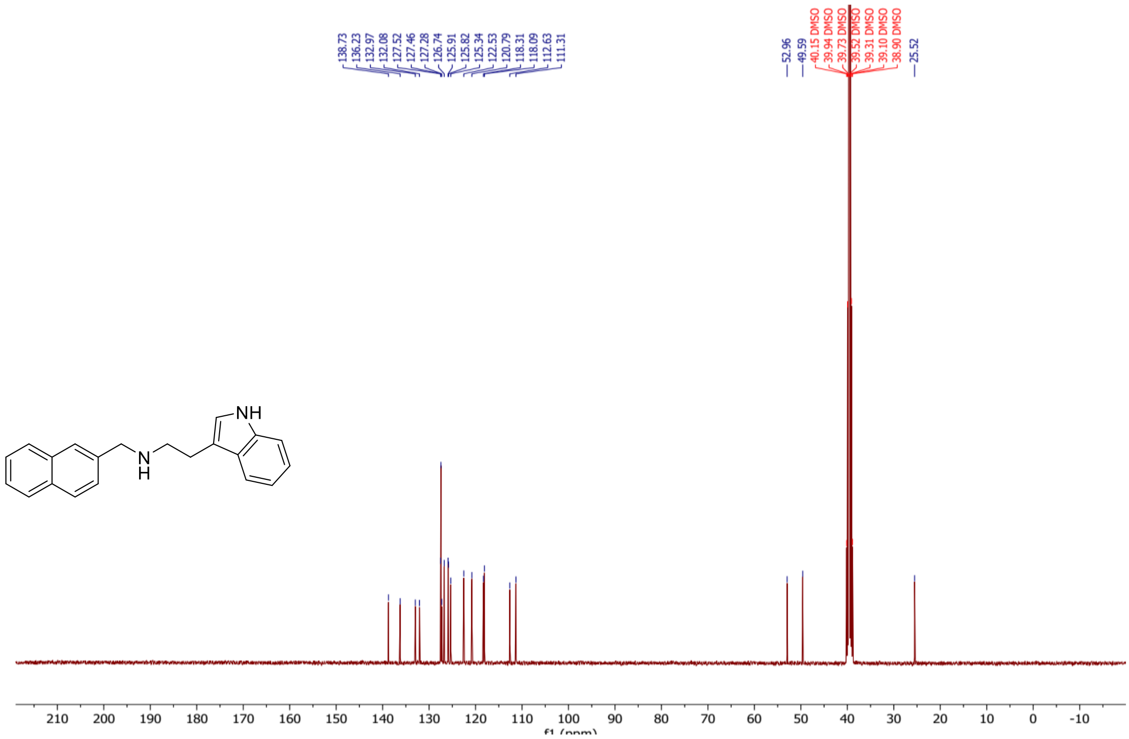

*3. (Compound **8** -^{13}CNMR-dept 135)*

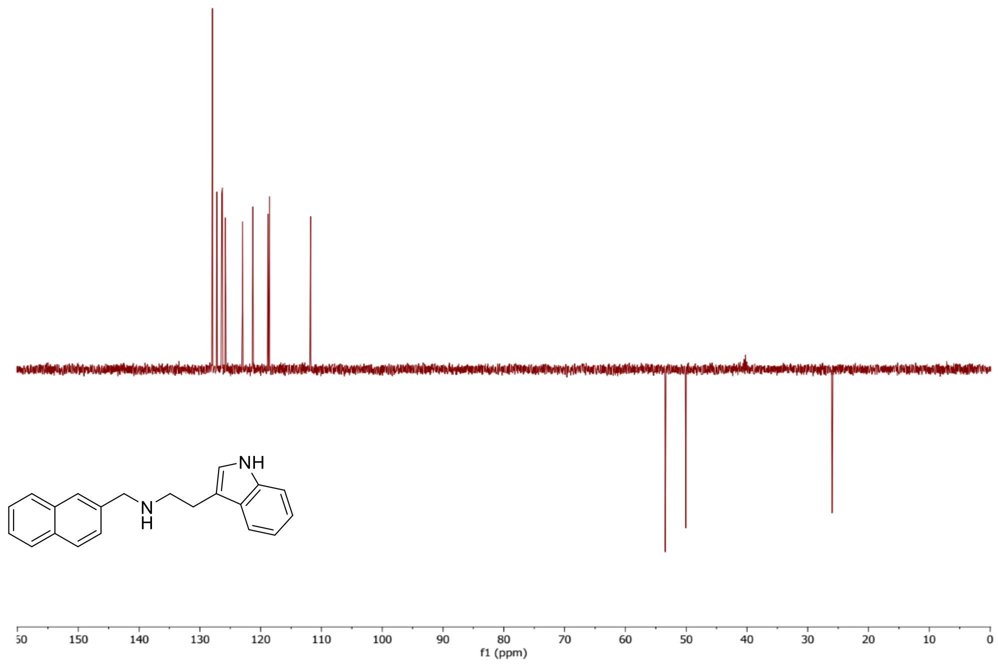

*4. (Compound **8**-HSQC)*

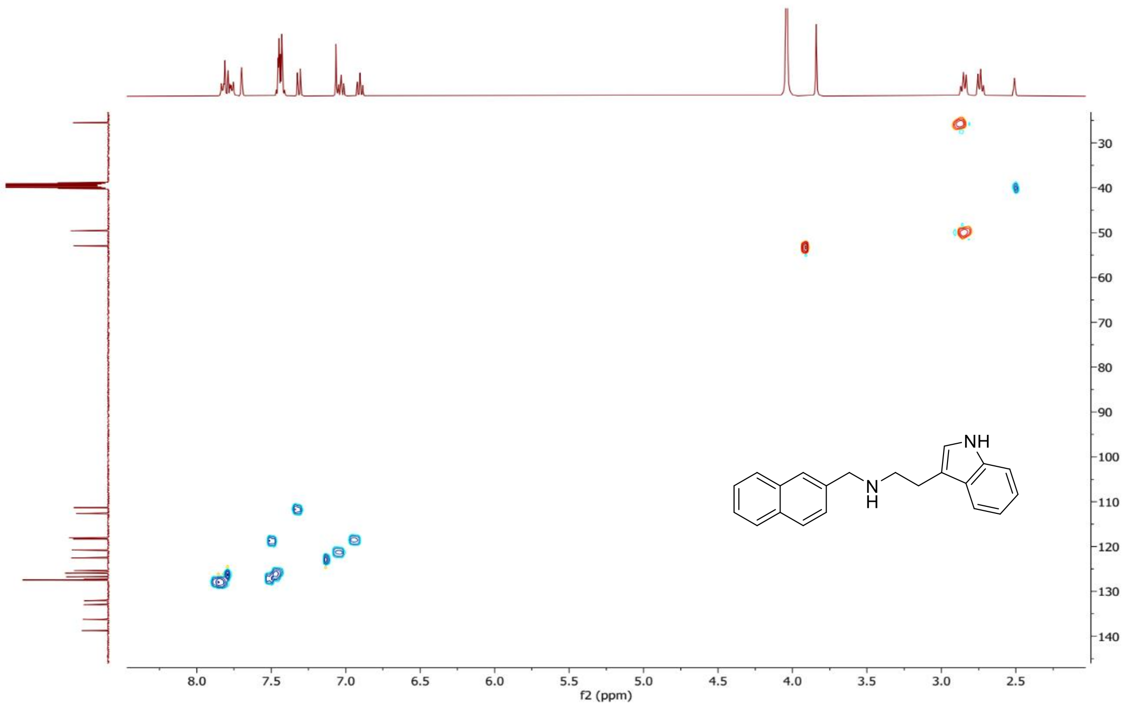

*5. (Compound **9**- 1HNMR)*

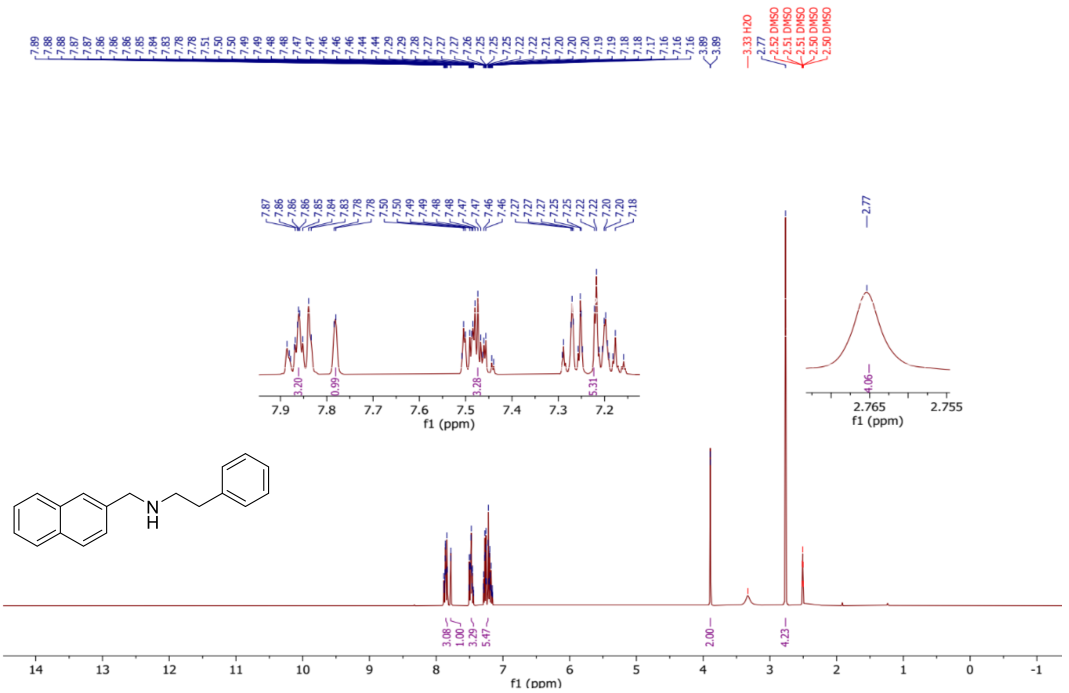

*6. (Compound **9** -13CNMR)*

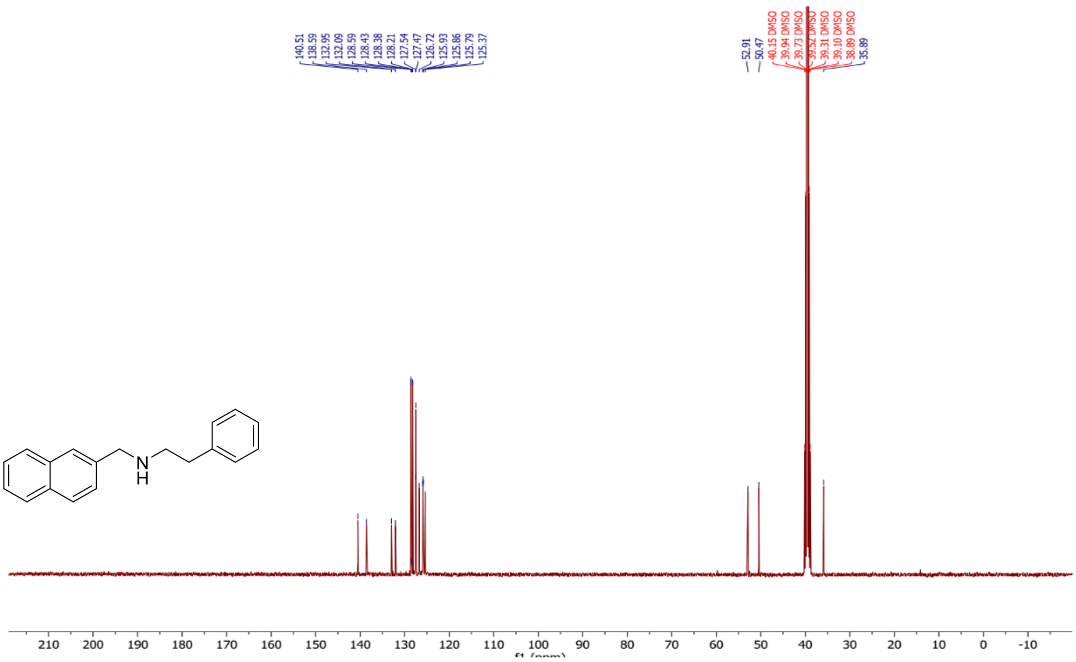

*7. Compound **9** – ^{13}CNMR-dept 135*

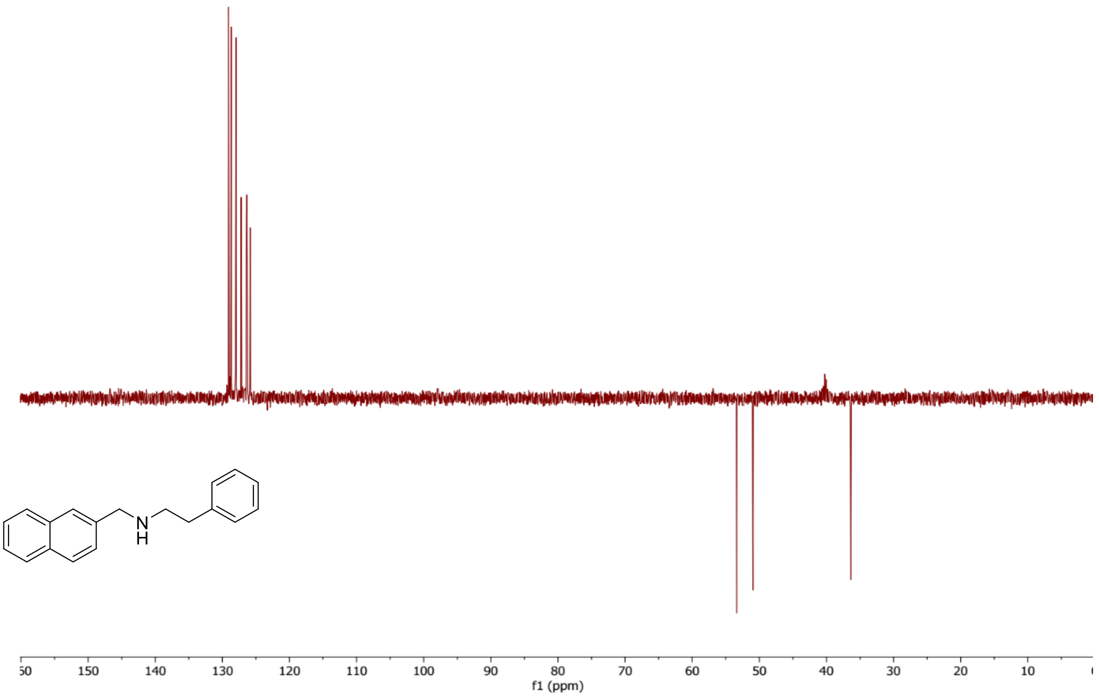

*8.Compound **9** -HSQC*

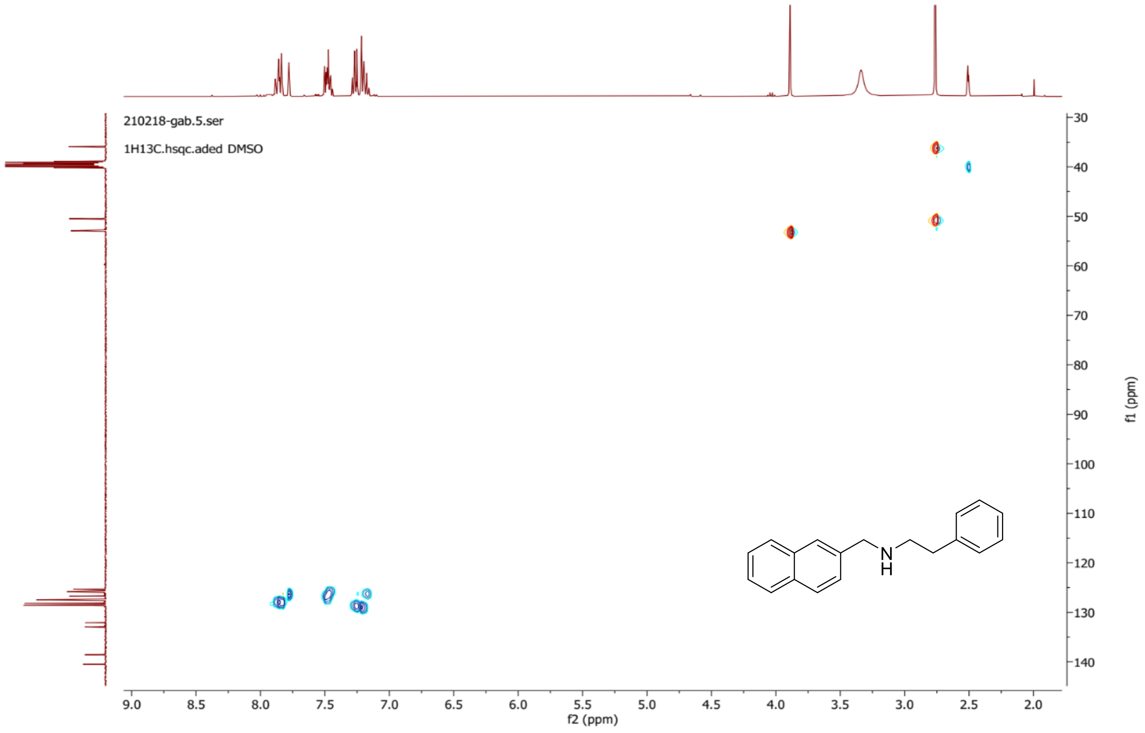

*9. (Compound **24** -^{1}HNMR)*

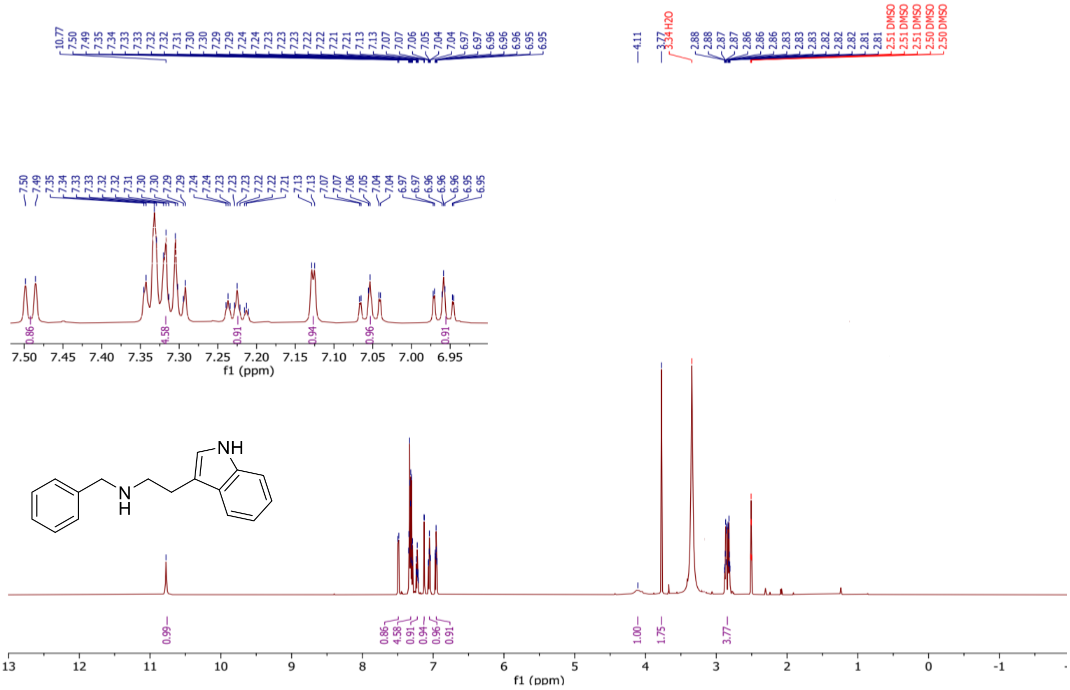

*10. Compound **24**- ^{13}CNMR*

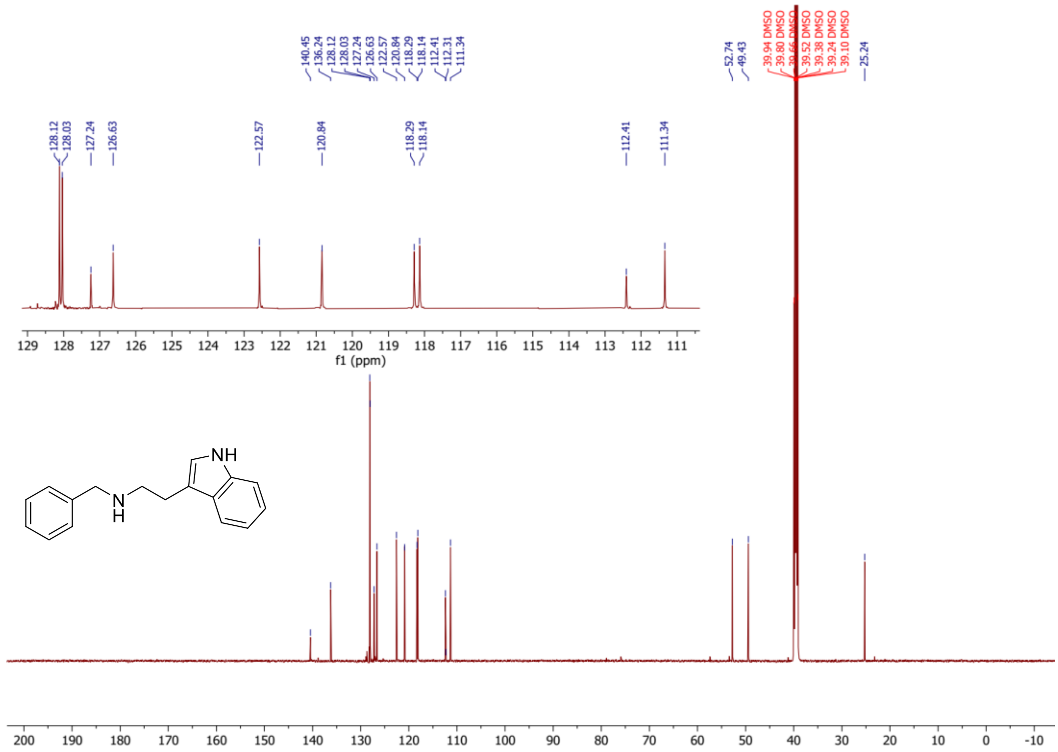

*11. Compound **24**- ^{13}CNMR-dept 135*

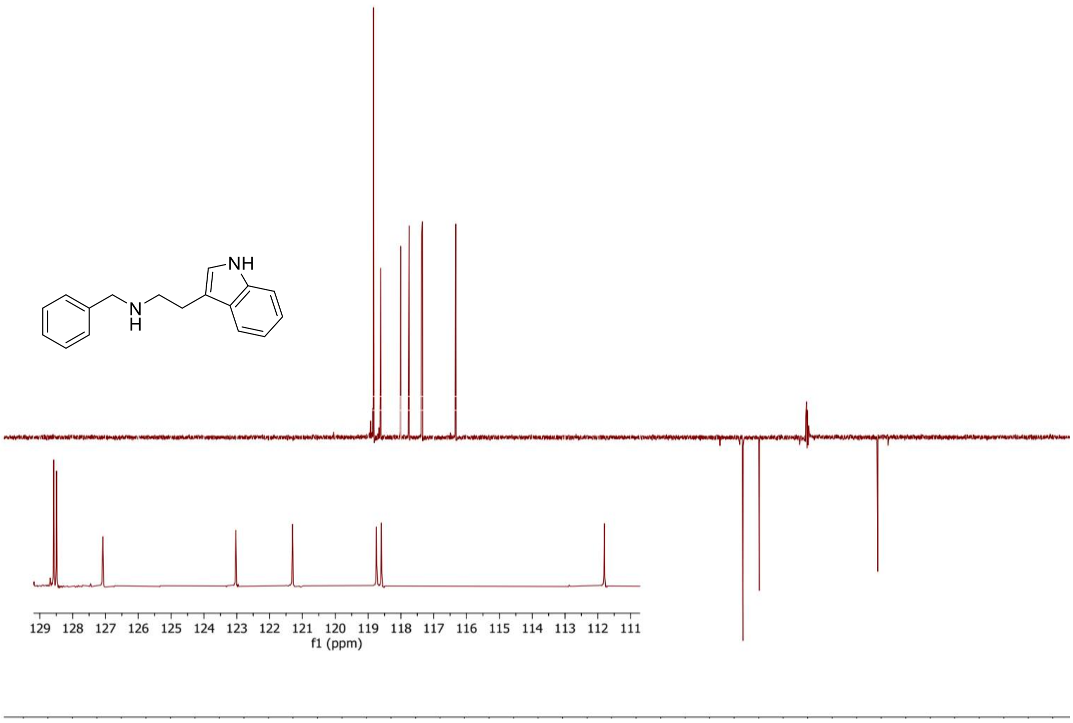

*12. Compound **24**- ^{13}CNMR-dept 90*

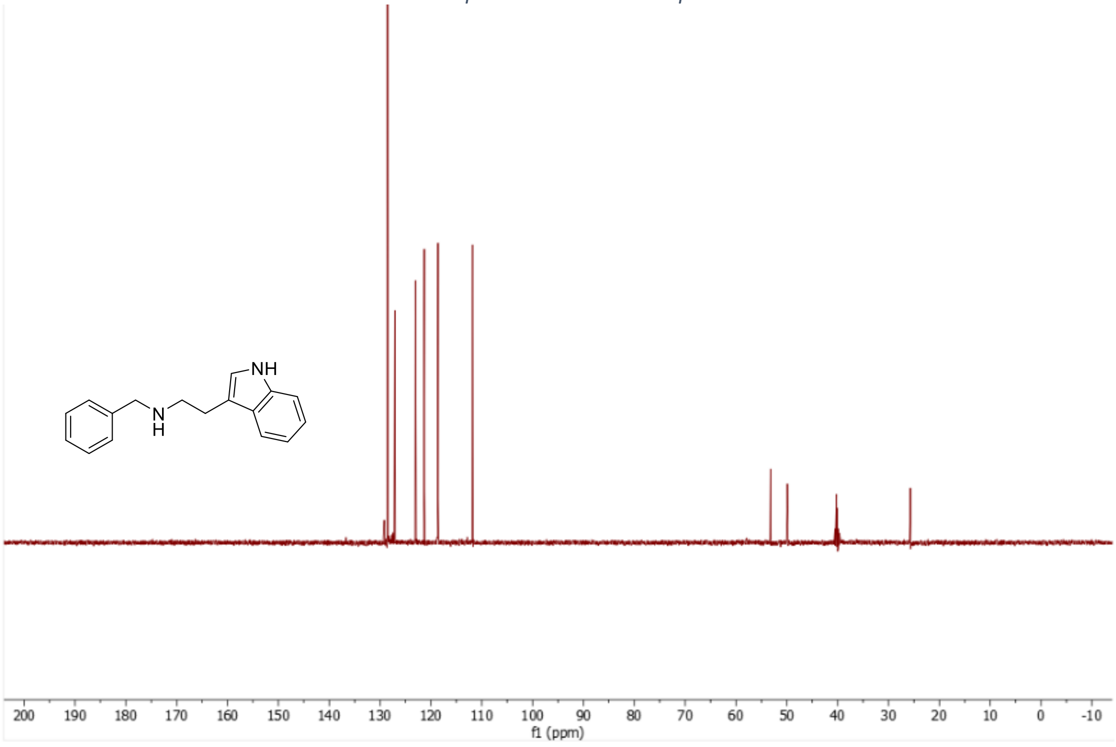

7- ^{1}H, and ^{13}CNMR of Boc-naphthyl-indole and naphthyl-phenyl based peptoids.

*13. Compound **11a**- ^{1}HNMR*

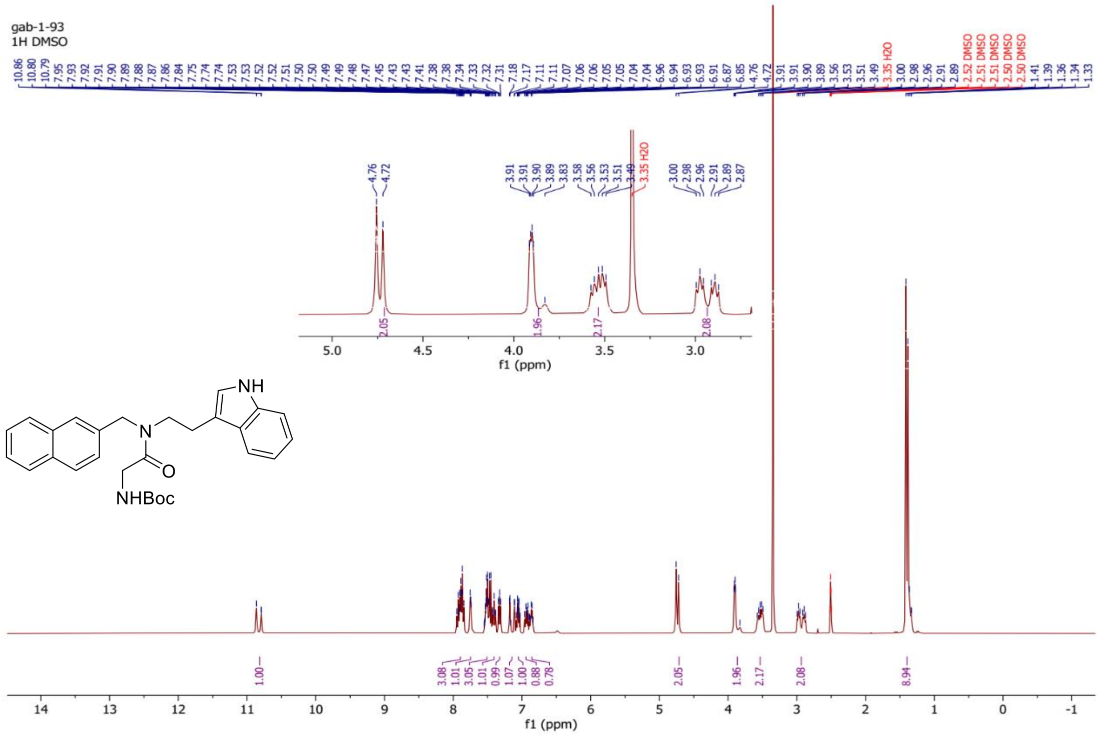

*14. Compound **11a**- ^{13}CNMR*

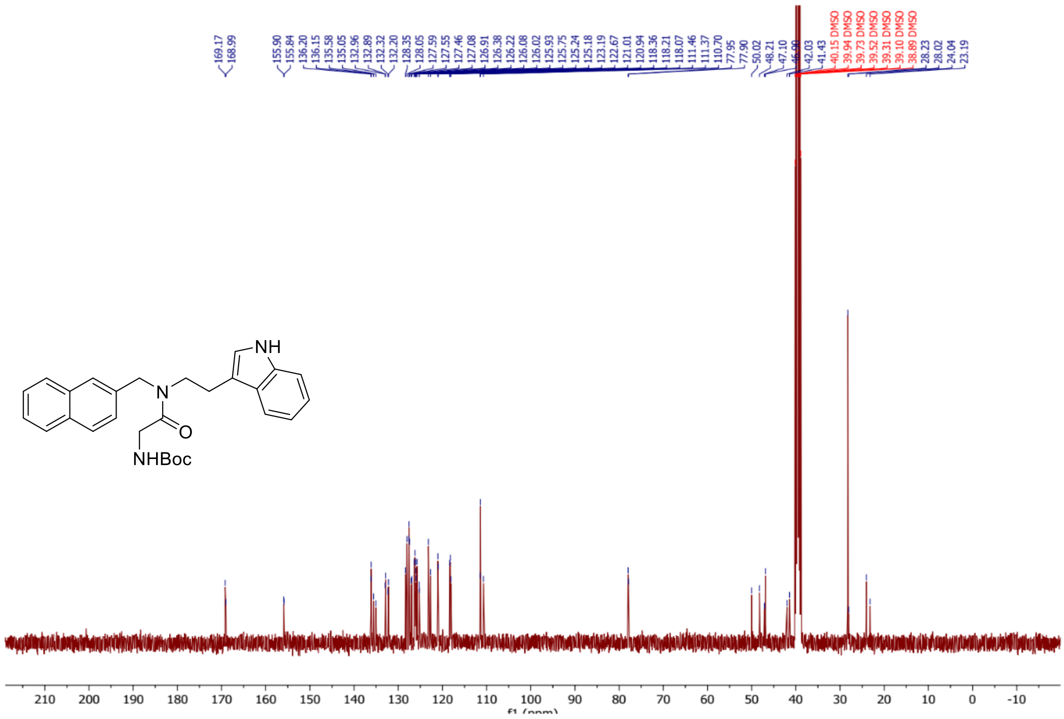

*15. Compound **11a**- ^{13}CNMR-dept 135*

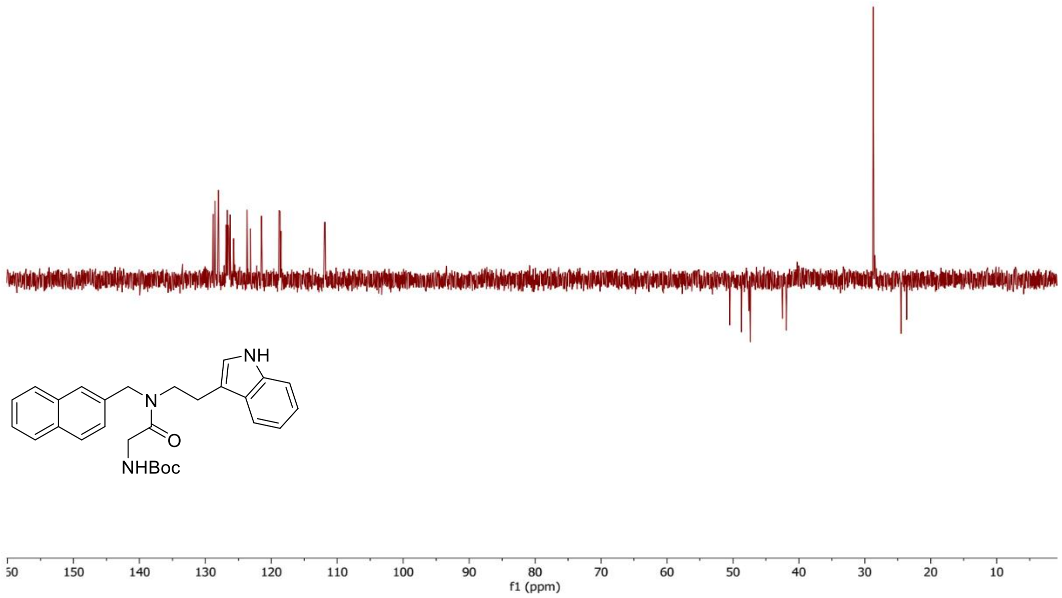

*16. Compound **11a**- -HSQC*

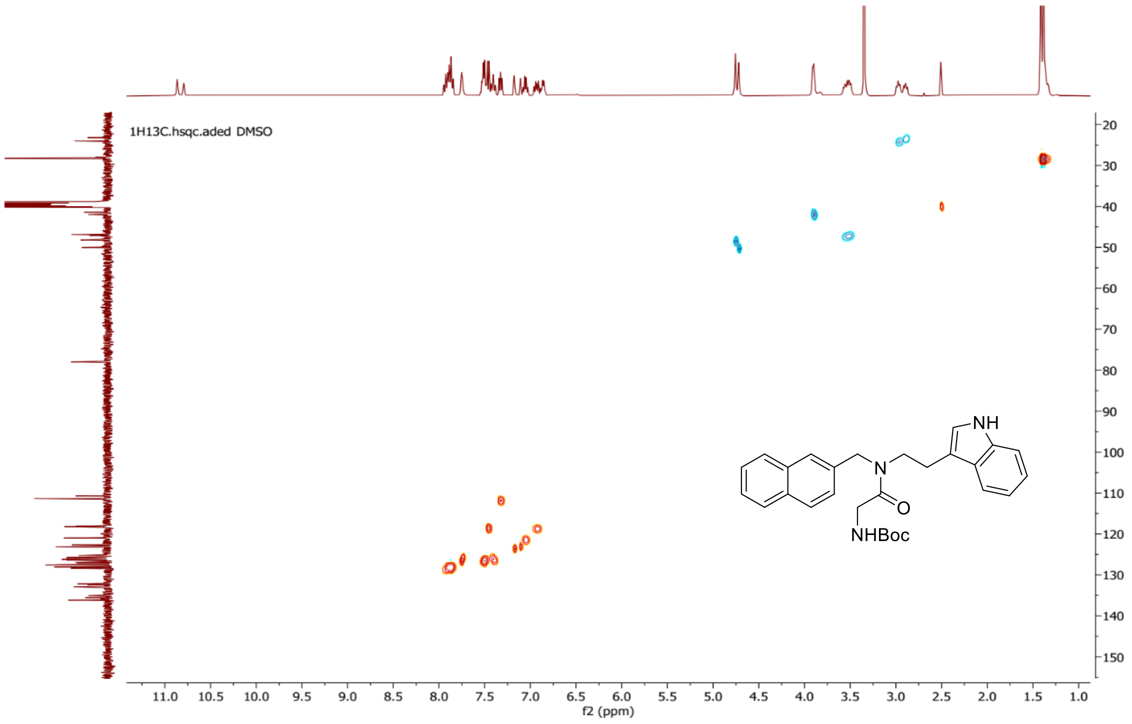

17. Compound ***11b*** *-^{1}HNMR*

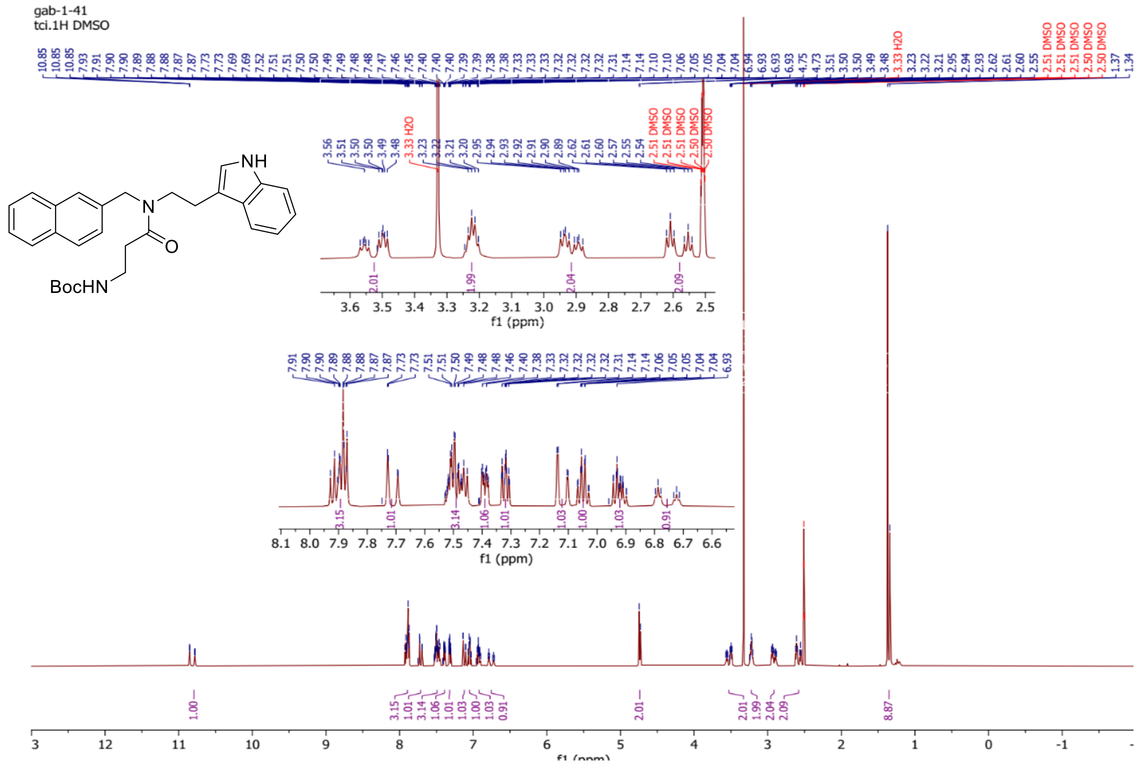

18. Compound ***11b*** *-^{13}CNMR*

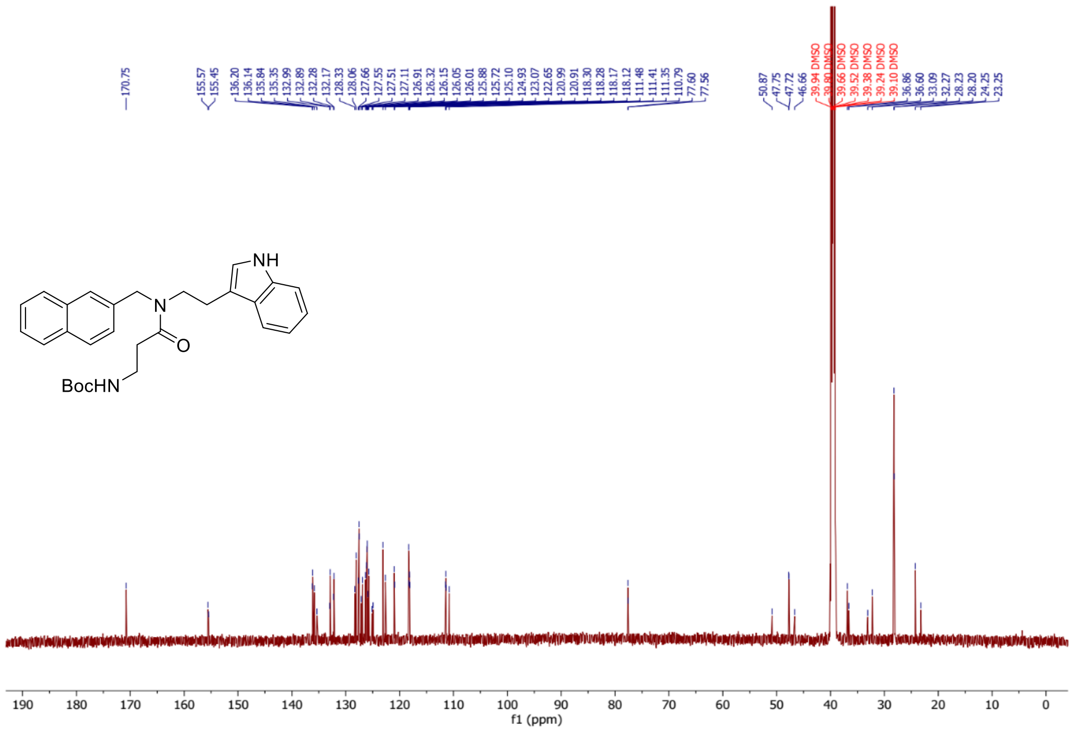

*19. Compound **11b** -^{13}CNMR dept135*

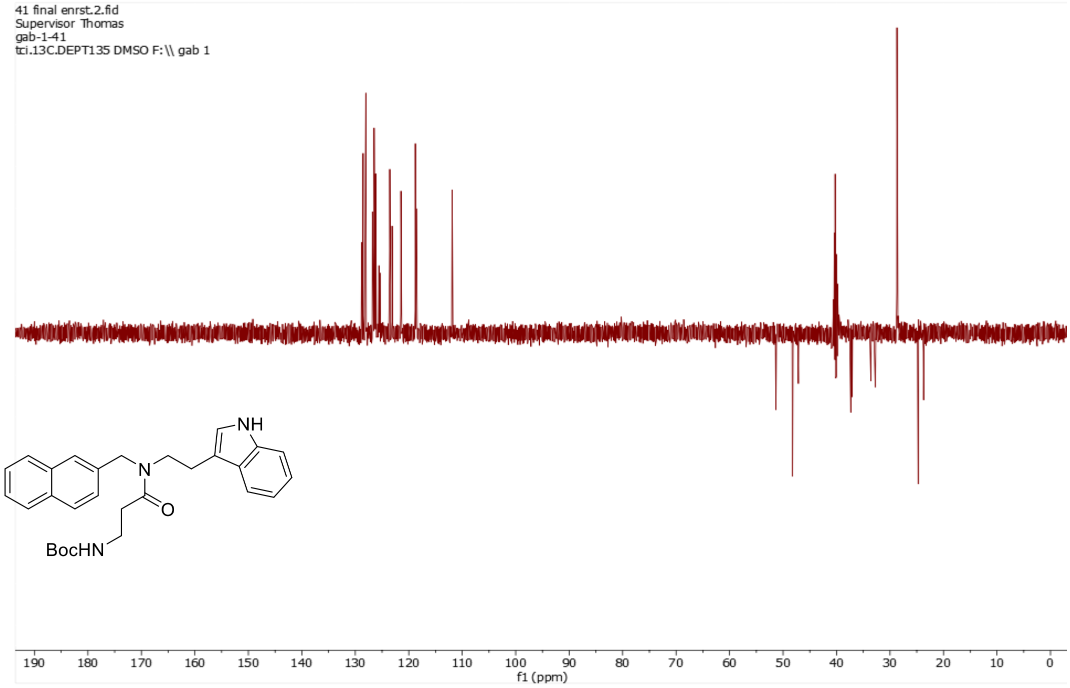

*20. Compound **11b** -HSQC*

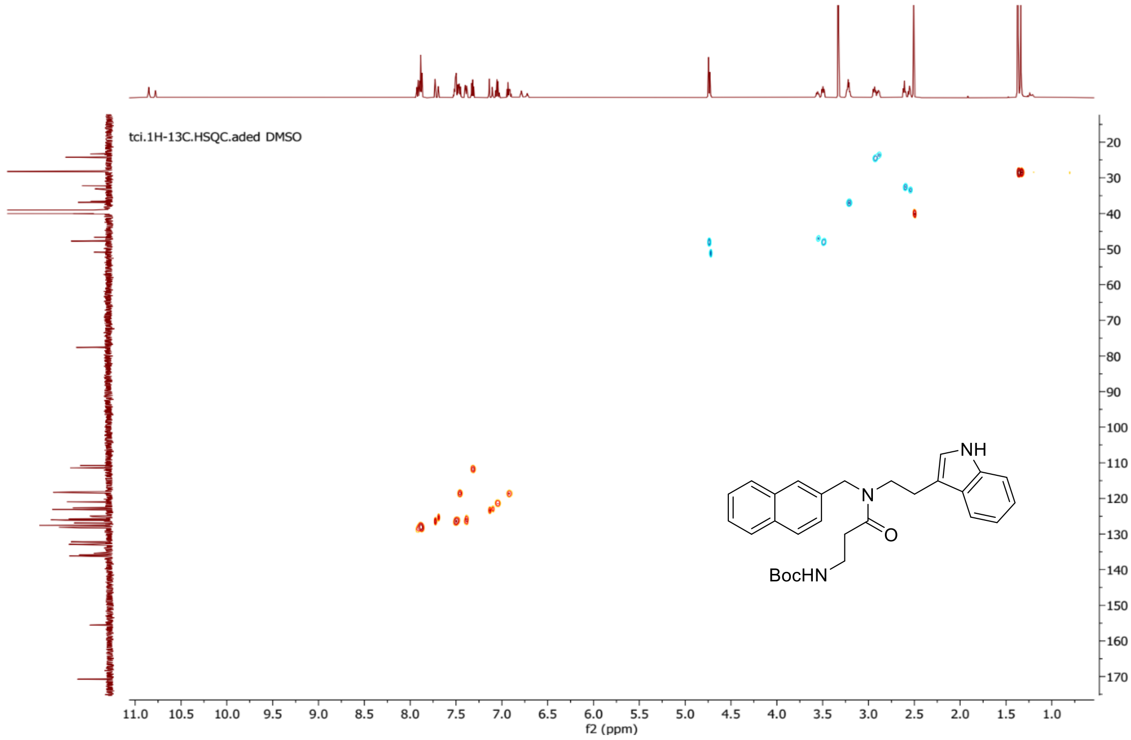

*21. Compound **11c** -^{1}HNMR*

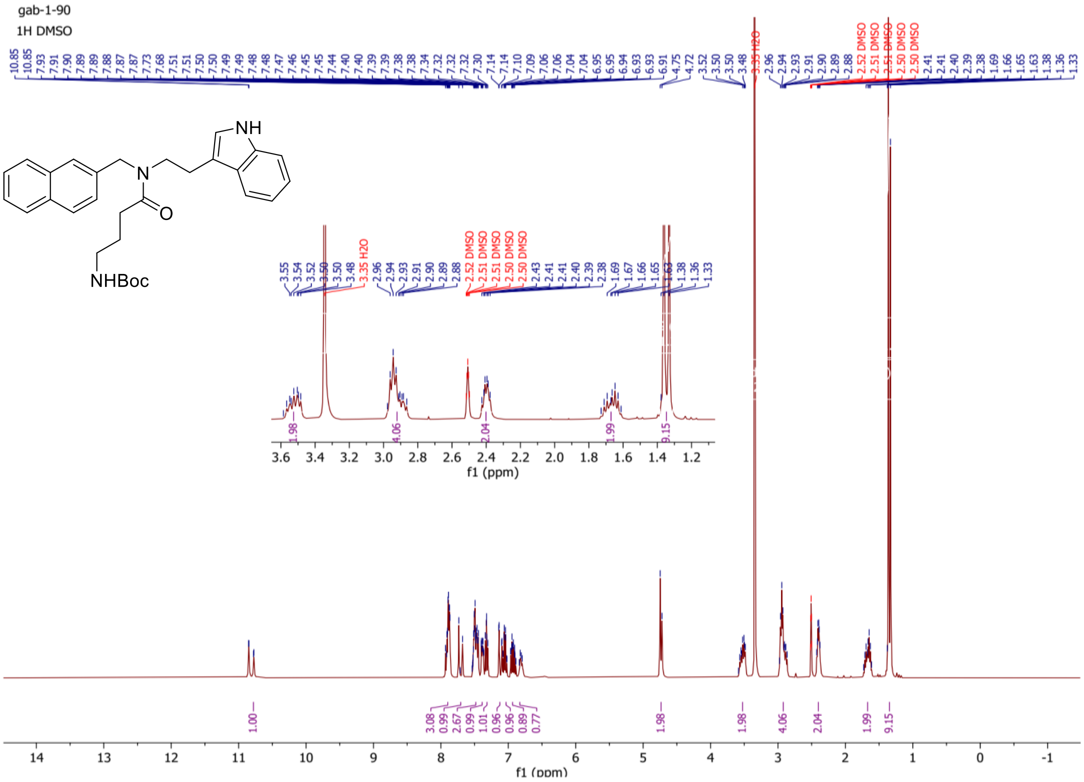

*22. Compound **11c** - ^{13}CNMR*

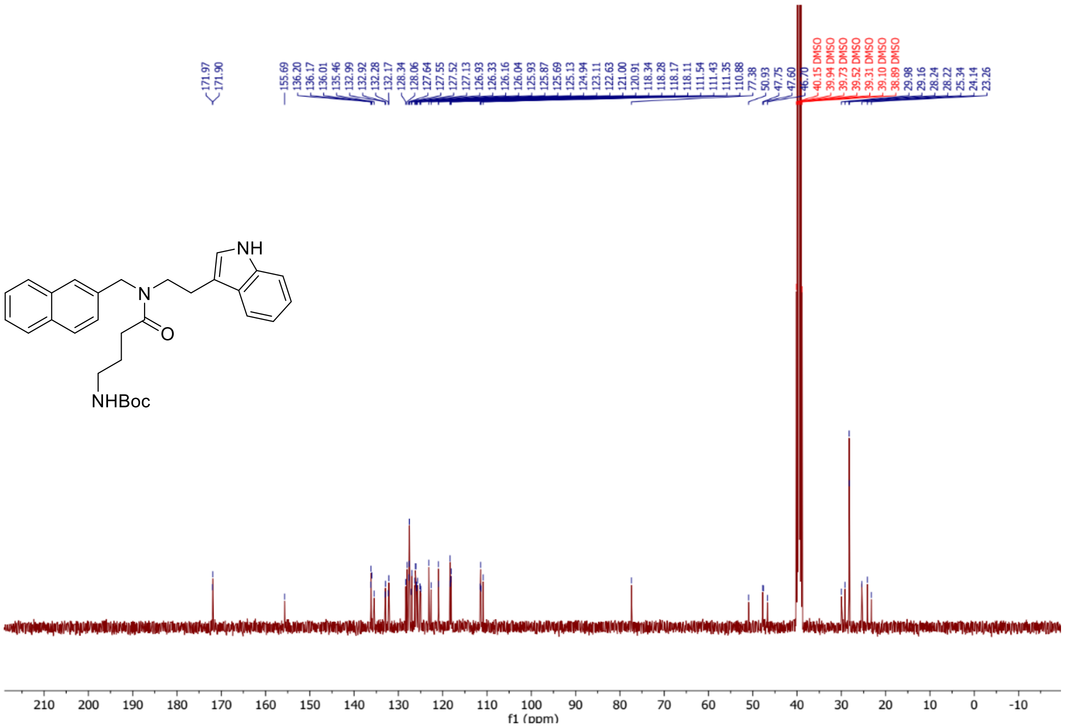

*23. Compound **11c** -^{13}CNMR dept 135*

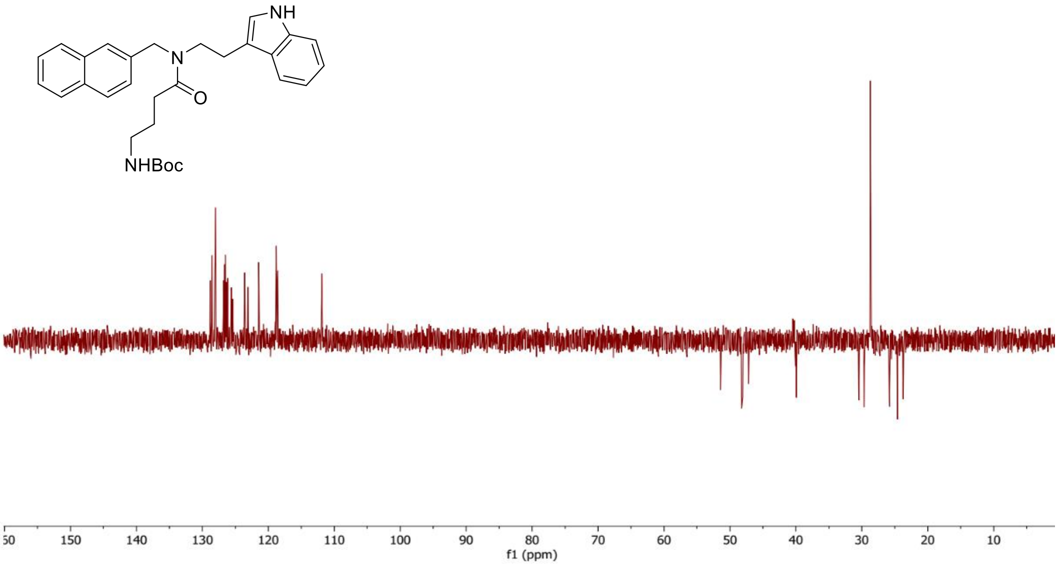

*24. (Compound **11c** - -HSQC)*

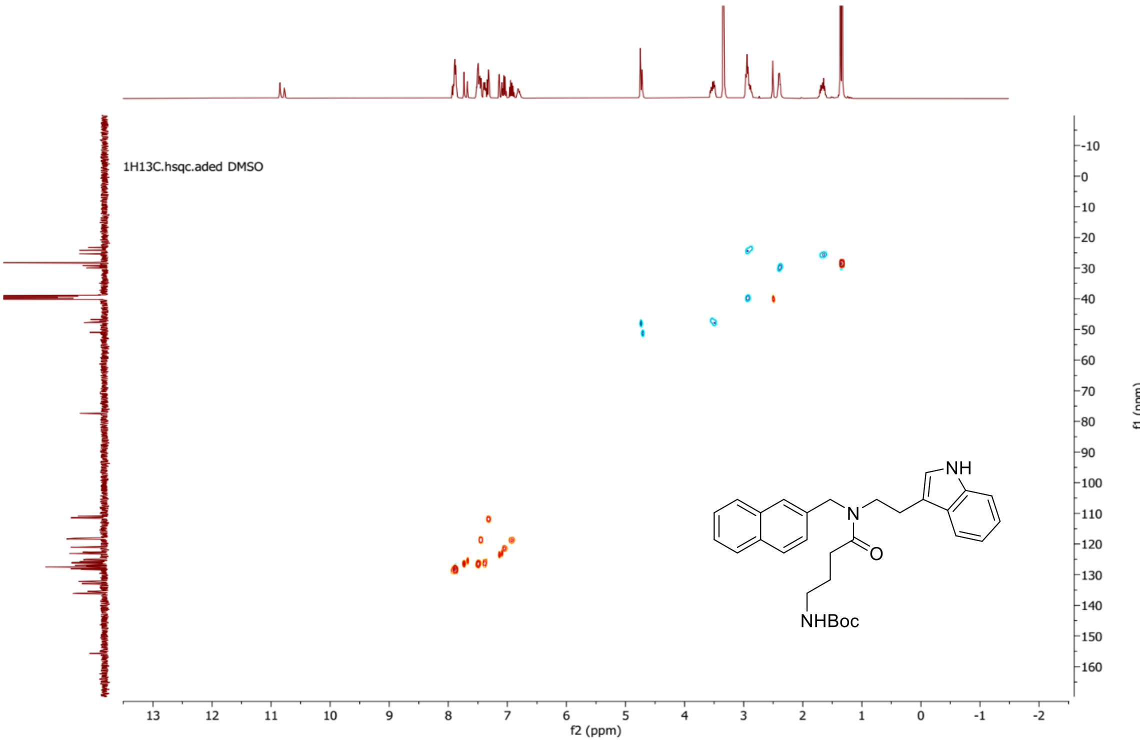

*25. (Compound **11d** - ^{1}HNMR)*

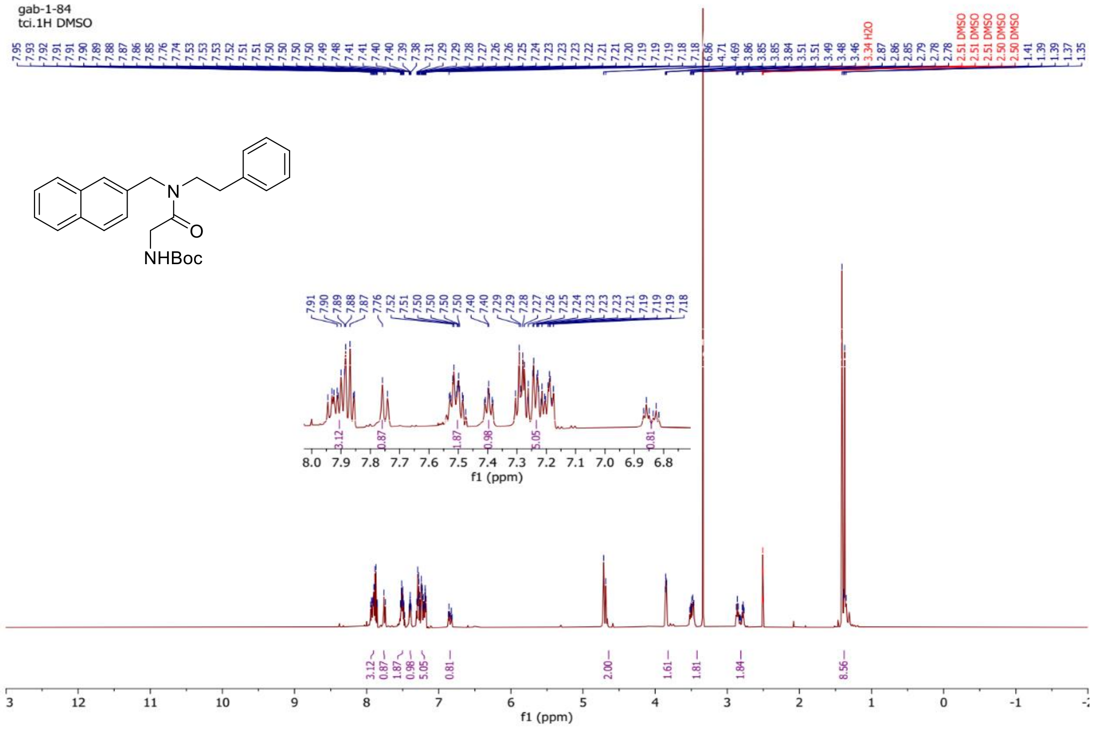

*26. Compound **11d** - ^{13}CNMR*

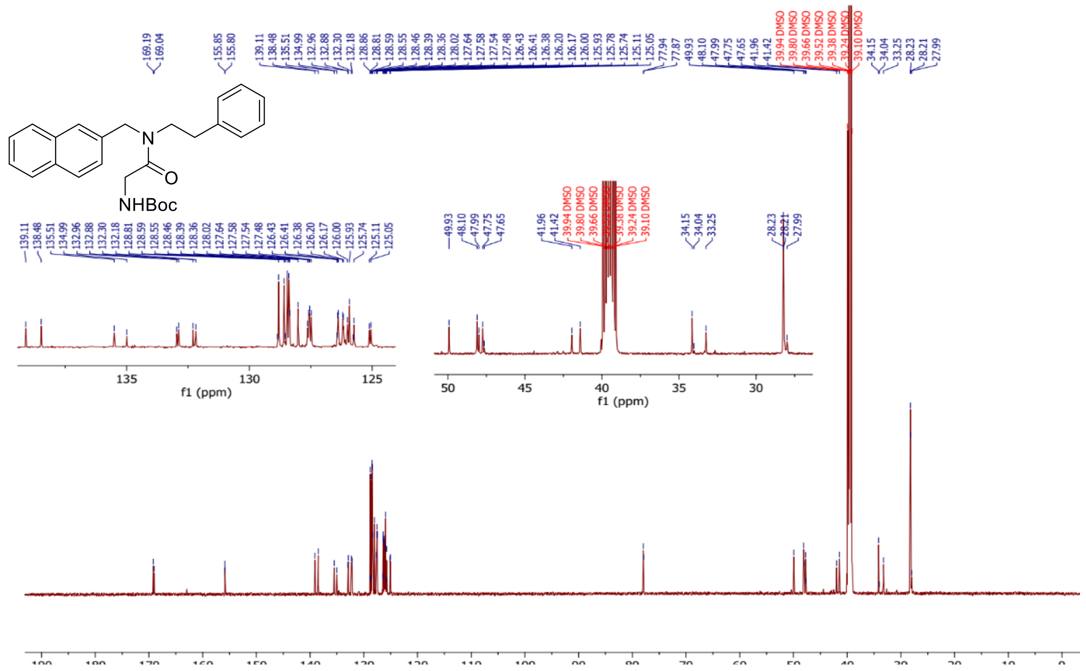

*27. Compound **11d** - ^{13}CNMR dept 135*

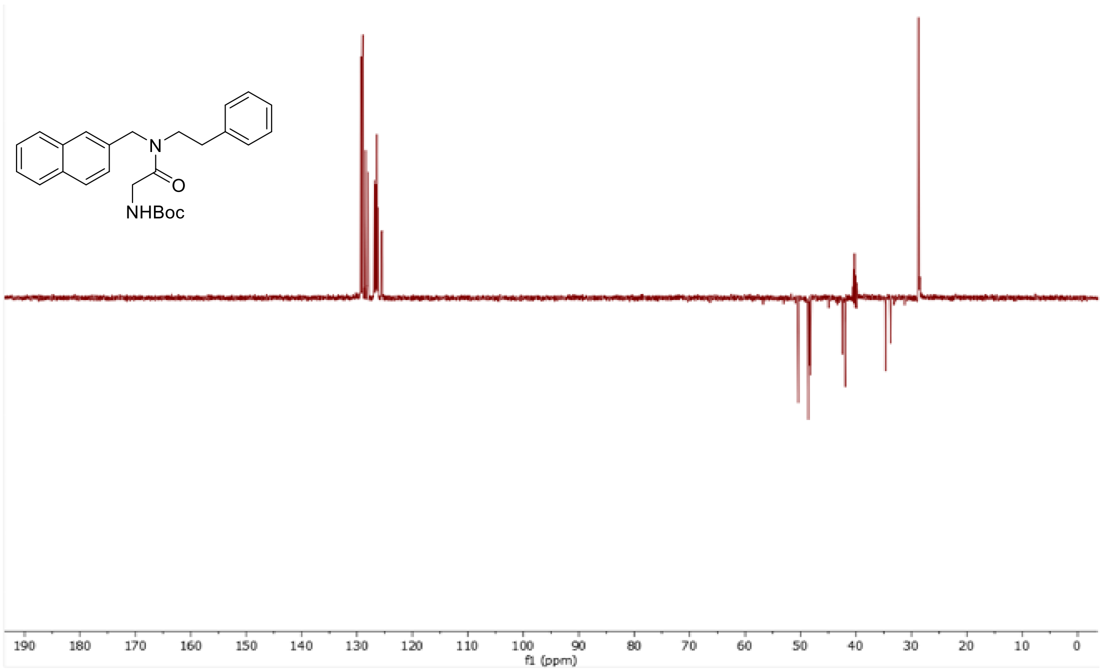

*28. Compound **11d** - HSQC)*

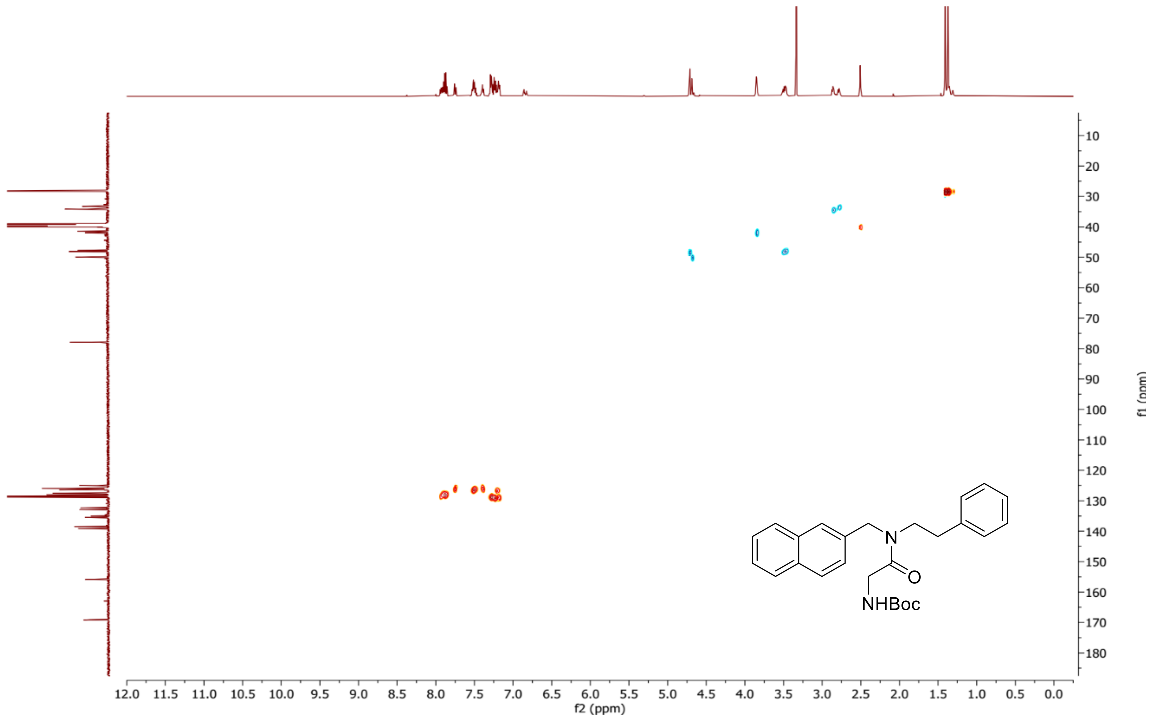

*29. Compound **11e** - ^{1}HNMR*

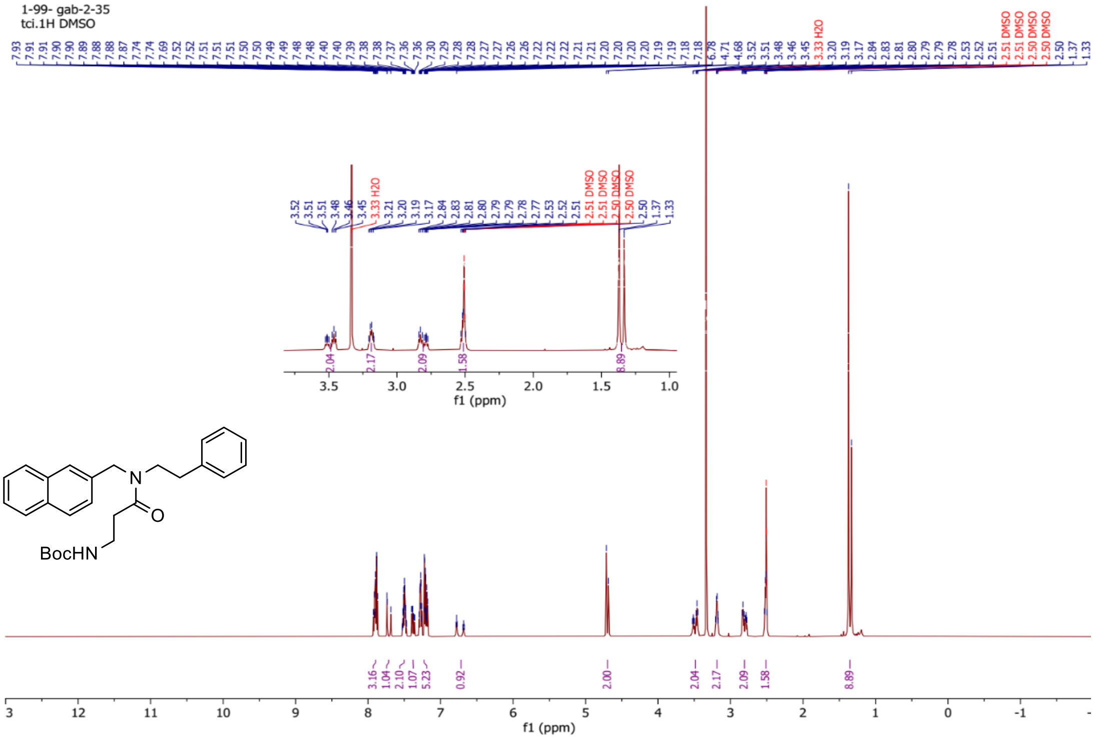

*30. Compound **11e** - ^{13}CNMR*

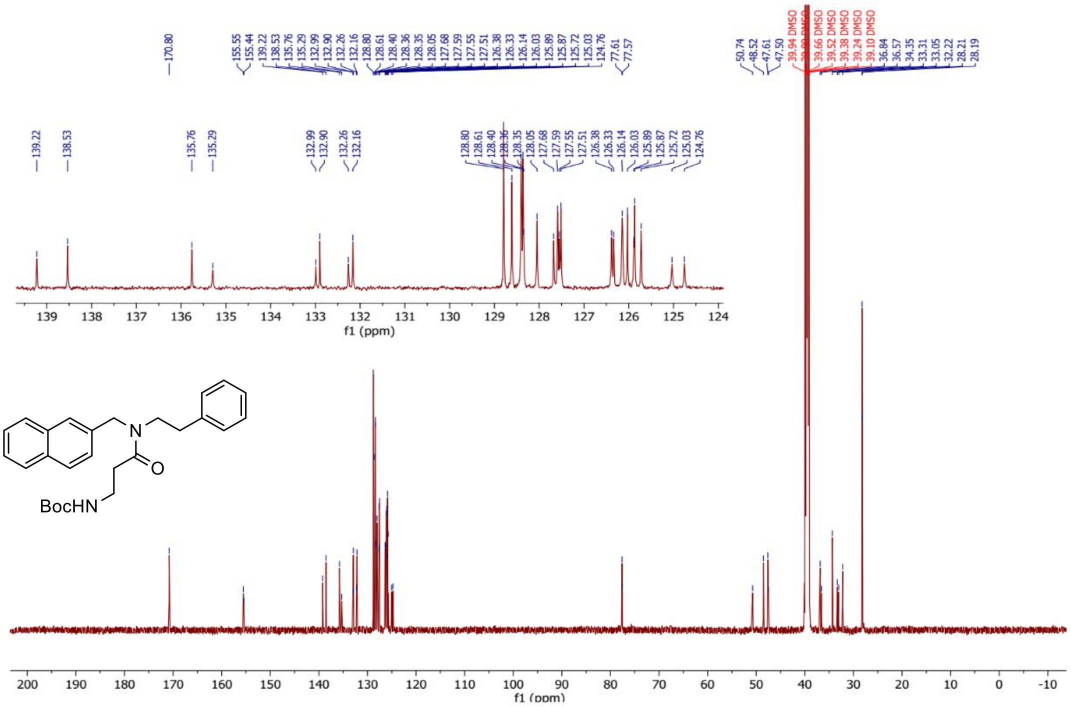

*31. Compound **11e** - ^{13}CNMR-dept 135*

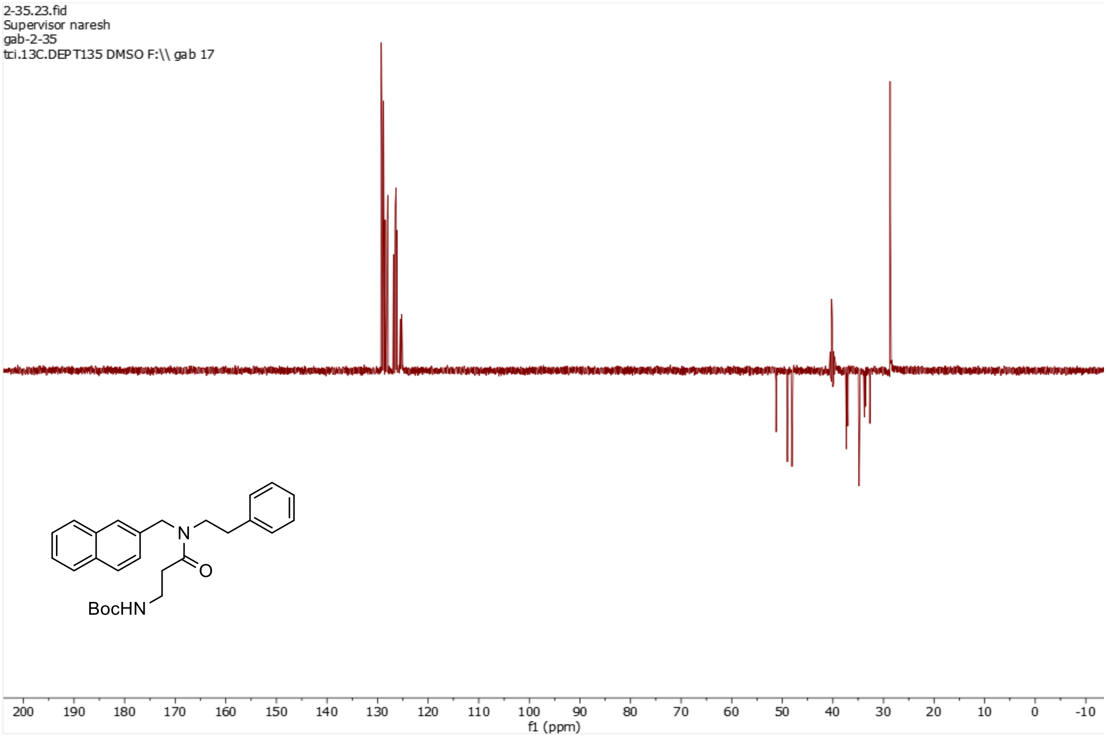

*32. Compound **11e** - HSQC*

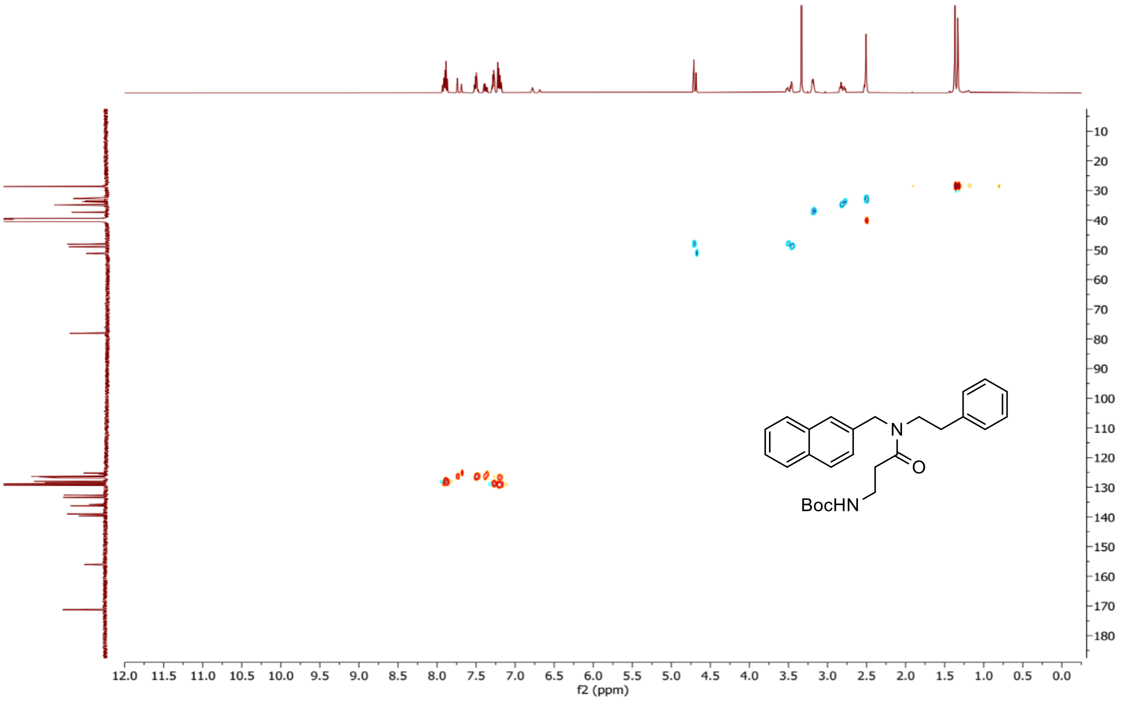

*33. Compound **11f** - ^{1}HNMR)*

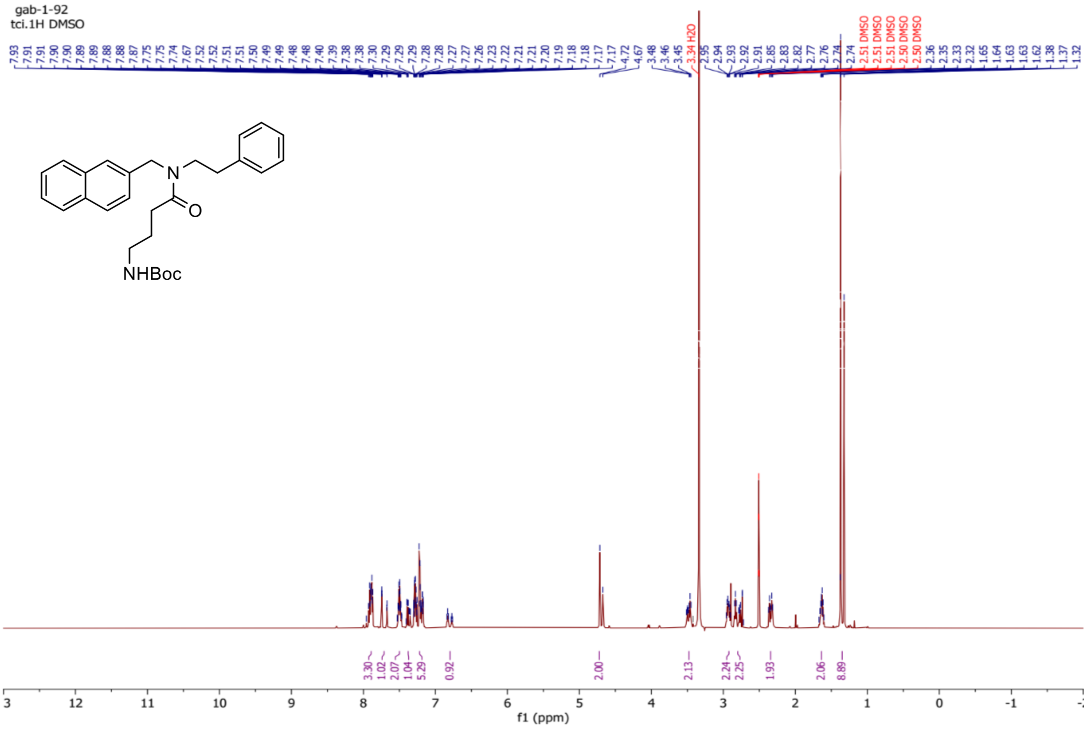

*34. Compound **11f** -^{13}CNMR*

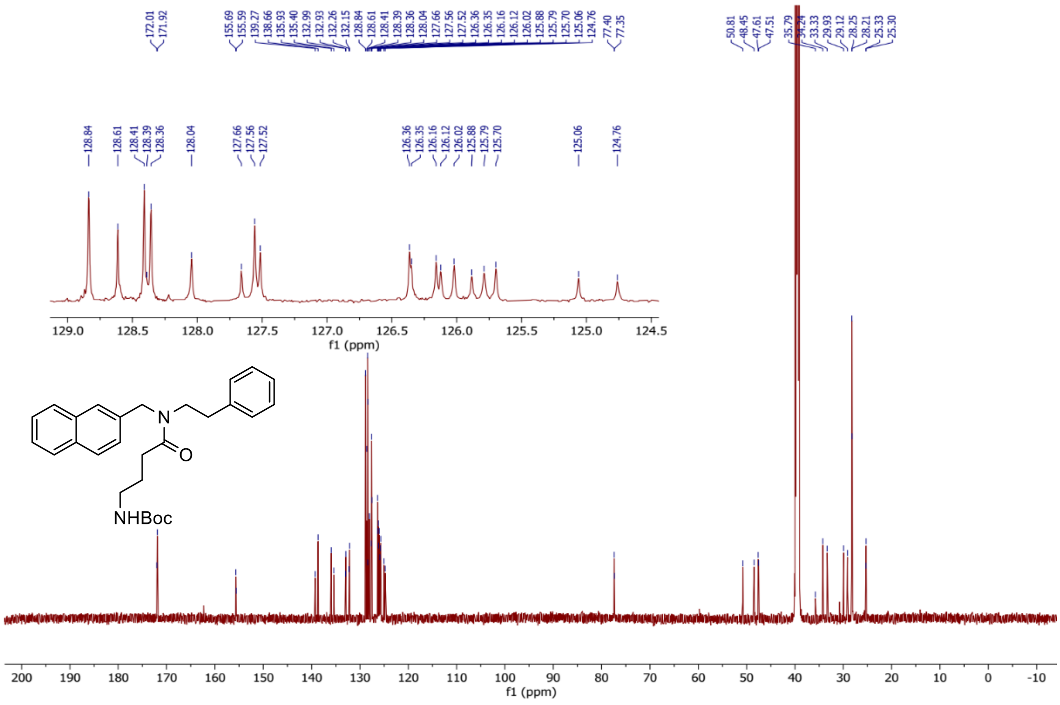

*35. Compound **11f** - ^{13}CNMR-dept 135*

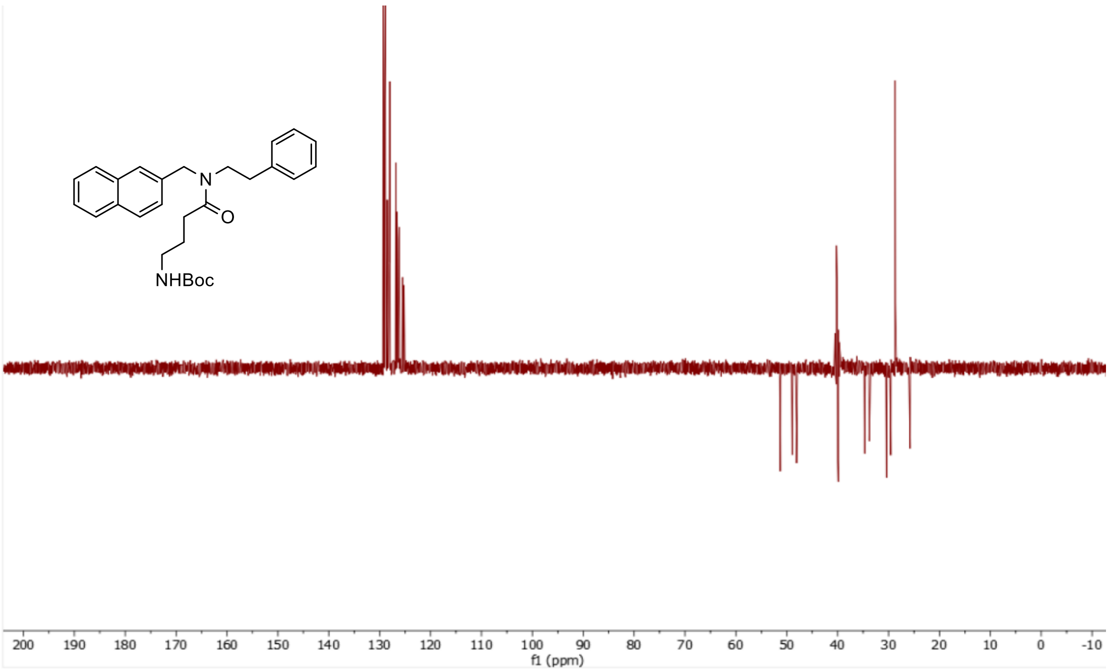

*36. Compound **11f** -HSQC)*

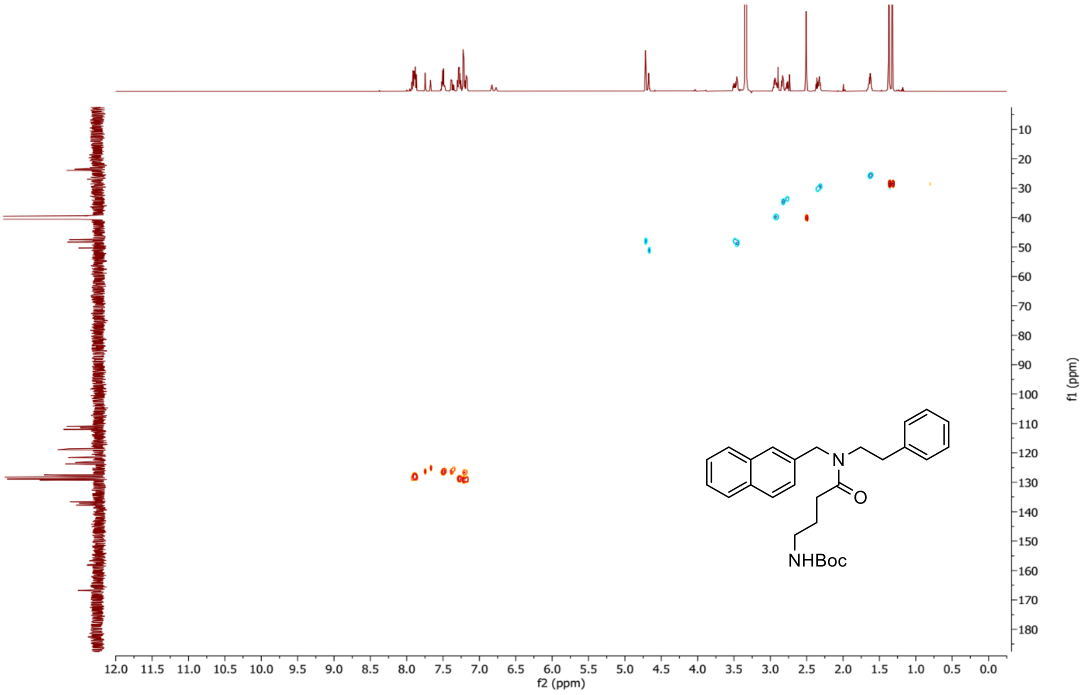

*37. Compound **25**-^{1}HNMR*

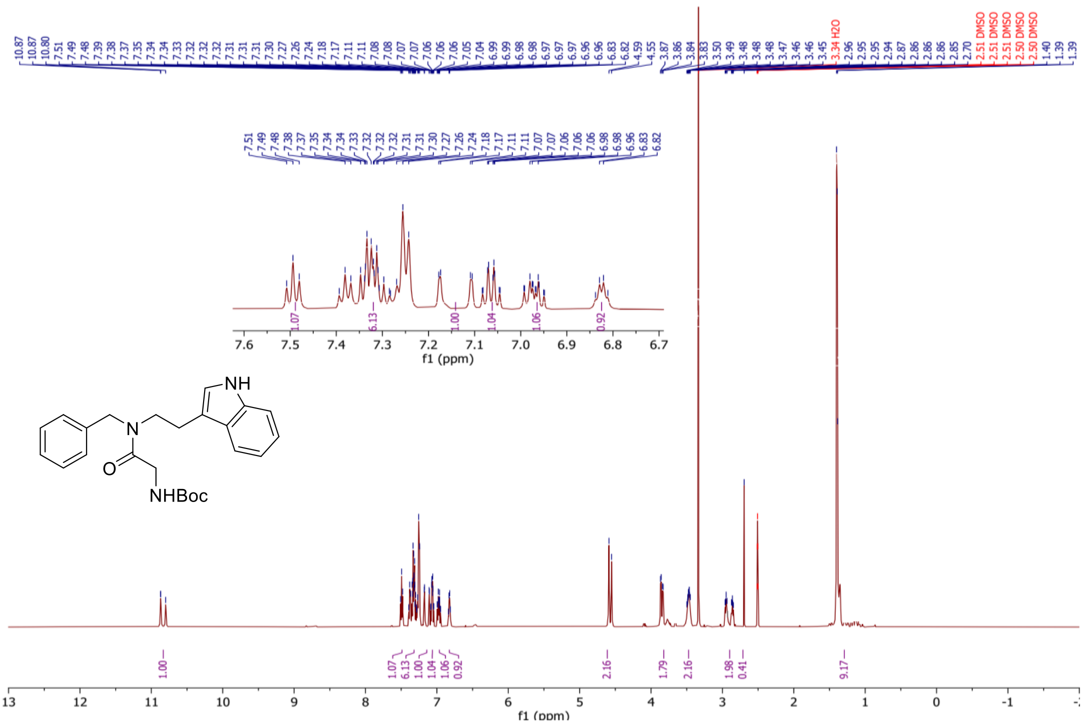

*38. Compound **25** -^{13}CNMR*

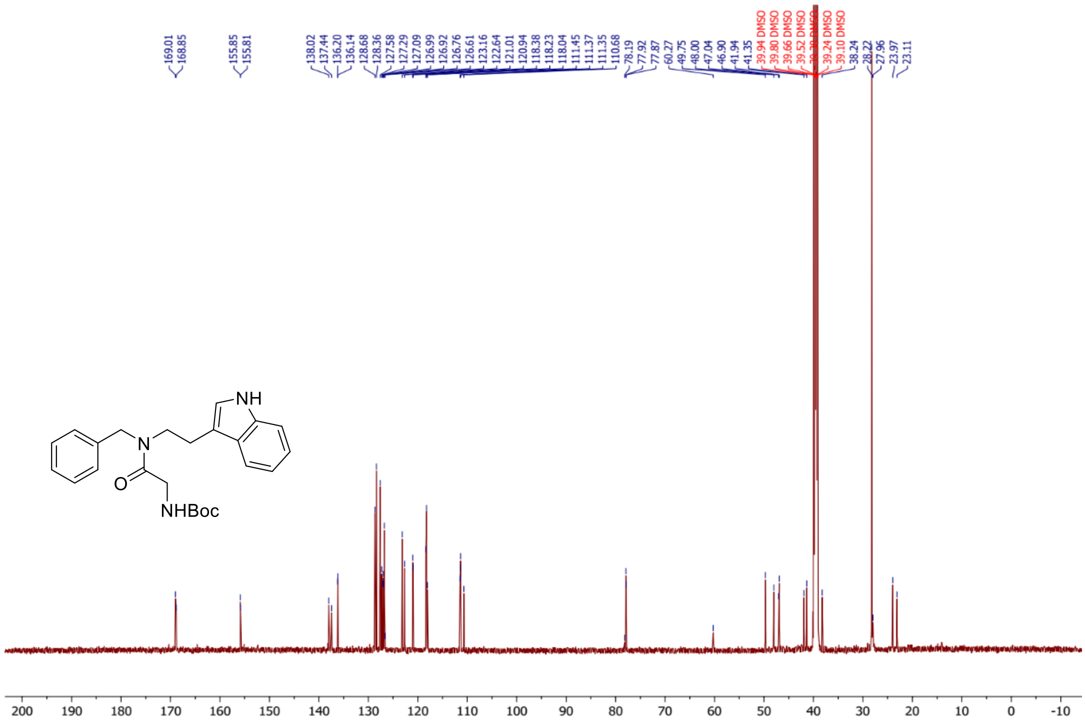

*39. Compound **25** -^{13}CNMR dept 135*

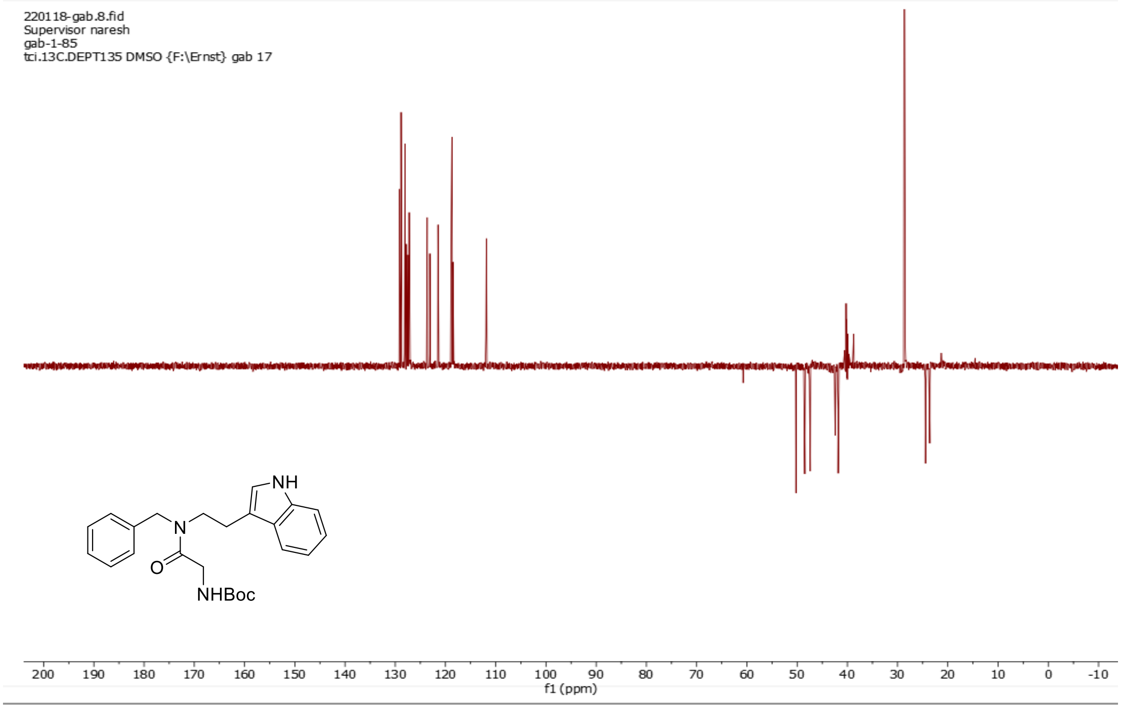

*40. Compound **25** -HSQC*

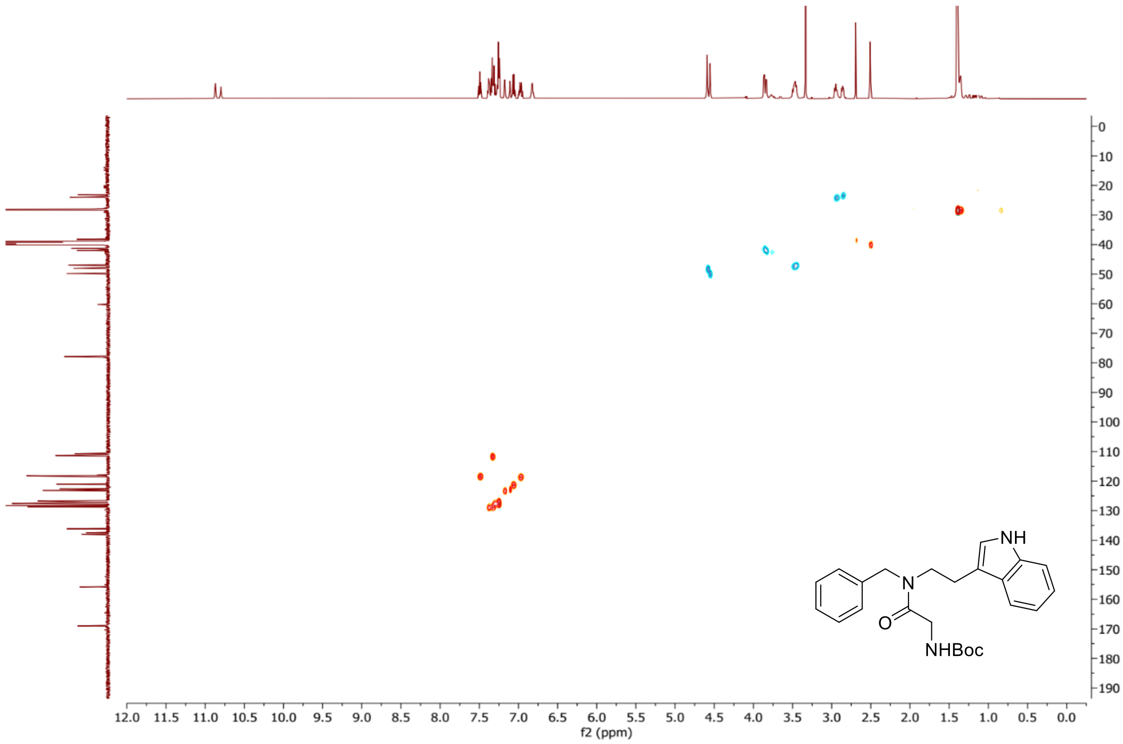

*41. Compound **18b** -^{1}HNMR*

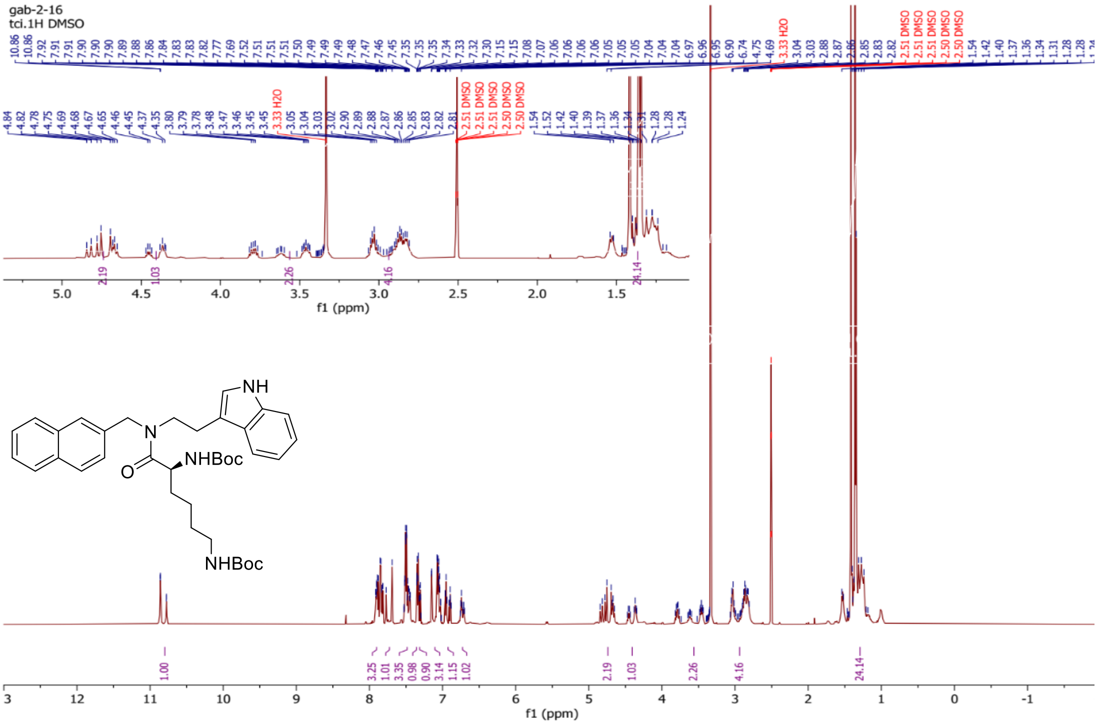

*42. Compound **18b**- ^{13}CNMR*

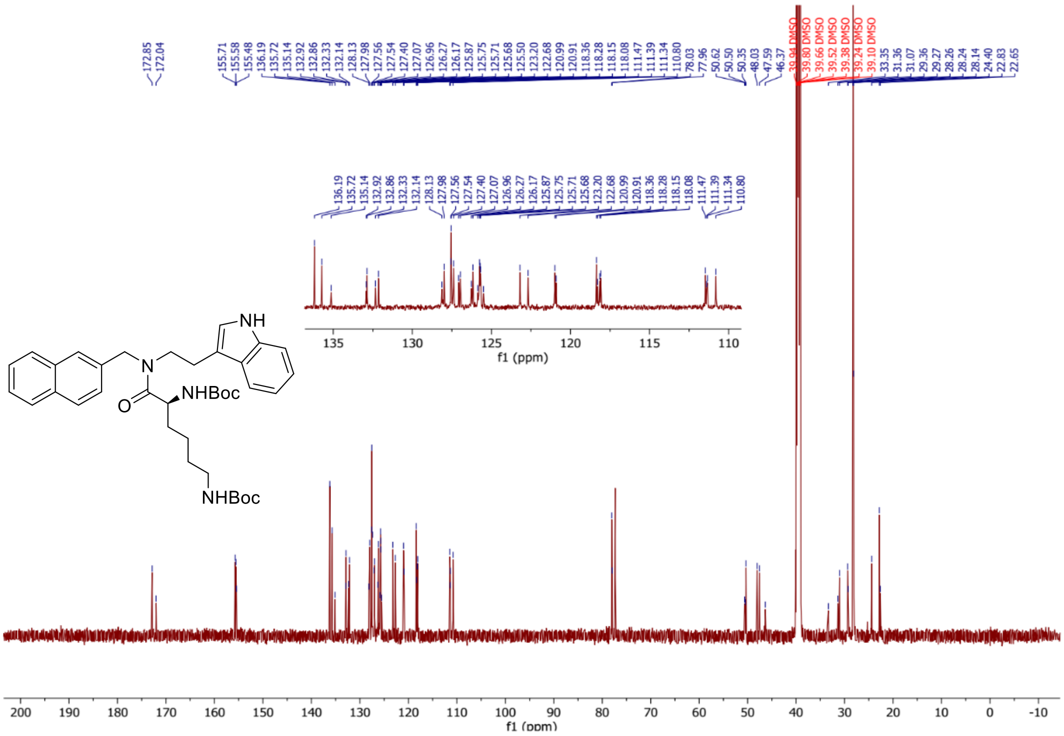

*43. Compound **18b**- ^{13}CNMR dept 135*

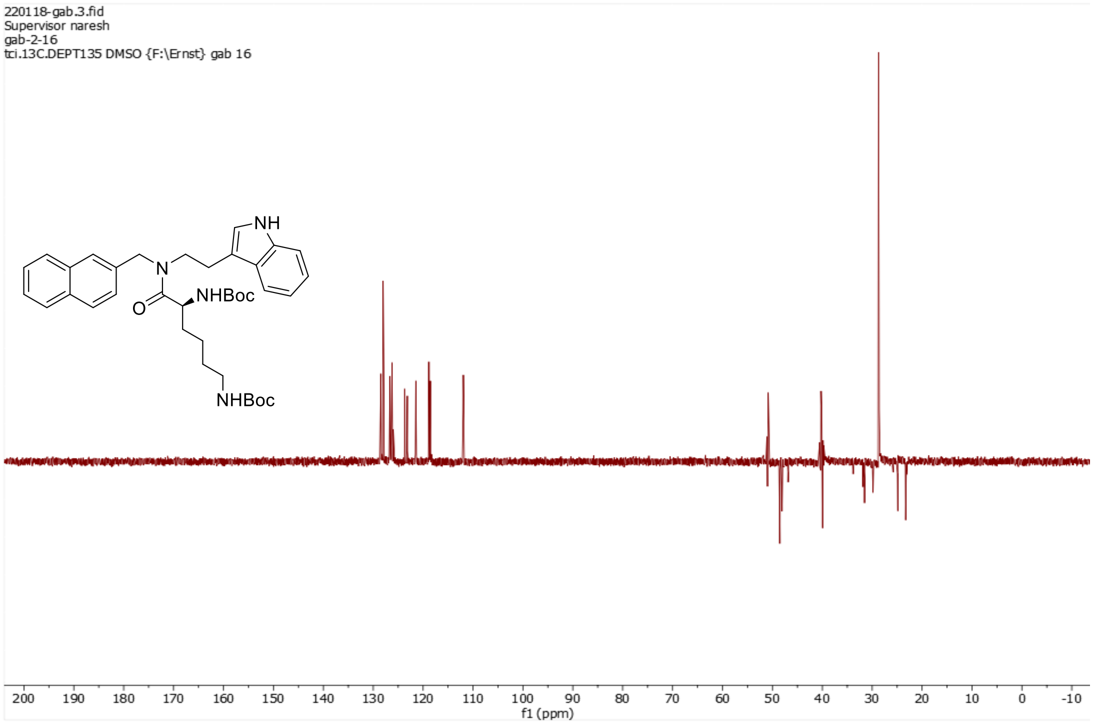

*44. Compound **18b**- HSQC*

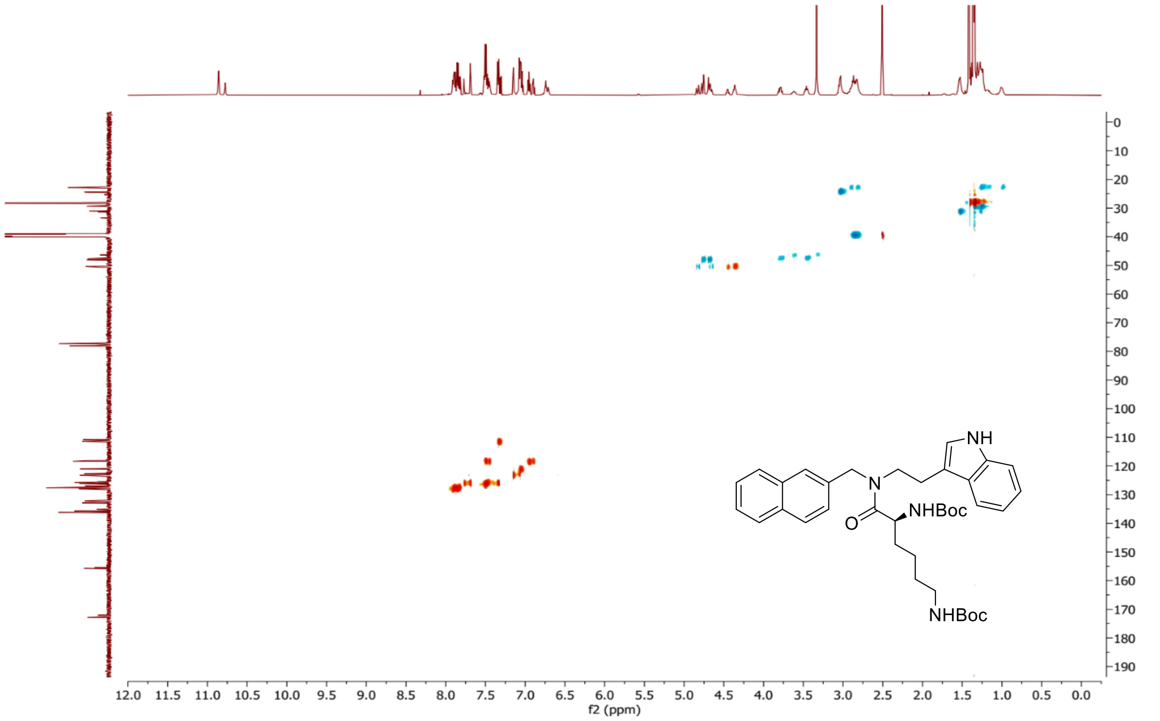

*45. Compound **18a** -^{1}HNMR*

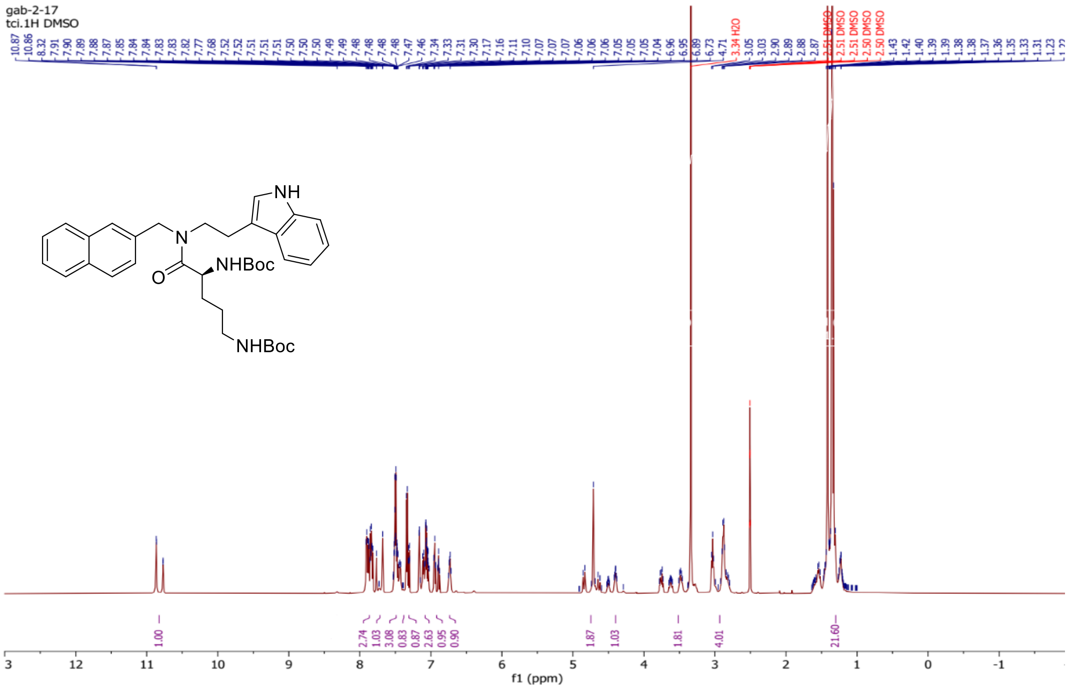

*46. Compound **18a** -^{13}CNMR*

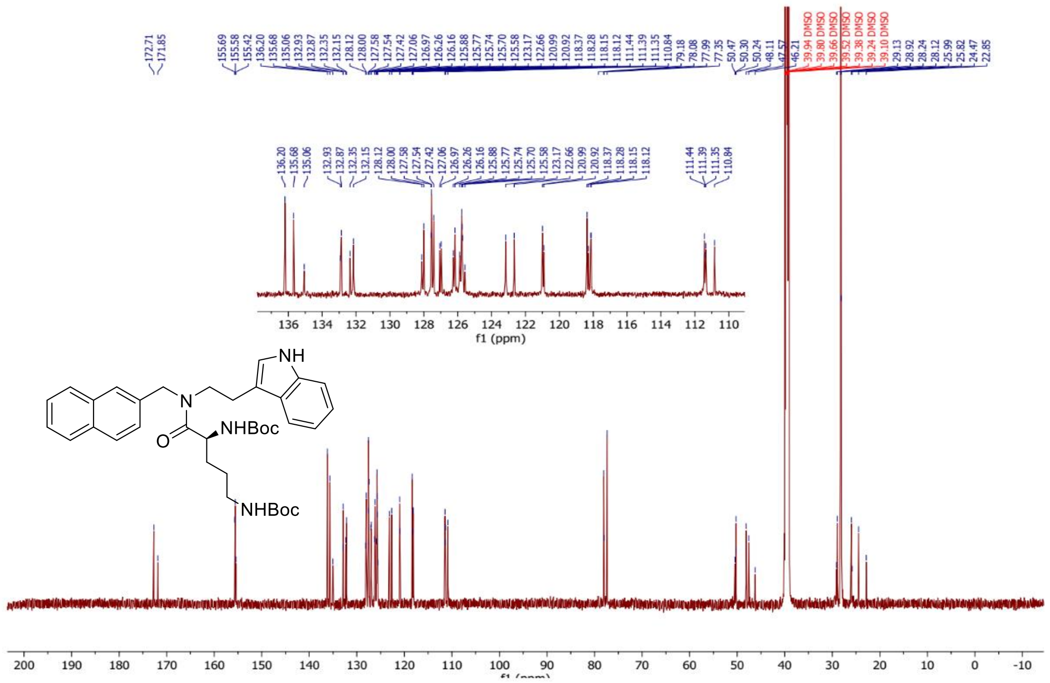

*47. Compound **18a** -^{13}CNMR dept 135*

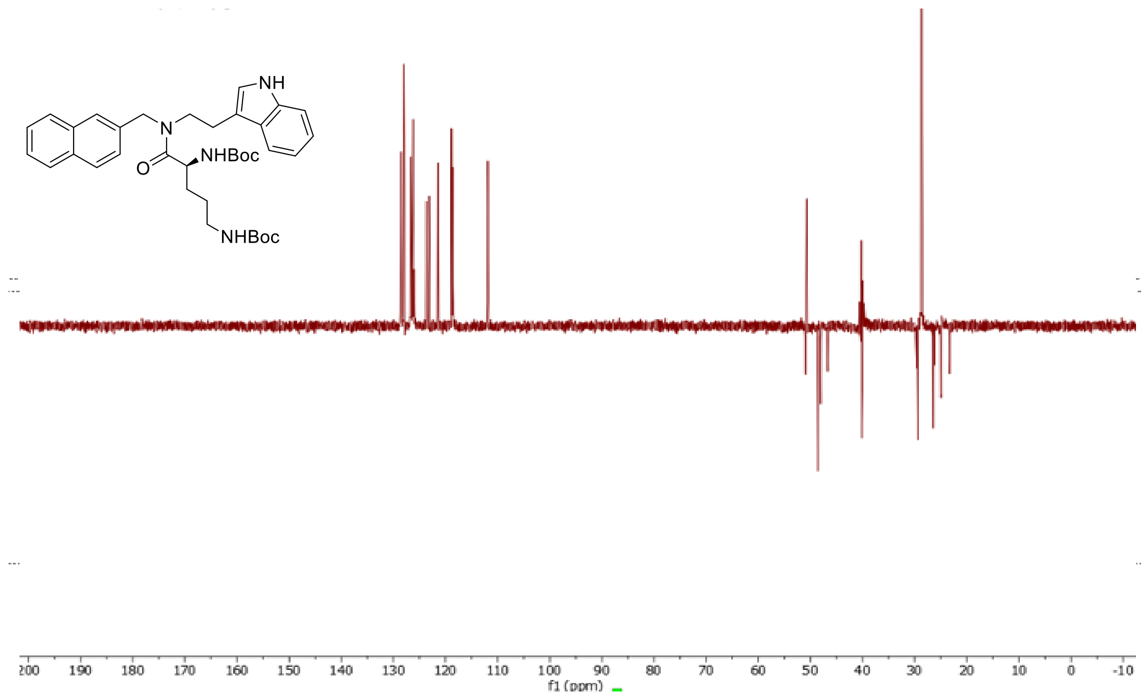

*48. Compound **18a** - HSQC*

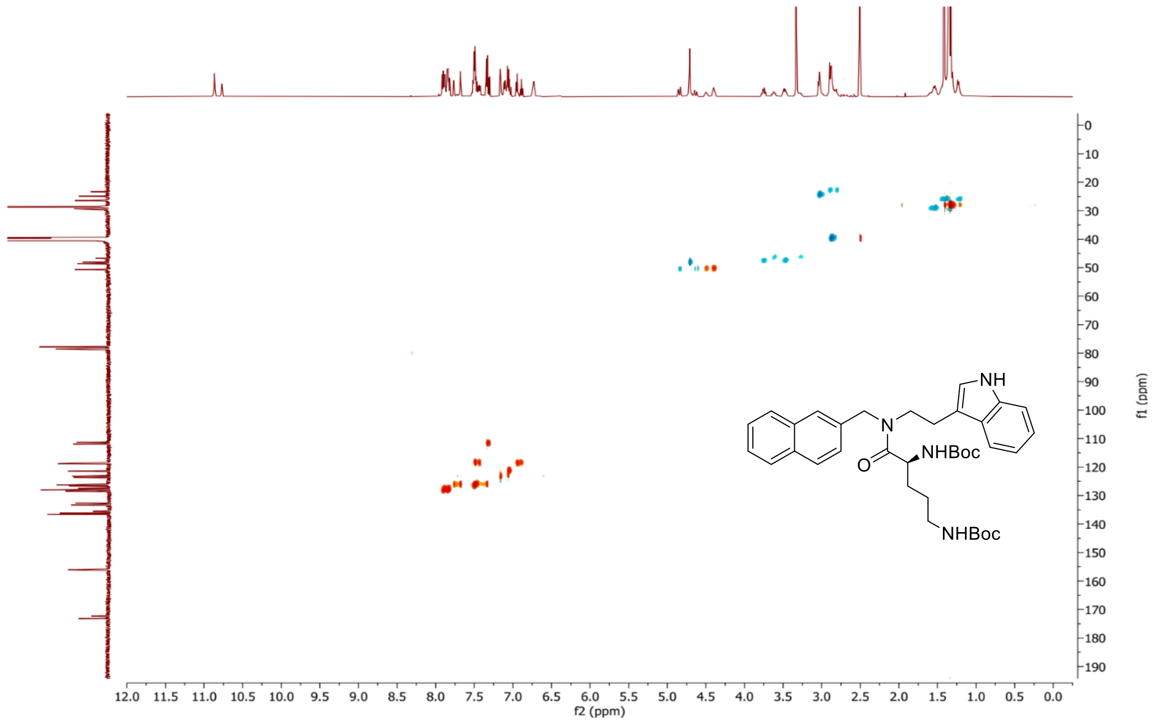

*49. Compound **21** -^{1}HNMR*

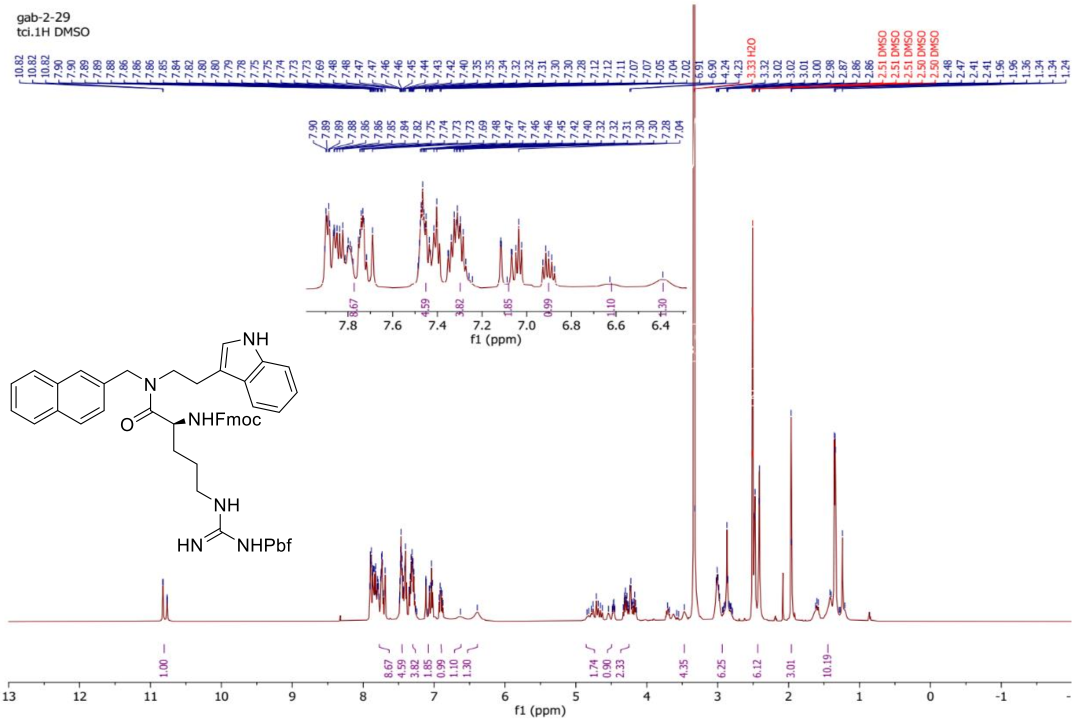

*50. Compound **21** -^{13}CNMR*

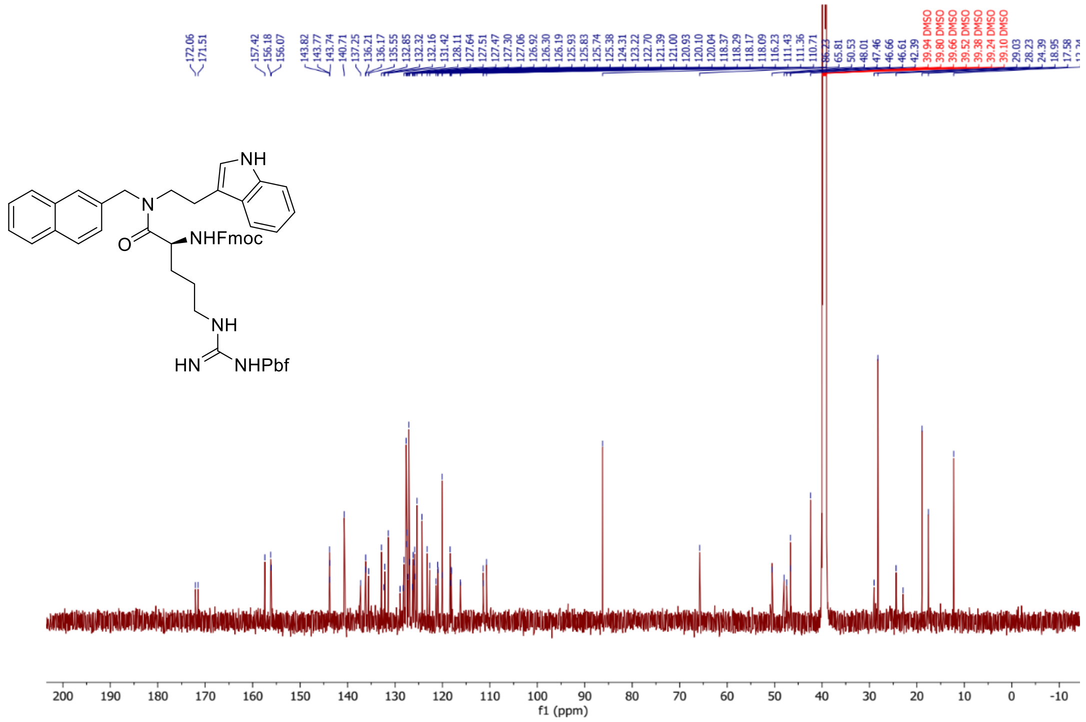

*51. Compound **21** -^{13}CNMR dept 135*

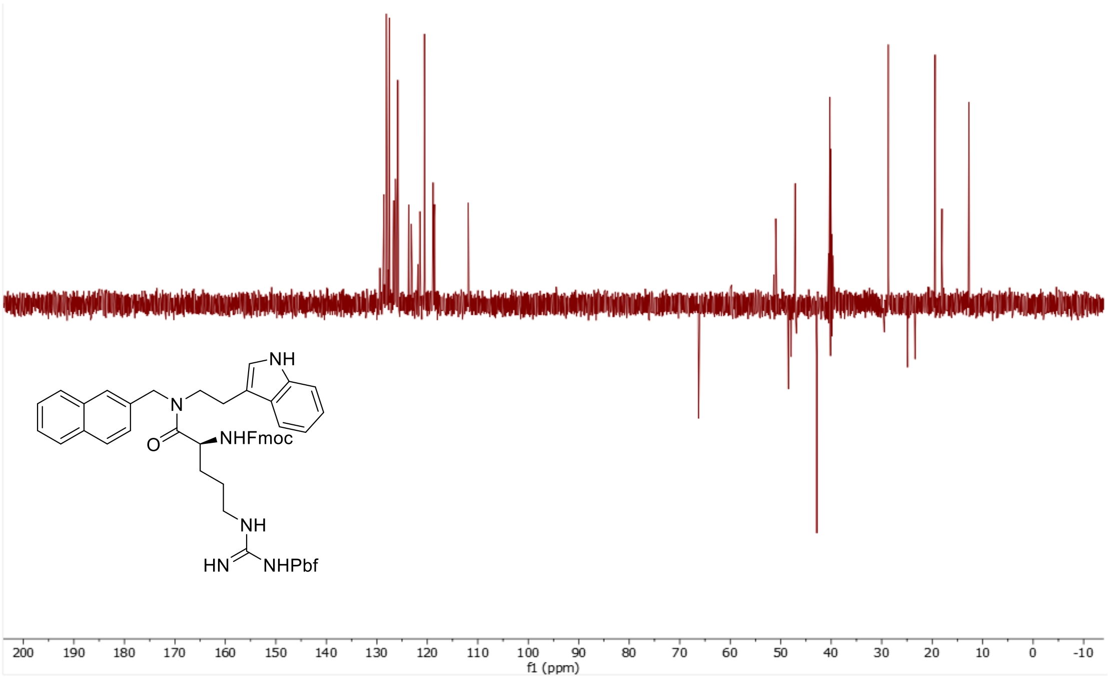

*52. Compound **21**- HSQC*

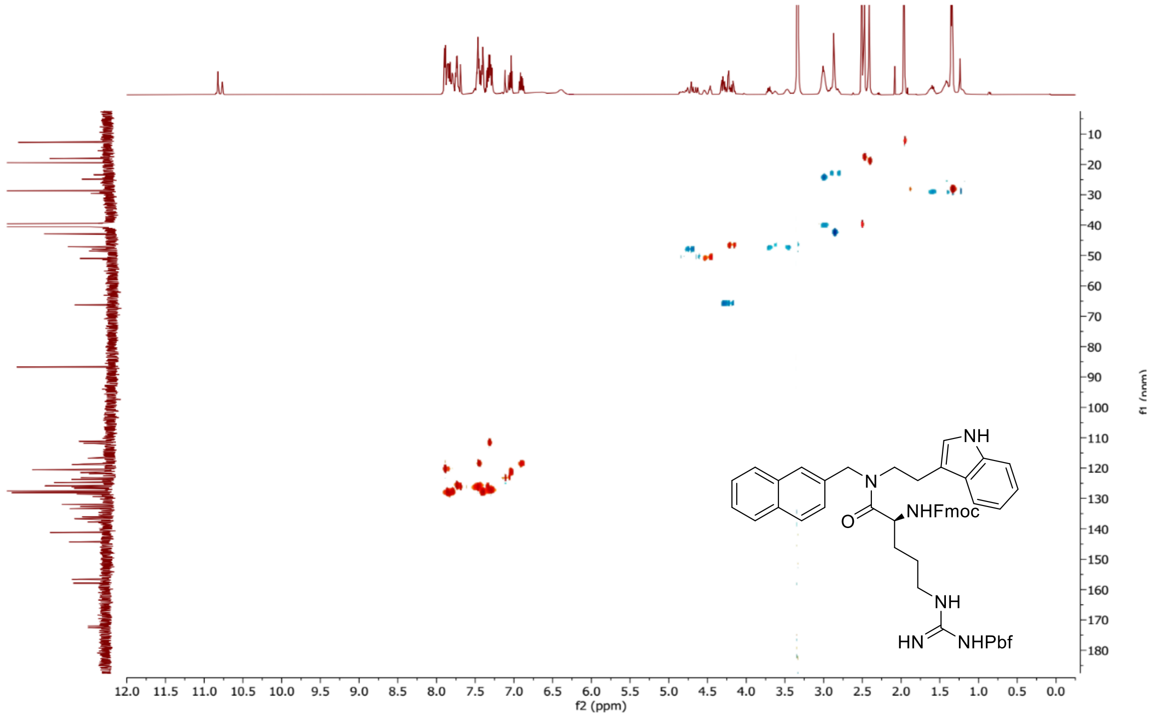

8- ^{1}H, and ^{13}CNMR of mono-amine naphthyl-indole and naphthyl-phenyl based peptoids

*53. Compound **12a** -^{1}HNMR*

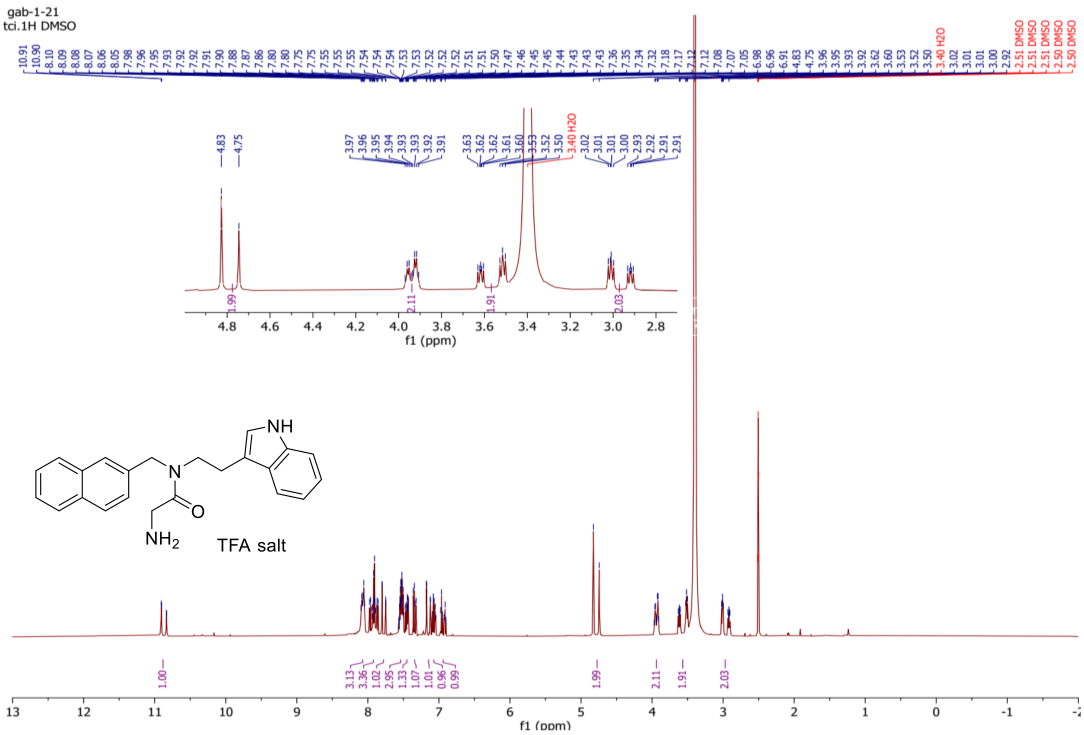

*54. Compound **12a** -^{13}CNMR*

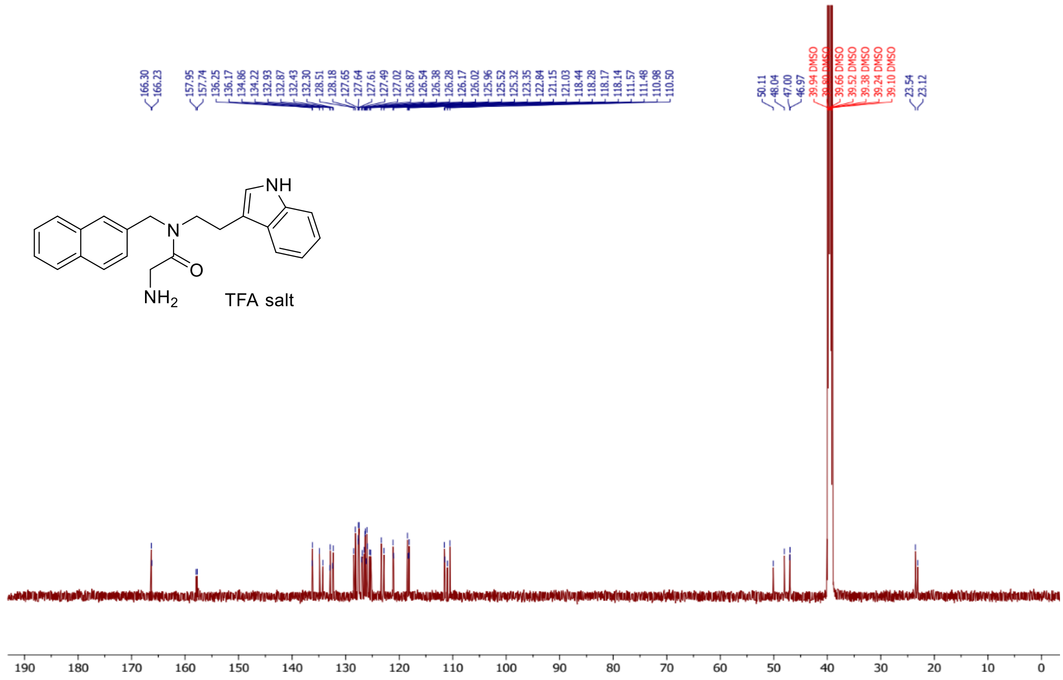

*55. Compound **12a** -^{13}CNMR dept 135*

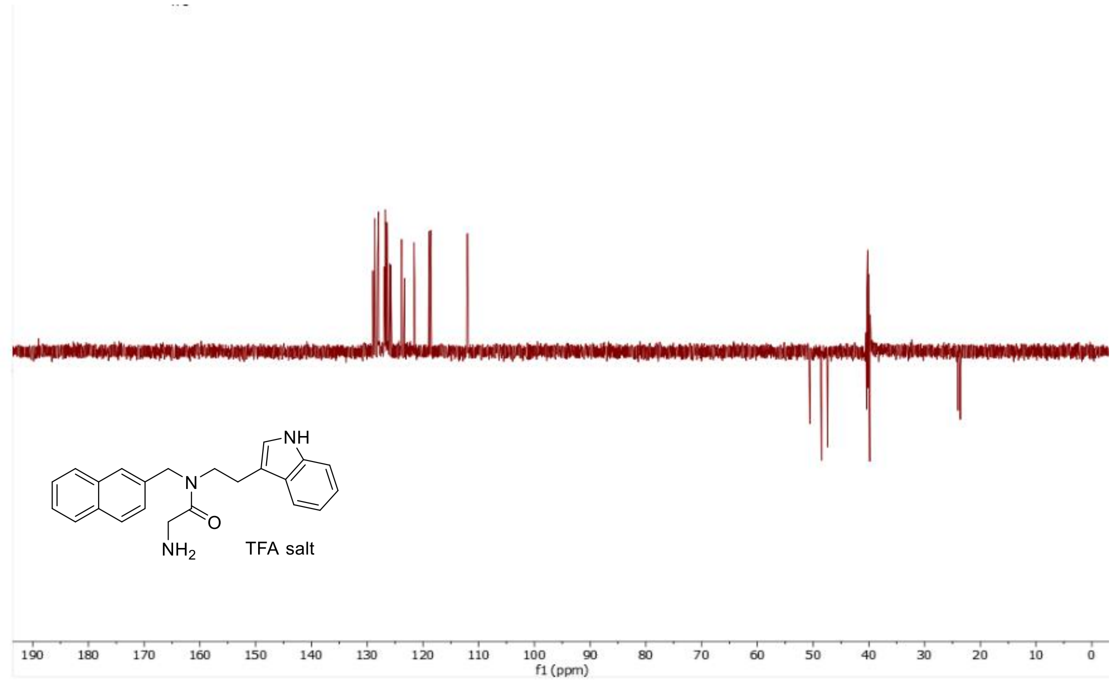

*56. Compound **12a** -HSQC*

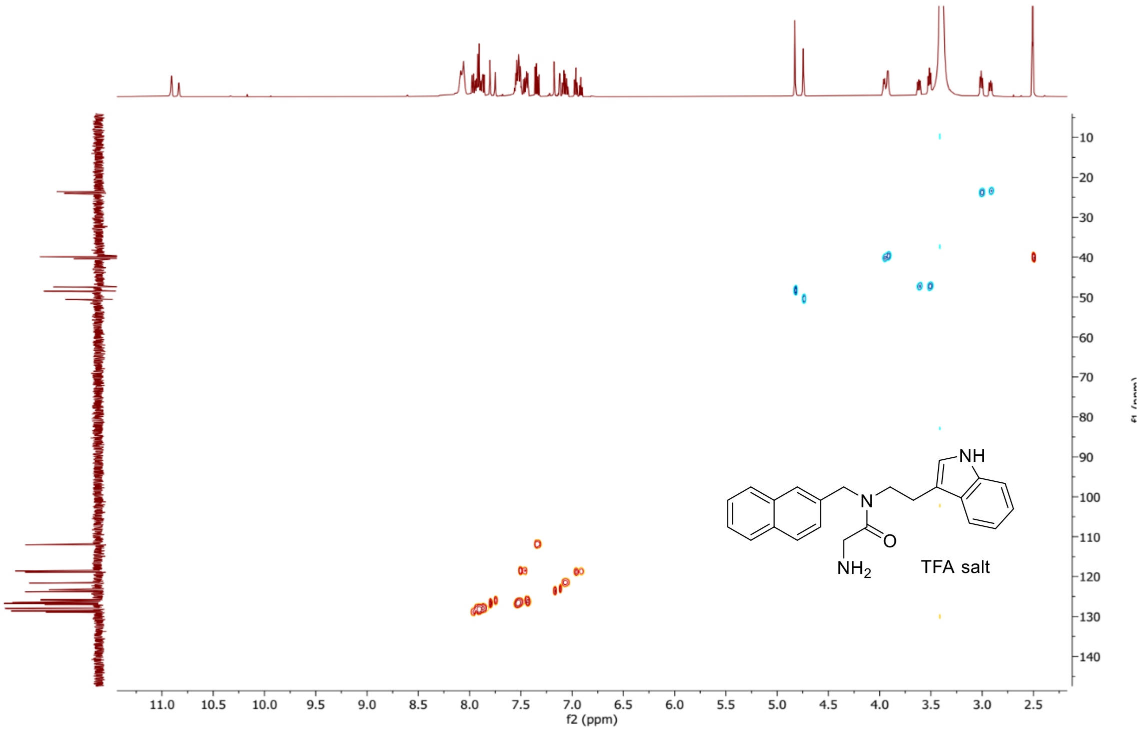

*57. Compound **12b** -^{1}HNMR*

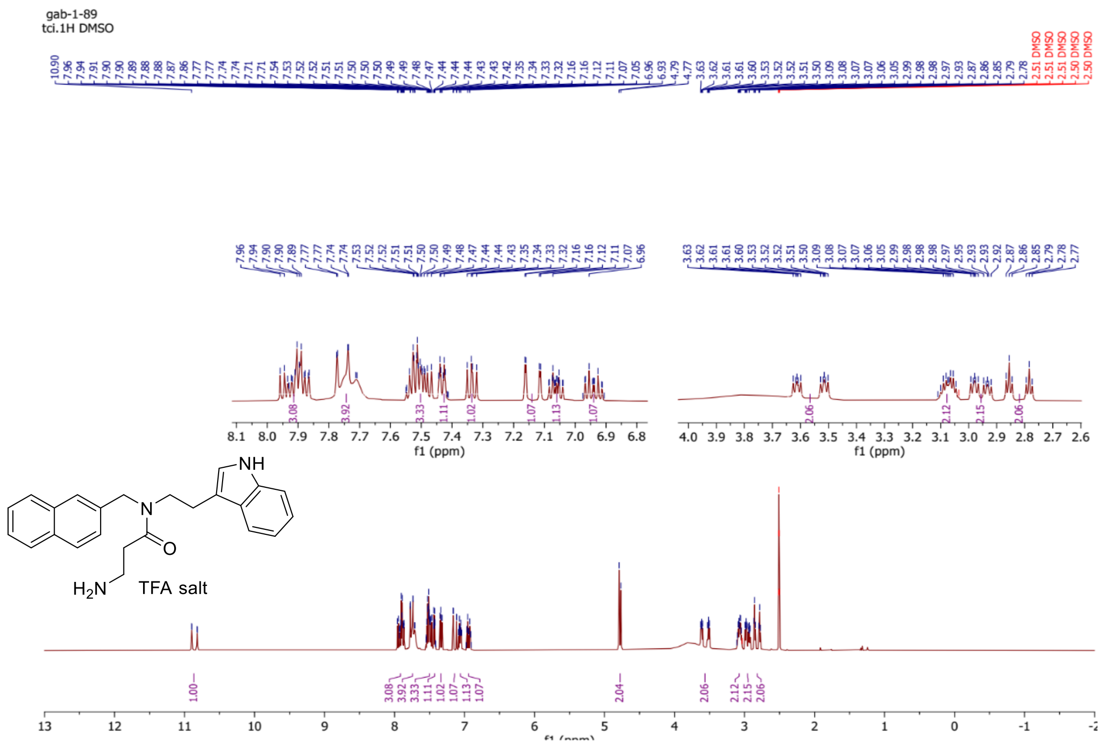

*58. Compound **12b** -^{13}CNMR*

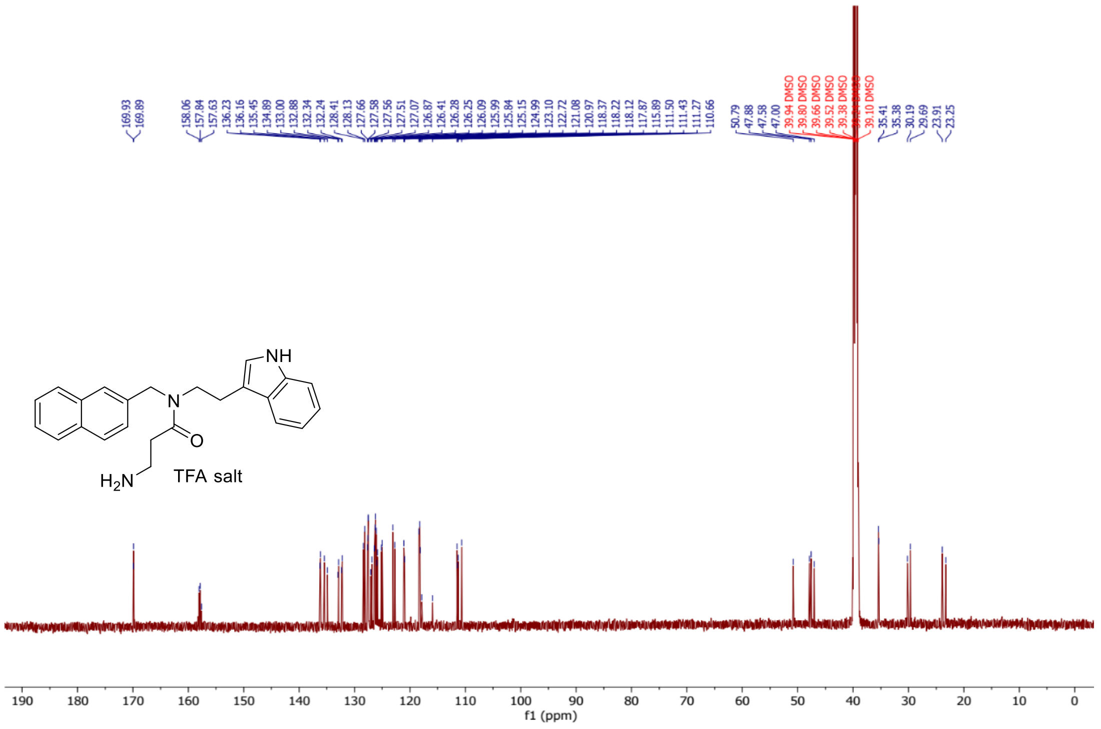

*59. Compound **12b**-^{13}CNMR dept 135*

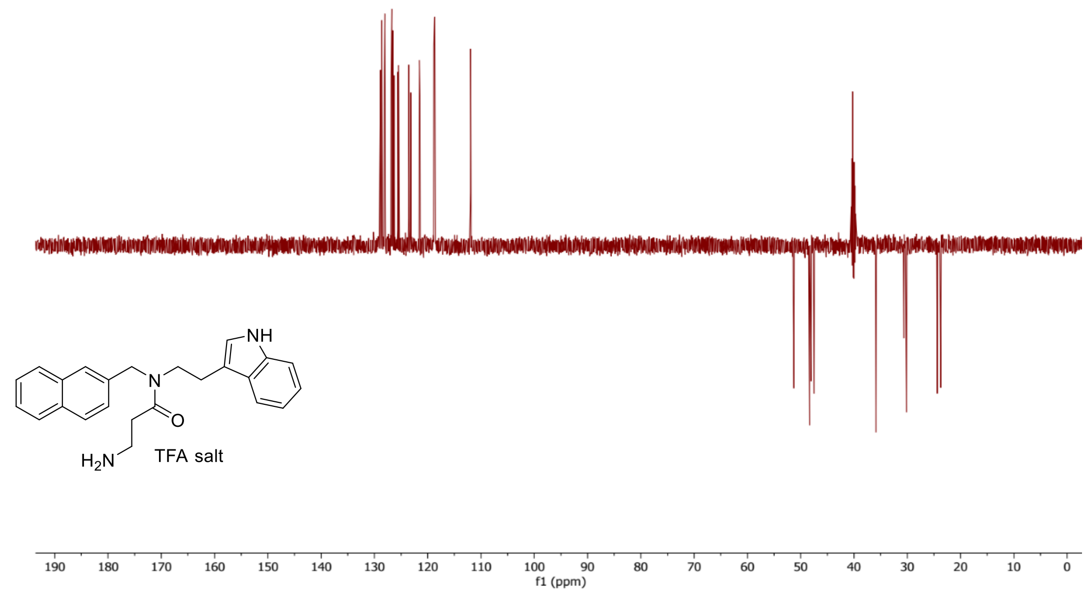

*60. Compound **12b**-HSQC*

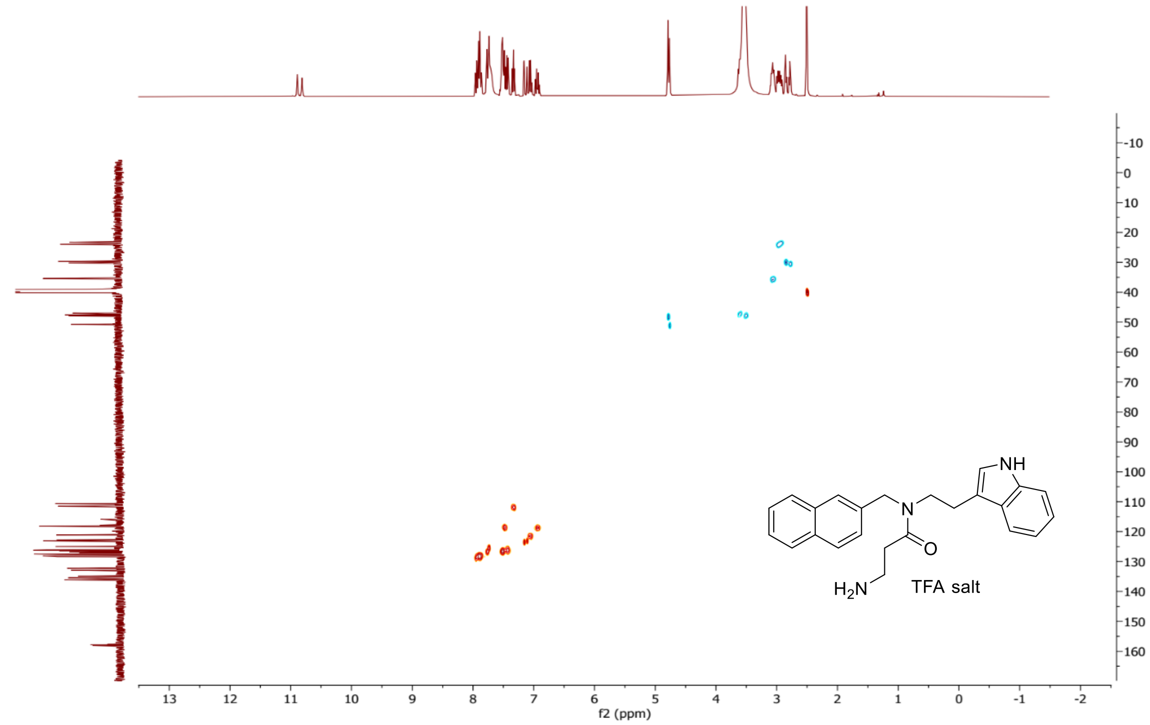

*61. Compound **12c**- ^{1}HNMR*

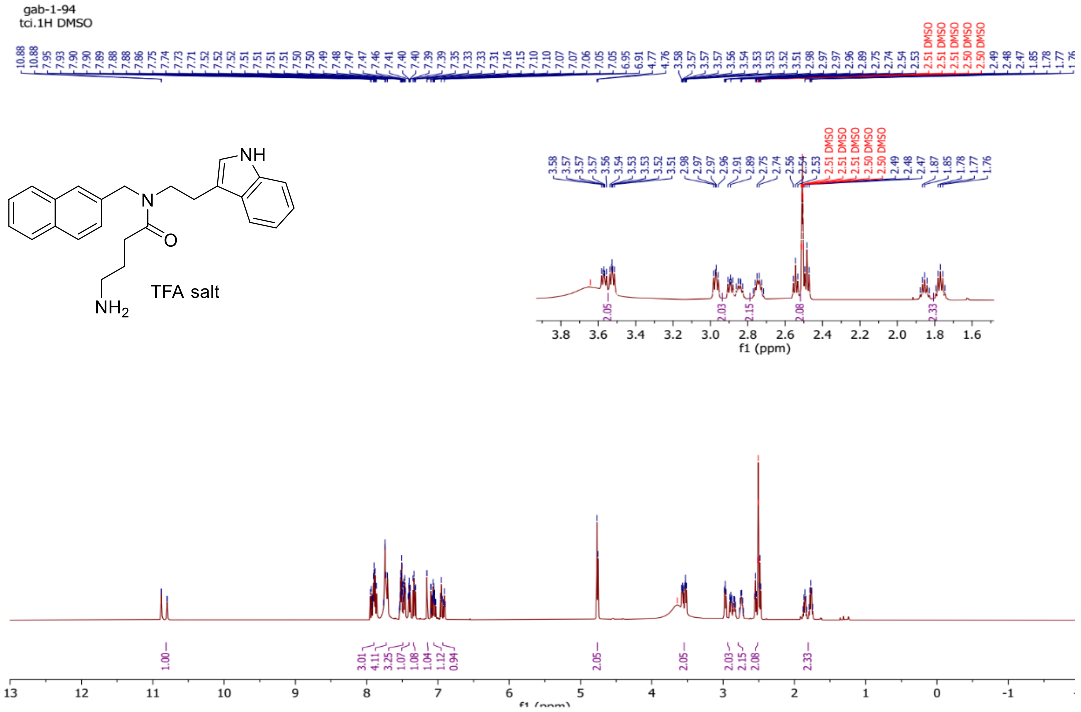

*62. Compound **12c**- ^{13}CNMR*

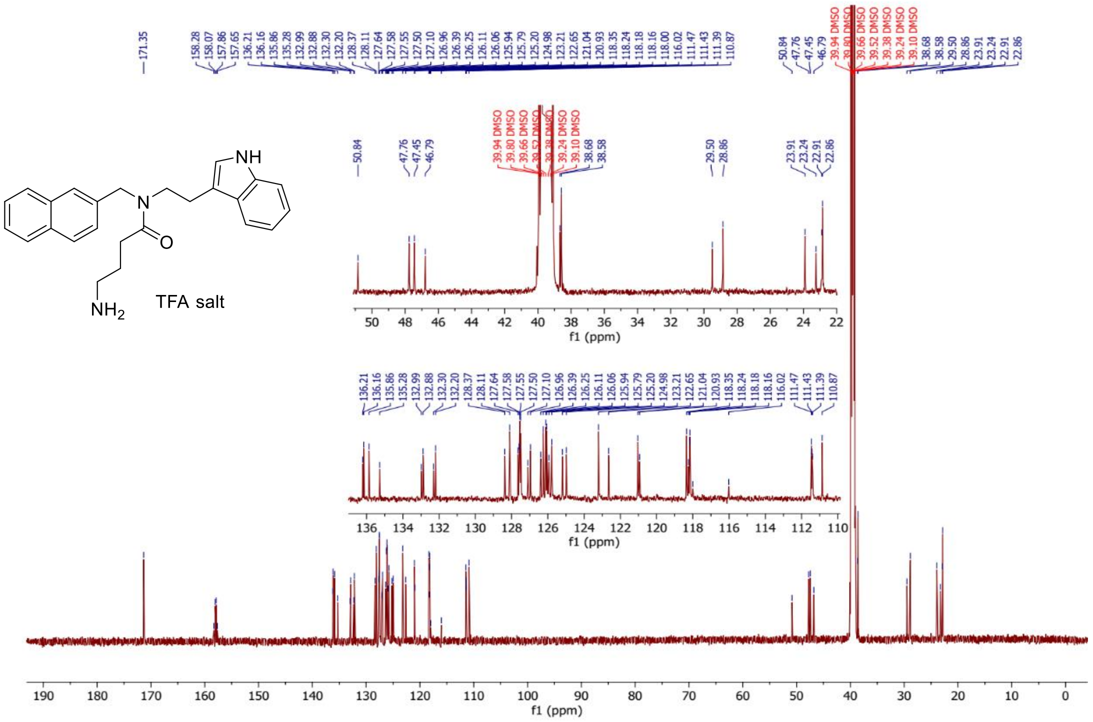

*63. Compound **12c**- ^{13}CNMR dept 135*

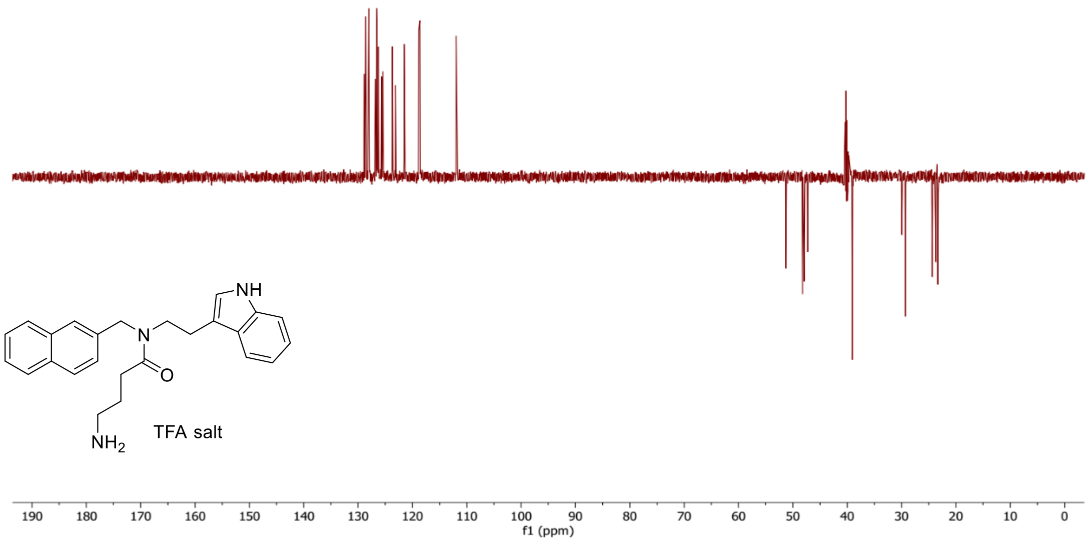

*64. Compound **12c**- HSQC*

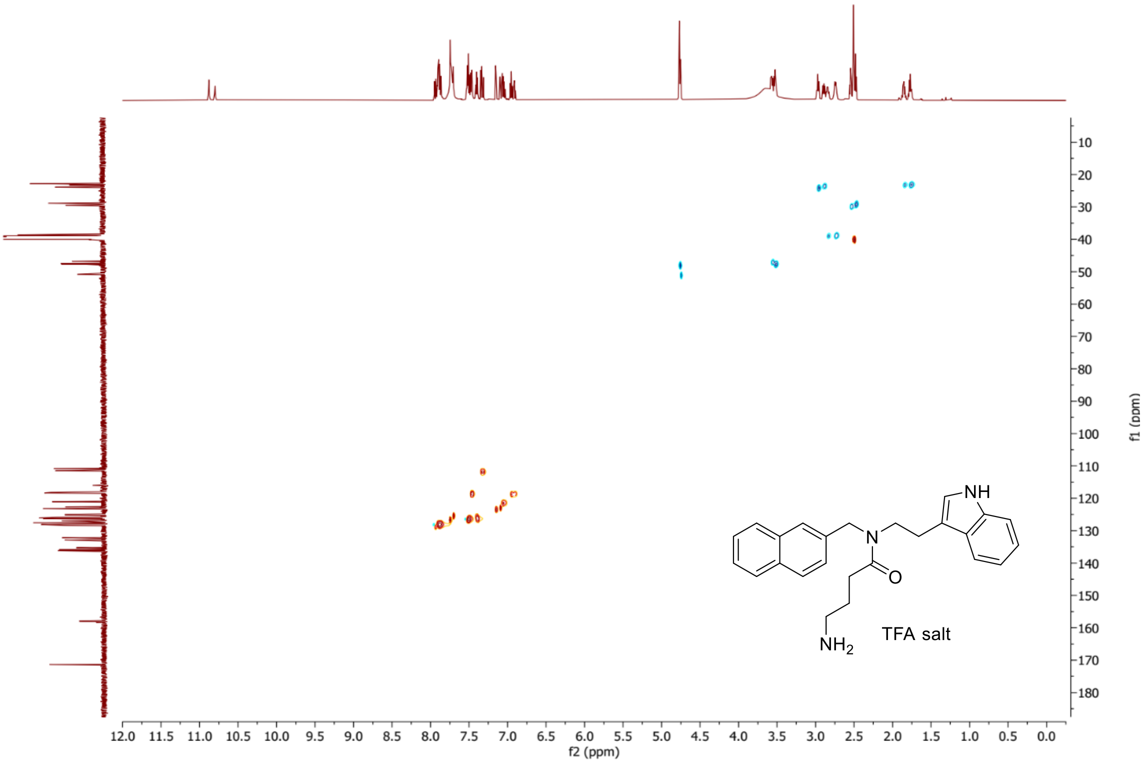

*65. Compound **12d**- ^{1}HNMR*

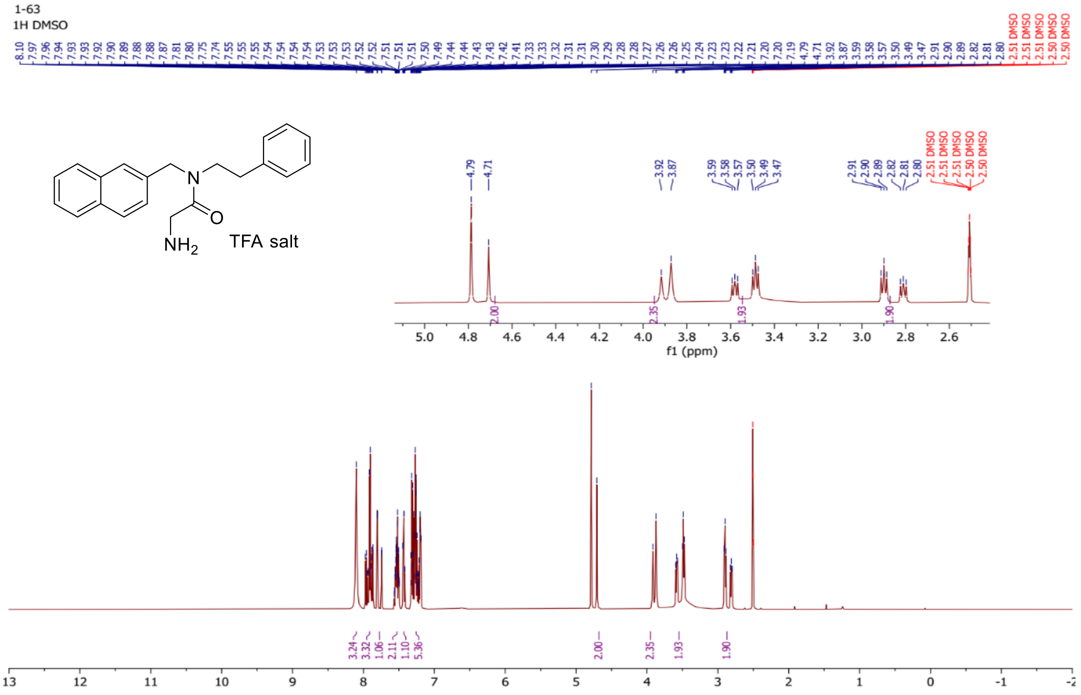

*66. Compound **12d**- ^{13}CNMR*

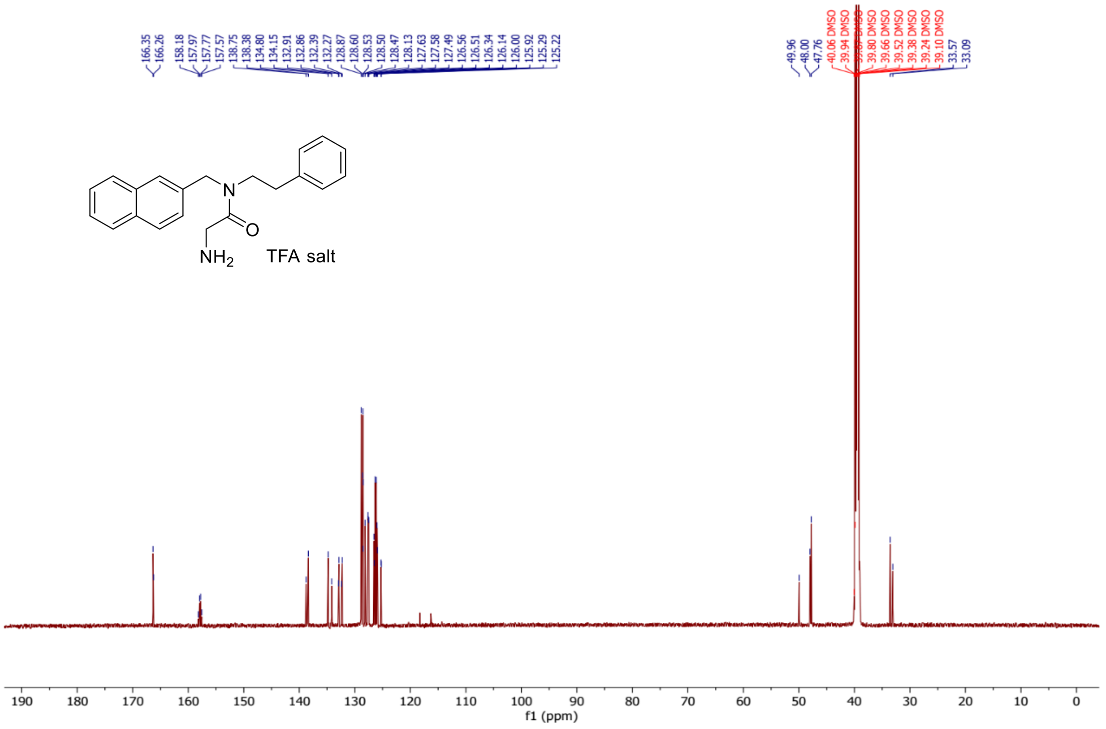

*67. Compound **12d**- ^{13}CNMR dept 135*

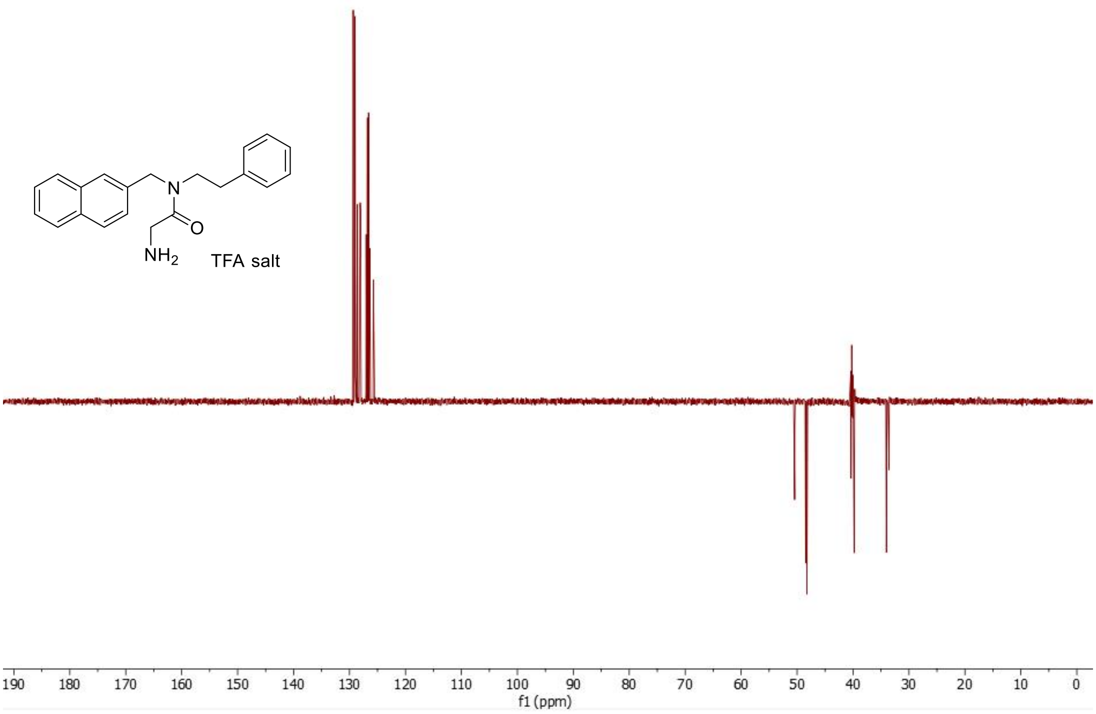

*68. Compound **12d**- HSQC*

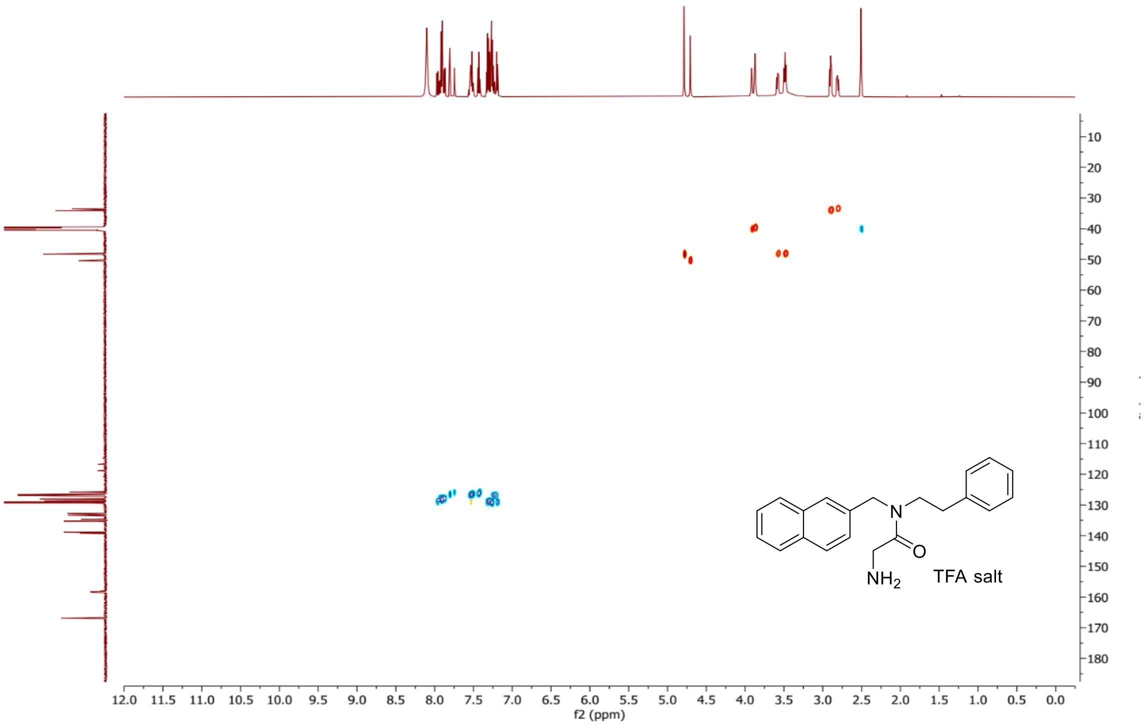

*69. Compound **12e**- ^{1}HNMR*

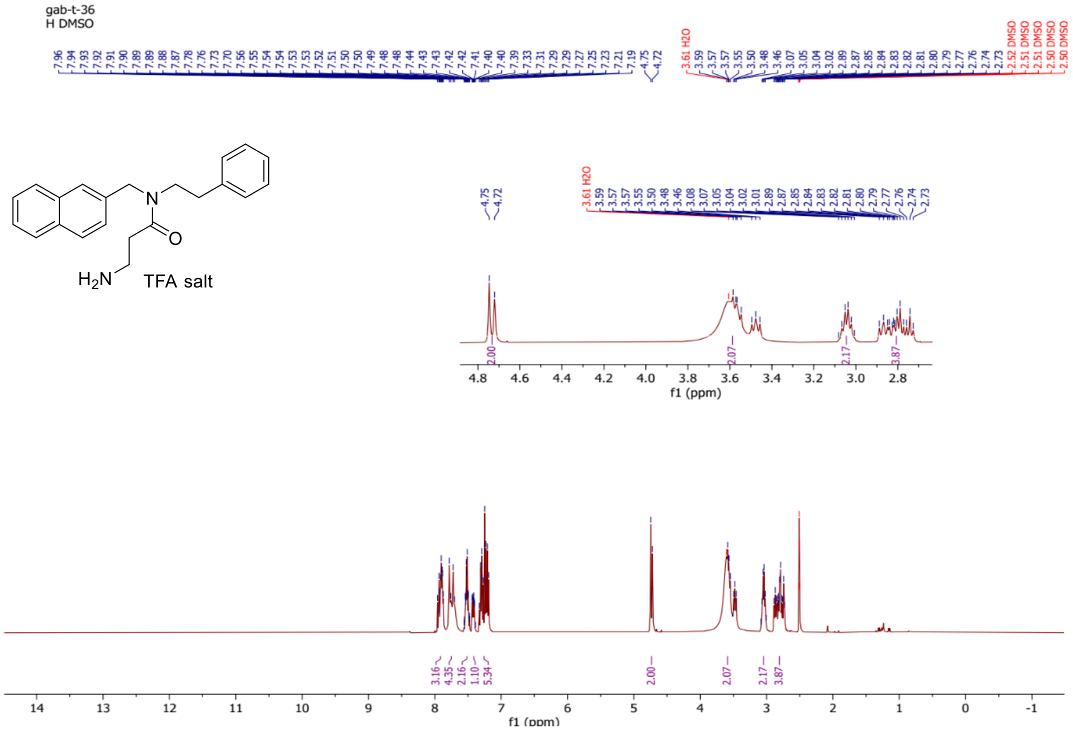

*70. Compound **12e**- ^{13}CNMR*

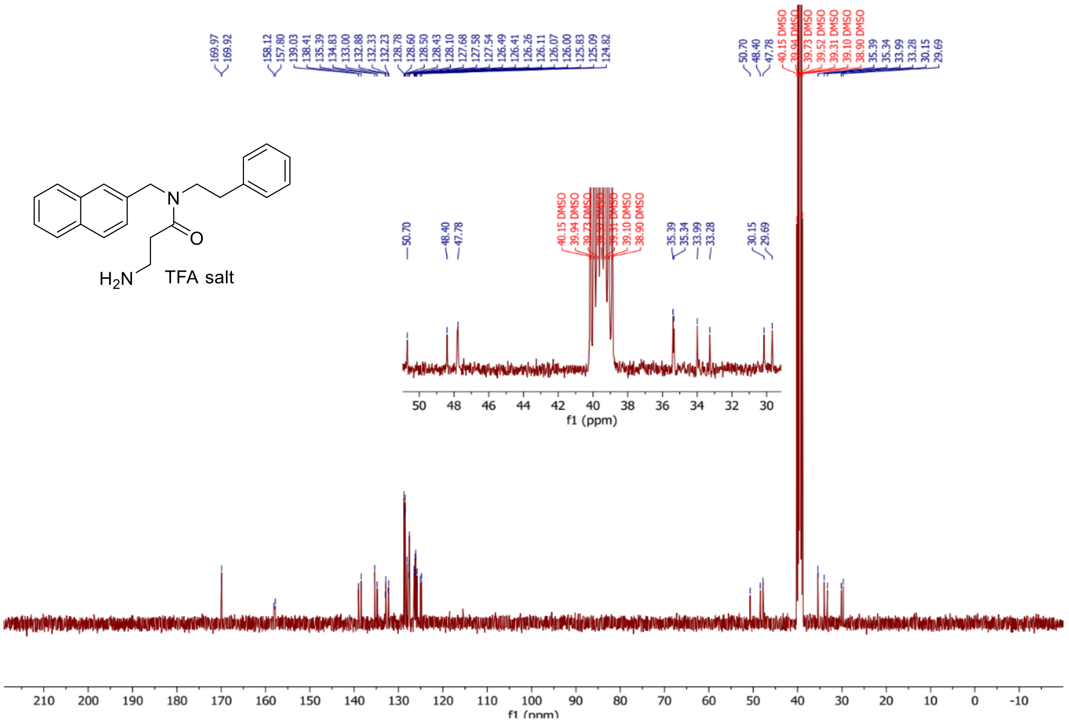

*71. Compound **12e**- ^{13}CNMR dept 135*

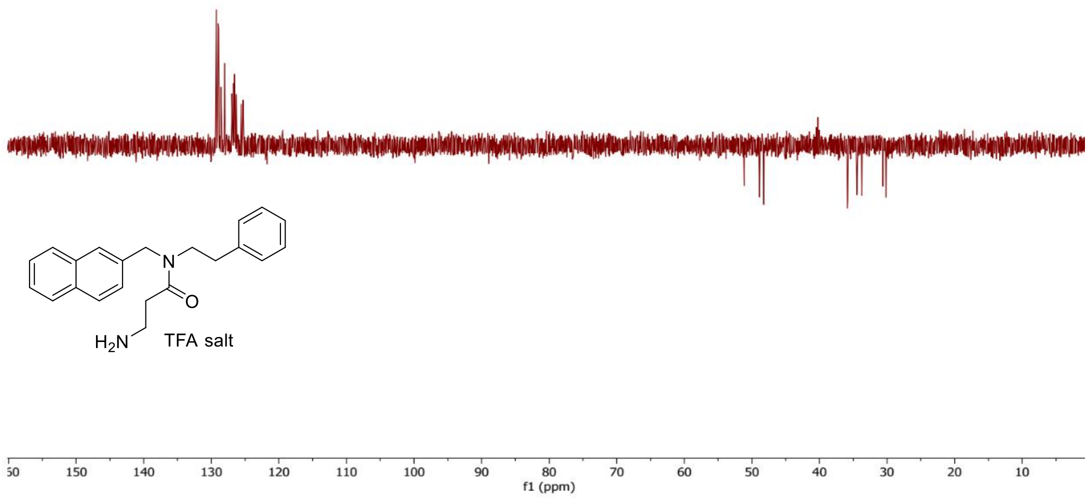

*72. Compound **12e**- HSQC*

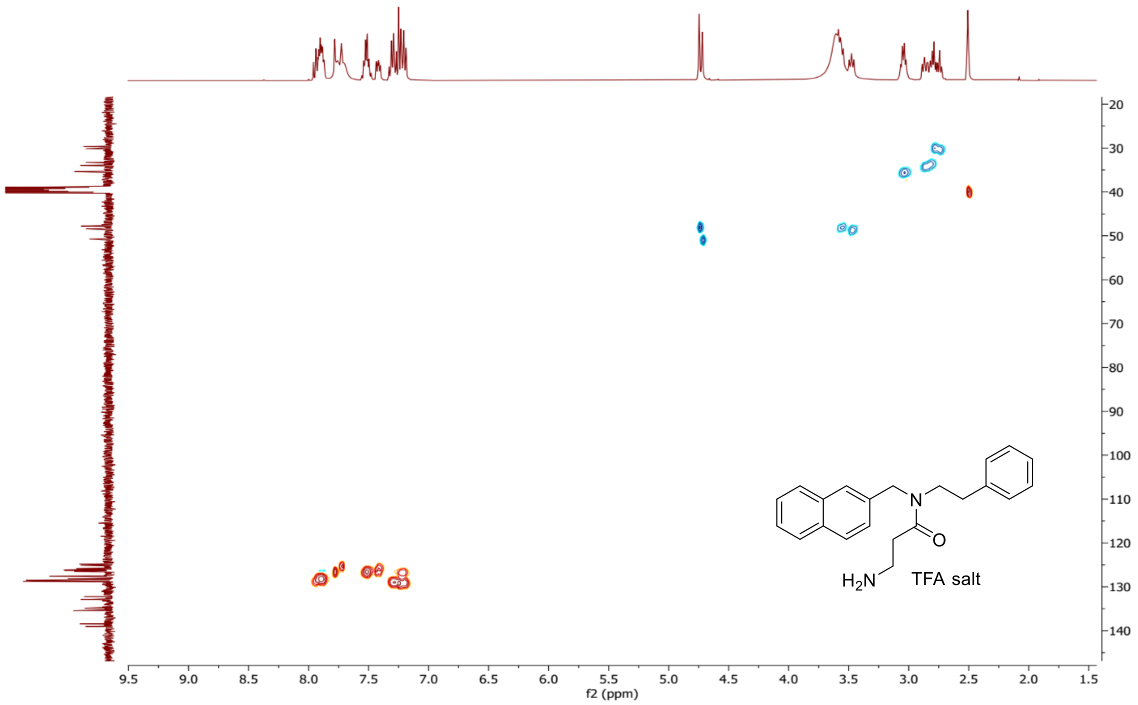

*73. Compound **12f**- ^{1}HNMR*

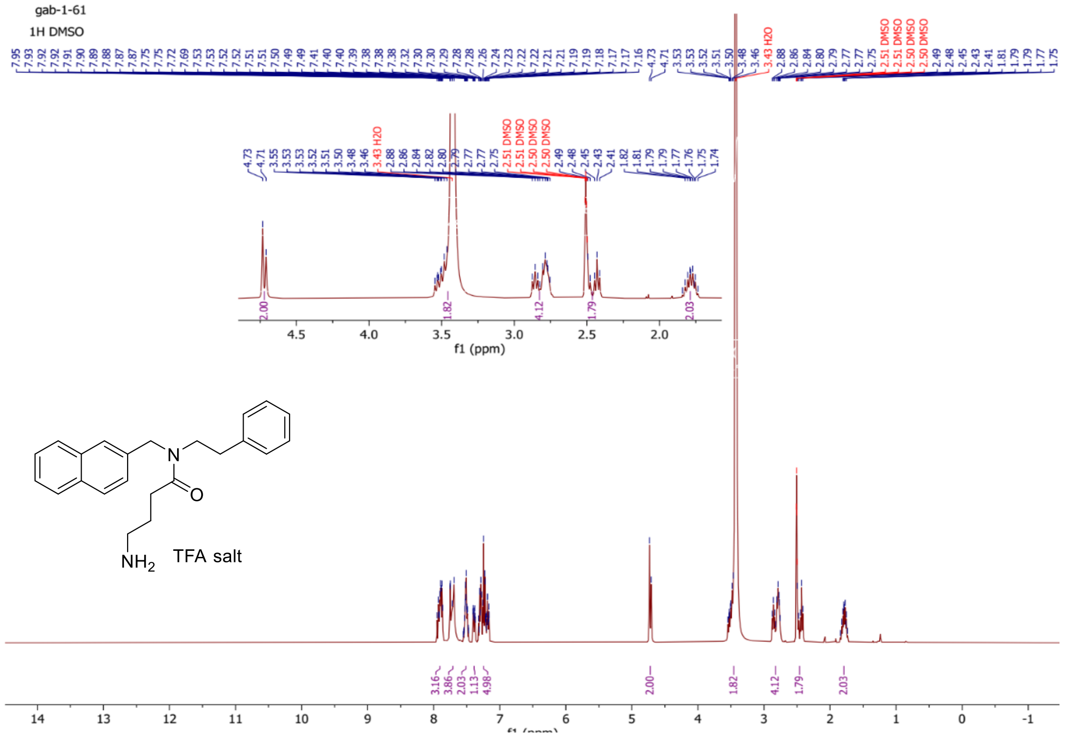

*74. Compound **12f**- ^{13}CNMR*

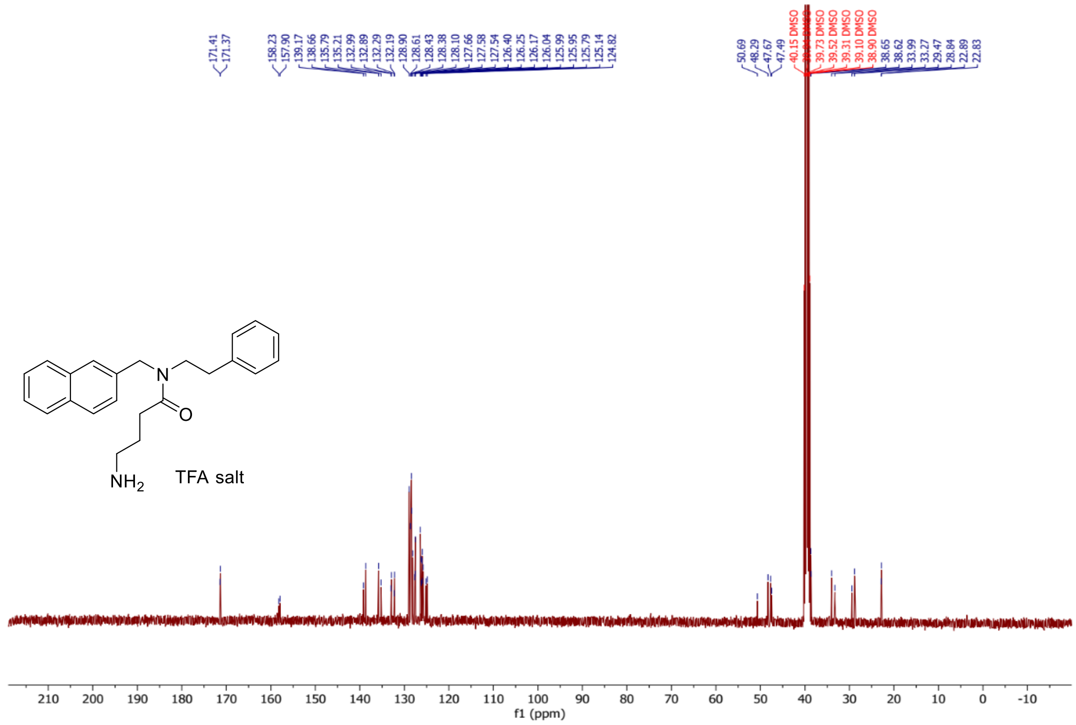

*75. Compound **12f**- ^{13}CNMR dept 135*

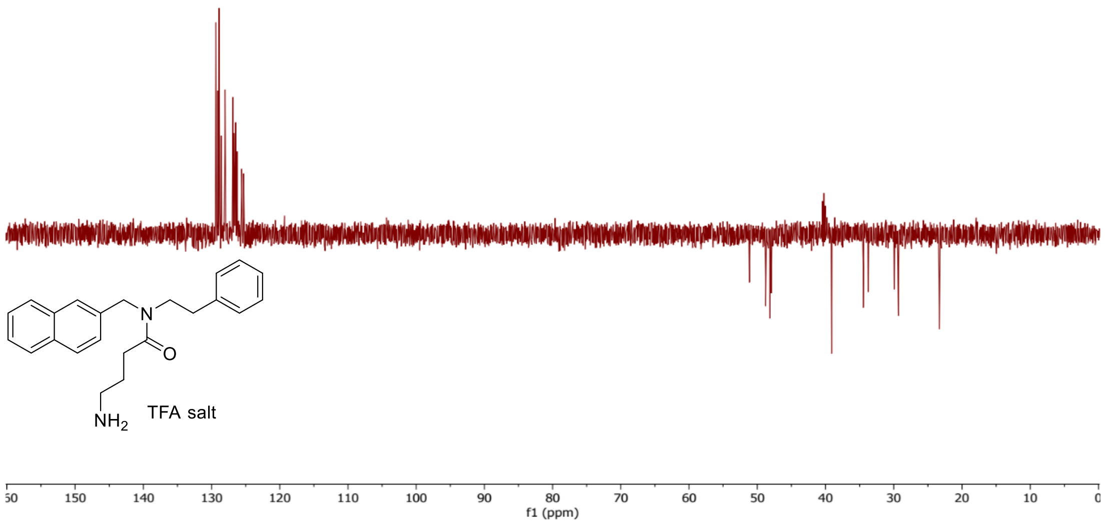

*76. Compound **12f**- HSQC*

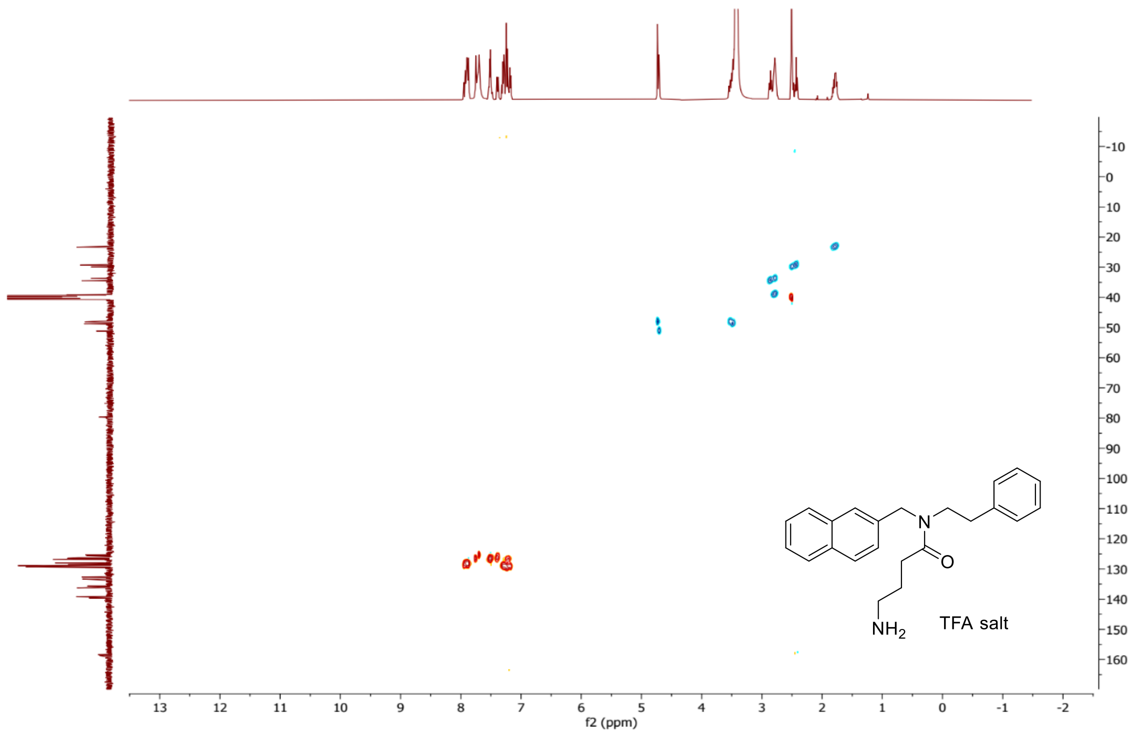

*77. Compound **26**- ^{1}HNMR*

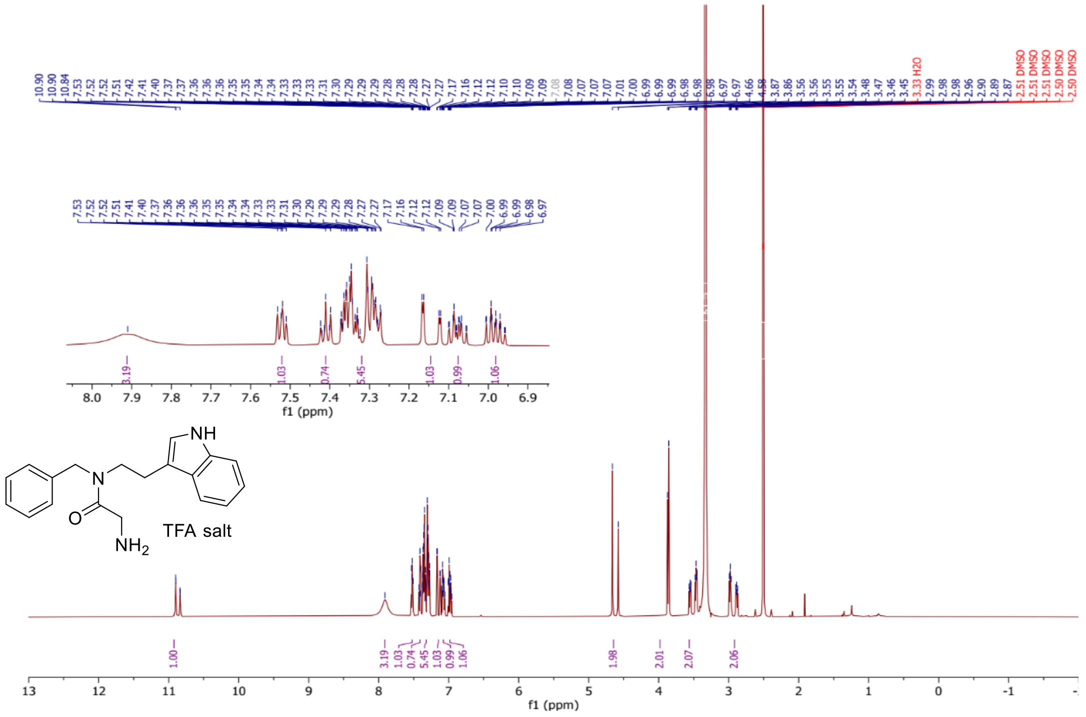

*78. Compound **26**- ^{13}CNMR*

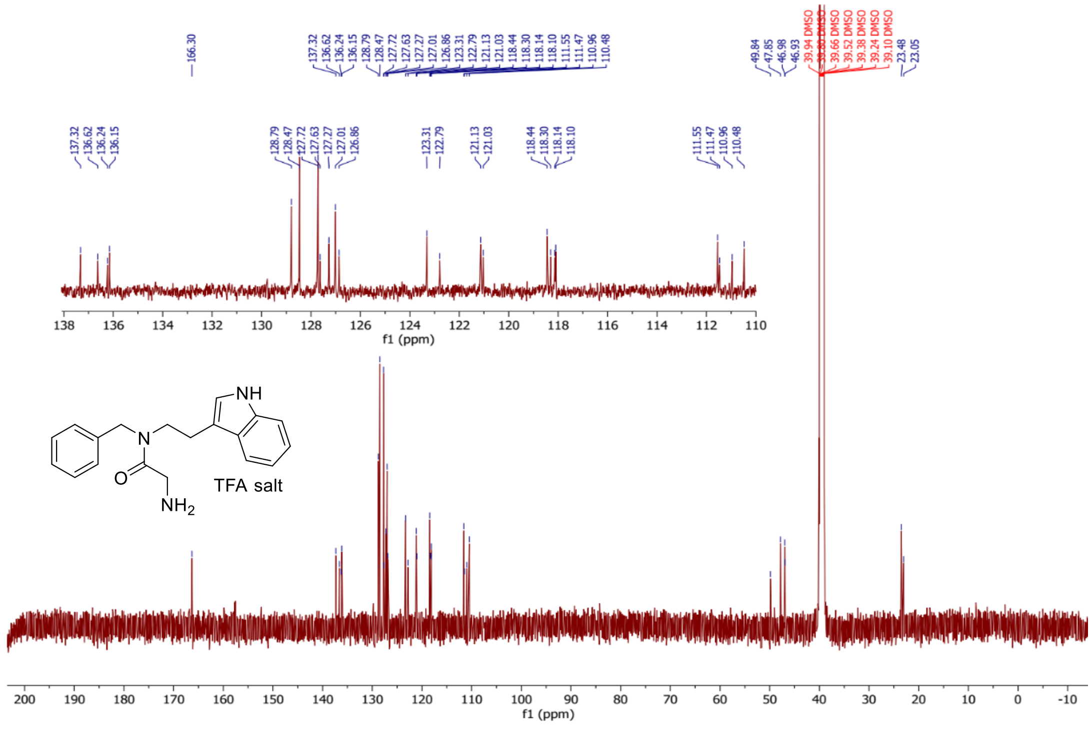

*79. Compound **26**- ^{13}CNMR dept 135*

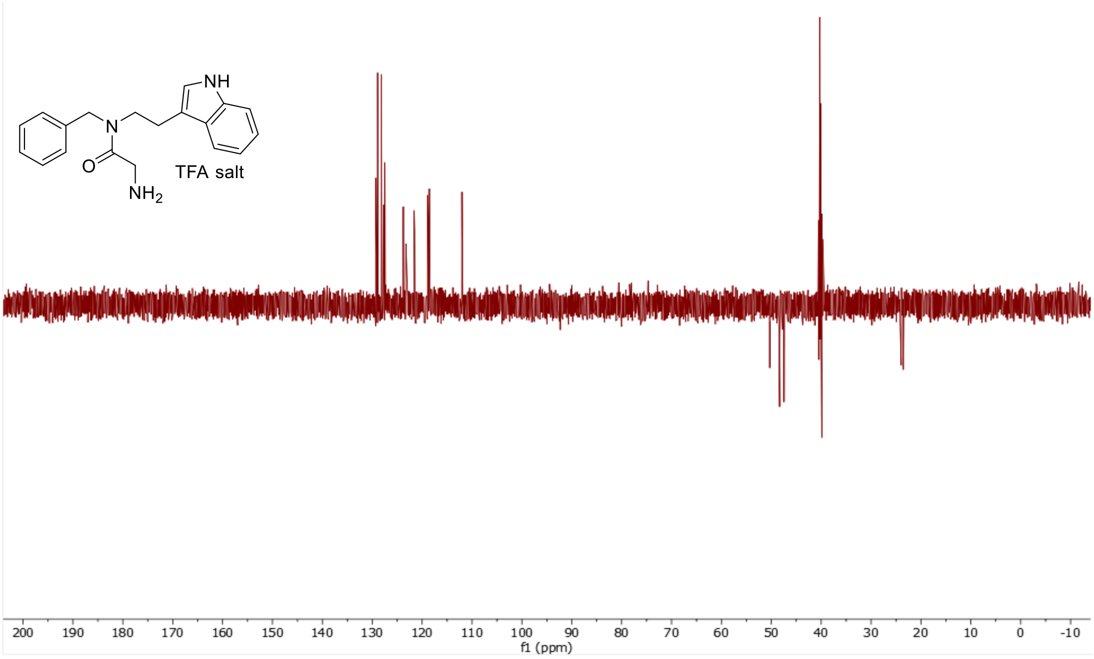

*80. Compound **26**- HSQC*

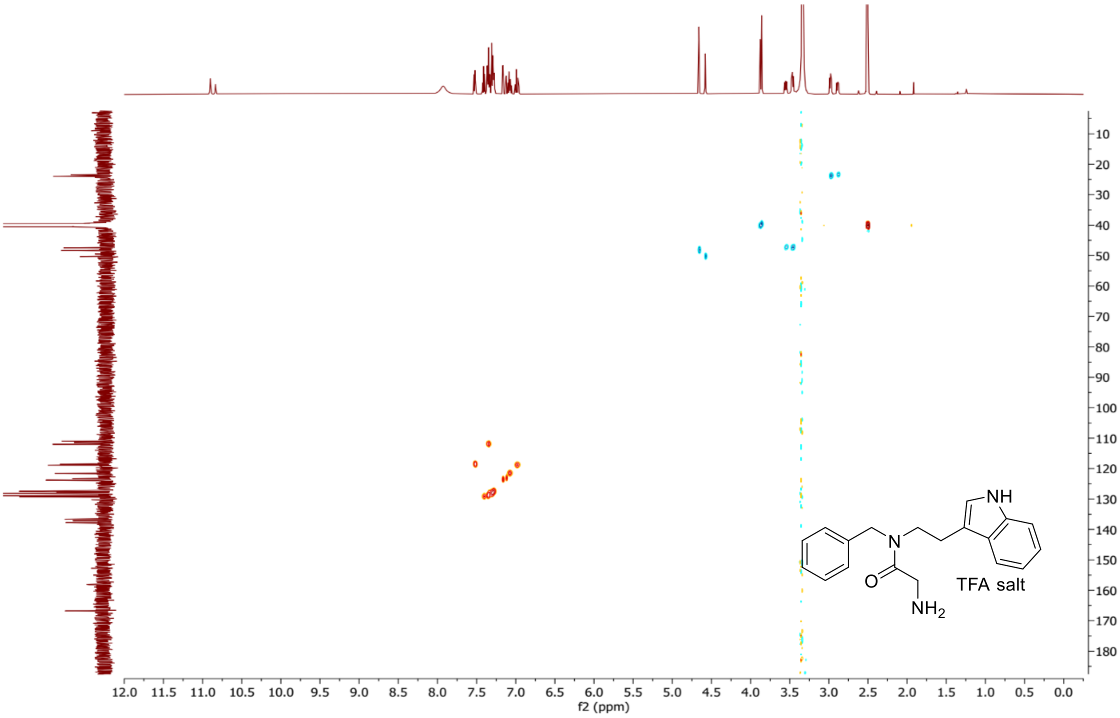

9- ^{1}H, and ^{13}CNMR of diamino-naphthyl- indole based peptoids.

*81. Compound **19b**- ^{1}HNMR*

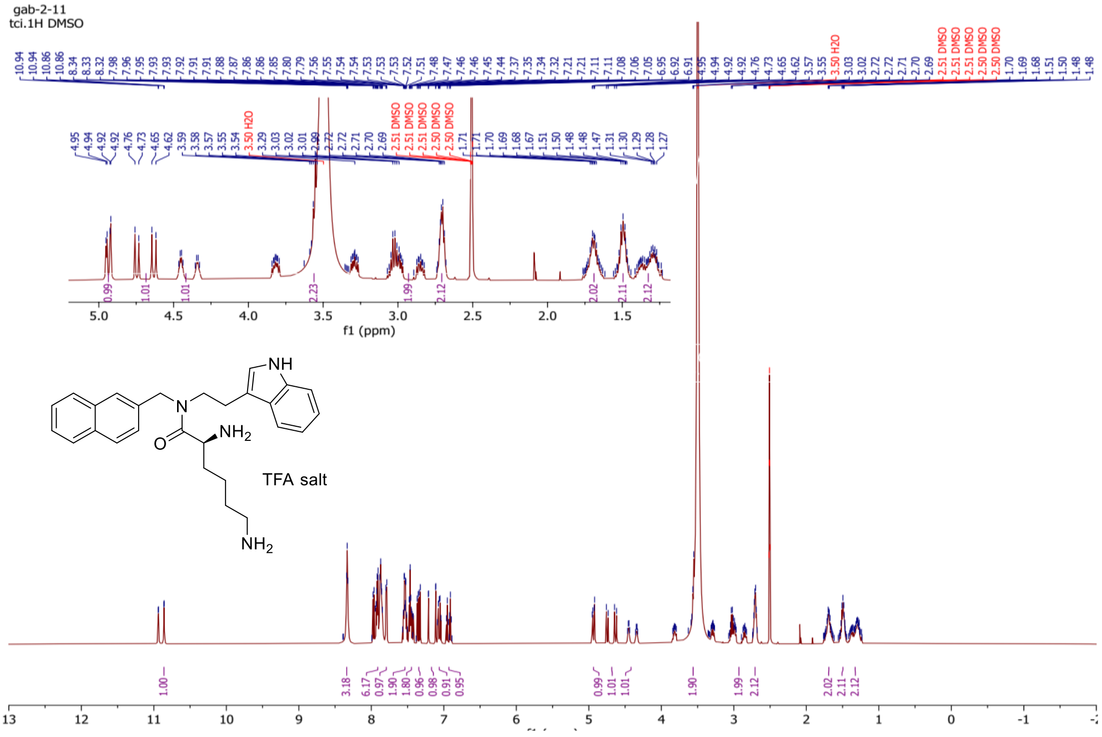

*82. Compound **19b**- ^{13}CNMR*

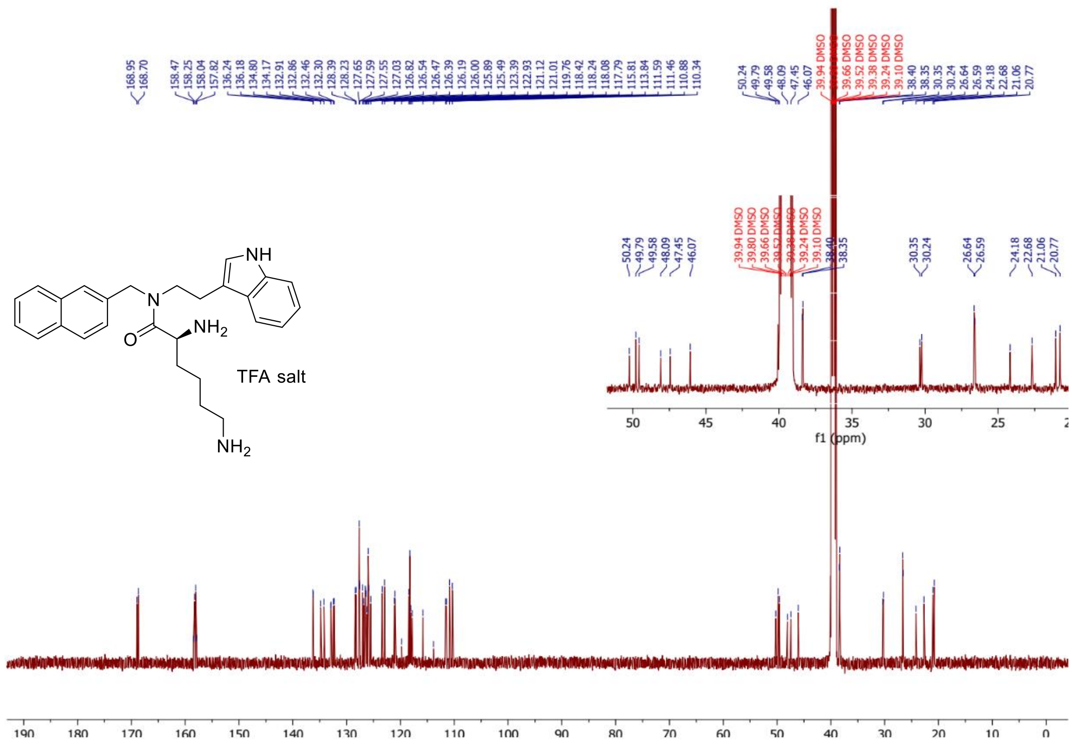

*83. Compound **19b**- ^{13}CNMR dept 135*

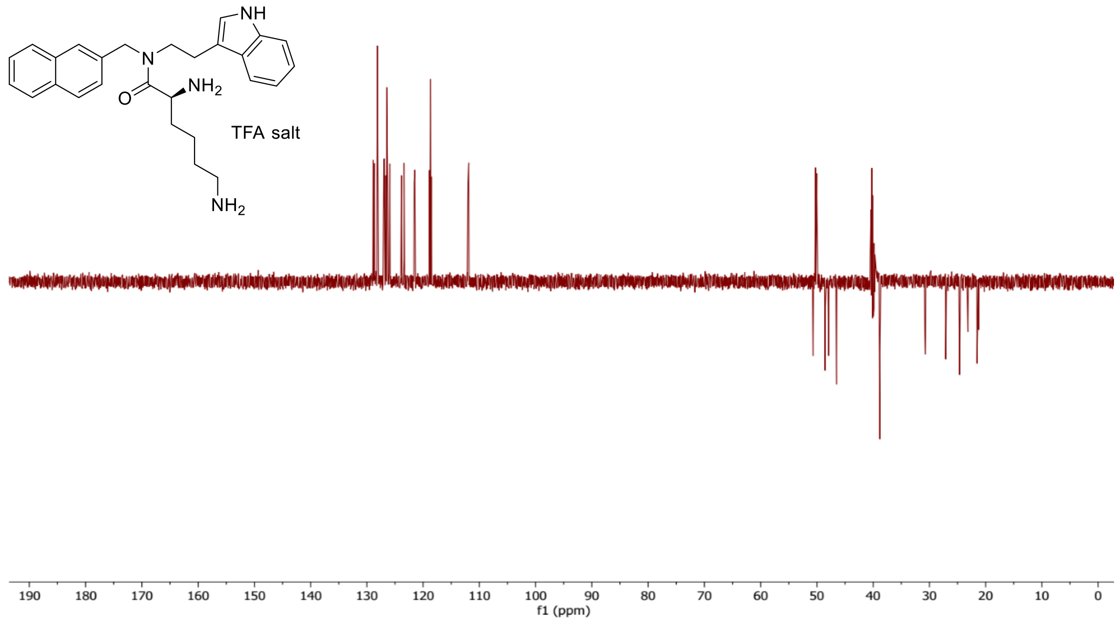

*84. Compound **19b**- HSQC*

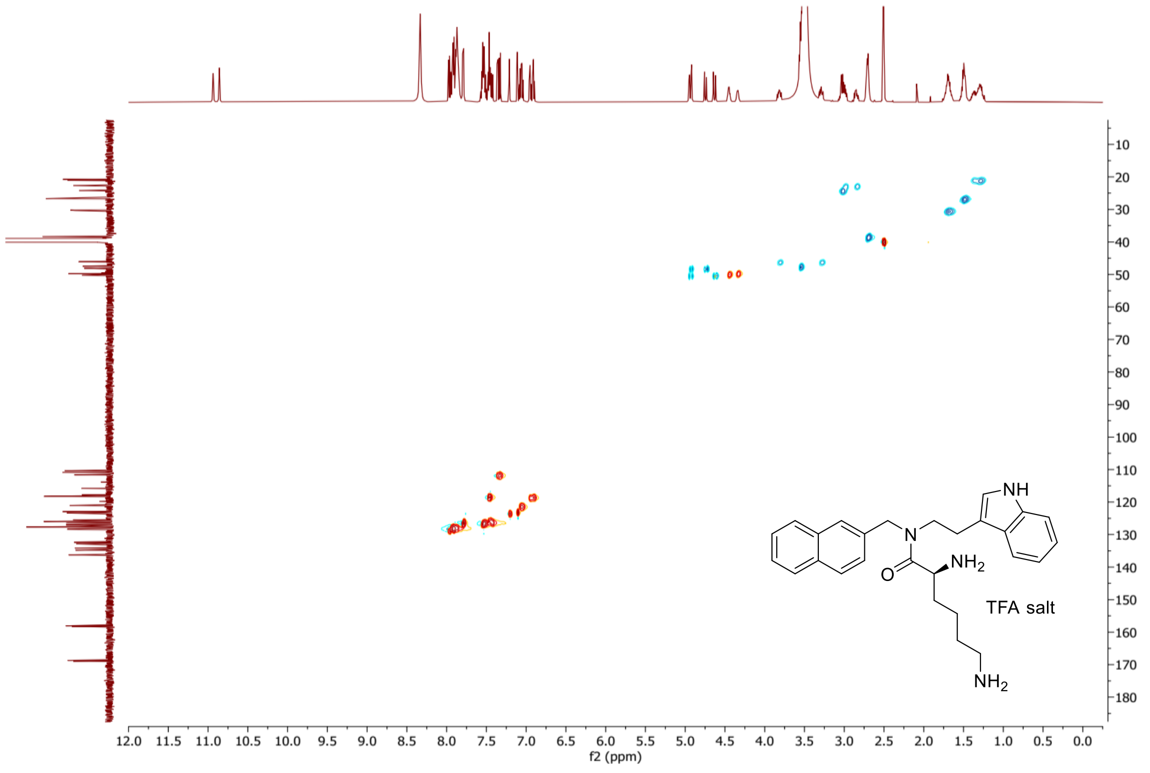

*85. Compound **19a**- ^{1}HNMR*

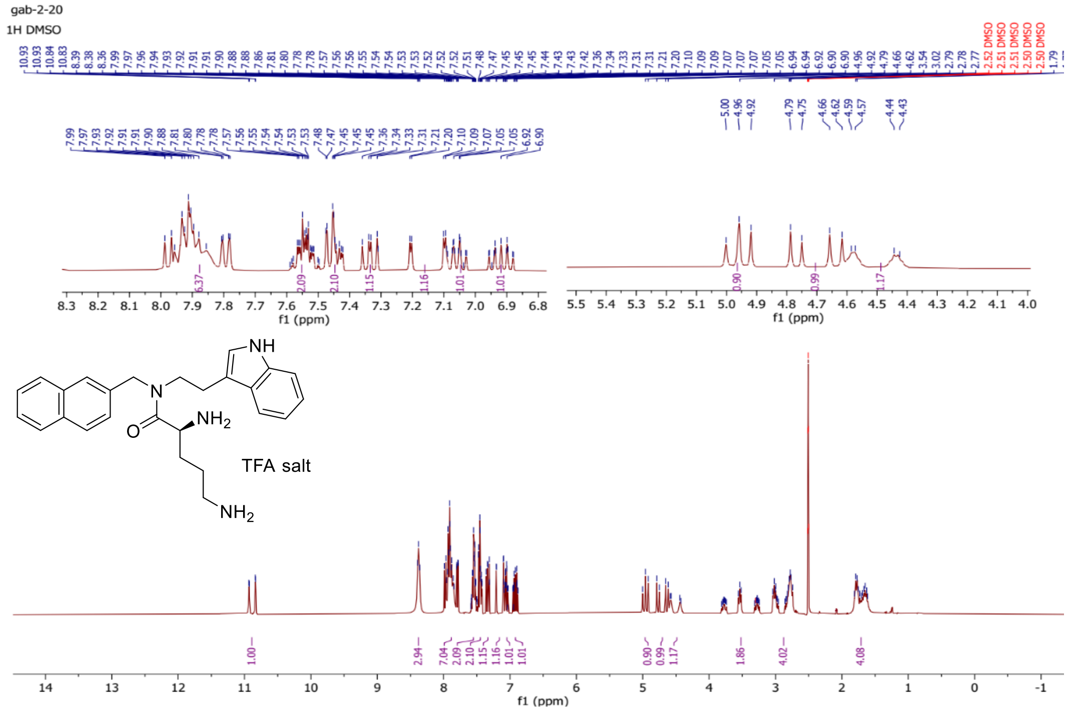

*86. Compound **19a**- ^{13}CNMR*

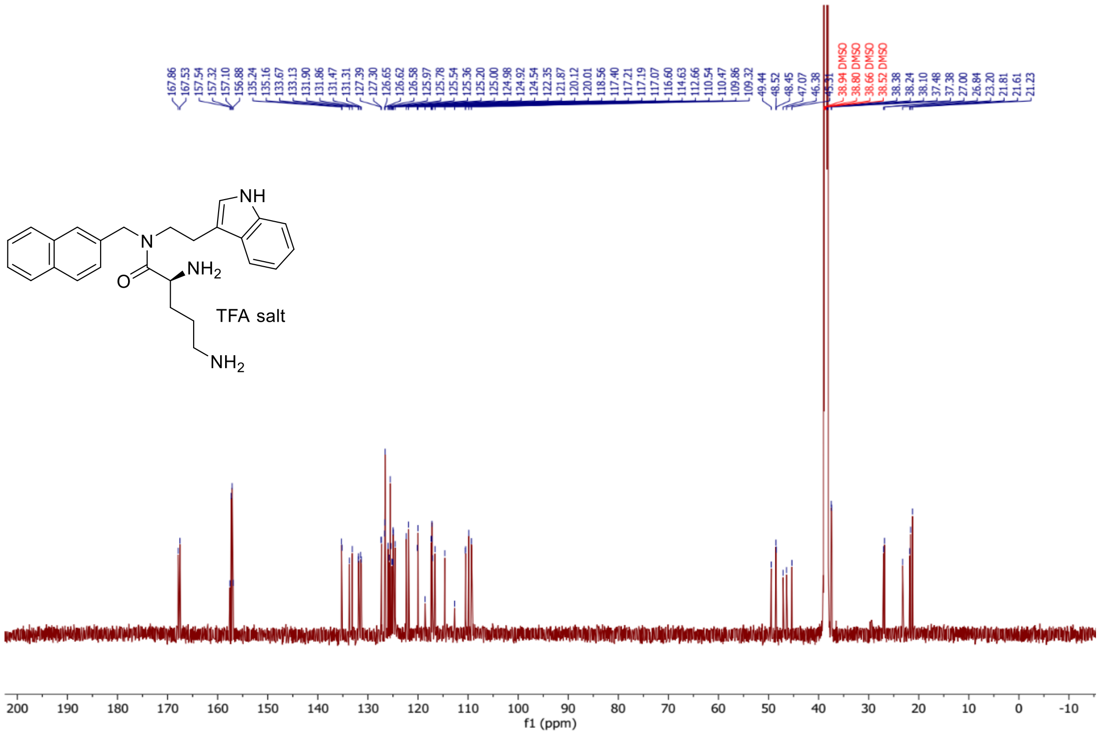

*87. Compound **19a**- ^{13}CNMR dept 135*

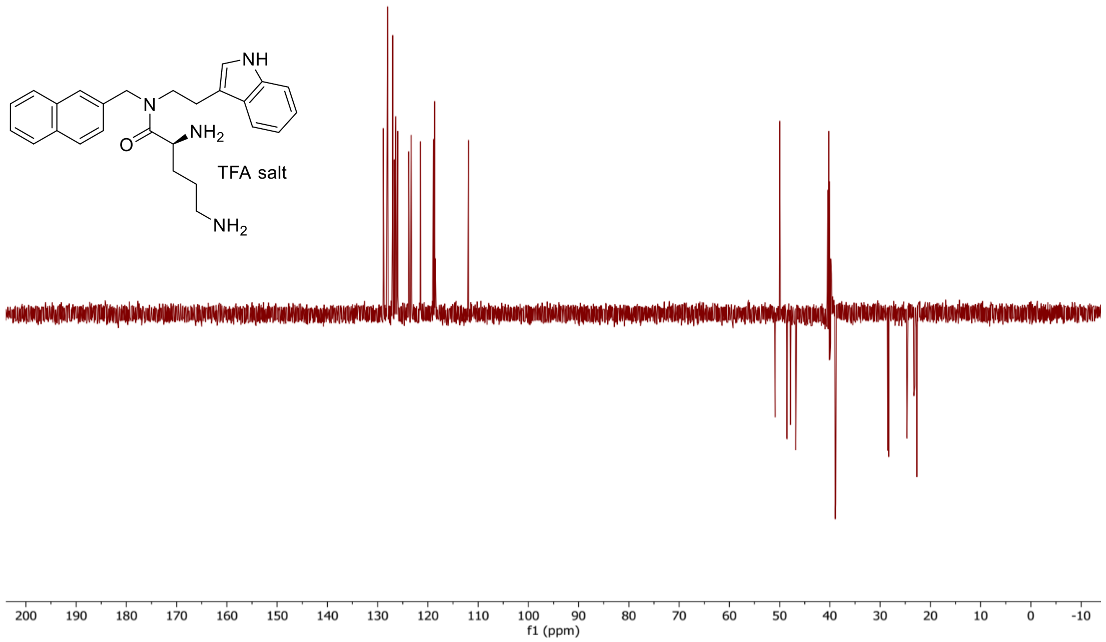

*88. Compound **19a**- HSQC*

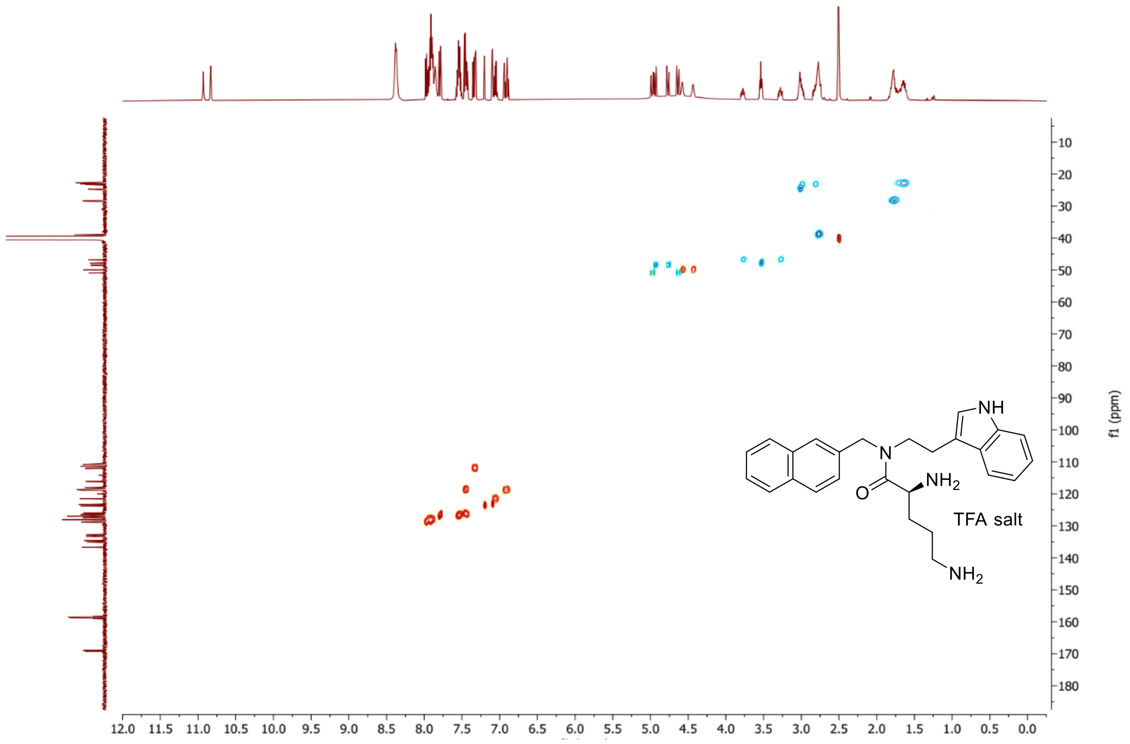

*89. Compound **22**- ^{1}HNMR*

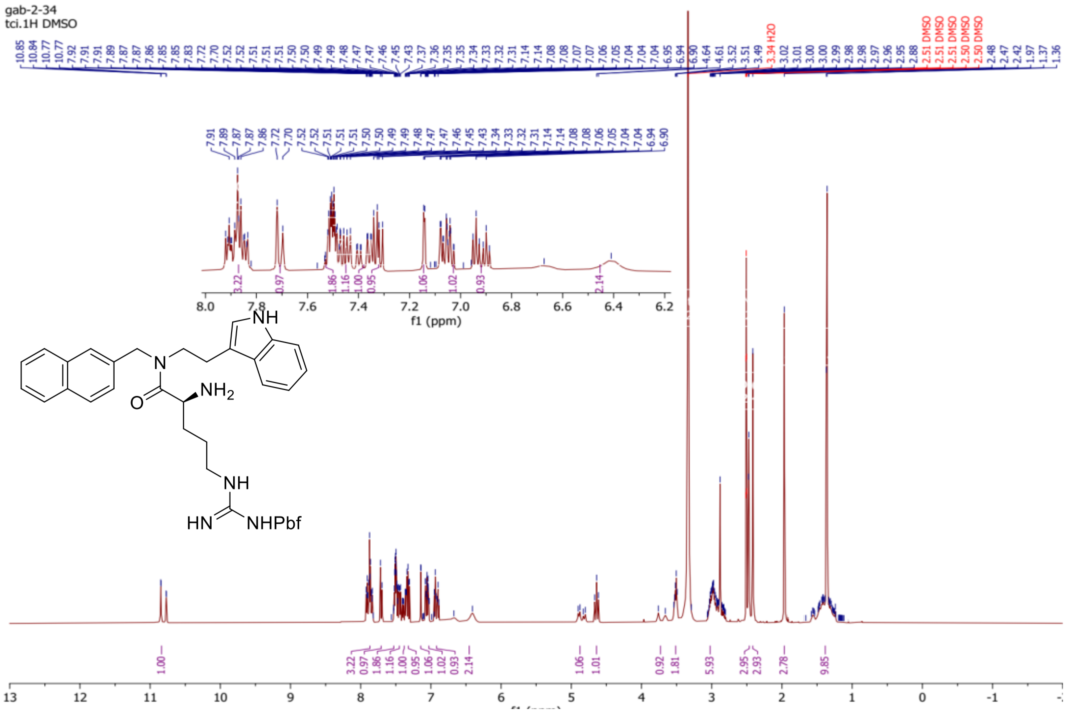

*90. Compound **22**- ^{13}CNMR*

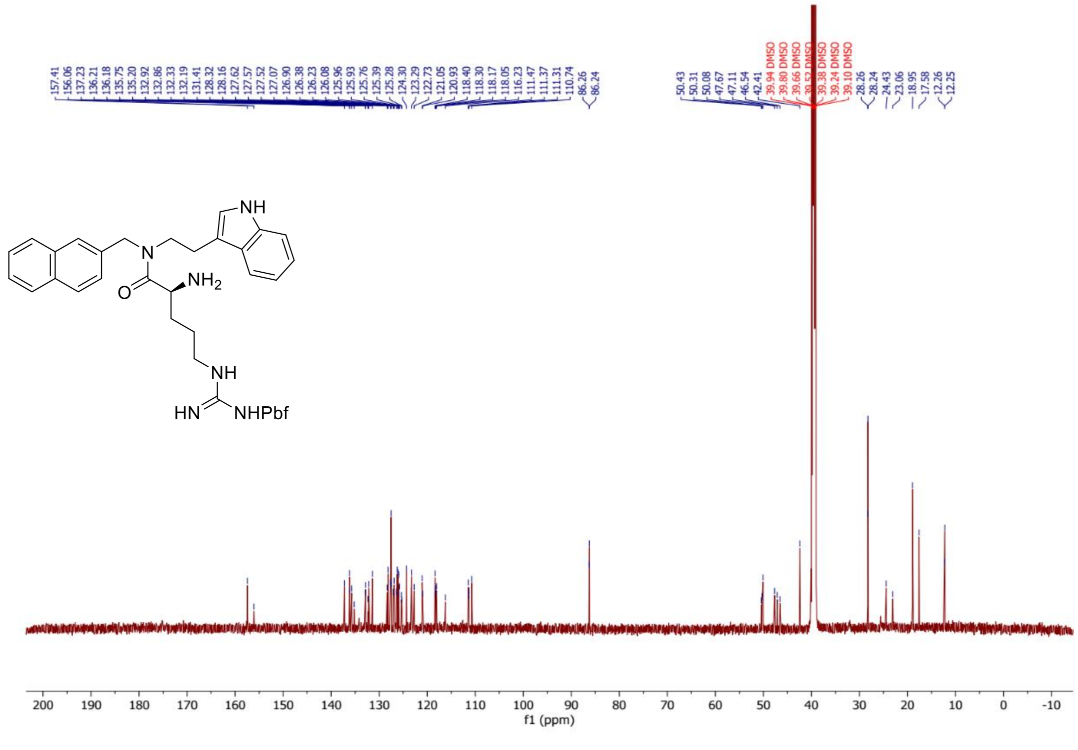

*91. Compound **22**- ^{13}CNMR dept 135*

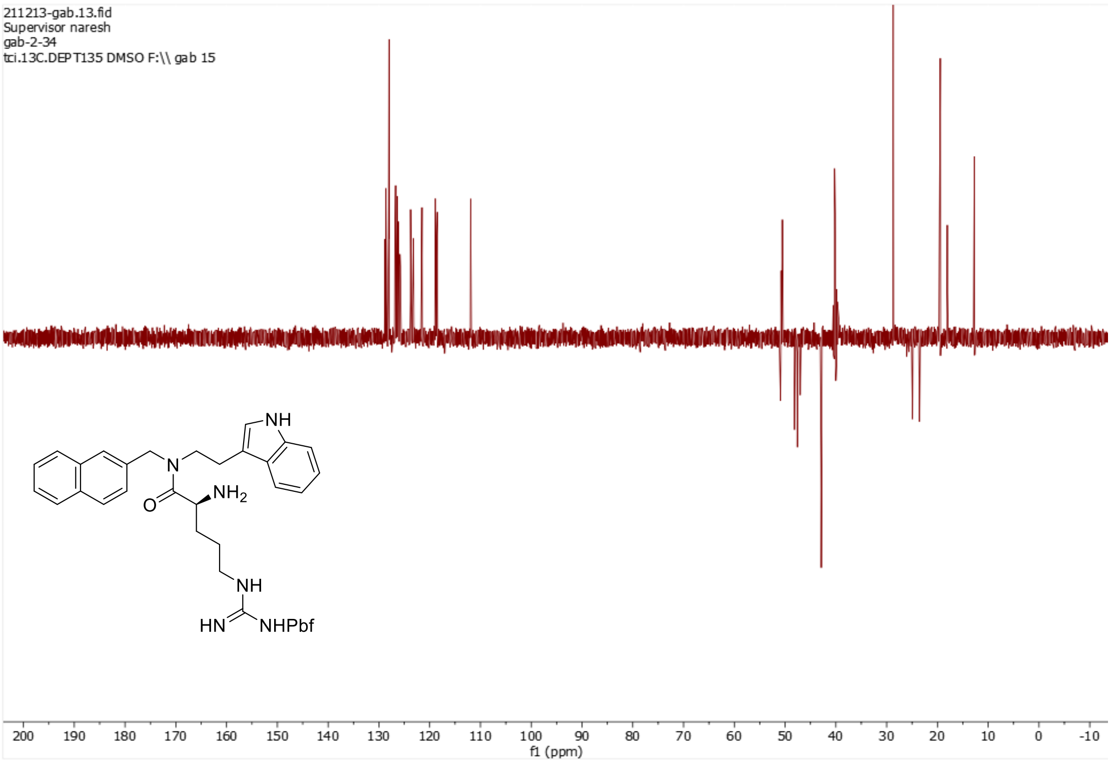

*92. Compound **22**- HSQC*

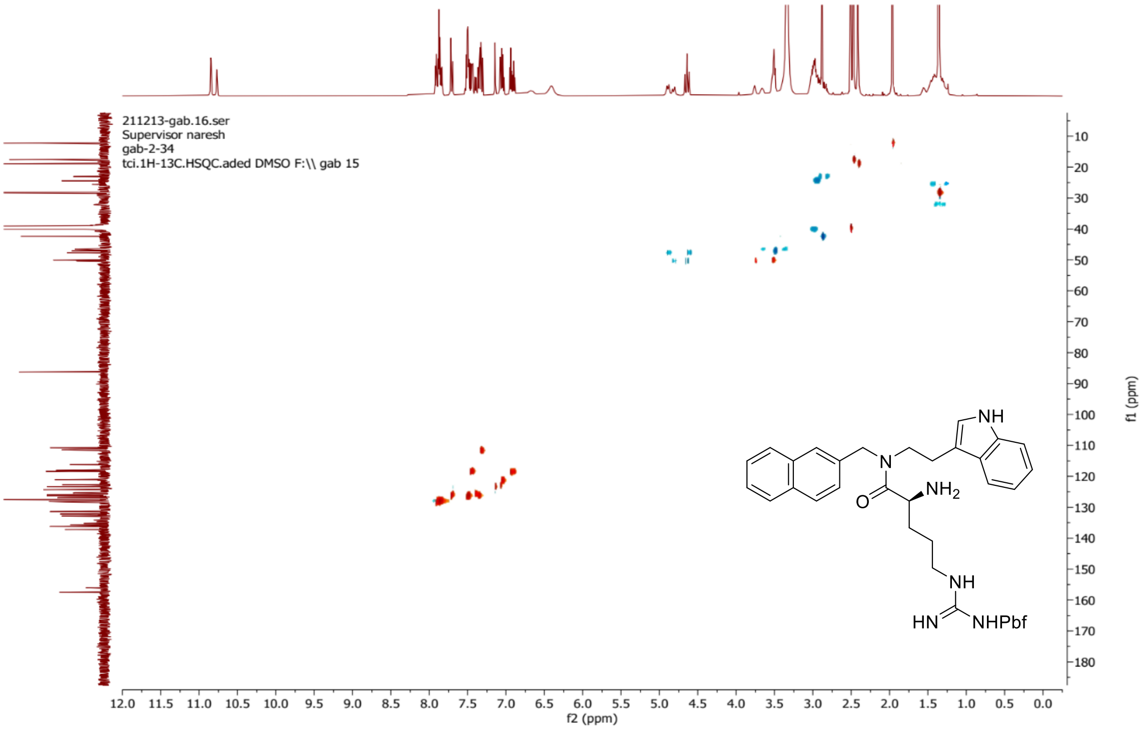

10- ^{1}H, and ^{13}CNMR of mono-guanidine naphthyl-indole and naphthyl-phenyl based peptoids**.**

*93. Compound **13a**- ^{1}HNMR*

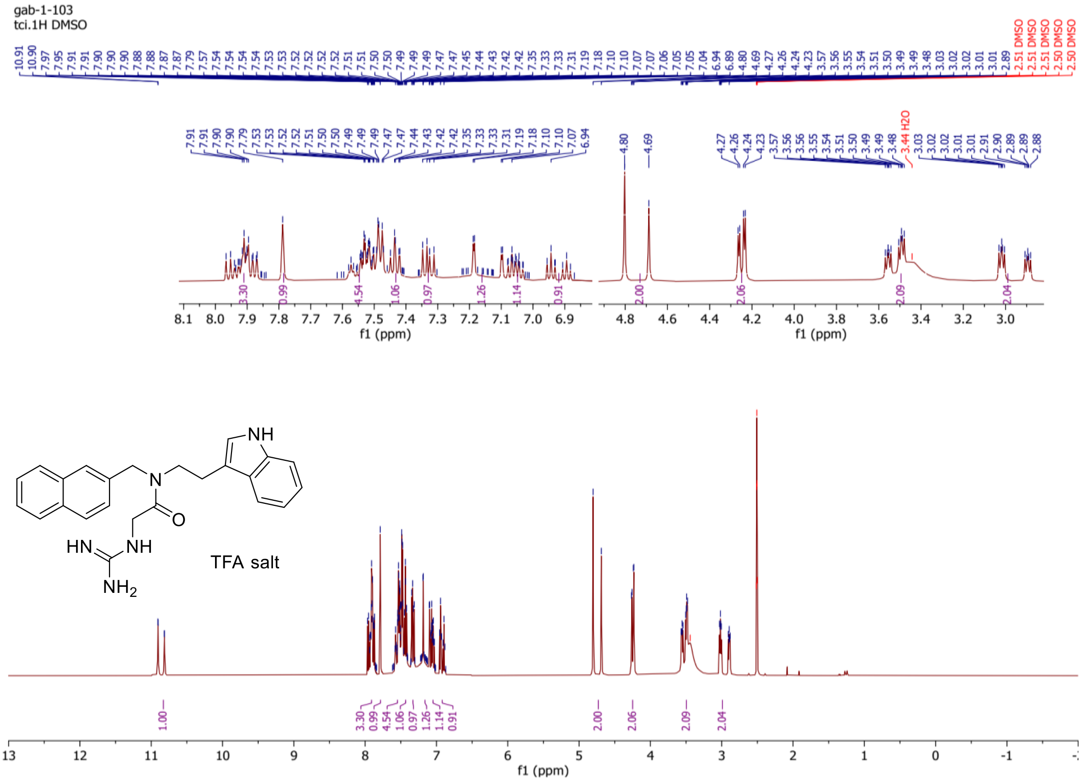

*94. Compound **13a**- ^{13}CNMR*

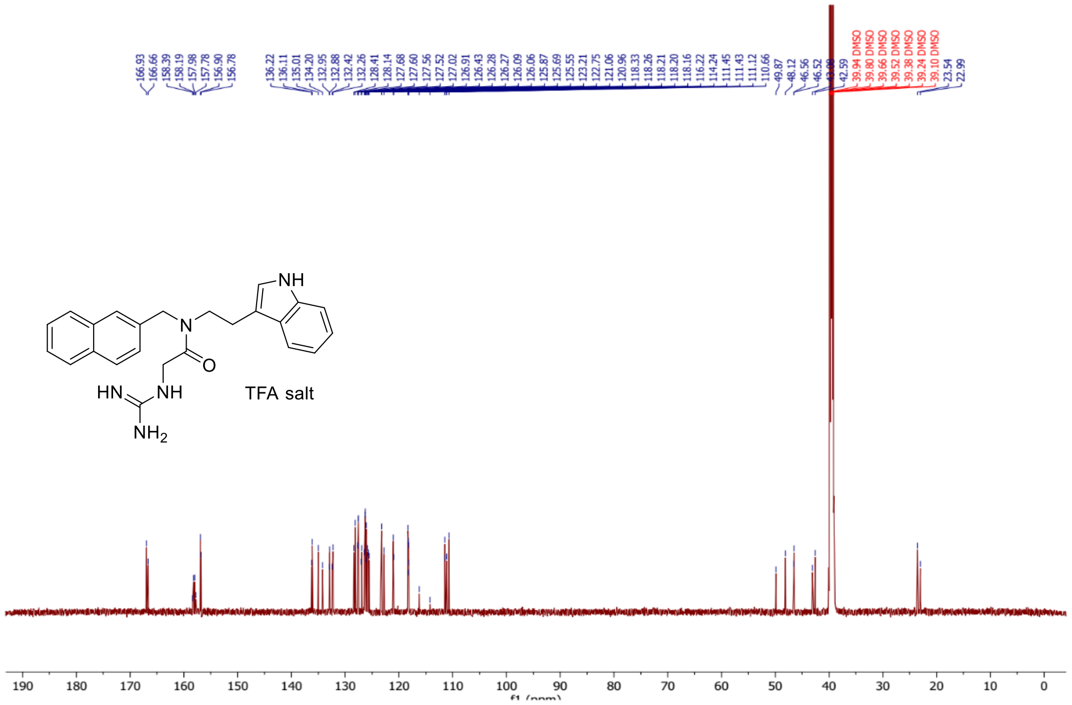

*95. Compound **13a**- ^{13}CNMR dept 135*

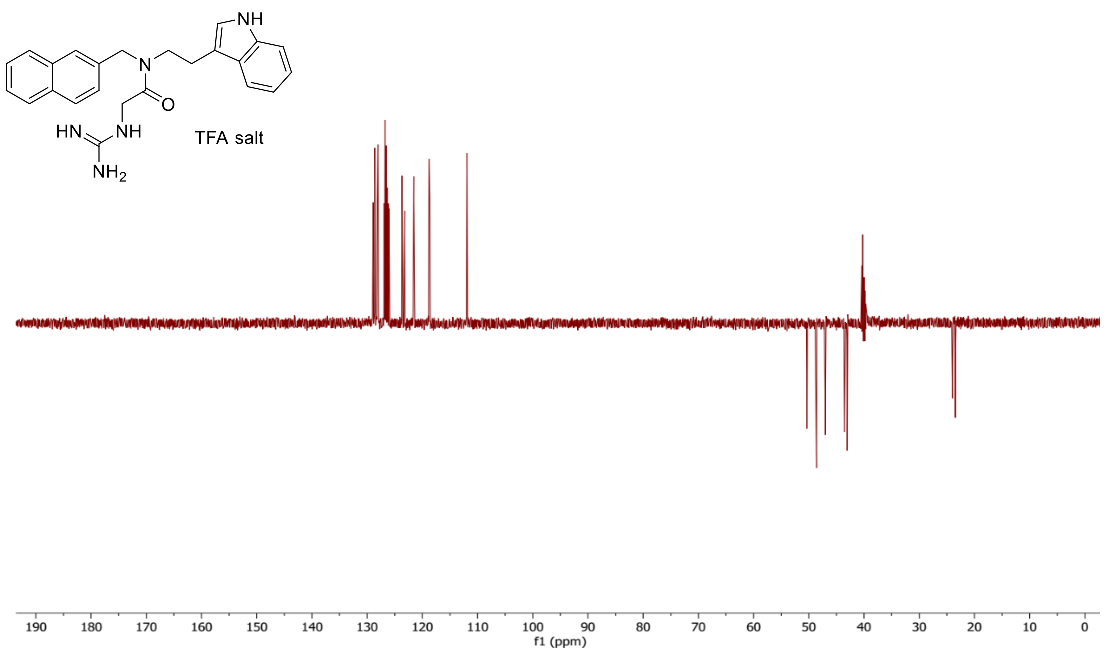

*96. Compound **13a**- HSQC*

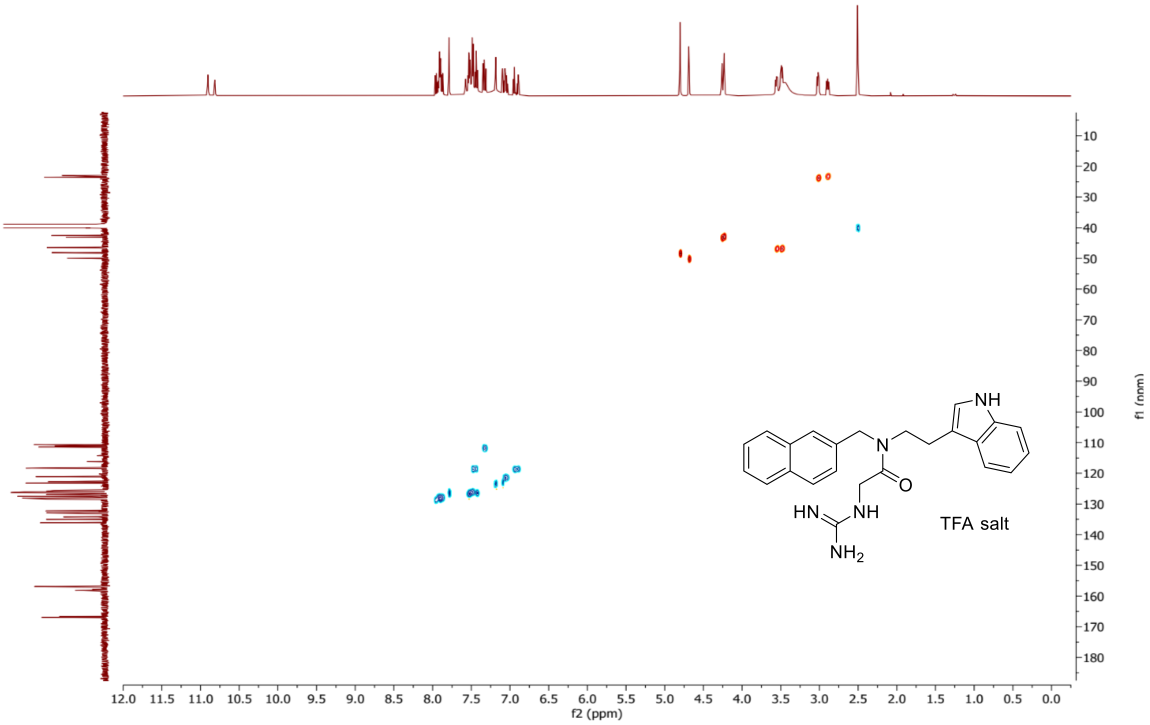

*97. Compound **13b**- ^{1}HNMR*

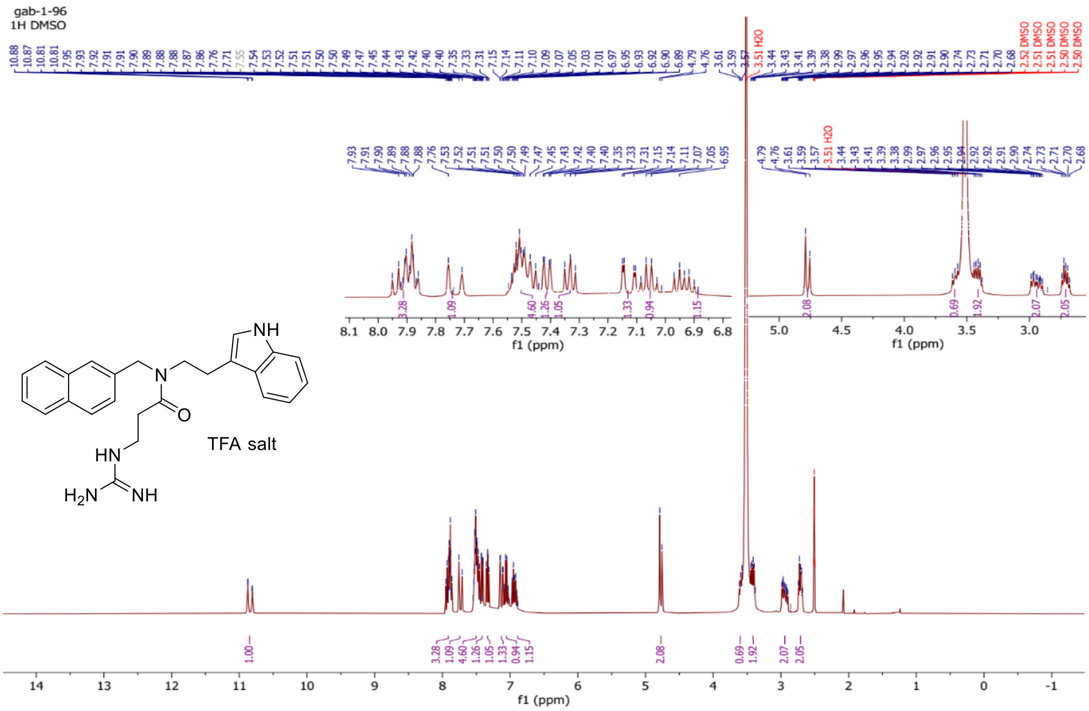

*98. Compound **13b**- ^{13}CNMR*

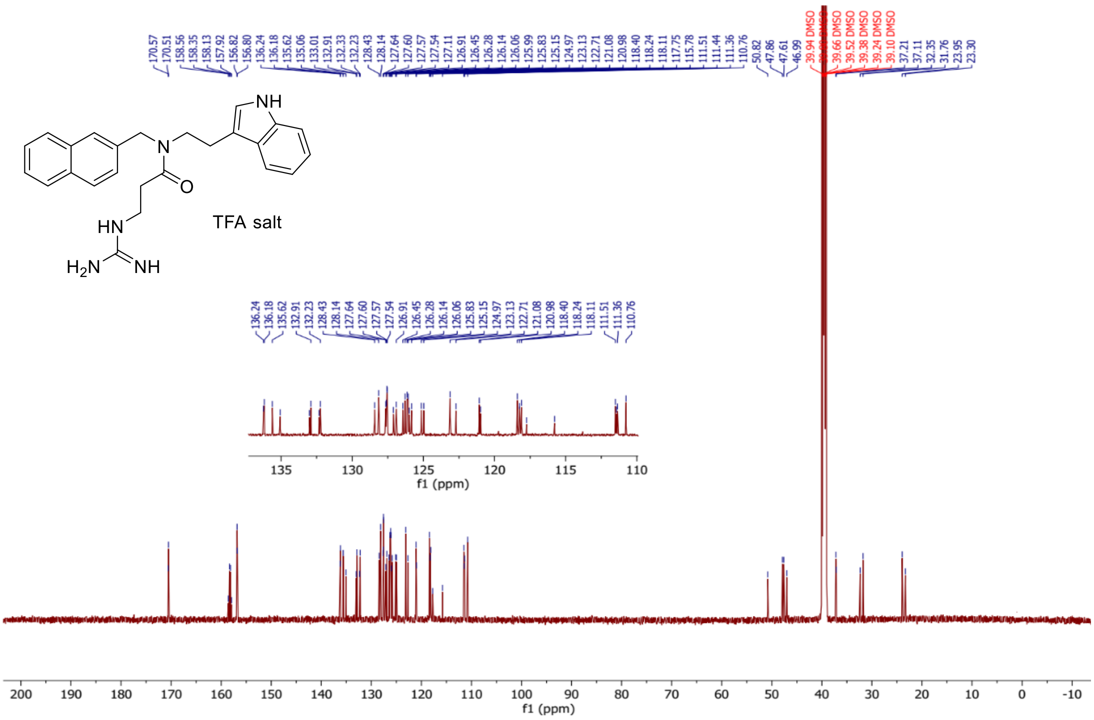

*99. Compound **13b**- ^{13}CNMR dept 135*

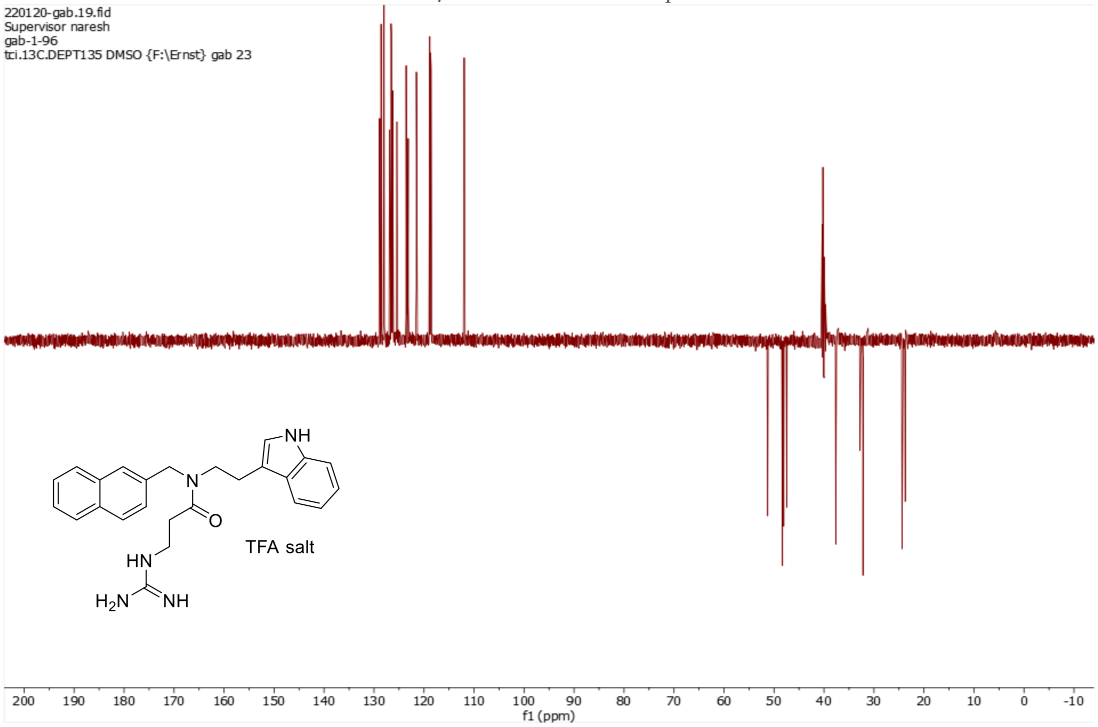

*100. Compound **13b**-HSQC*

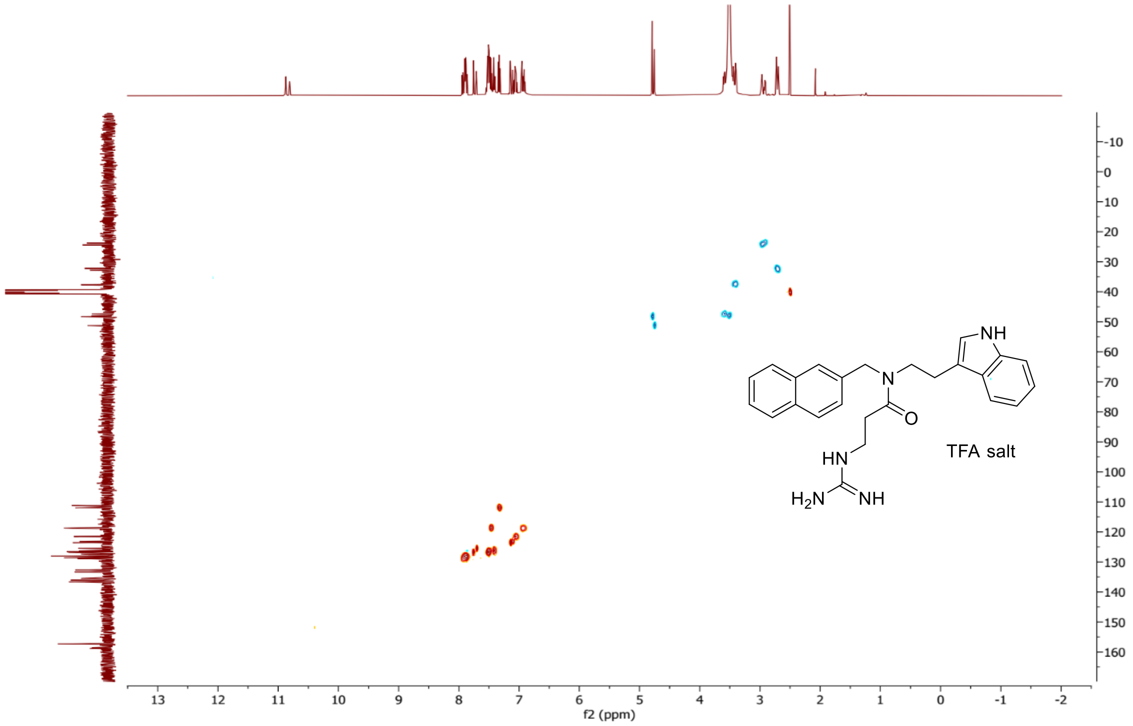

*101. Compound **13c**- ^{1}HNMR*

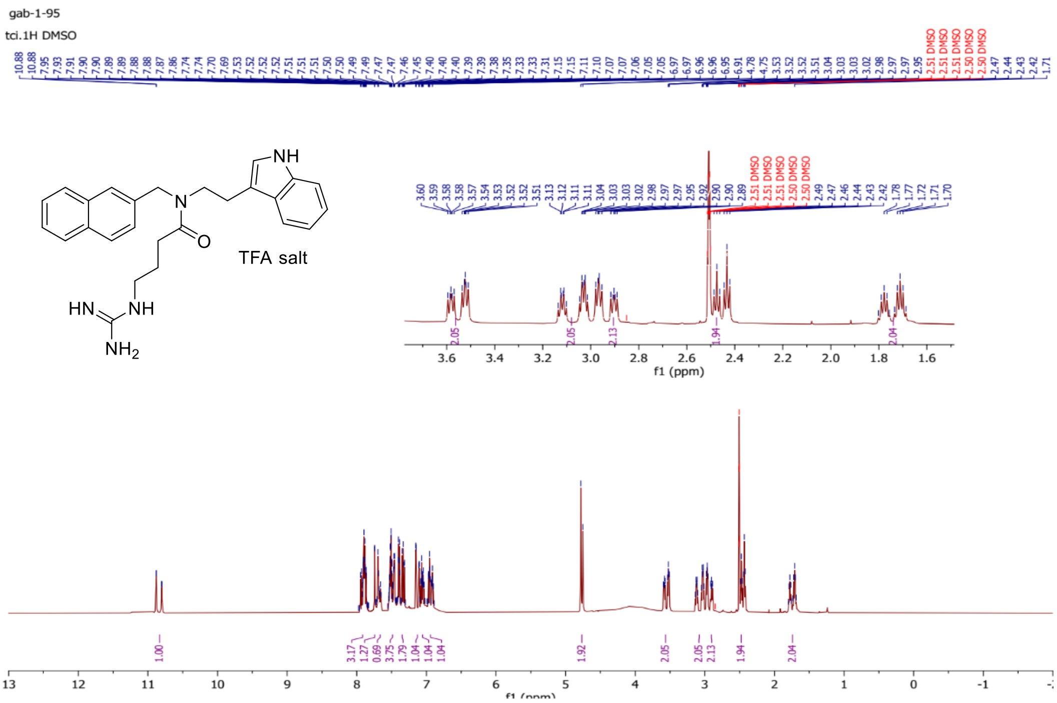

*102. Compound **13c**- ^{13}CNMR*

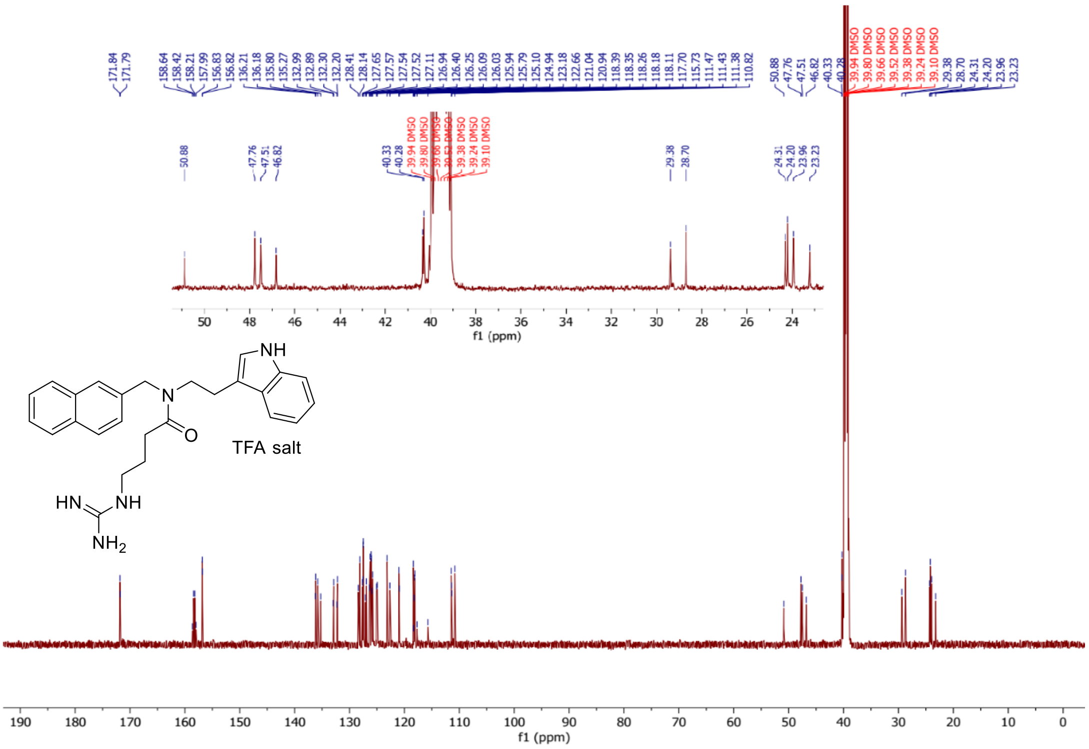

*103. Compound **13c**- ^{13}CNMR dept 135*

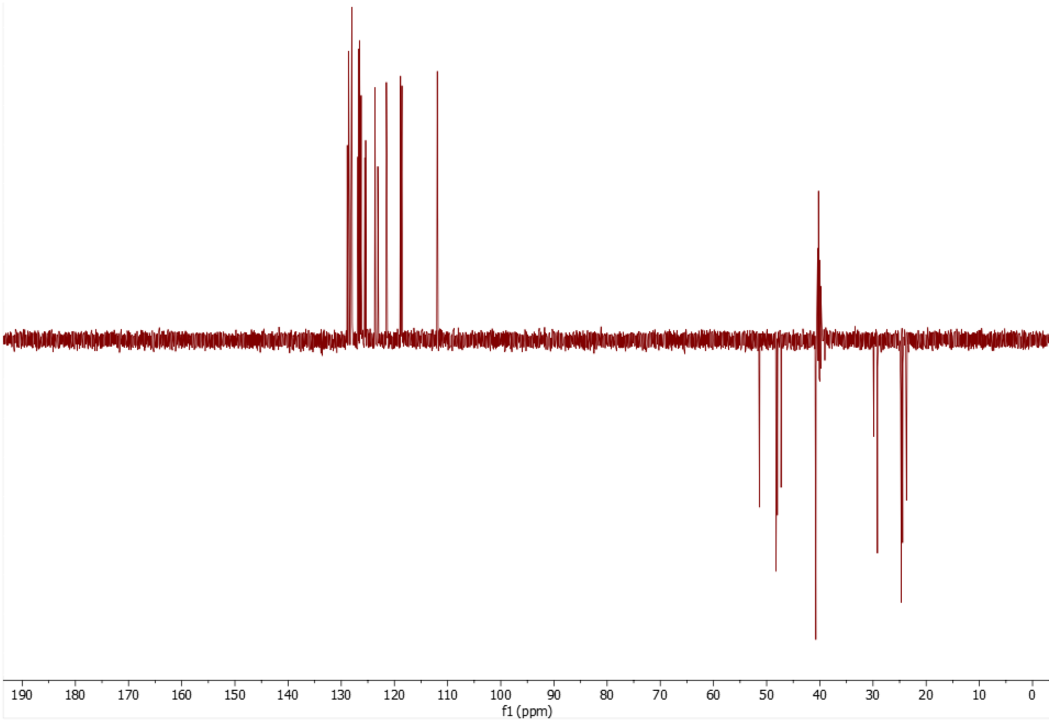

*104. Compound **13c**- HSQC*

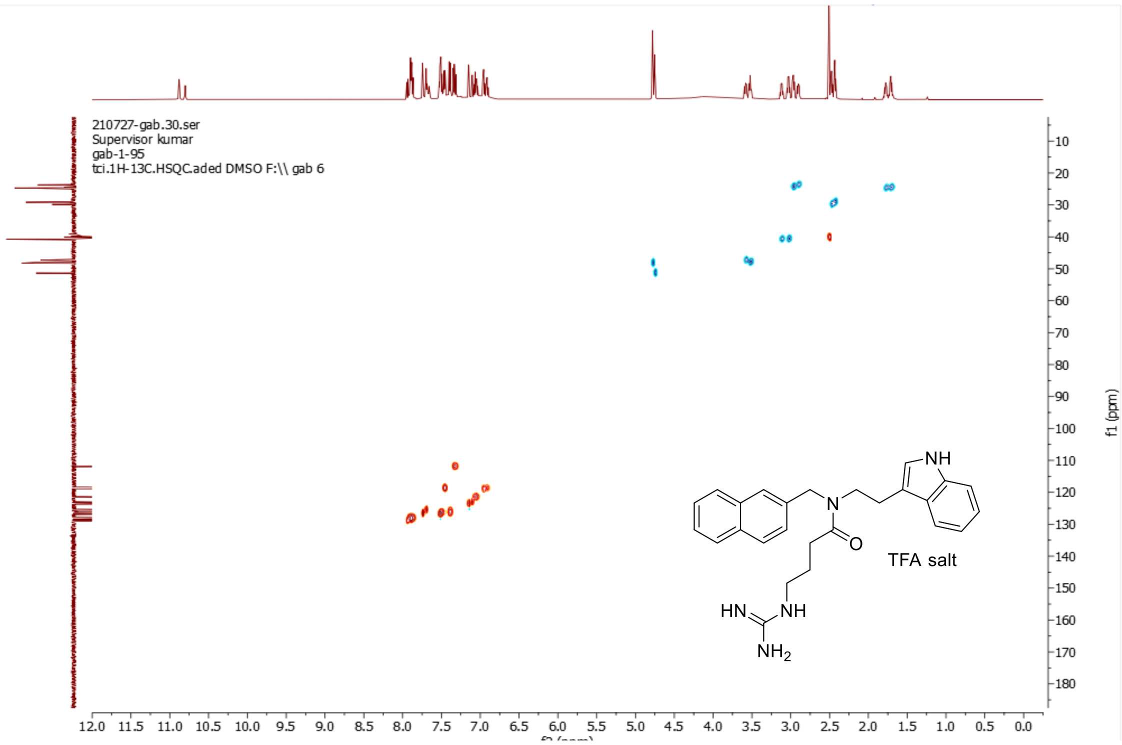

*105. Compound **13d**- ^{1}HNMR*

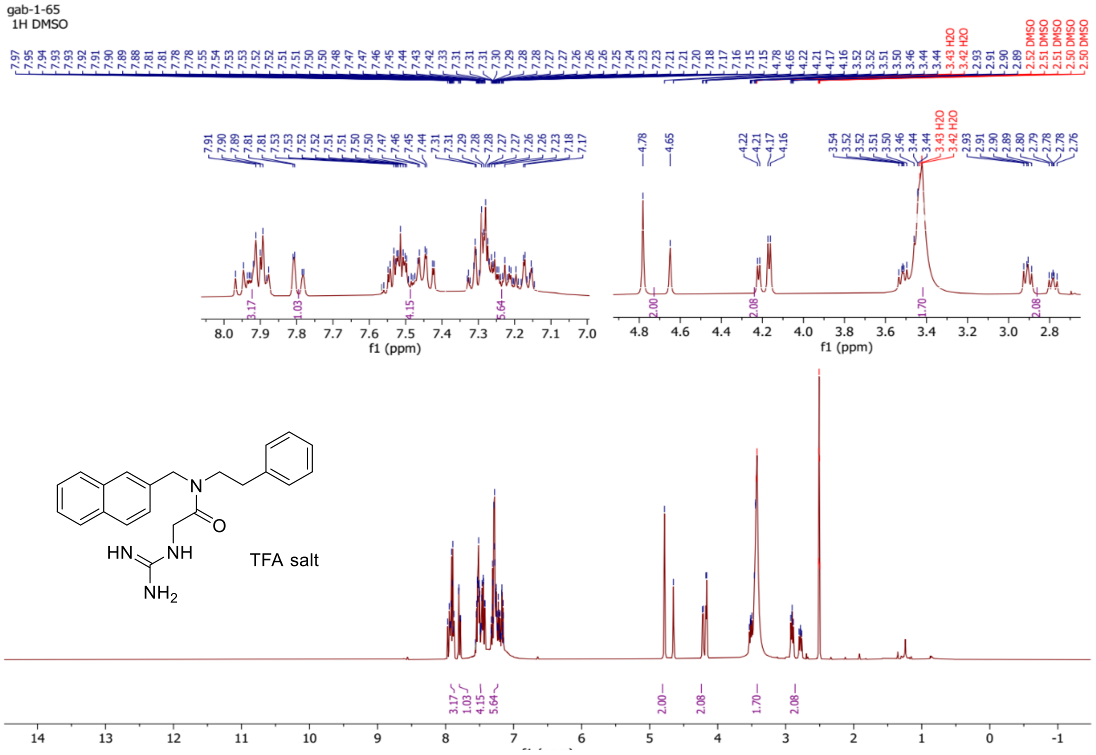

*106. Compound **13d**- ^{13}CNMR*

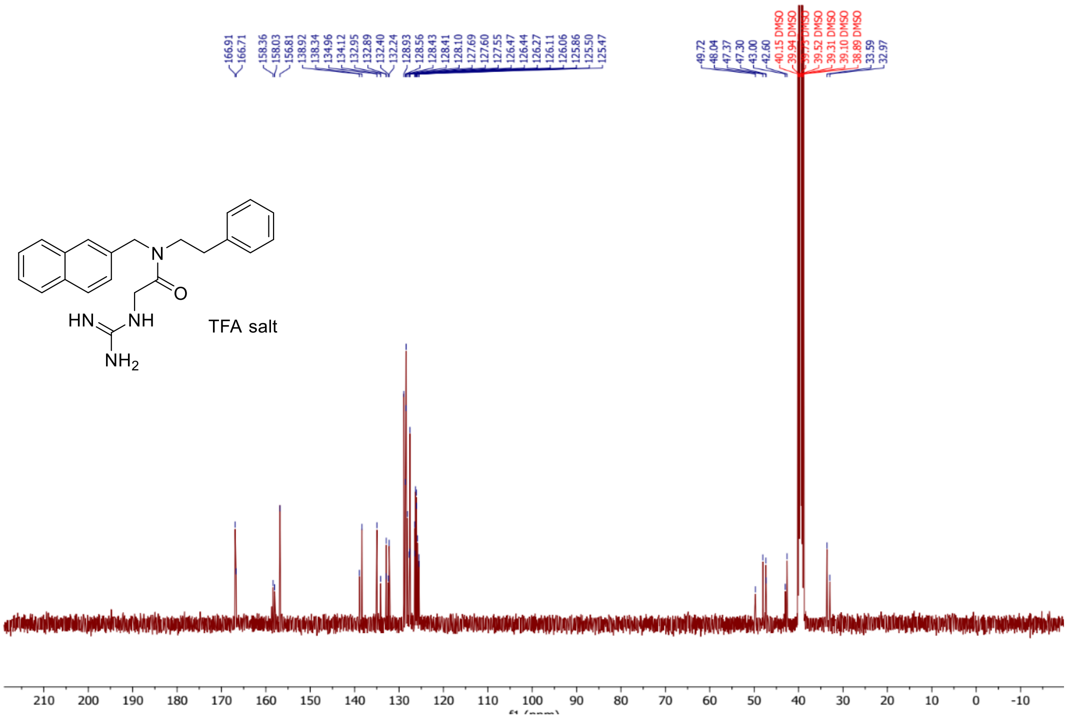

*107. Compound **13d**- ^{13}CNMR dept 135*

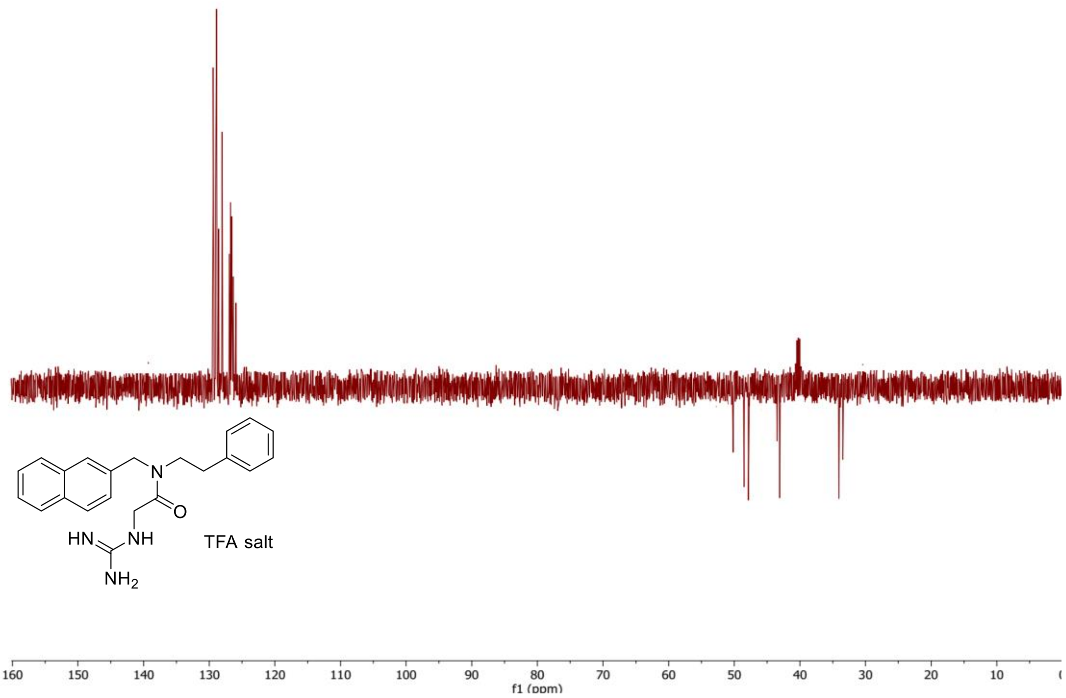

*108. Compound **13d**- HSQC*

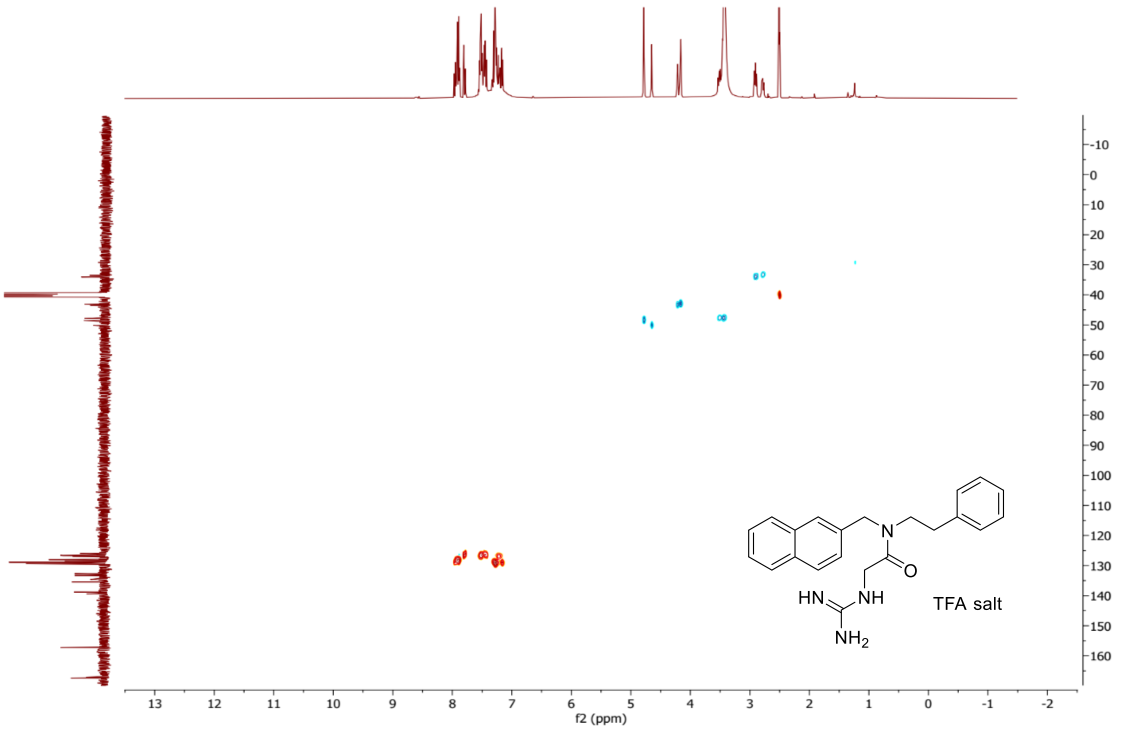

*109. Compound **13e**- ^{1}HNMR*

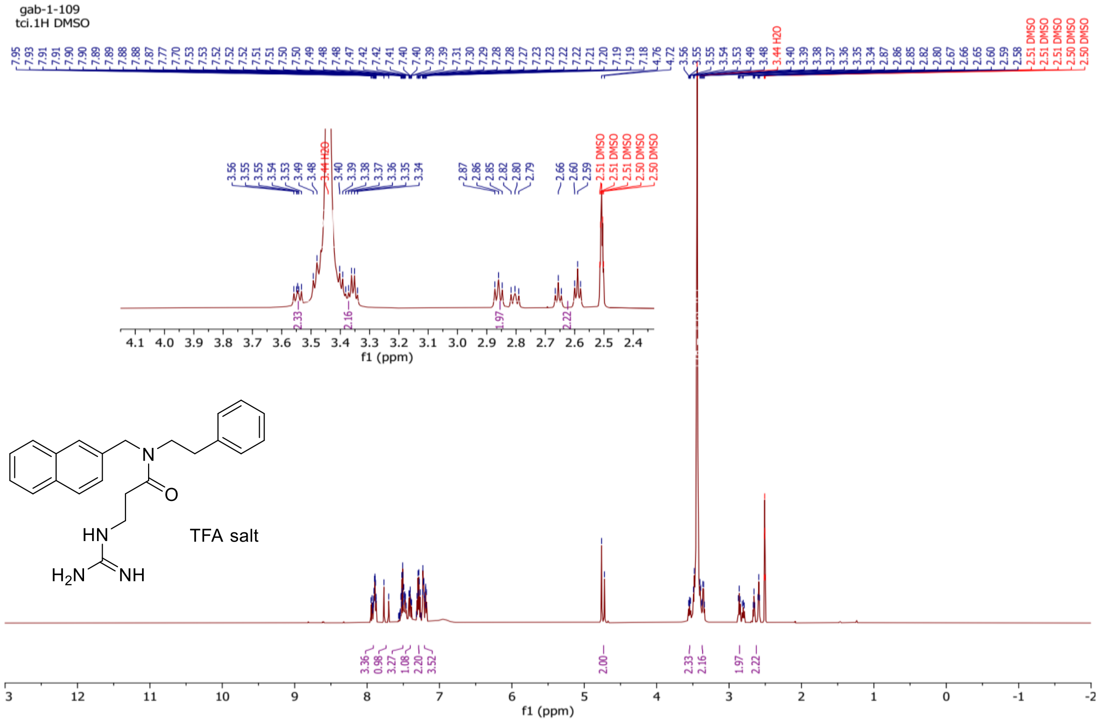

*110. Compound **13e**- ^{13}CNMR*

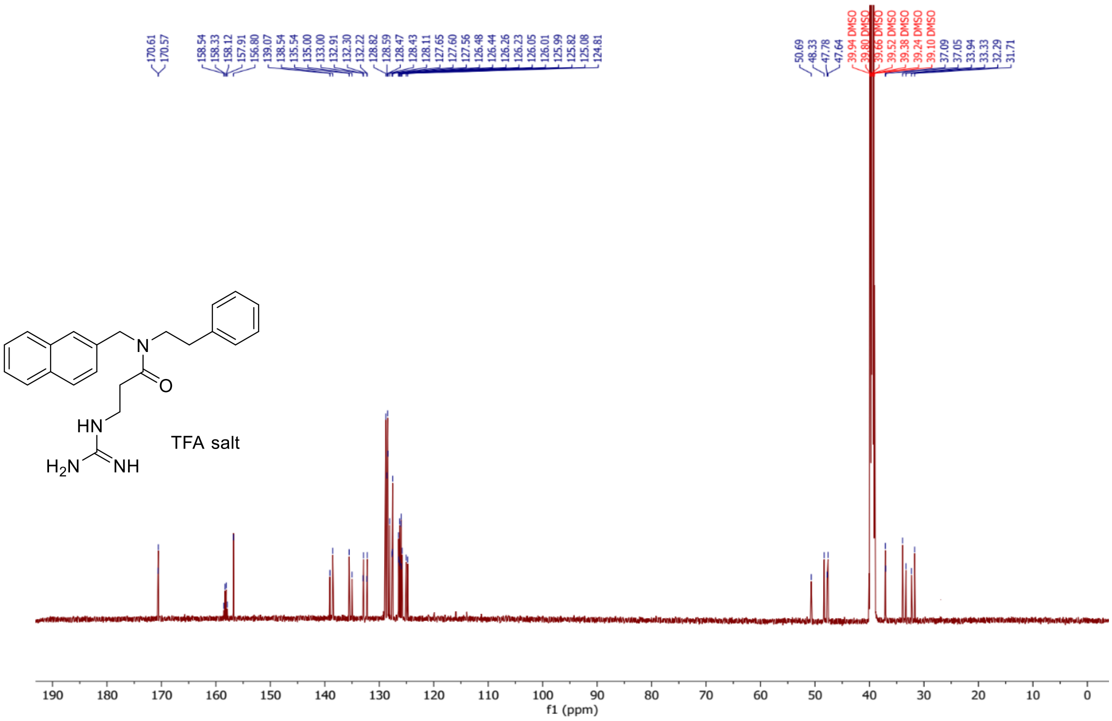

*111. Compound **13e**- ^{13}CNMR dept 135*

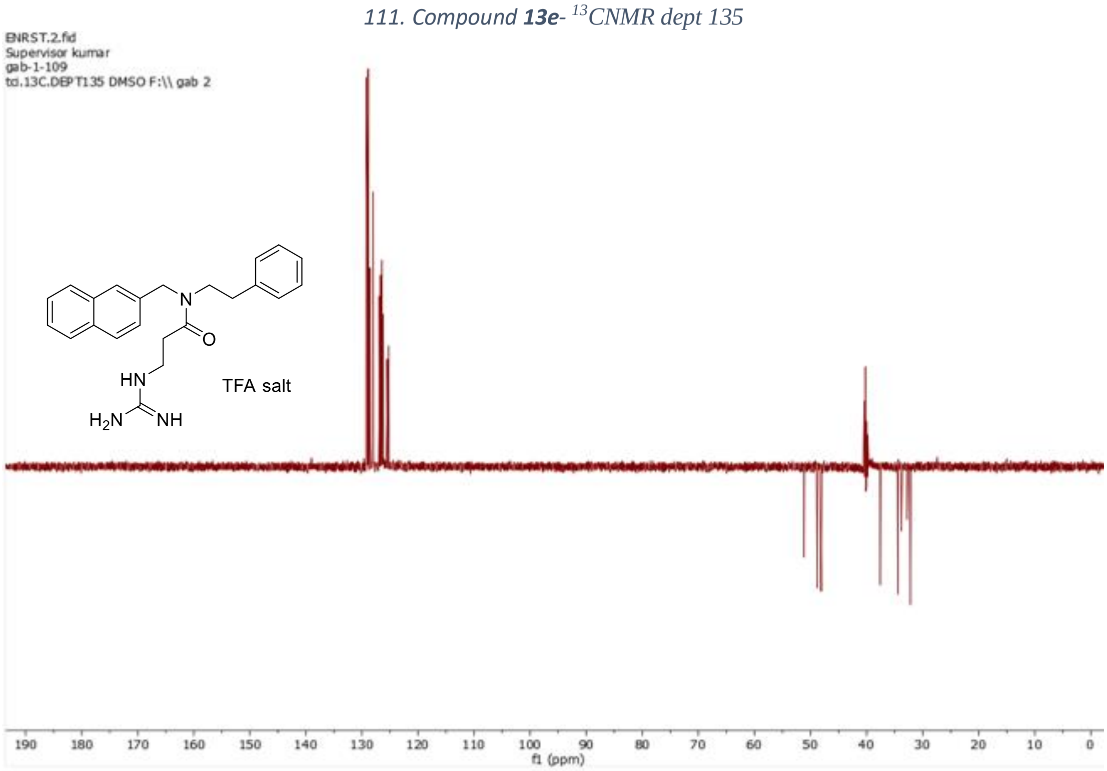

*112. Compound **13e**- HSQC*

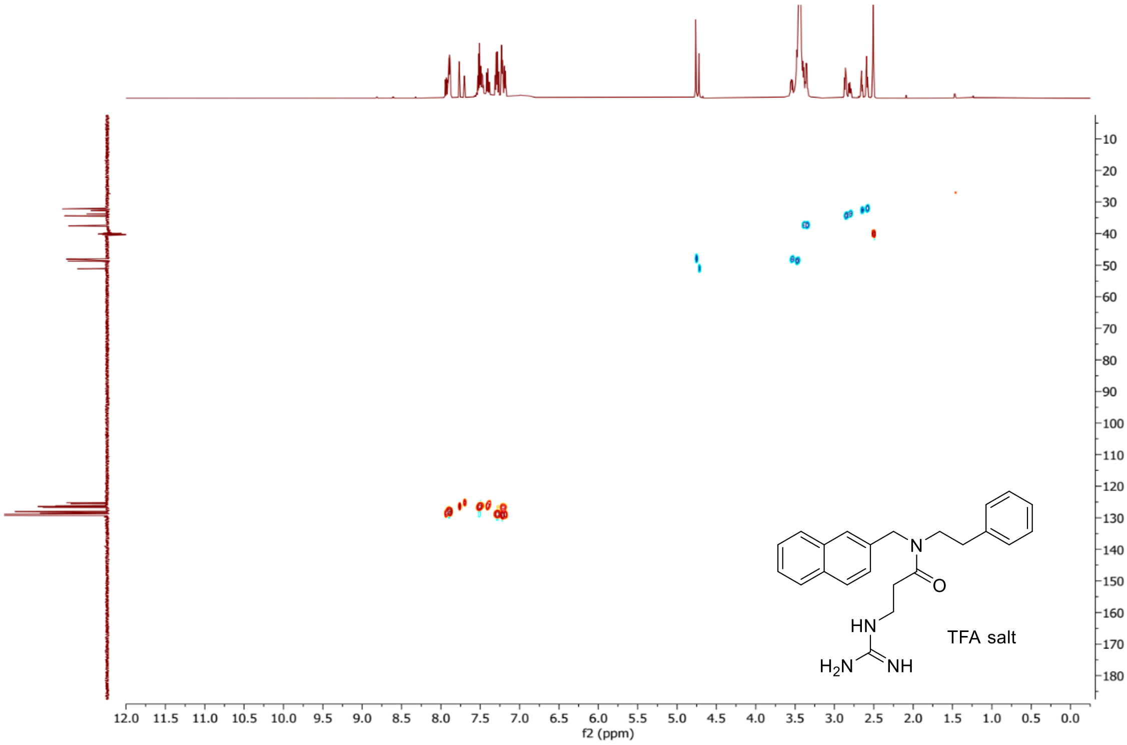

*113. Compound **13f**- ^{1}HNMR*

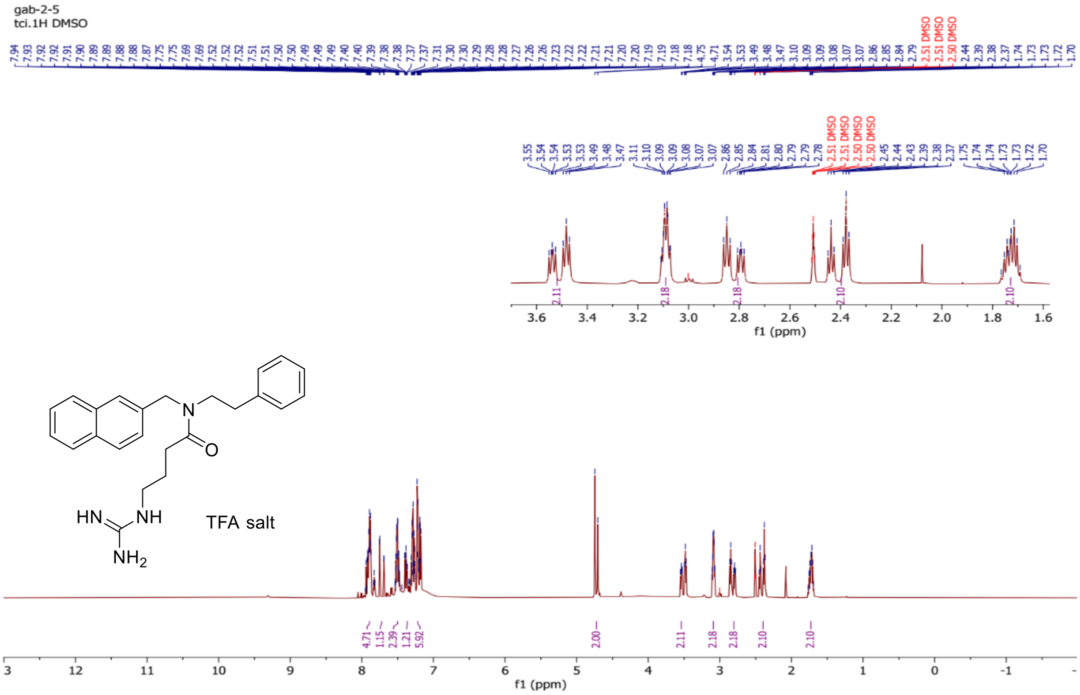

*114. Compound **13f**- ^{13}CNMR*

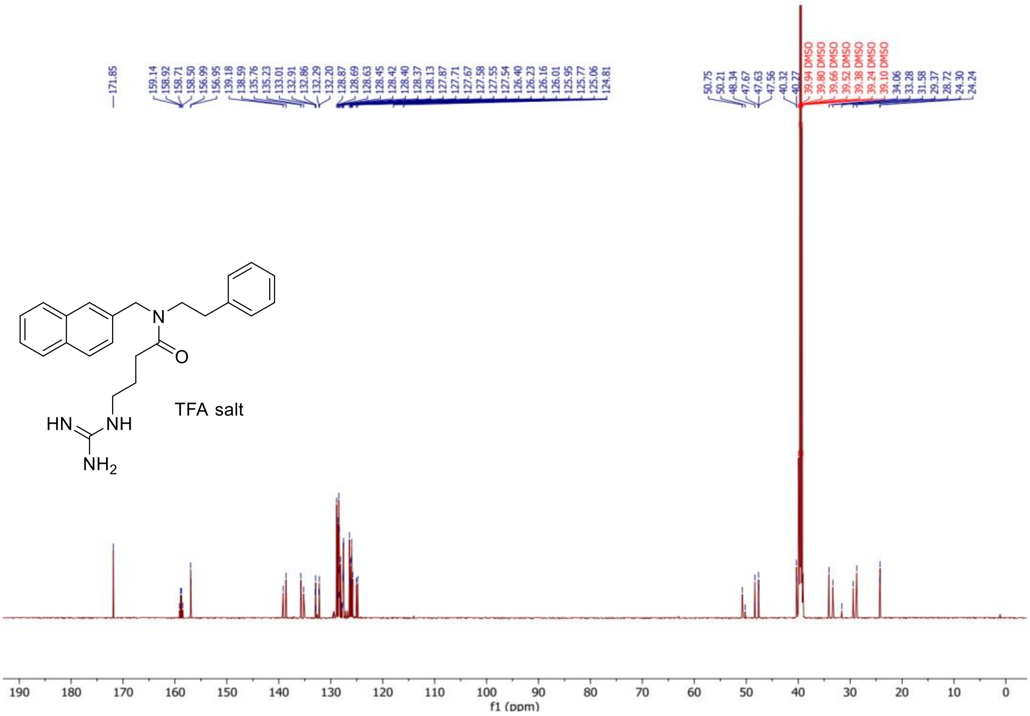

*115. Compound **13f**- ^{13}CNMR dept 135*

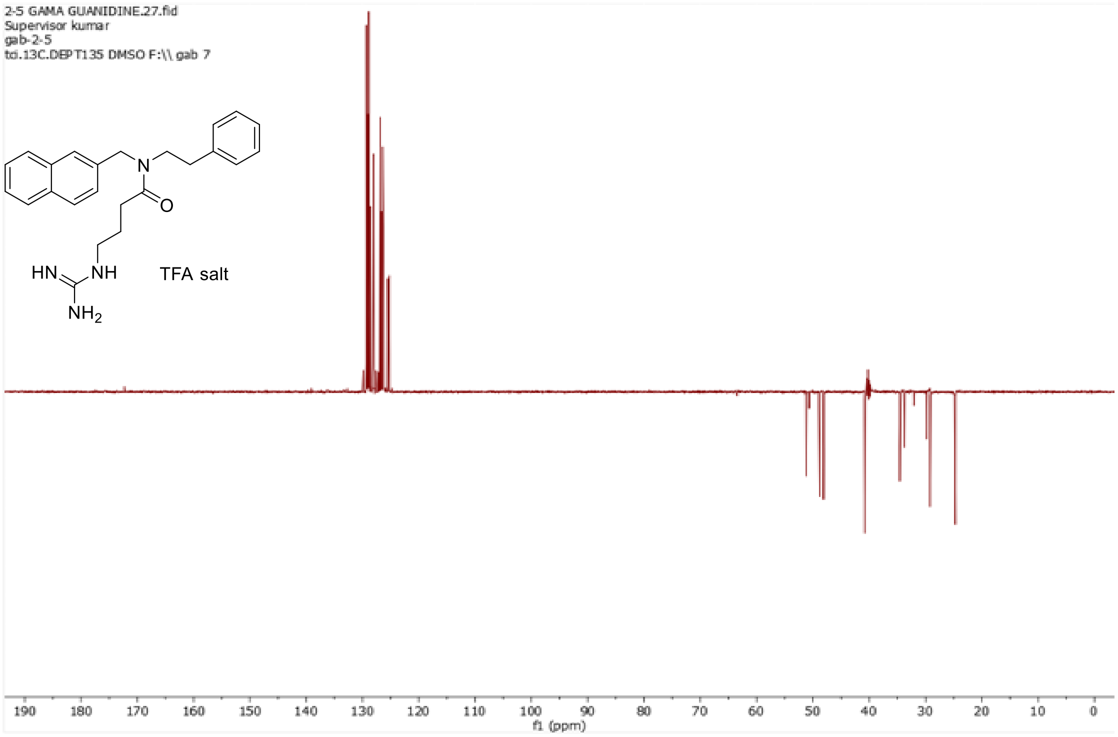

*116. Compound **13f**- HSQC*

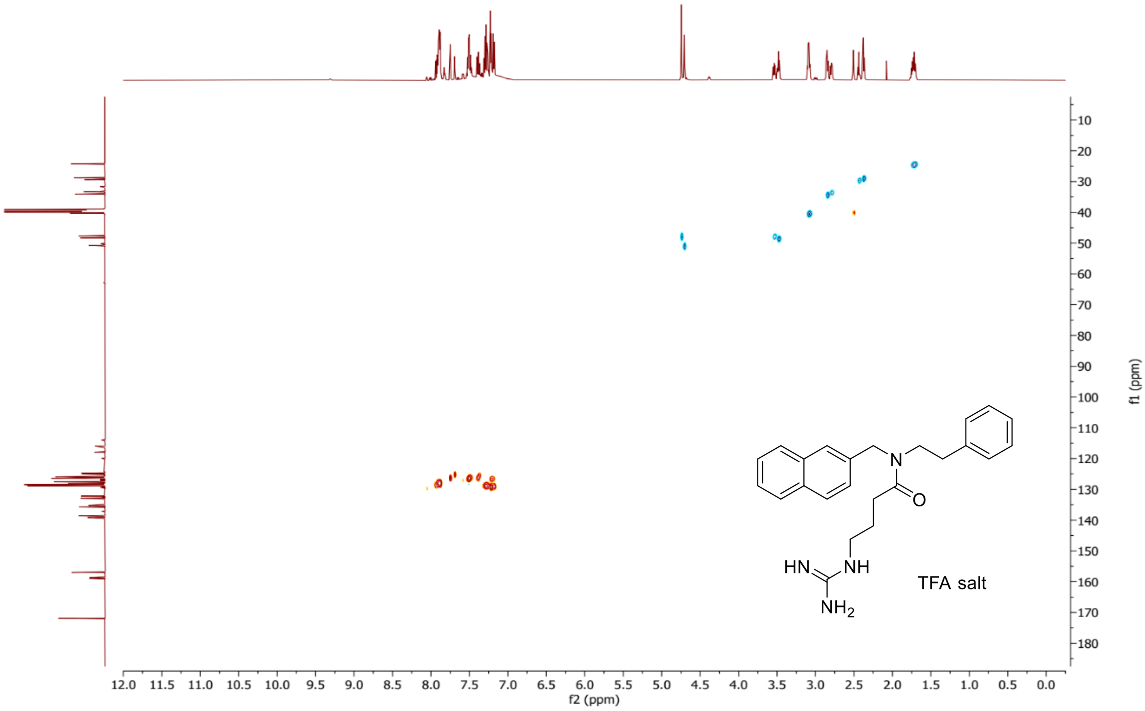

11- ^{1}H, and ^{13}CNMR of di-guanidine-naphthyl- indole based peptoids.

*117. Compound **20b**- ^{1}HNMR*

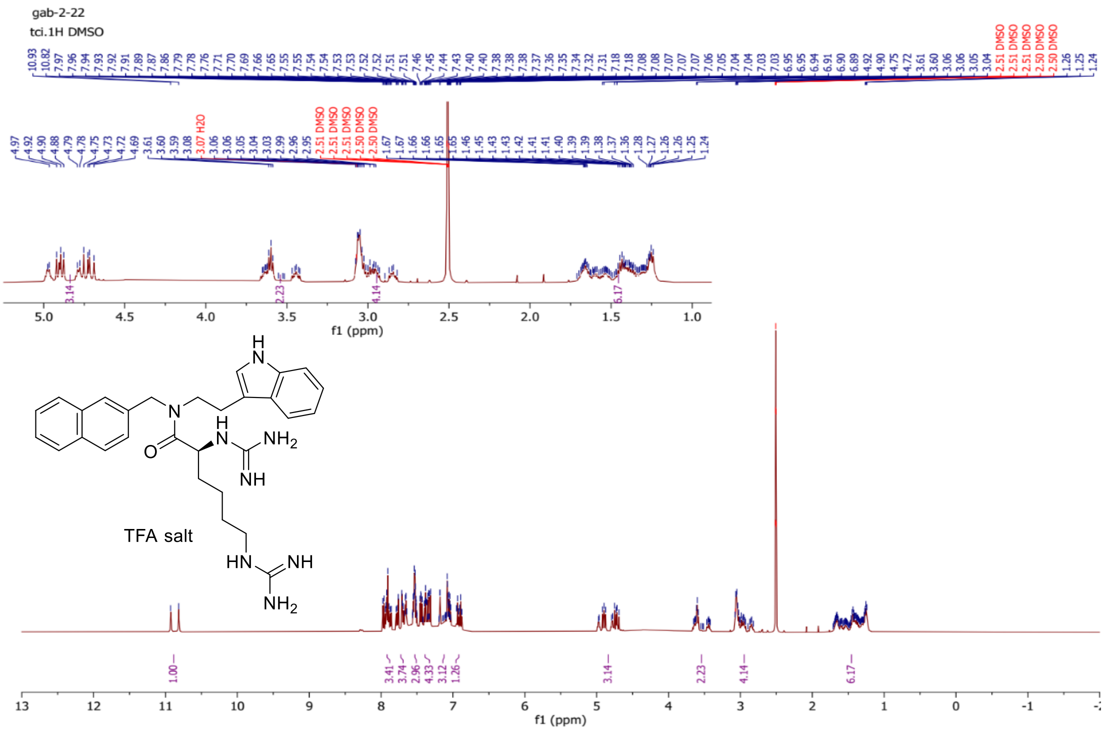

*118. Compound **20b**- ^{13}CNMR*

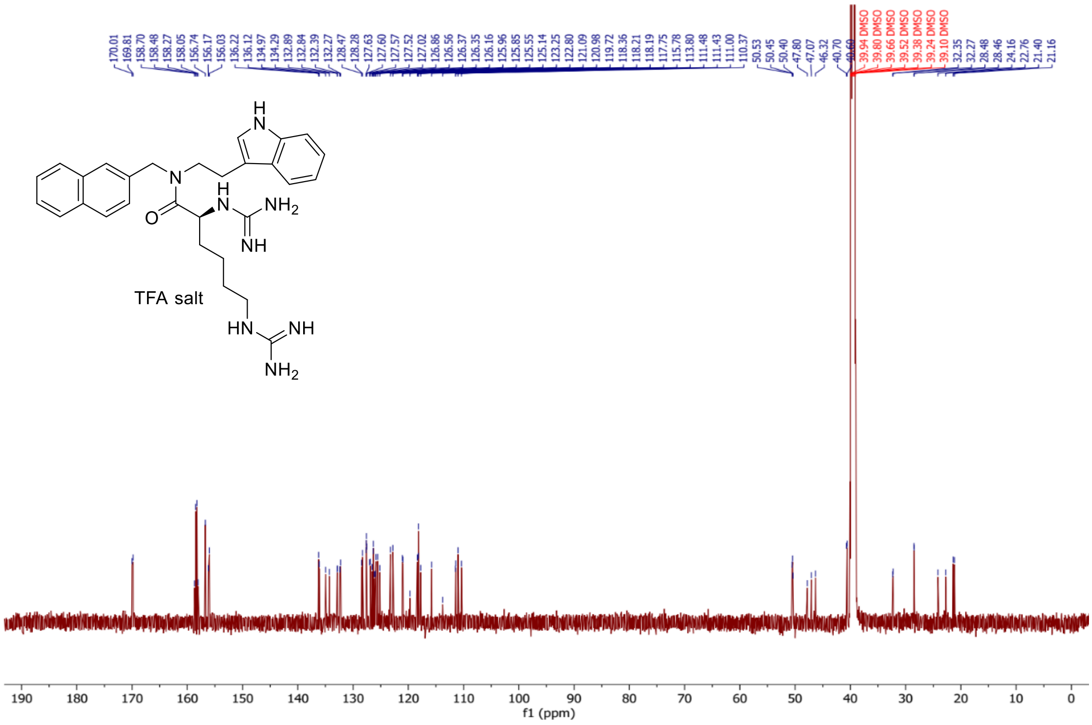

*119. Compound **20b**- ^{13}CNMR dept 135*

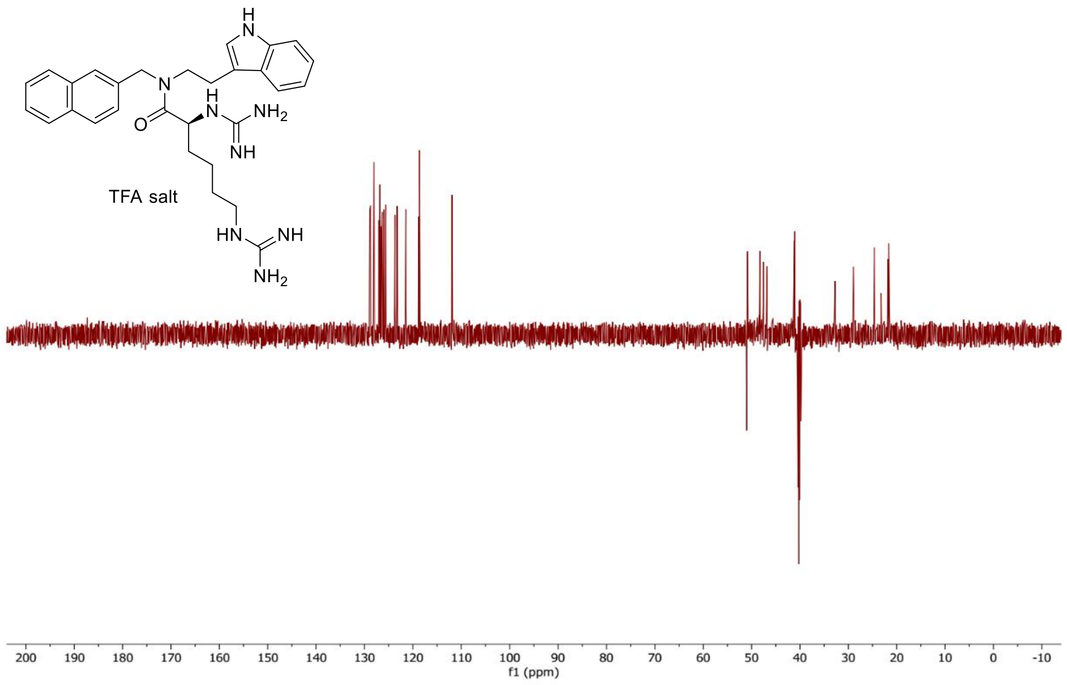

*120. Compound **20b**- HSQC*

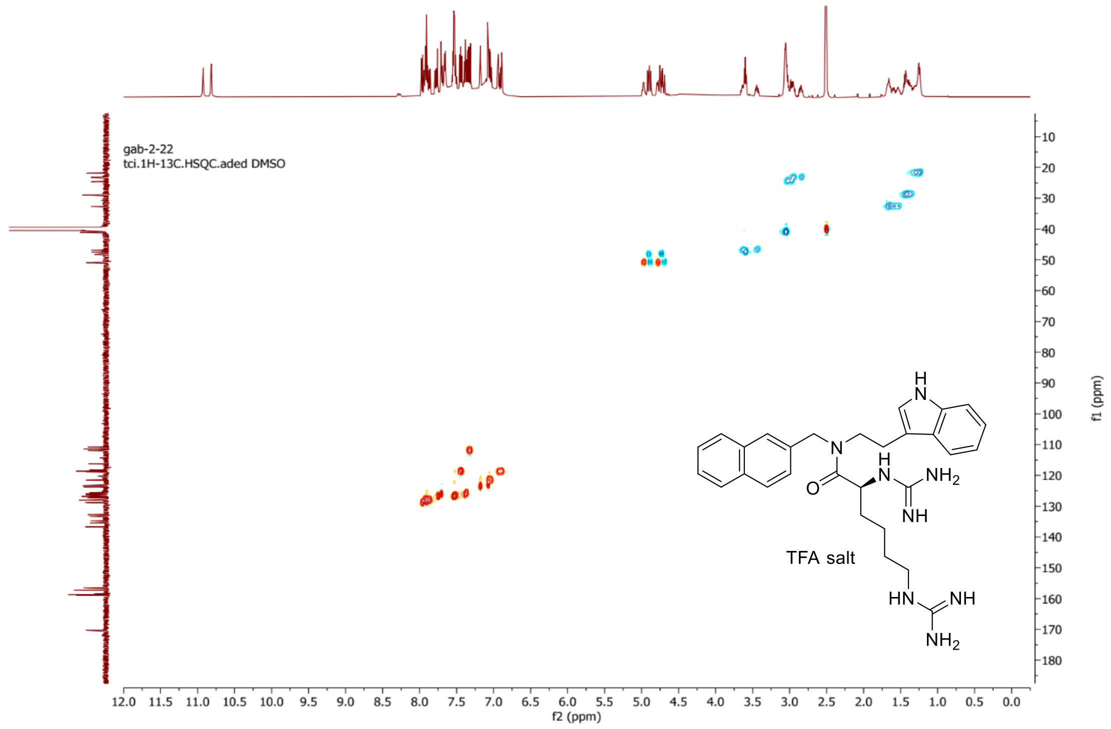

*121. Compound **20a**- ^{1}HNMR*

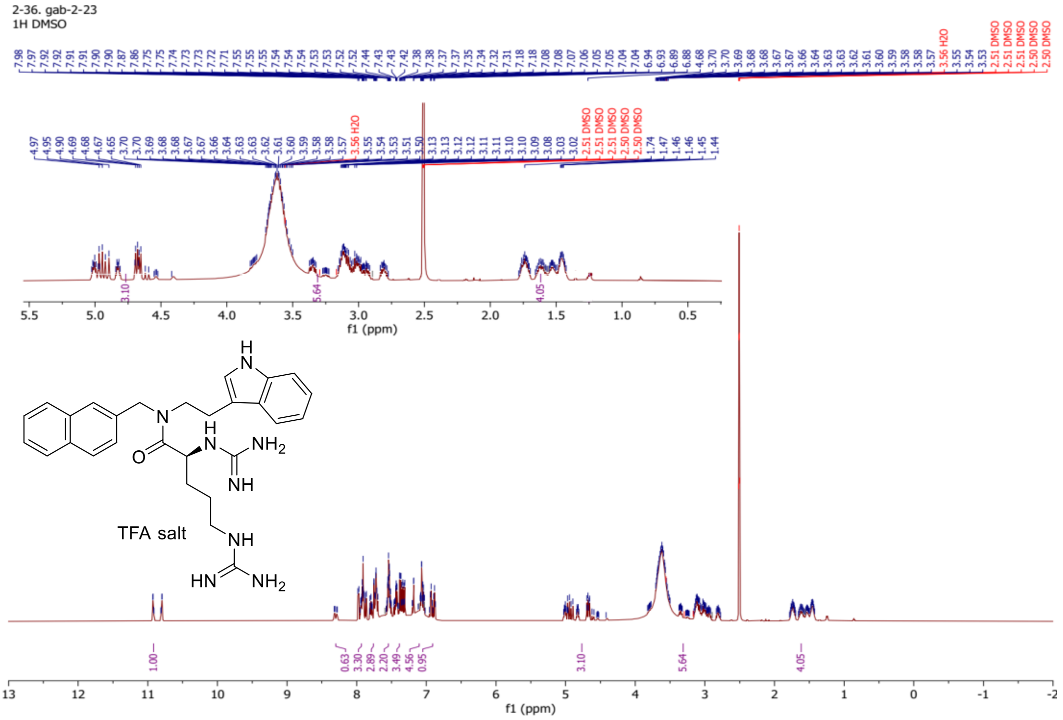

*122. Compound **20a**- ^{13}CNMR*

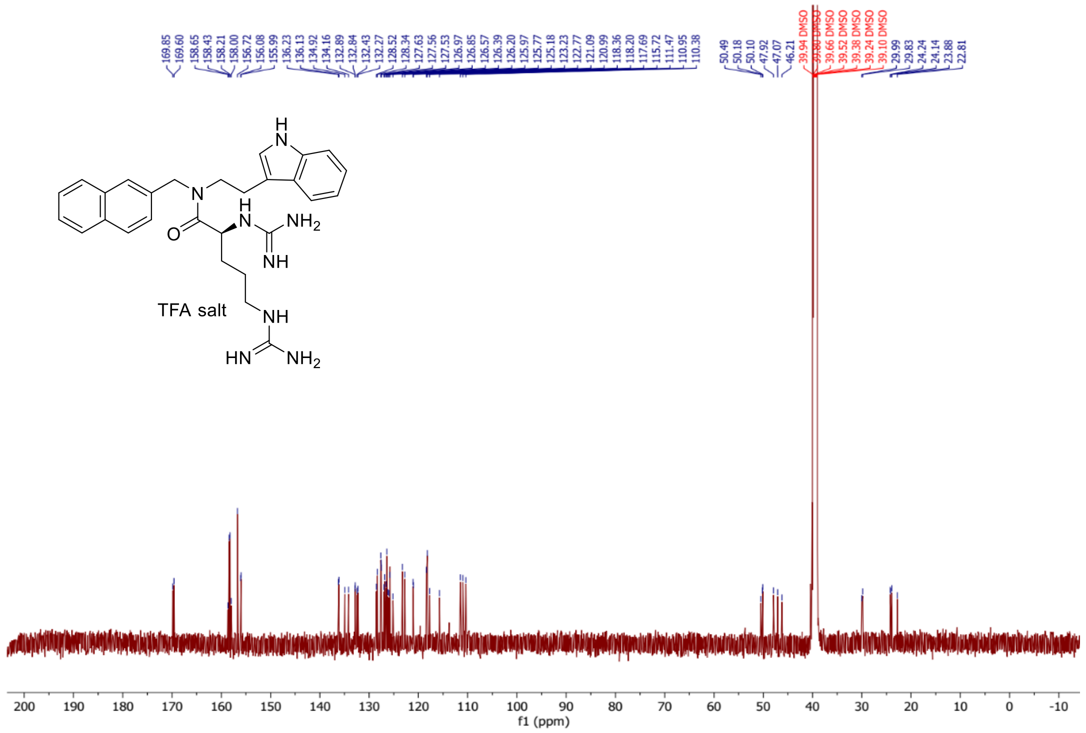

*123. Compound **20a**- ^{13}CNMR dept 135*

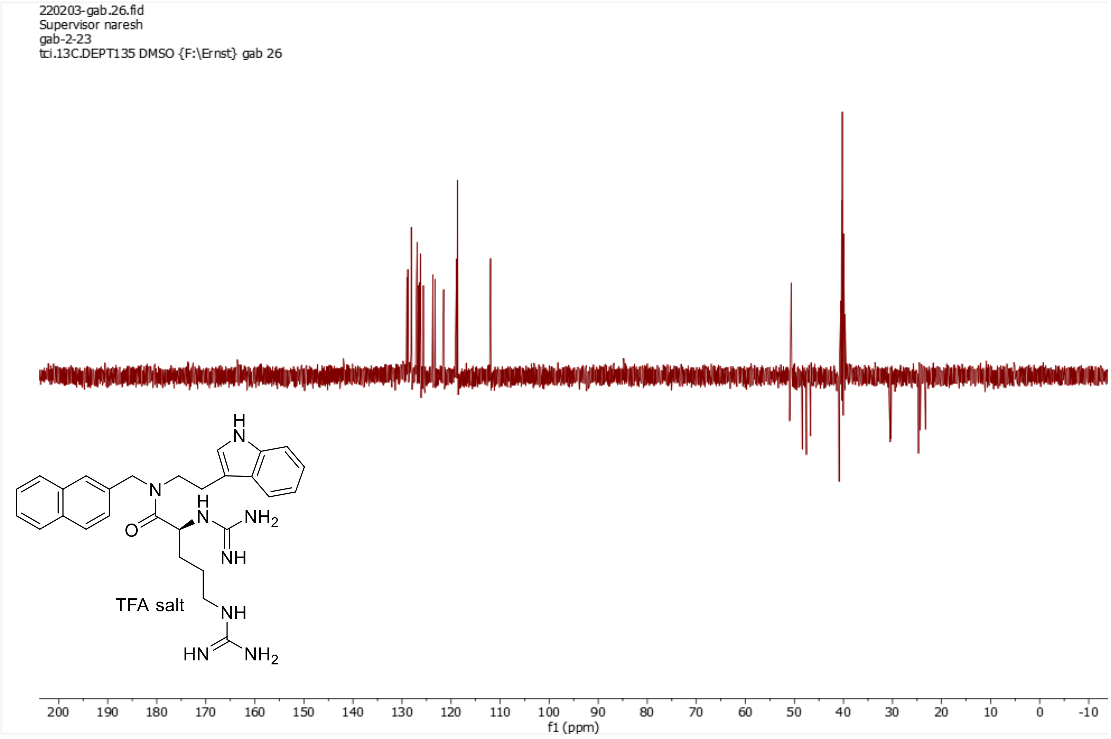

*124. Compound **20a**- HSQC*

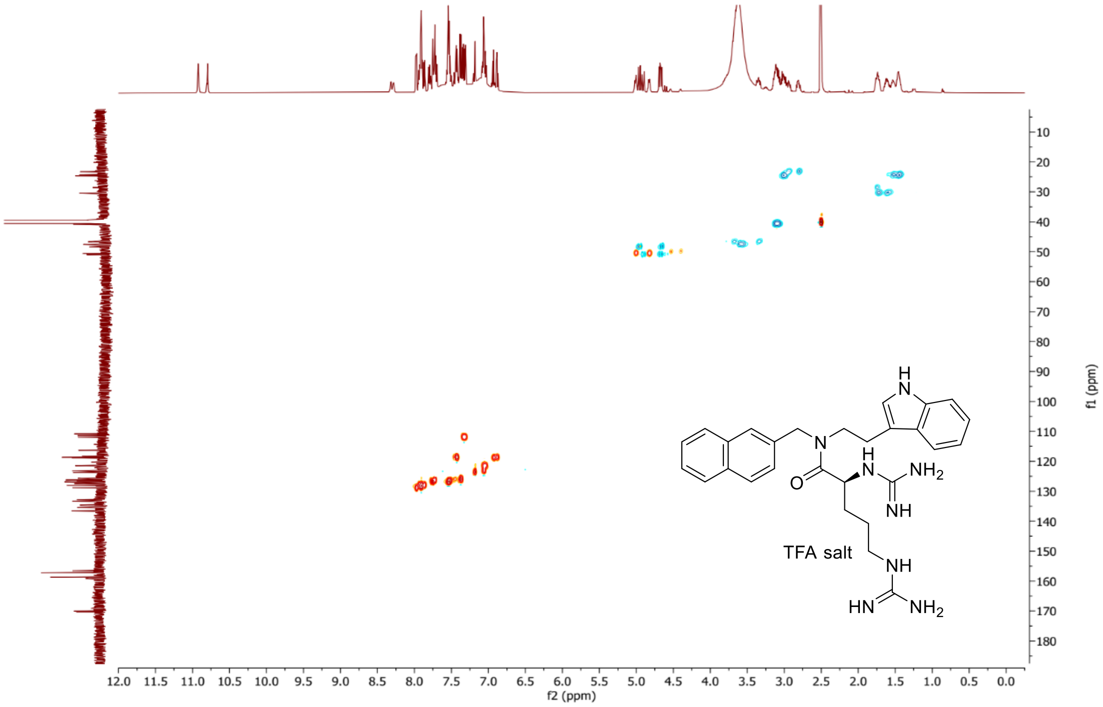

*125. Compound **23** -^{1}HNMR*

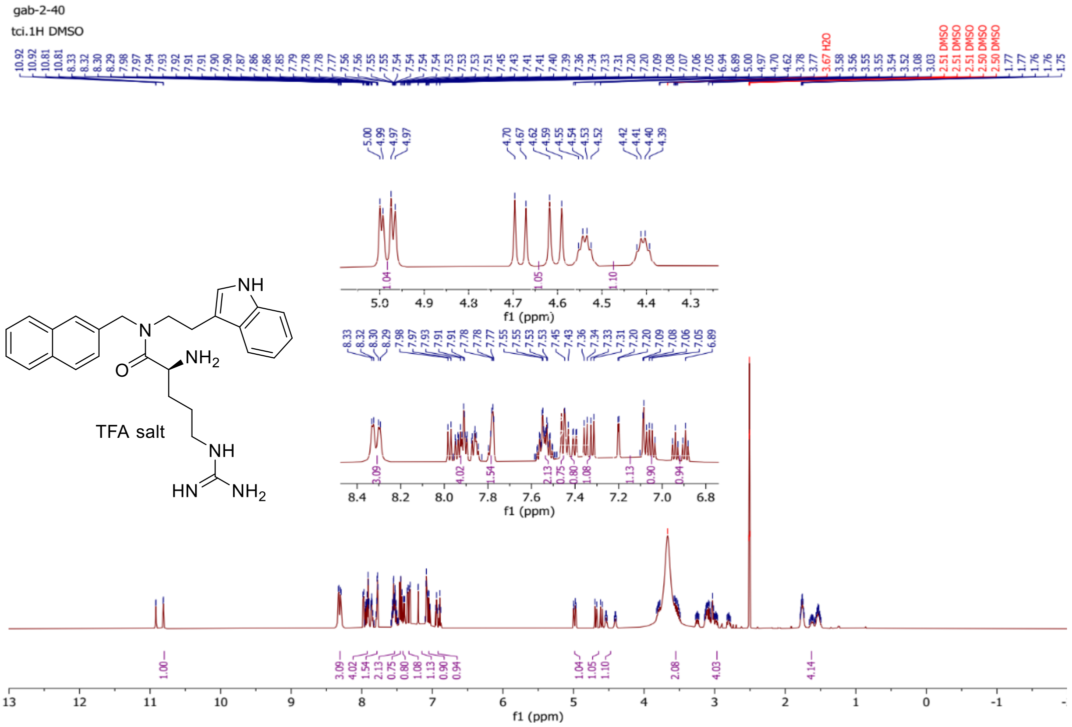

*126. Compound **23** -^{13}CNMR*

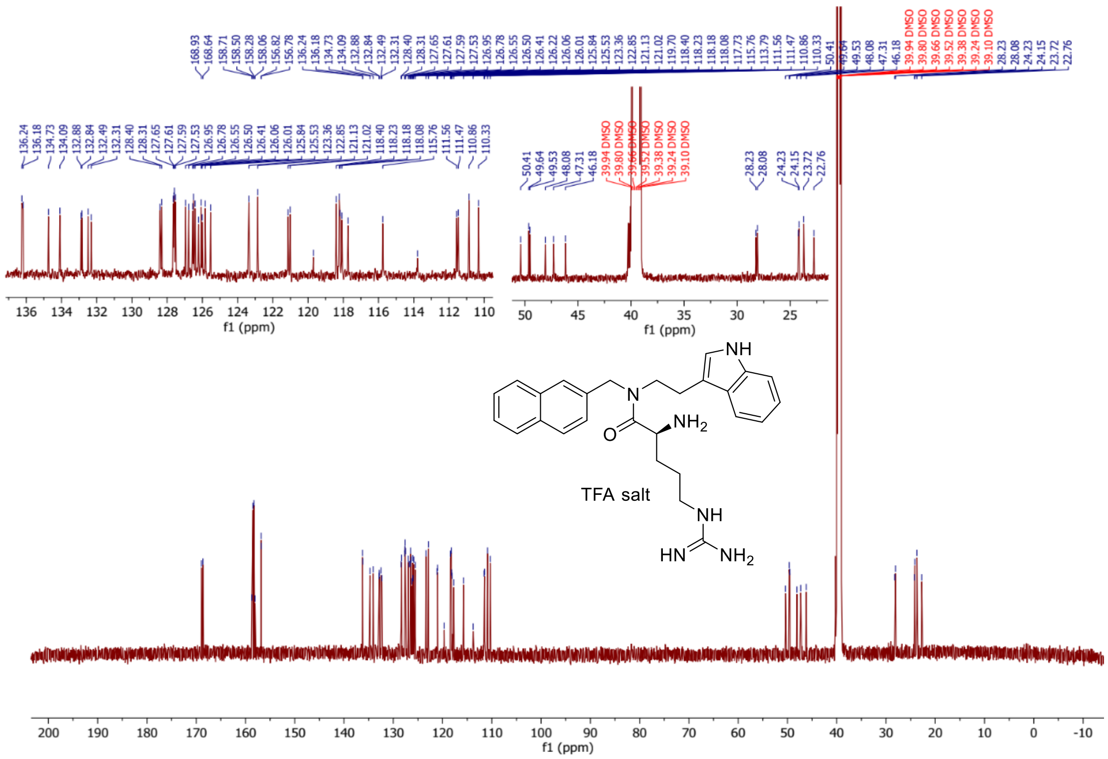

*127. Compound **23** -^{13}CNMR-dept 135*

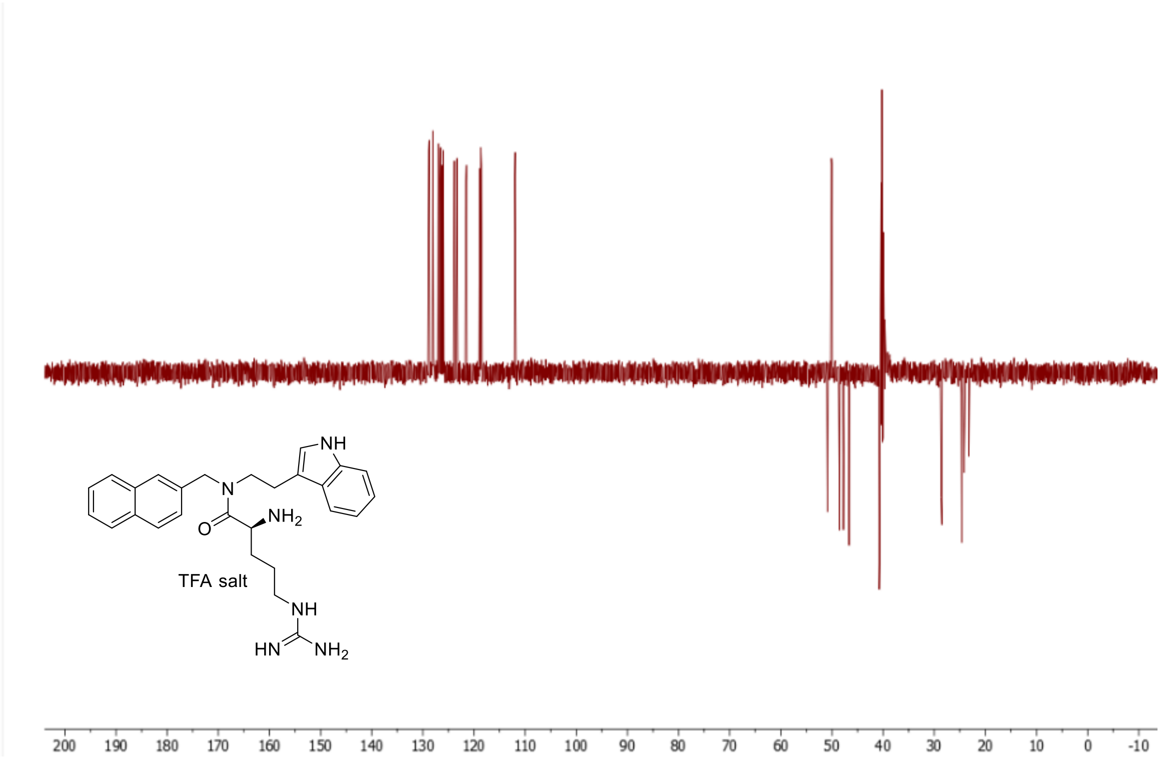

*128. Compound **23** -HSQC*

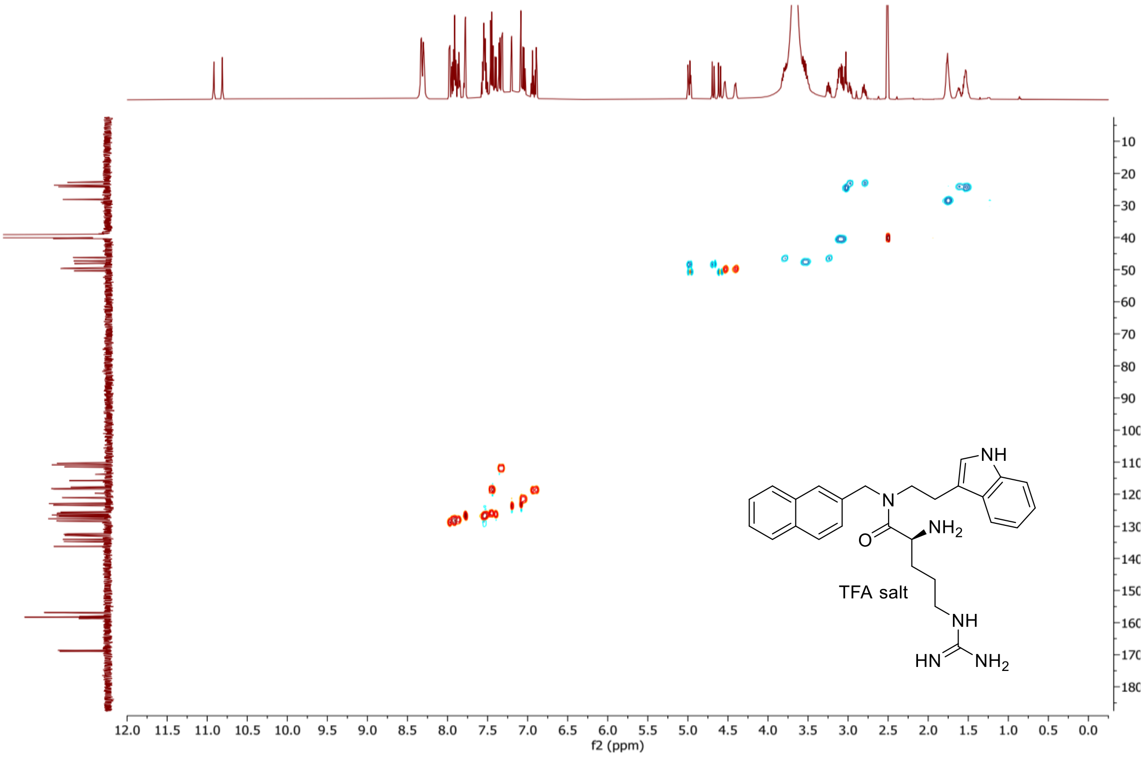

12- ^{1}H, and ^{13}CNMR of dimethyl- naphthyl-indole and naphthyl-phenyl based peptoids.

*129. Compound **14a** -^{1}HNMR*

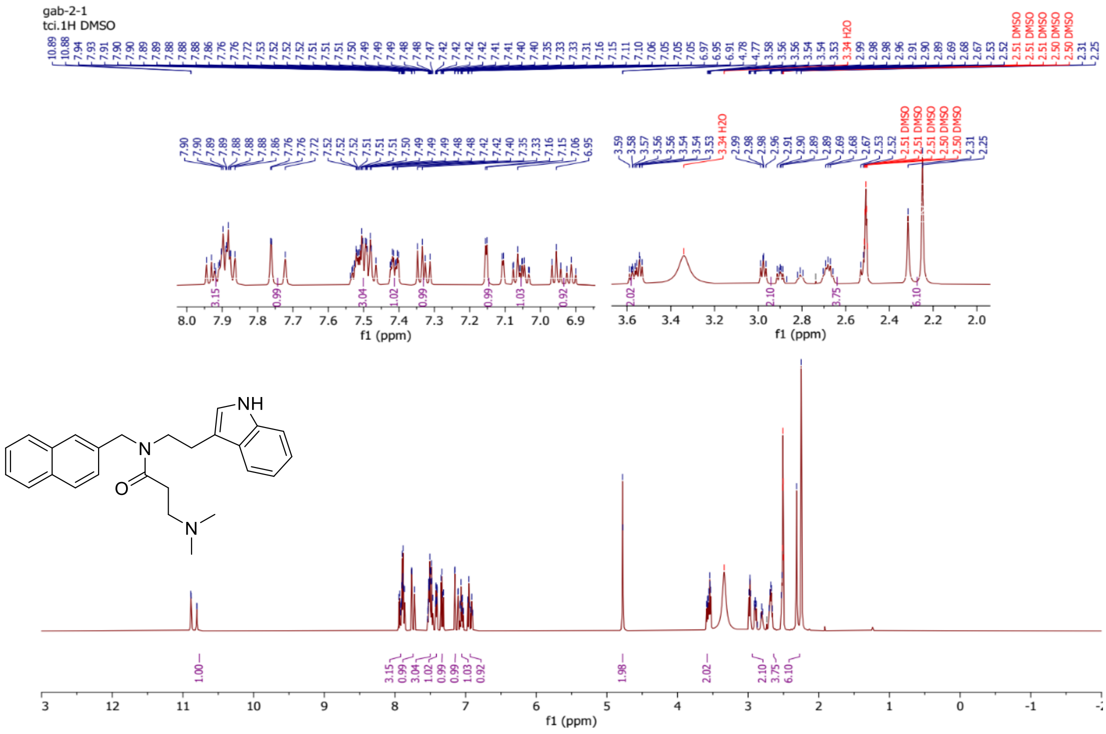

*130. Compound **14a** -^{13}CNMR*

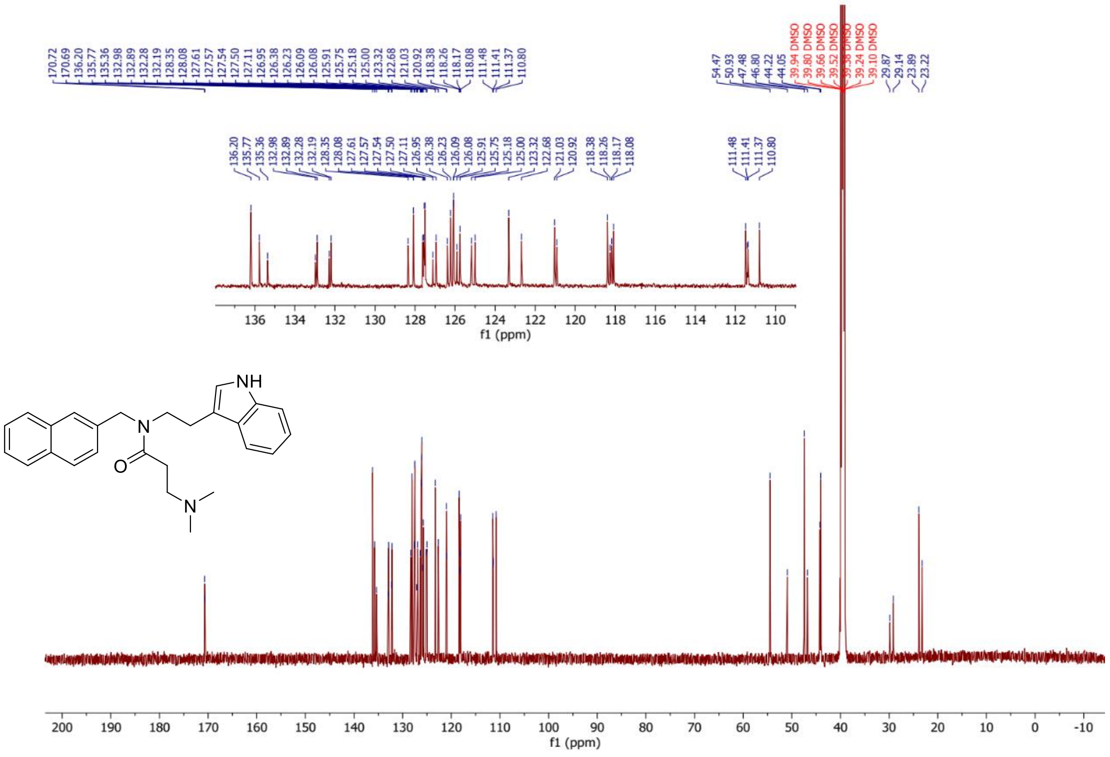

*131. Compound **14a** -^{13}CNMR-dept 135*

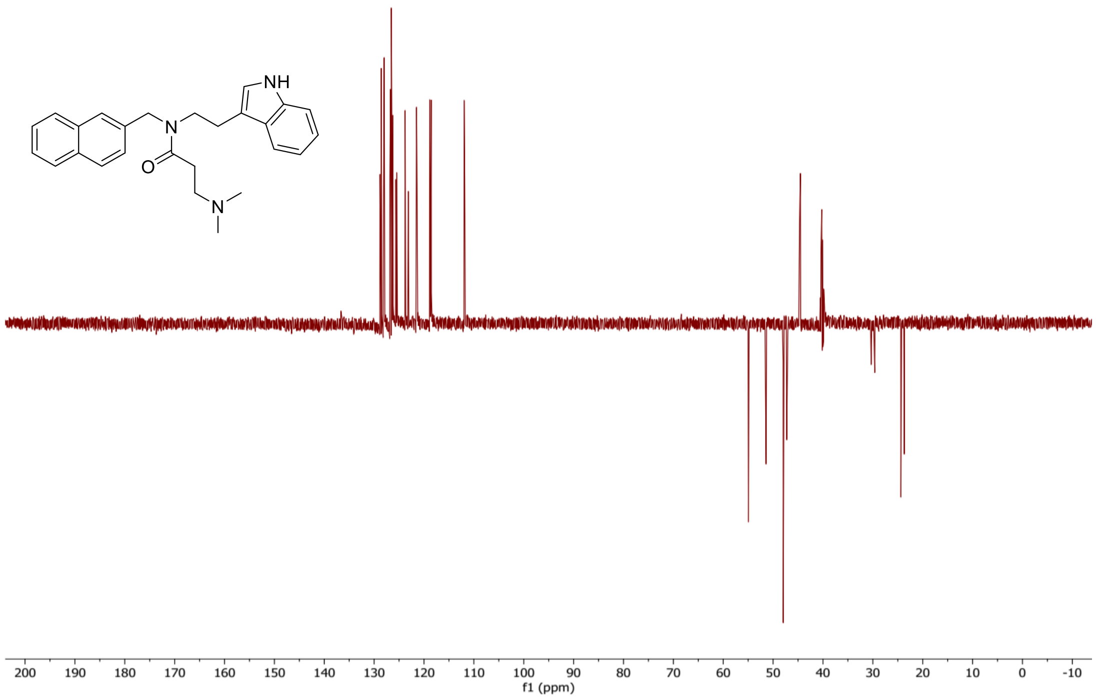

*132. Compound **14a** - HSQC*

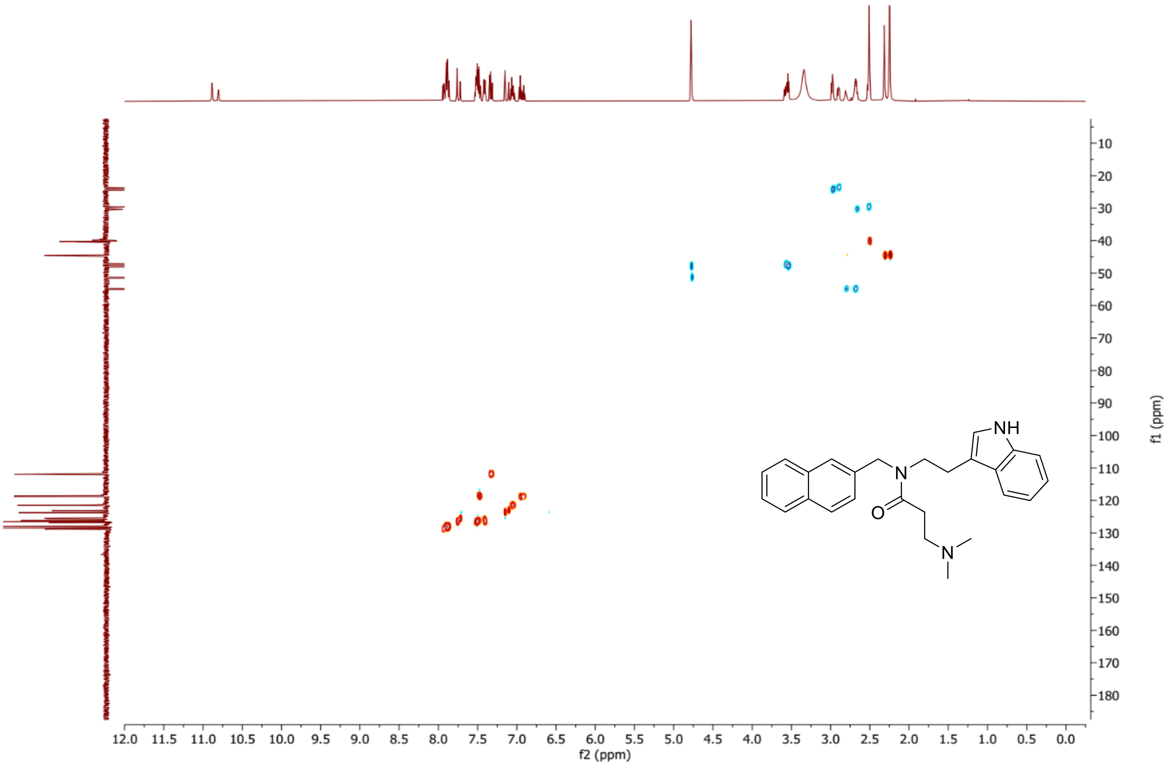

*133. Compound **14b** -^{1}HNMR*

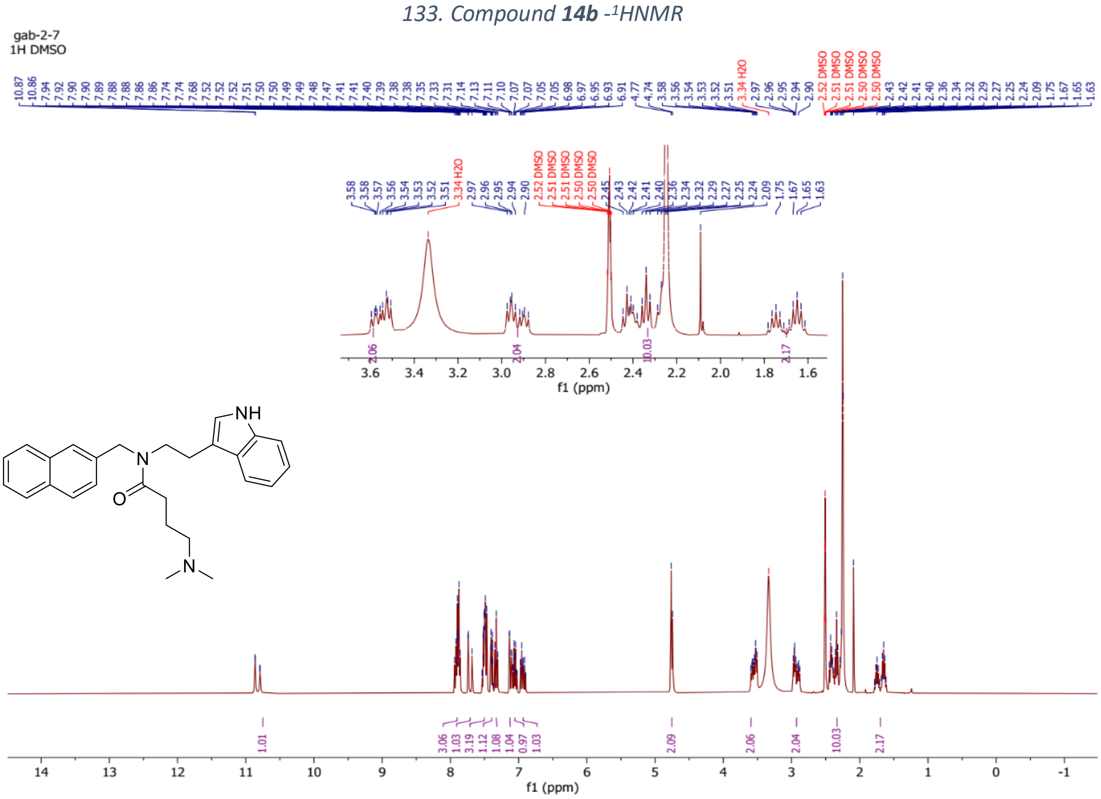

*134. Compound **14b** -^{13}CNMR*

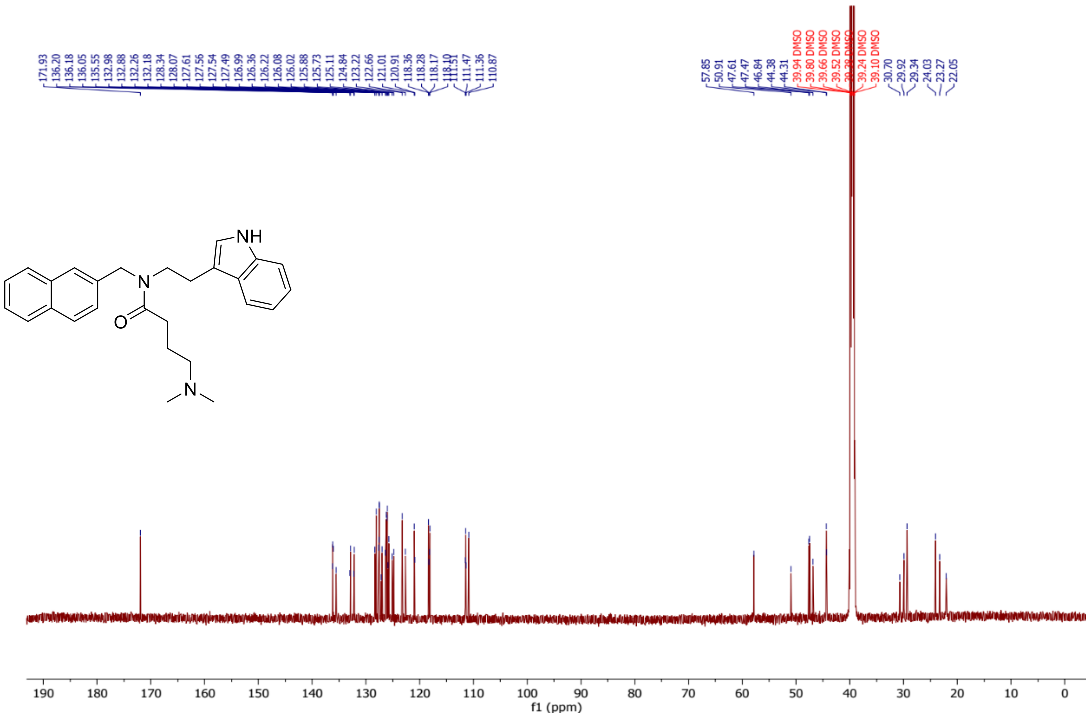

*135. Compound **14b** -^{13}CNMR-dept 135*

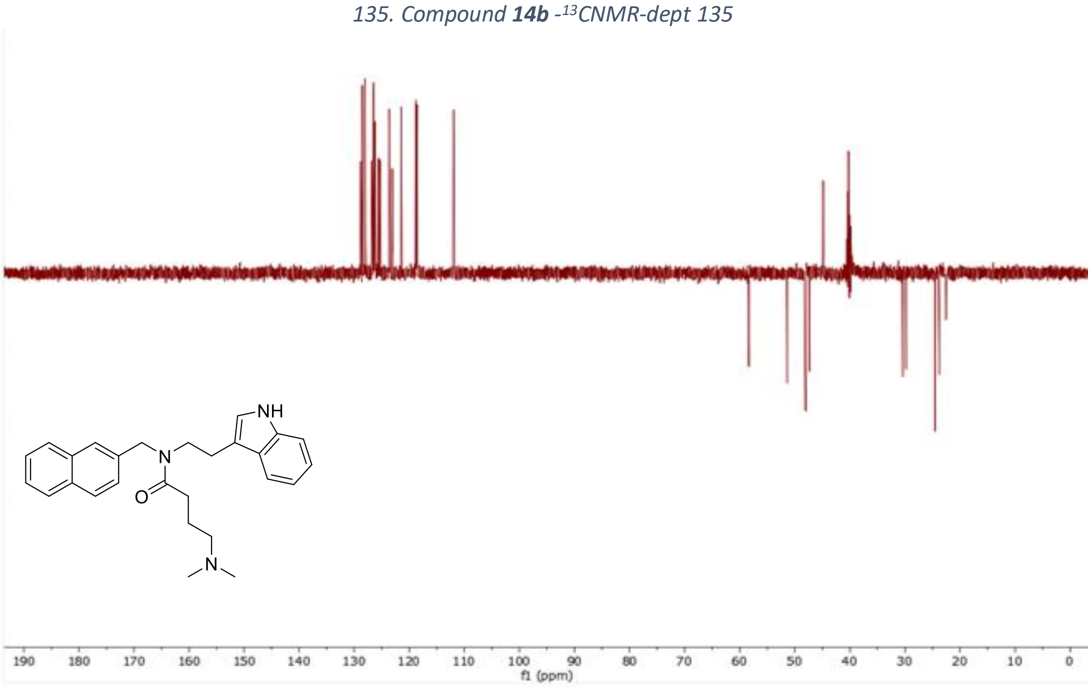

*136. Compound **14b** - HSQC*

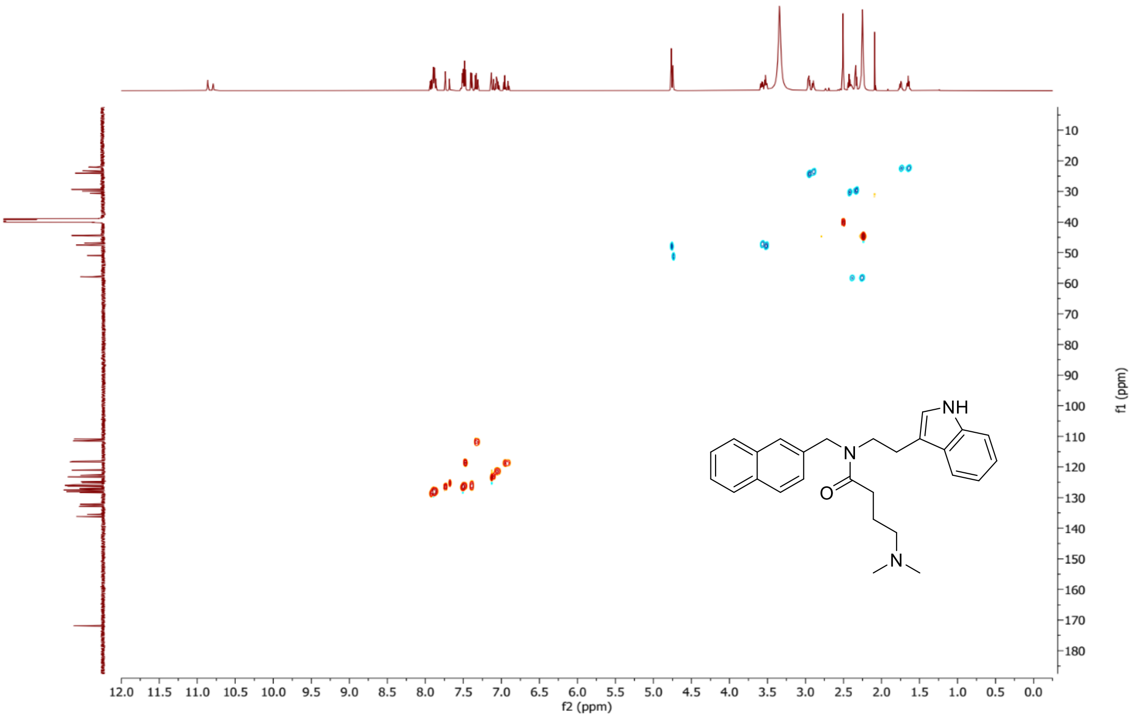

*137. Compound **14c** -^{1}HNMR*

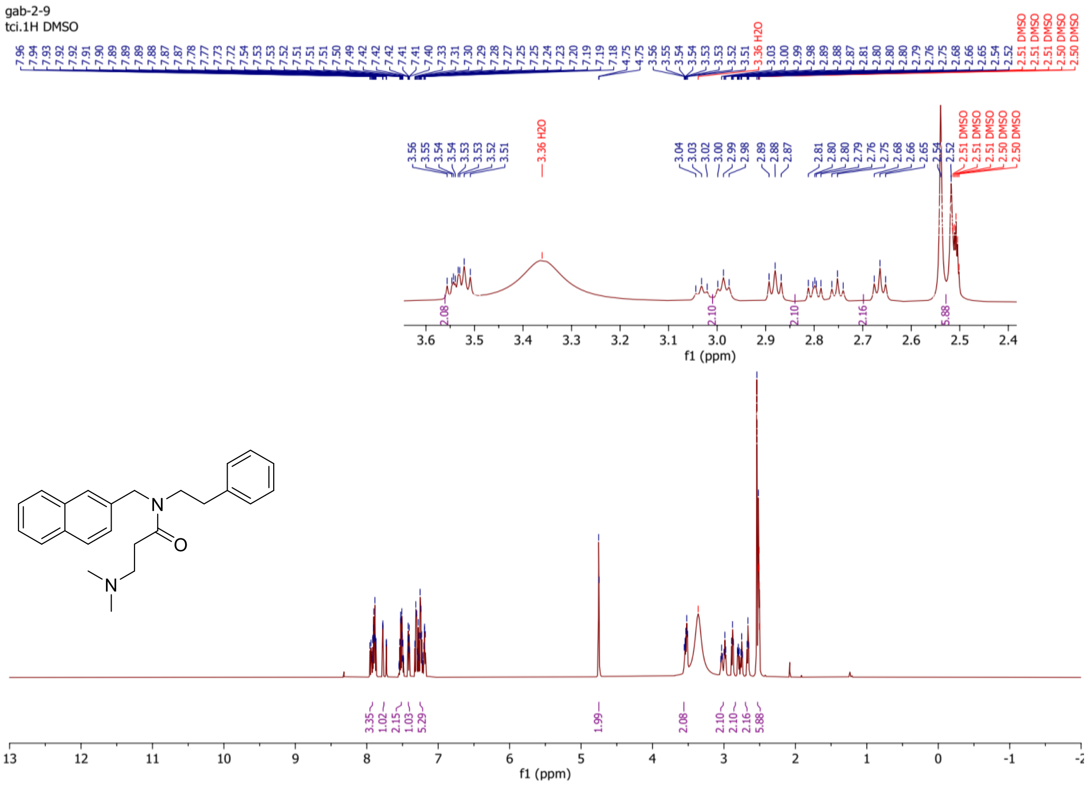

*138. Compound **14c** -^{13}CNMR*

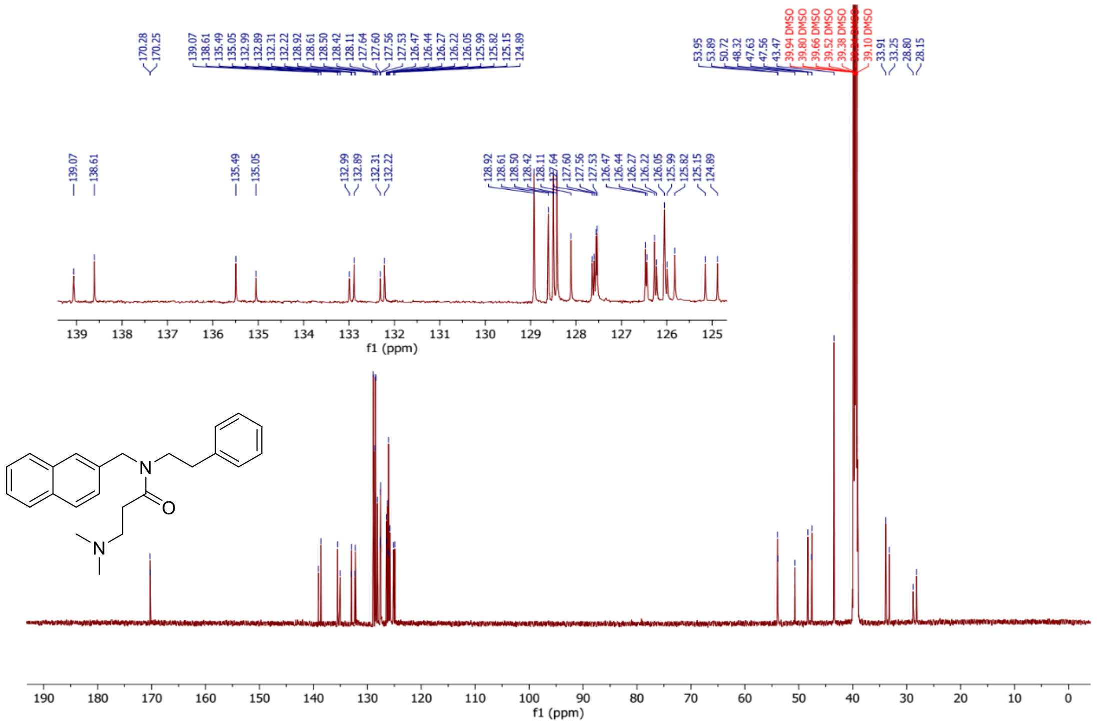

*139. Compound **14c** -¹³CNMR-dept 135*

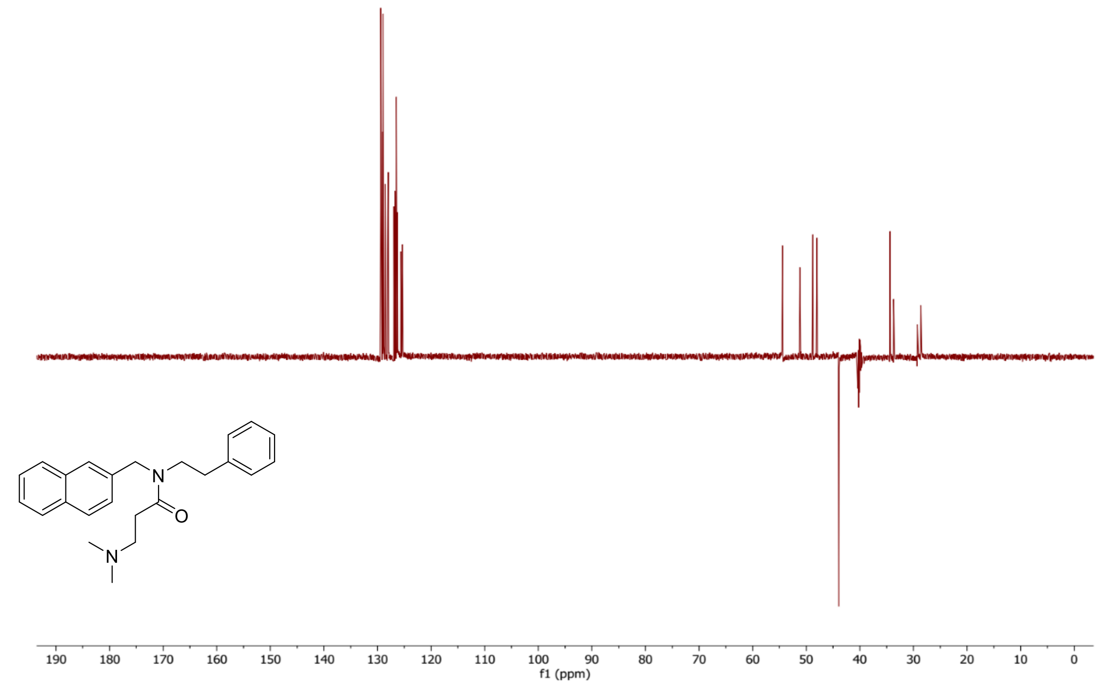

*140. Compound **14c** -HSQC*

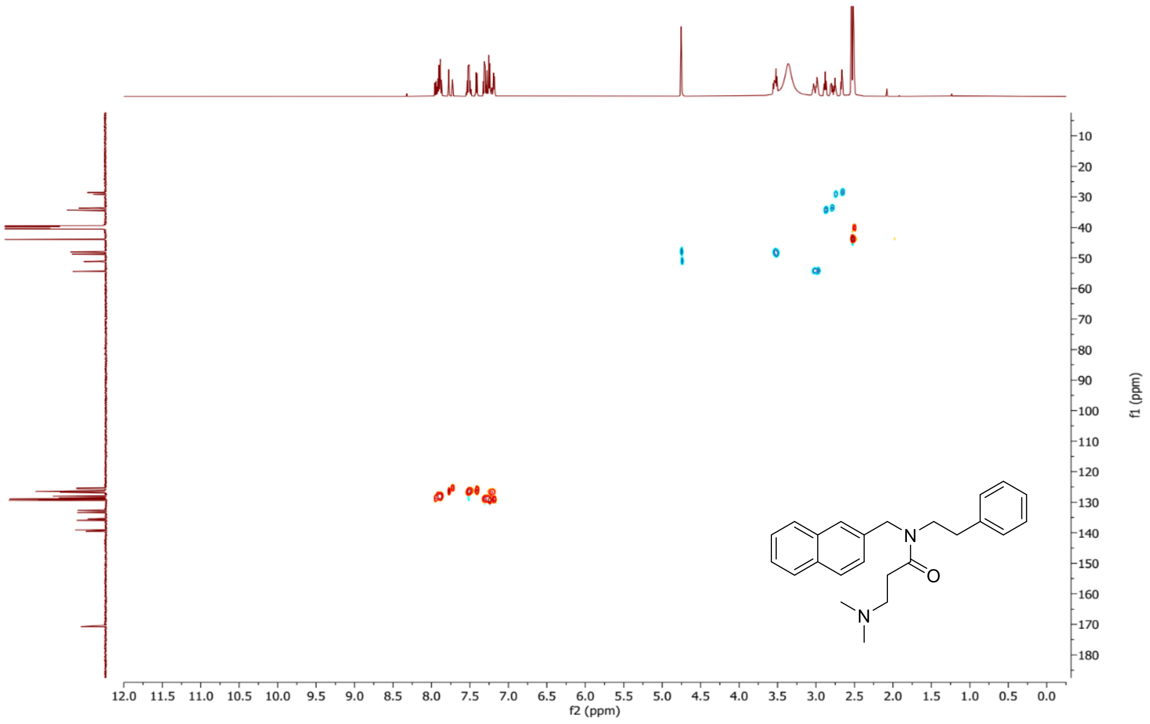

*141. Compound **14d** -^{1}HNMR*

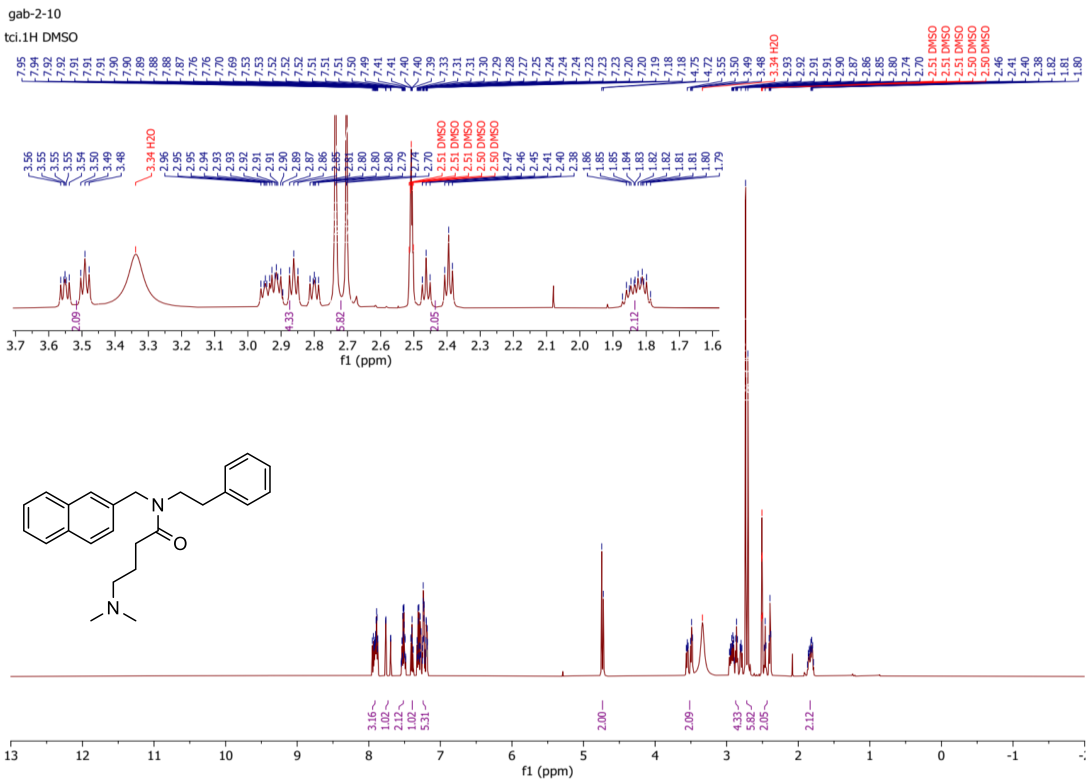

*142. Compound **14d** -^{13}CNMR*

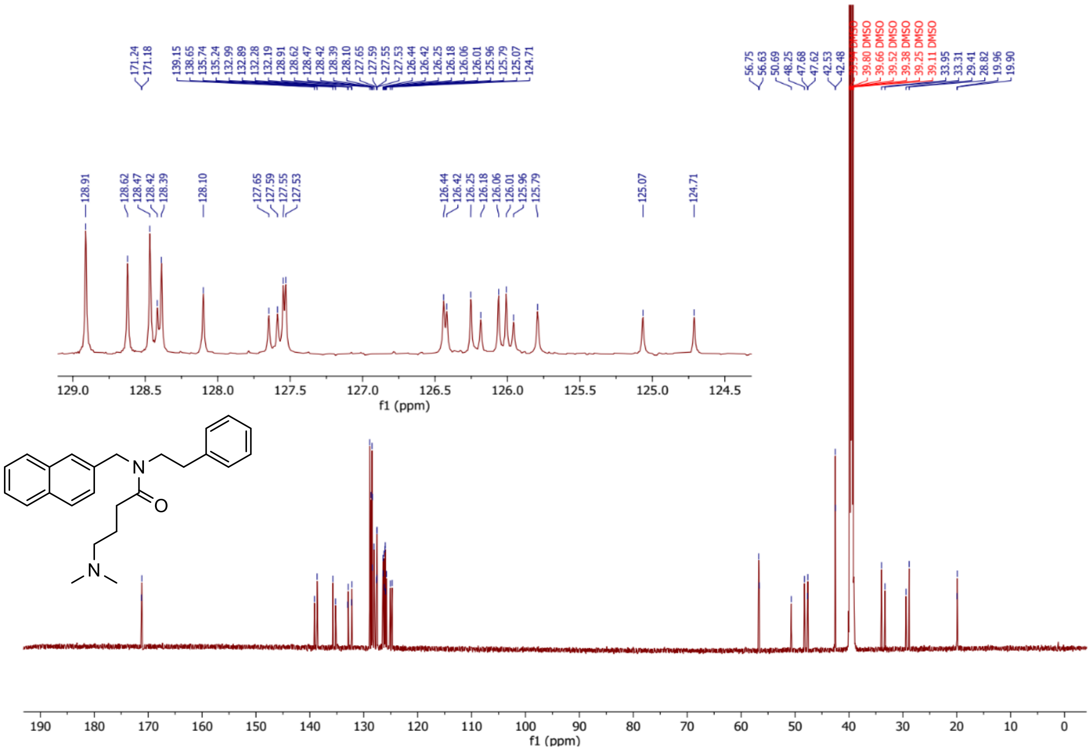

*143. Compound **14d** -^{13}CNMR-dept 135*

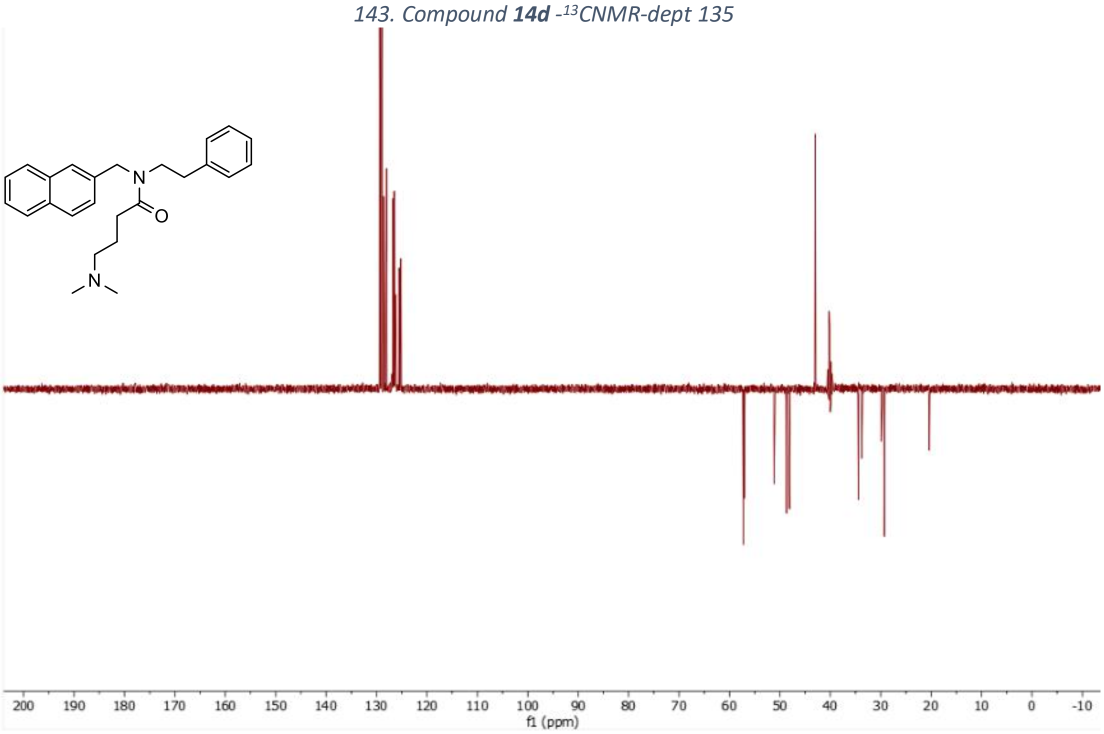

*144. Compound **14d** -HSQC*

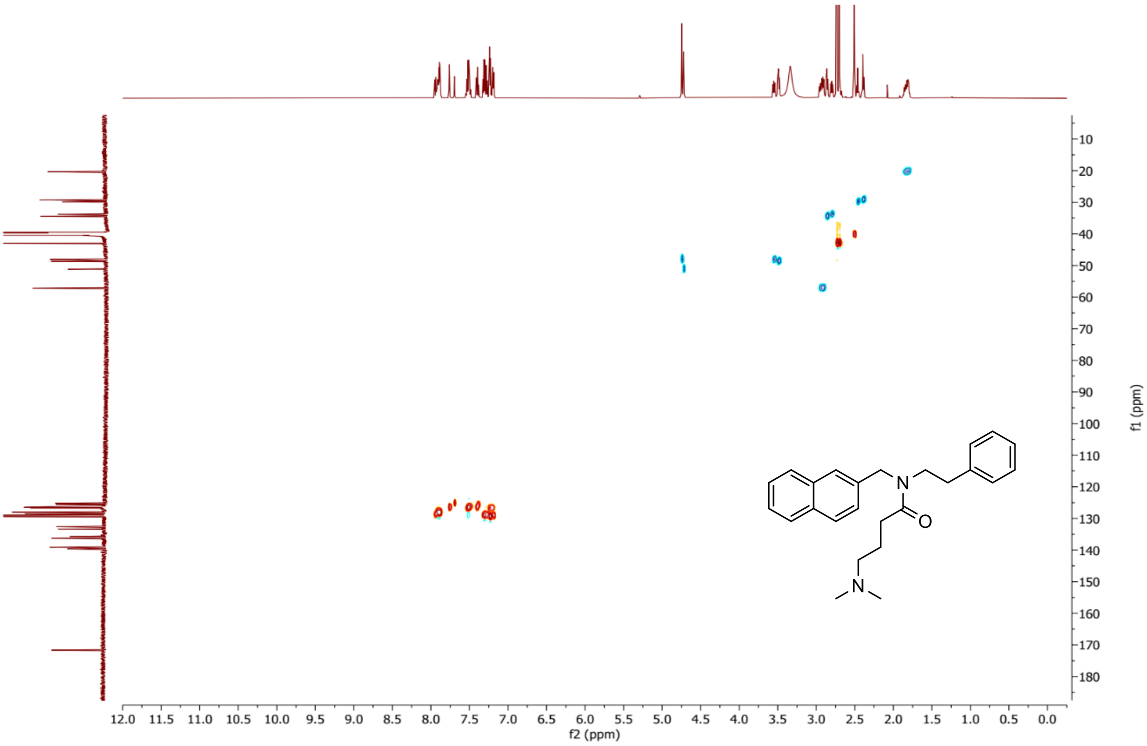

13- ^{1}H, and ^{13}CNMR of tertiary ammonium hydrochloride salts peptoids.

*145. Compound **15a**-^{1}HNMR*

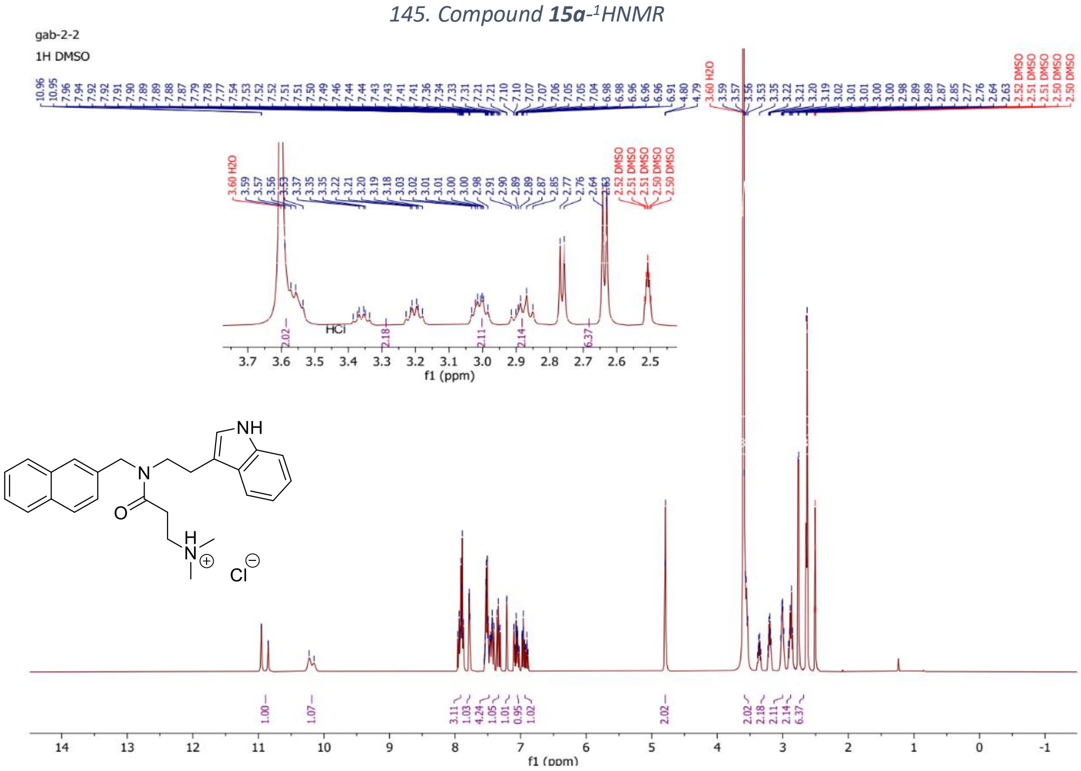

*146. Compound **15a** -^{13}CNMR*

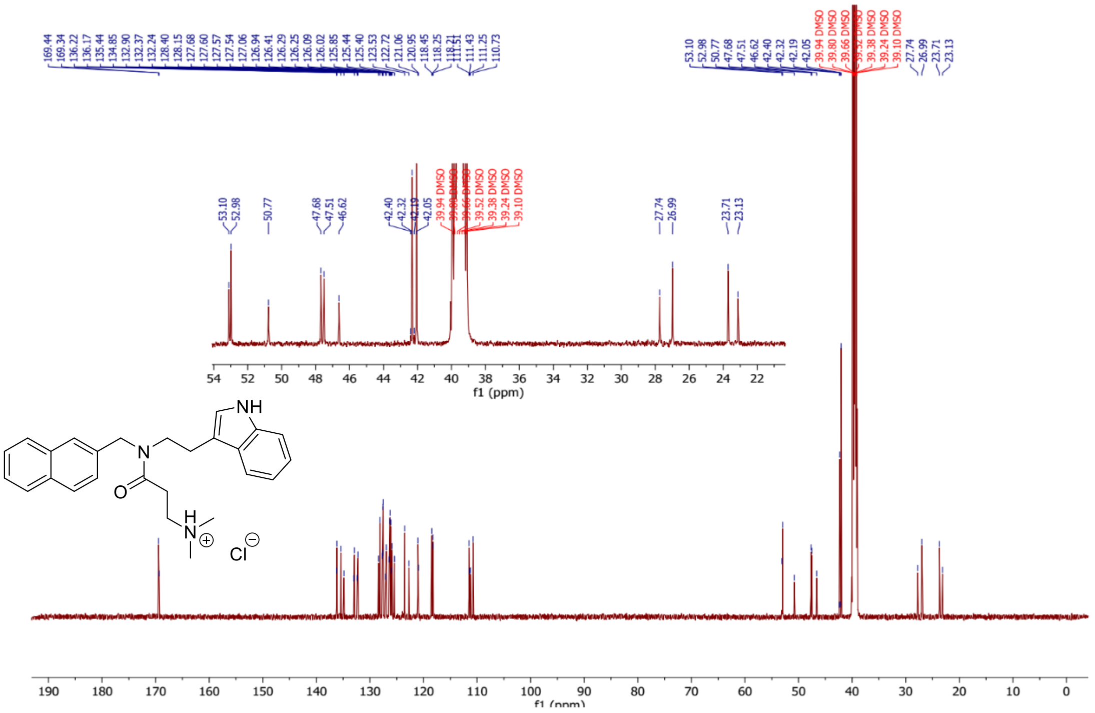

147. Compound **15a** -^{13}CNMR-dept 135

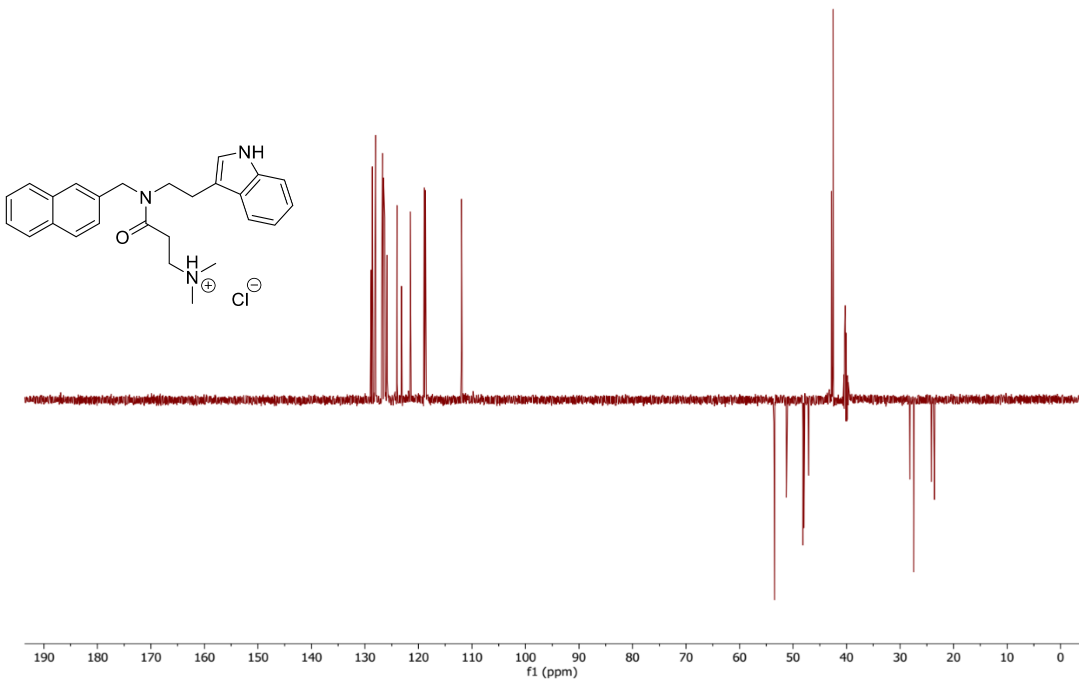

148. Compound **15a** -HSQC

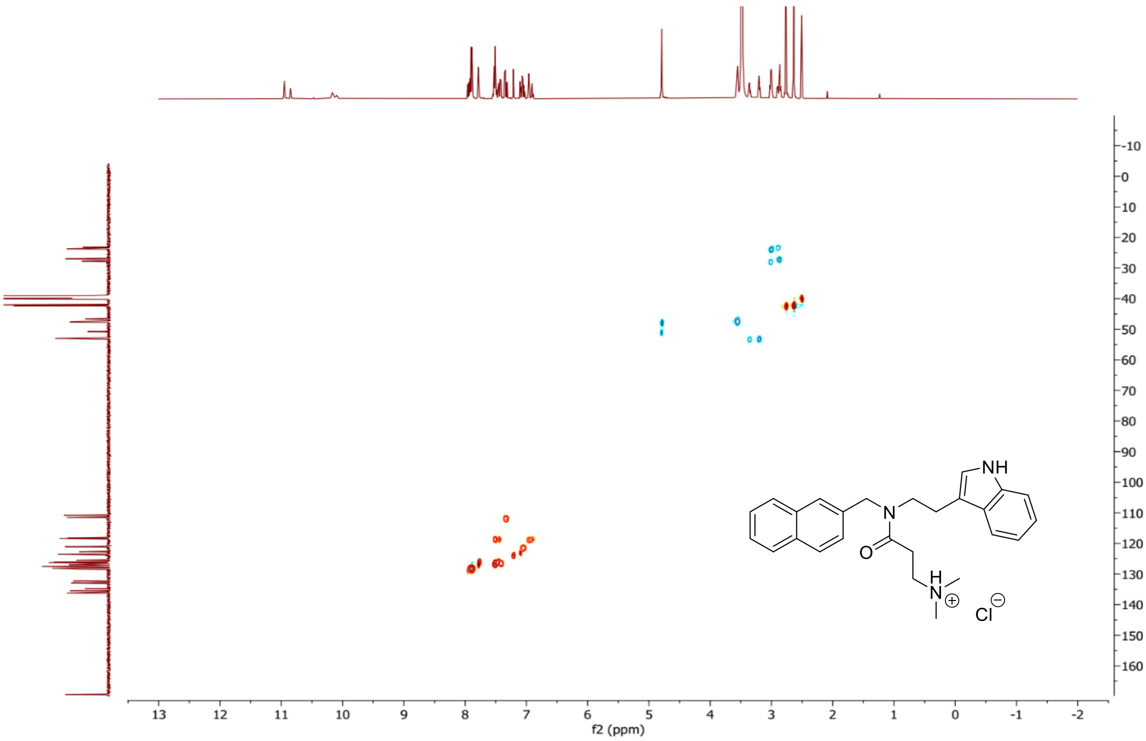

*149. Compound **15b** -^{1}HNMR*

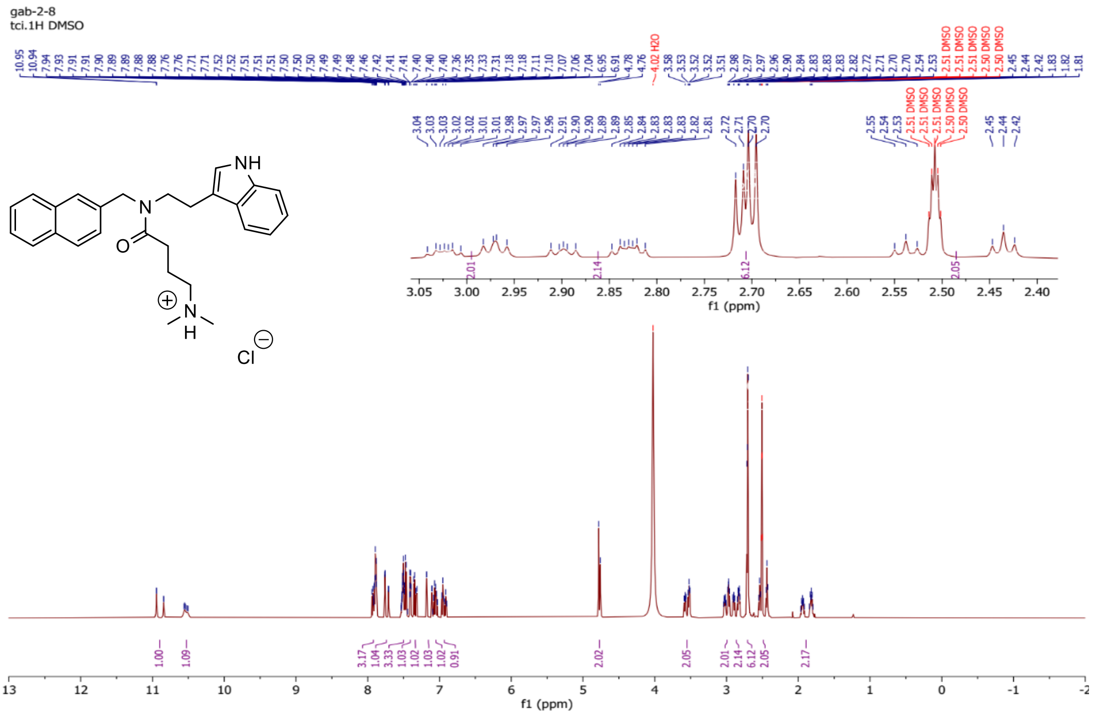

*150. Compound **15b** -^{13}CNMR*

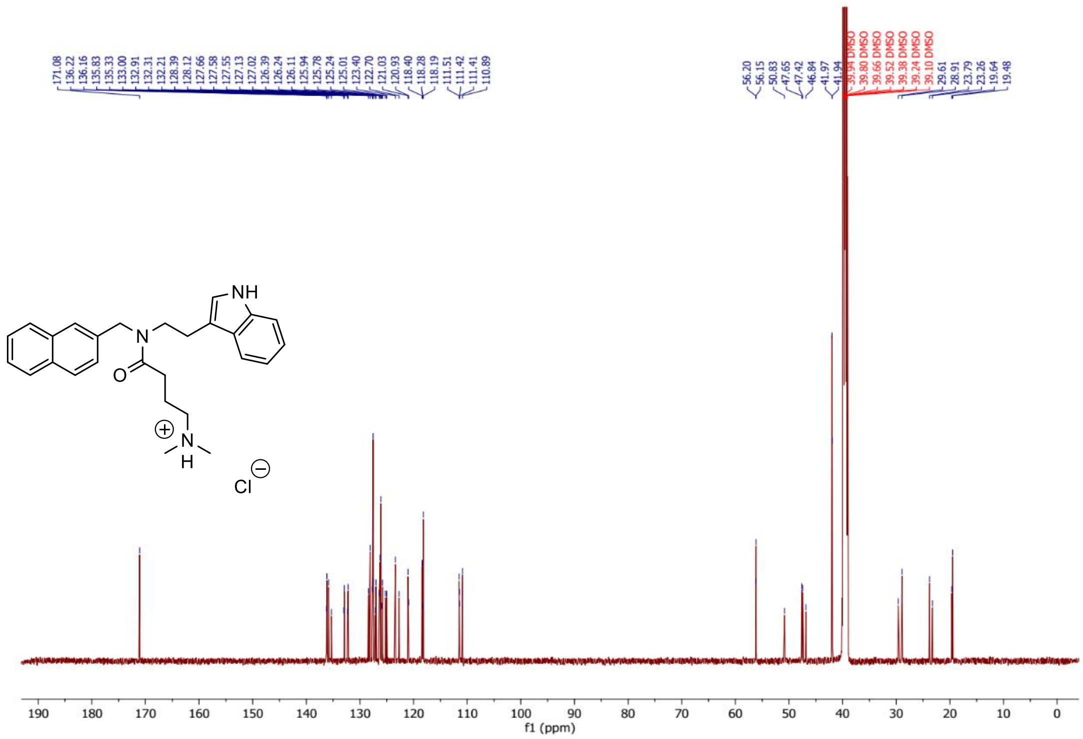

*151. Compound **15b** -13CNMR-dept 135*

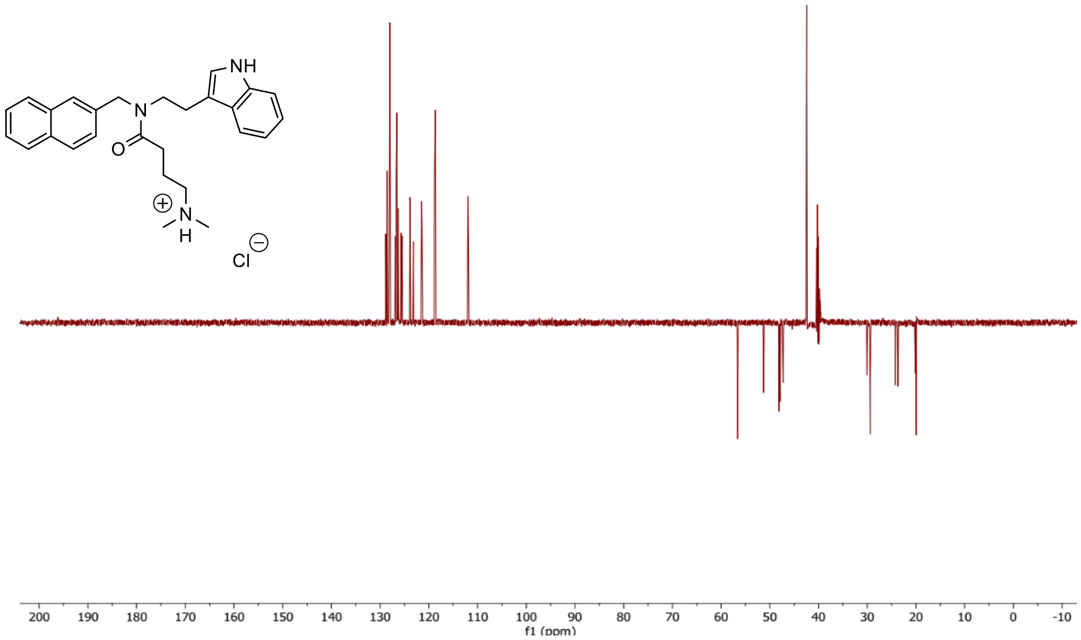

*152. Compound **15b** -HSQC*

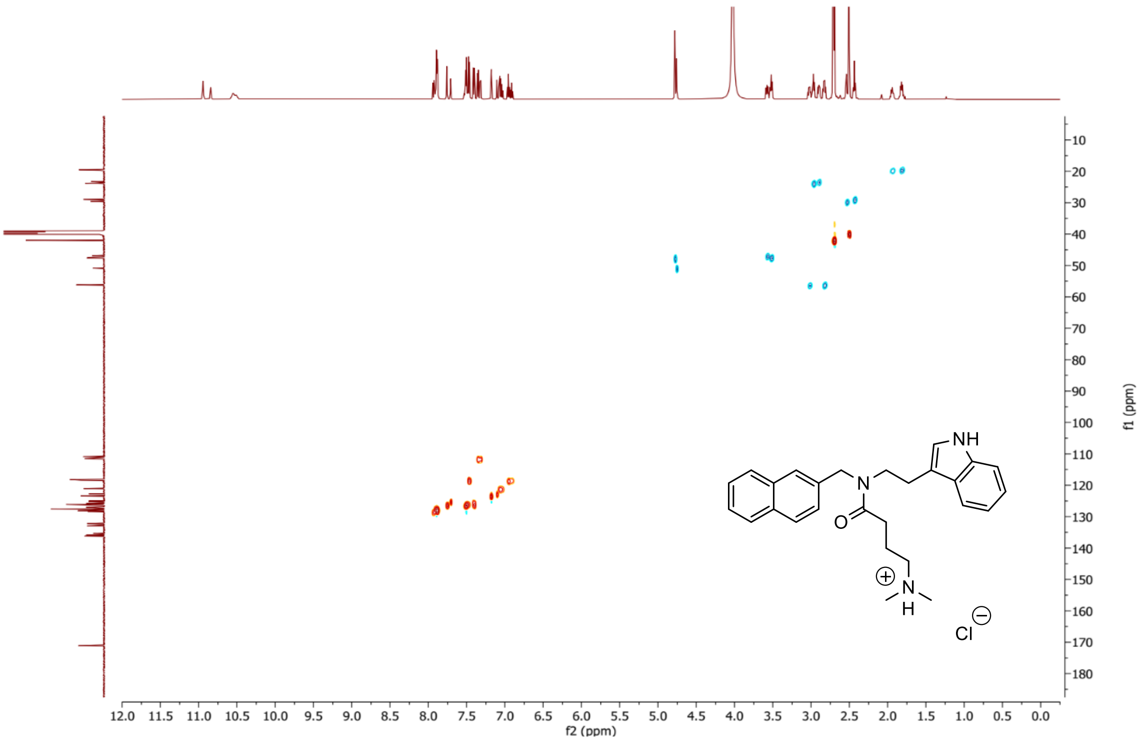

*153. Compound **15c** -^{1}HNMR*

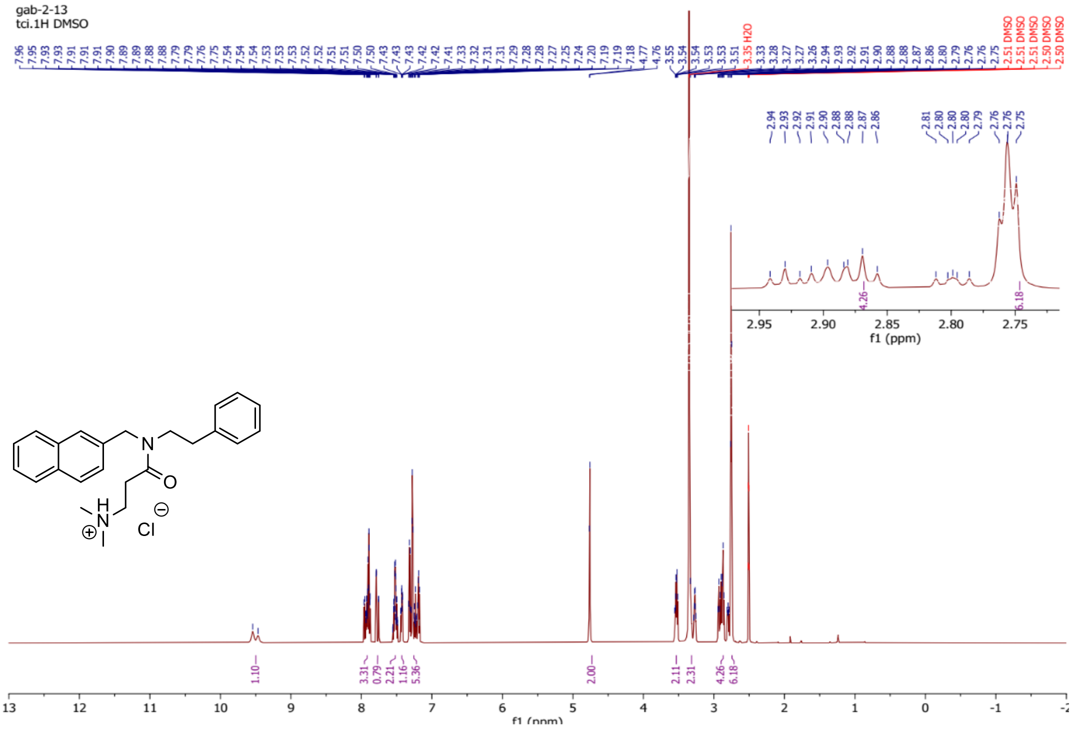

*154. Compound **15c** -^{13}CNMR*

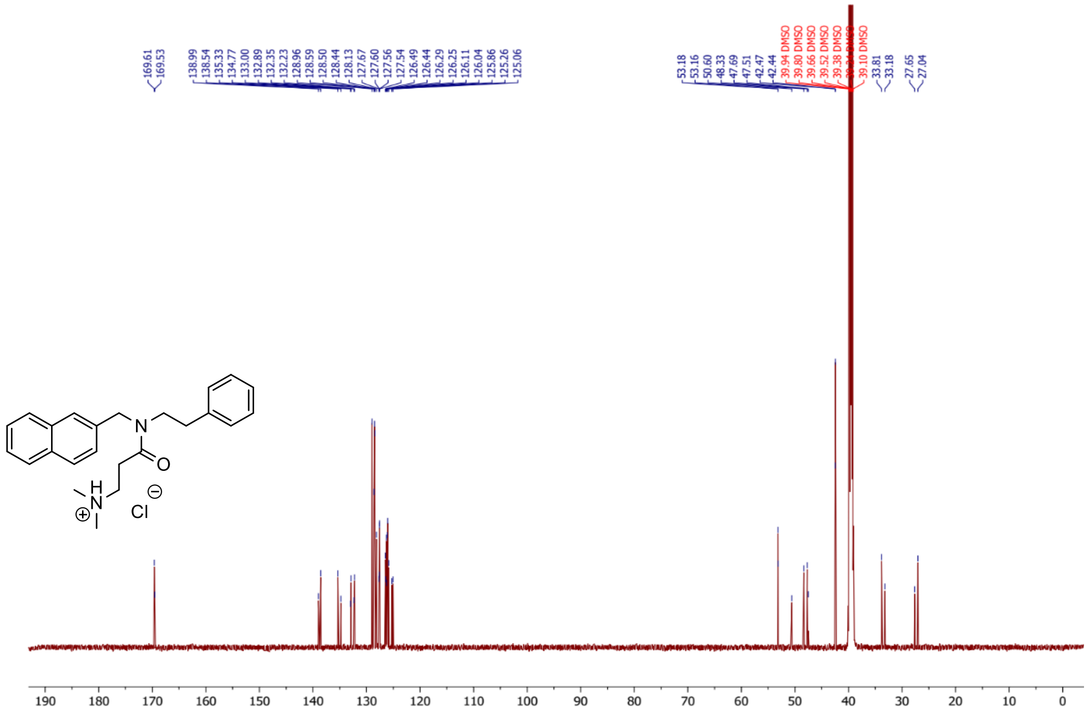

*155. Compound **15c** -^{13}CNMR-dept 135*

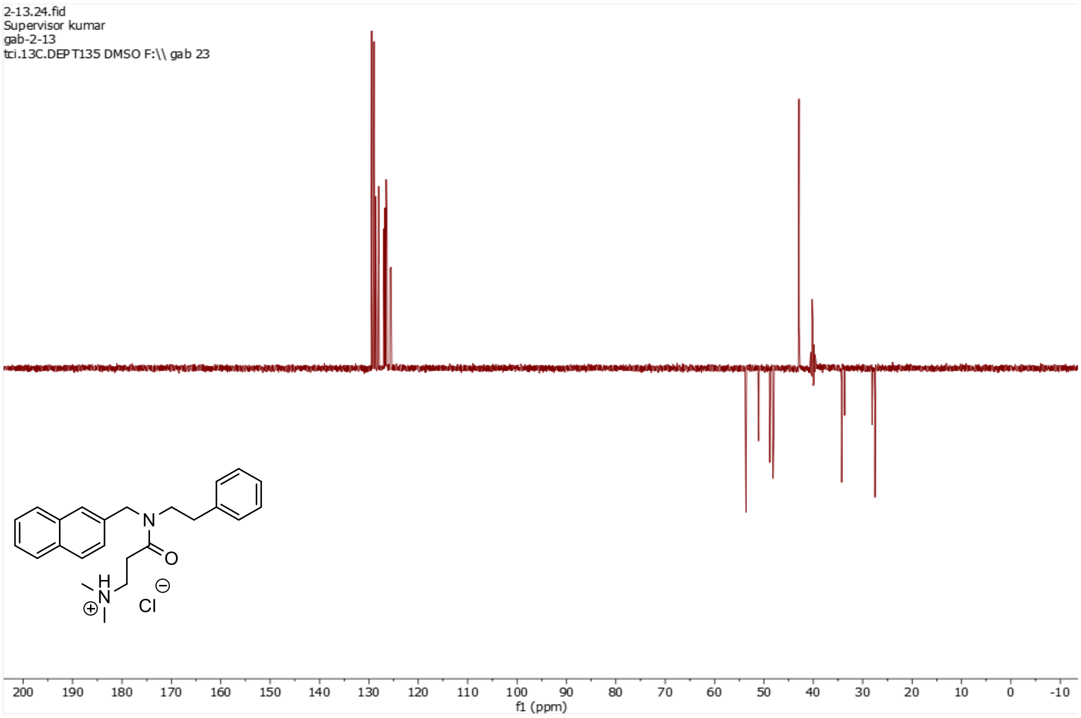

*156. Compound **15c** -HSQC*

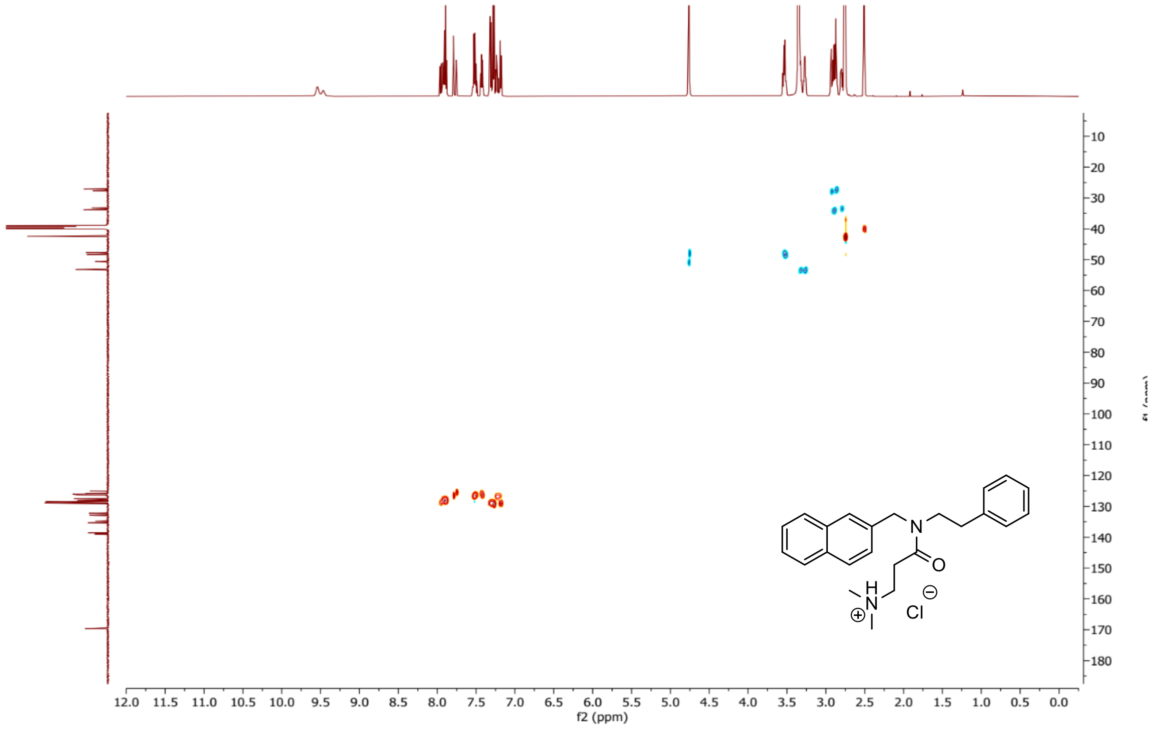

*157. Compound **15d**-^{1}HNMR*

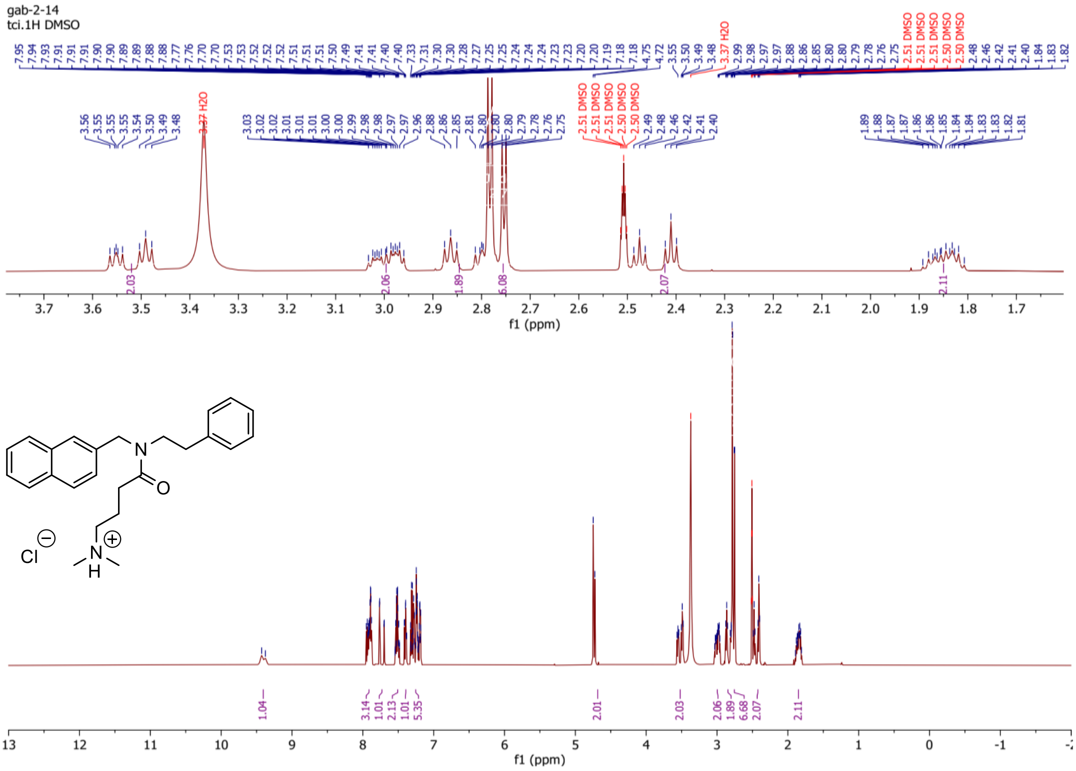

*158. Compound **15d** -^{13}CNMR*

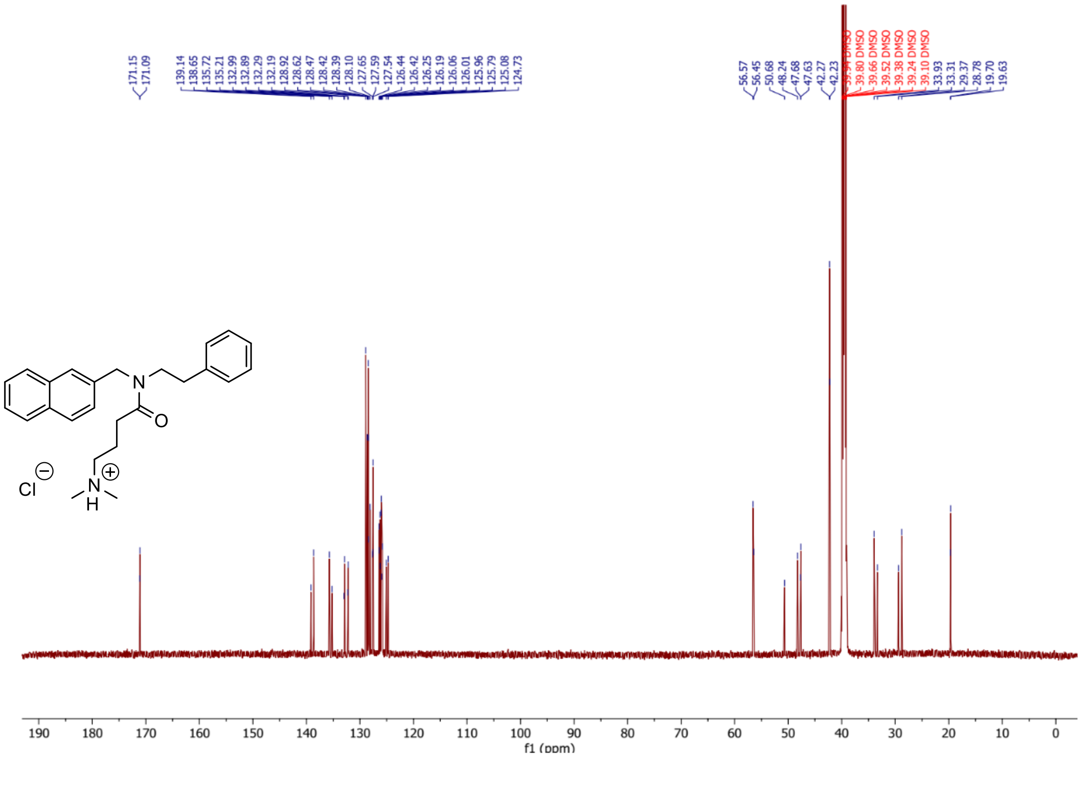

*159. Compound **15d** -^{13}CNMR-dept 135*

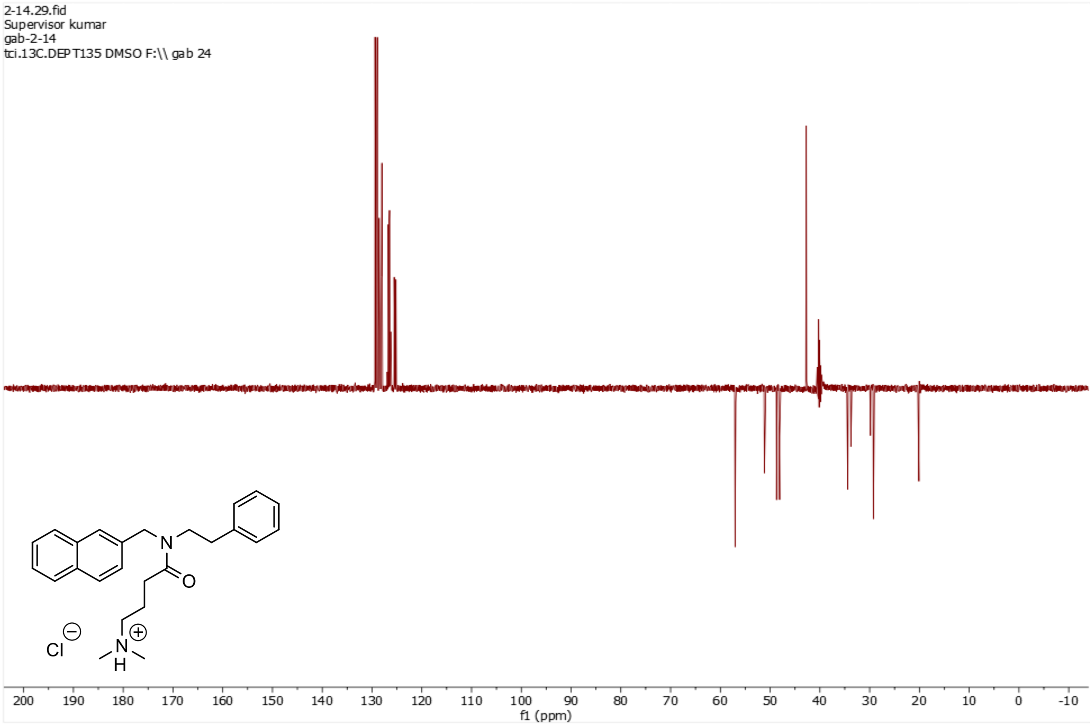

*160. Compound **15d** -HSQC*

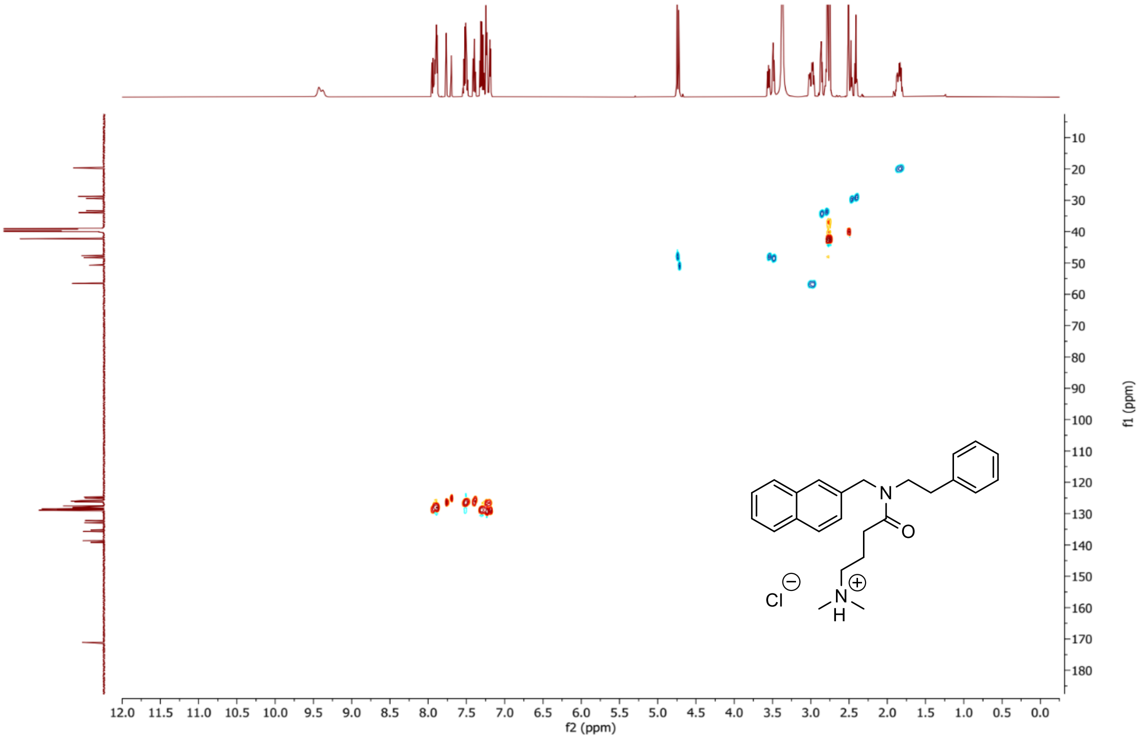

14- ^{1}H, and ^{13}CNMR of quaternary ammonium iodide salts.

*161. Compound **16a** -^{1}HNMR*

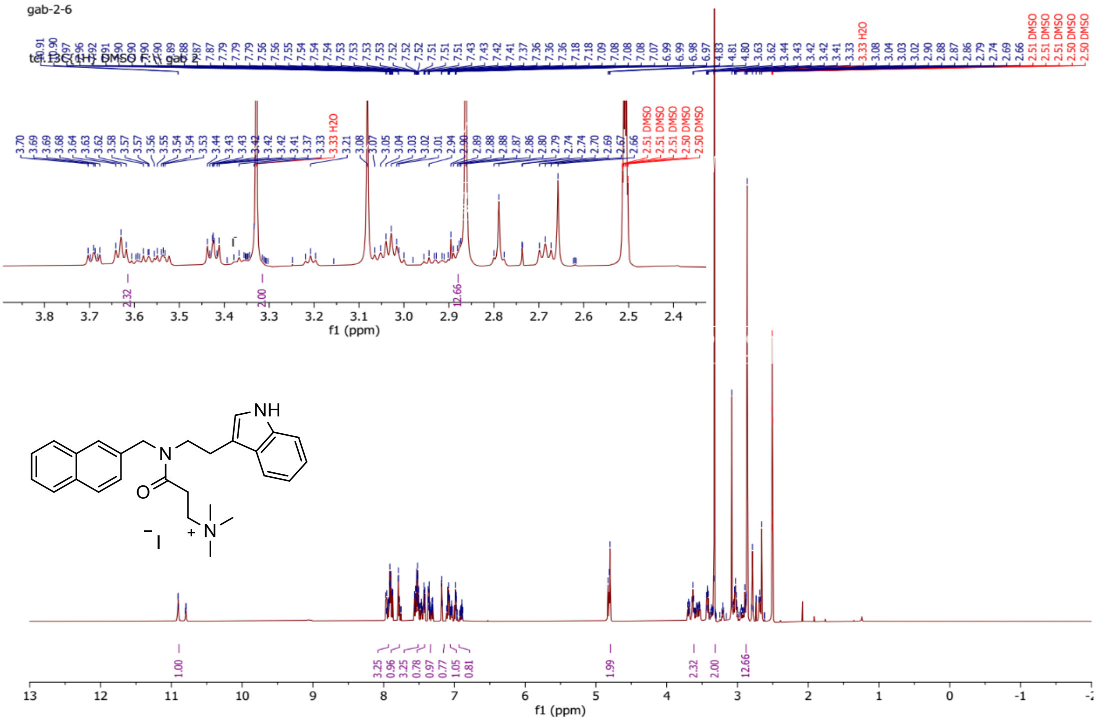

*162. Compound **16a** -^{13}CNMR*

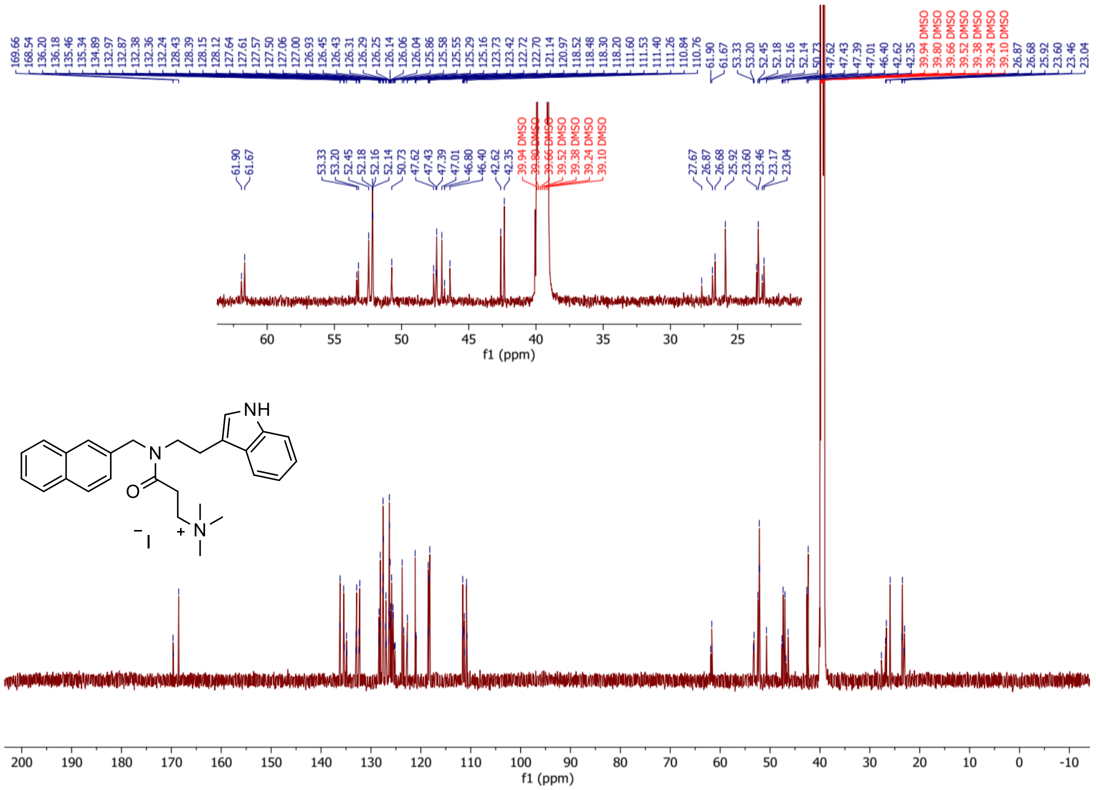

*163. Compound **16a** -^{13}CNMR-dept 135*

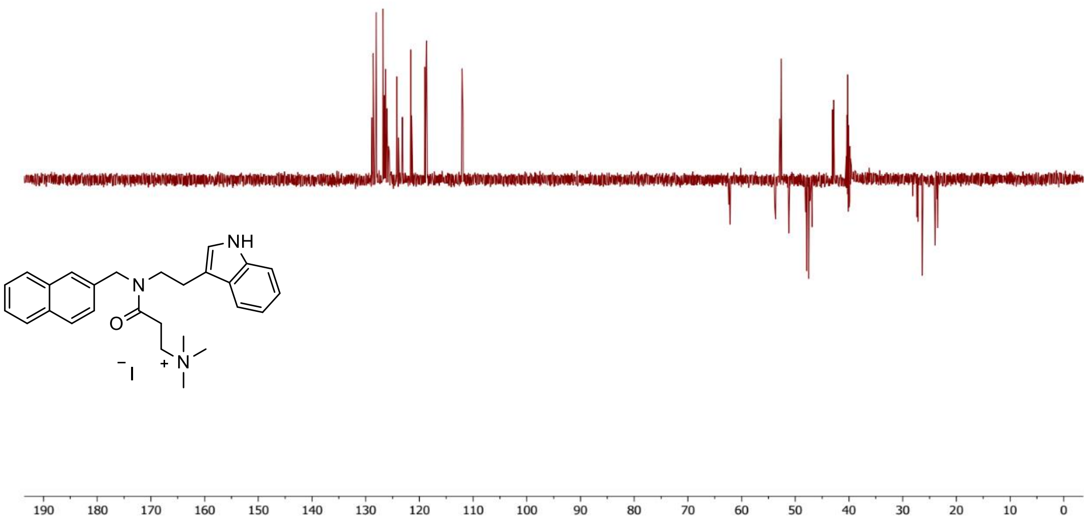

*164. Compound **16a** -HSQC*

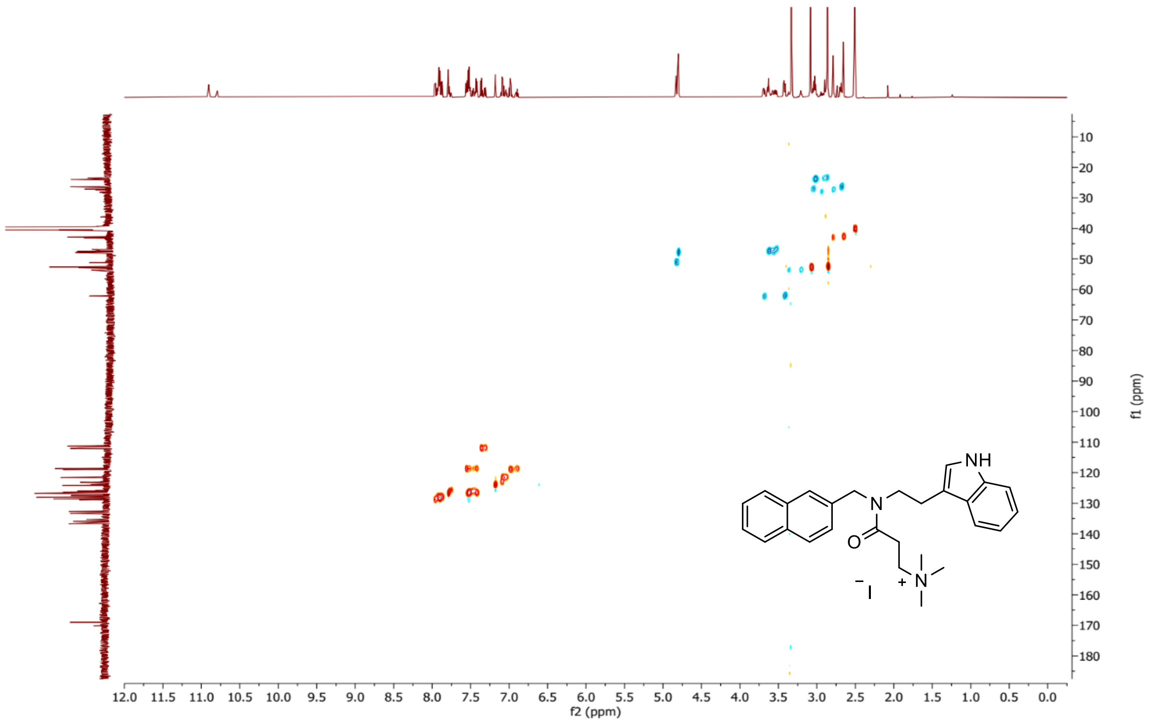

*165. Compound **16b** -^{1}HNMR*

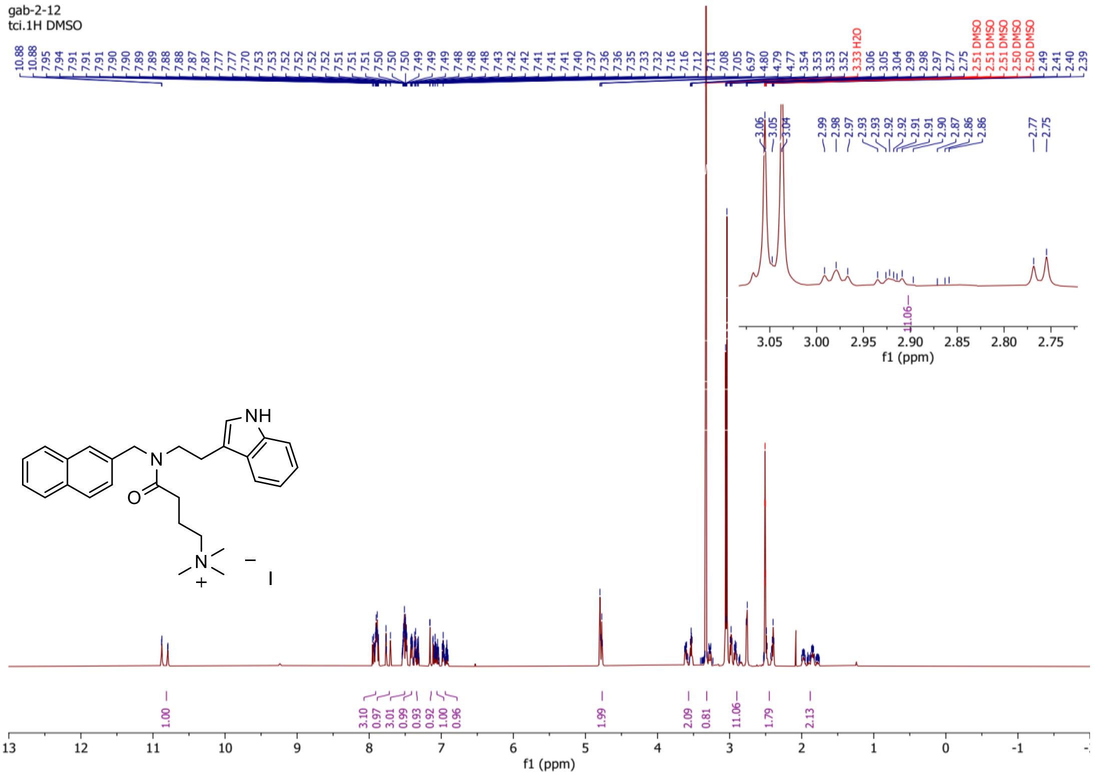

*166. Compound **16b** -^{13}CNMR*

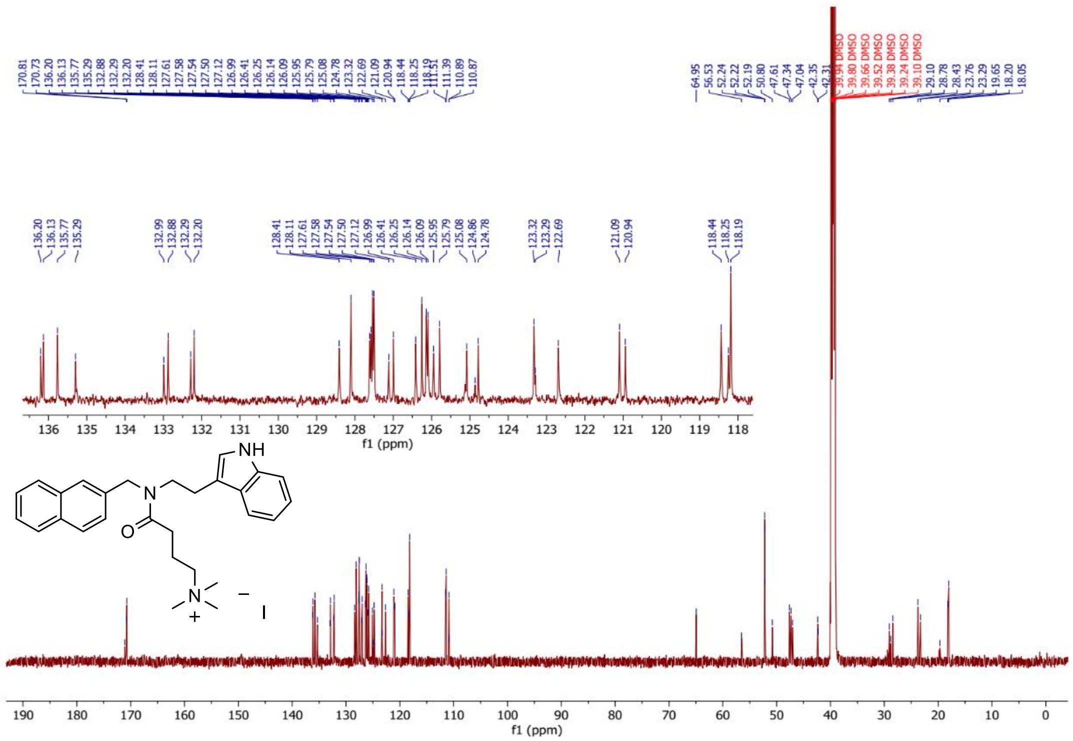

*167. Compound **16b** -^{13}CNMR-dept 135*

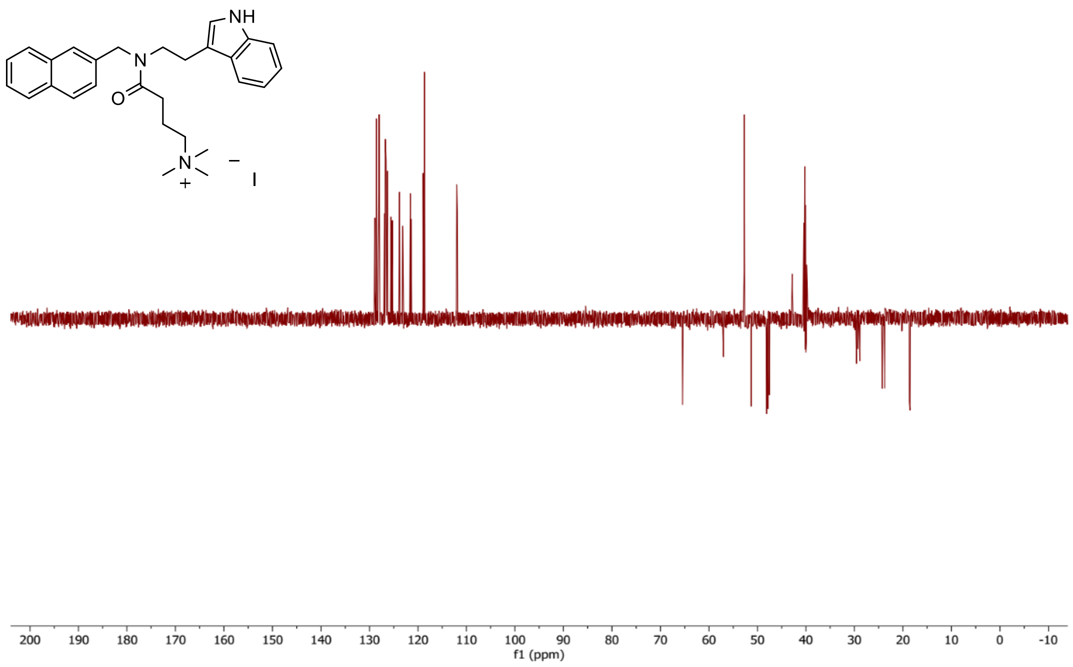

*168. Compound **16b** -HSQC*

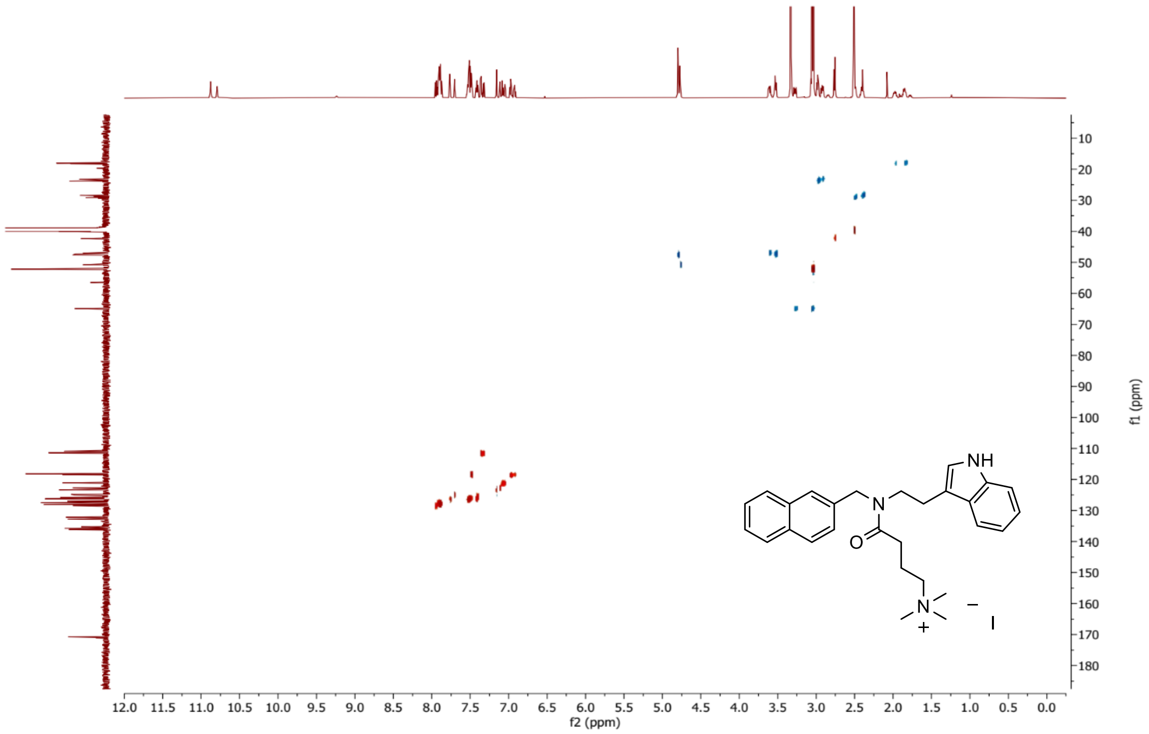

169. *Compound **16c** -^{1}HNMR*

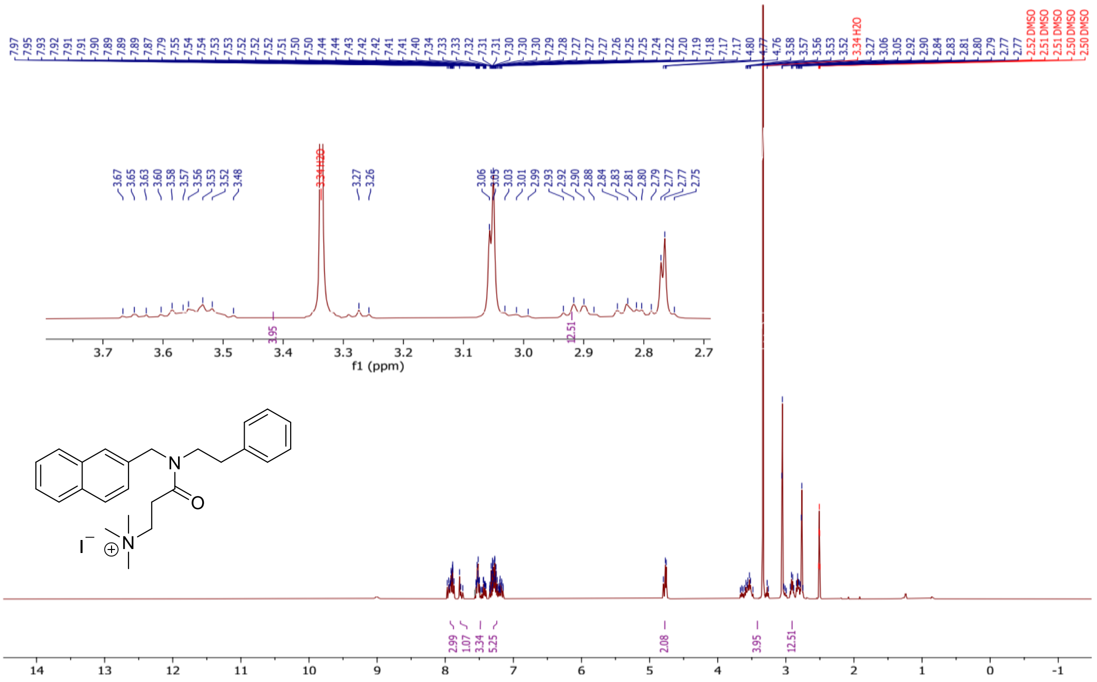

170. *Compound **16c** -^{13}CNMR*

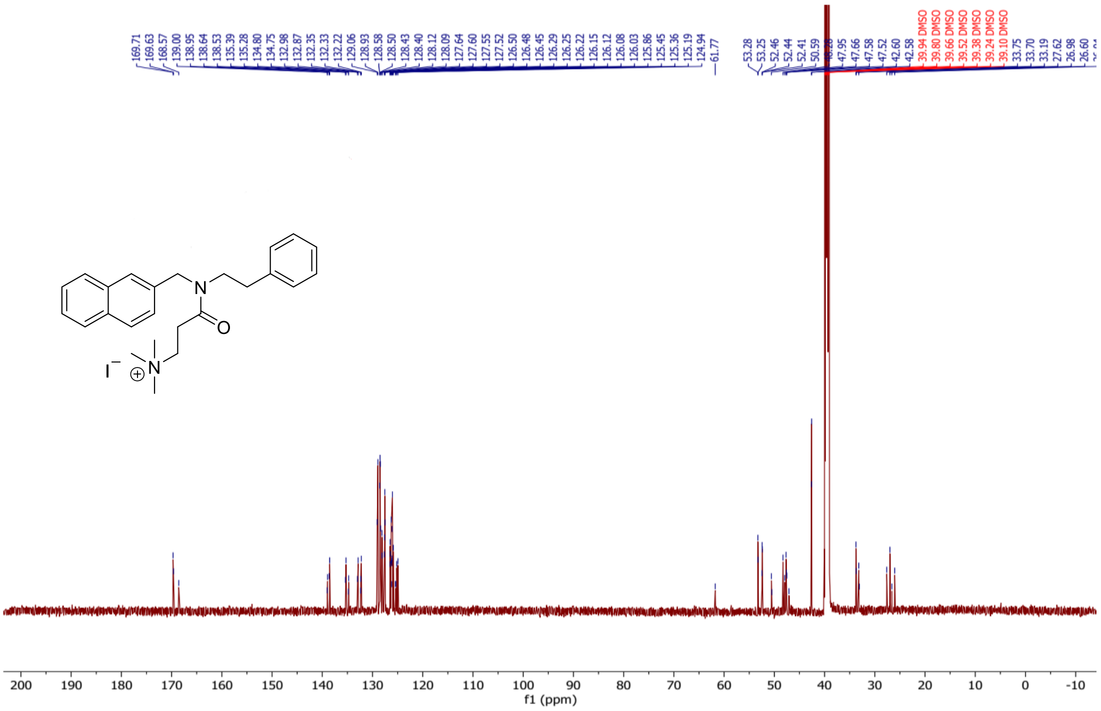

*171. Compound **16c**-^{13}CNMR-dept 135*

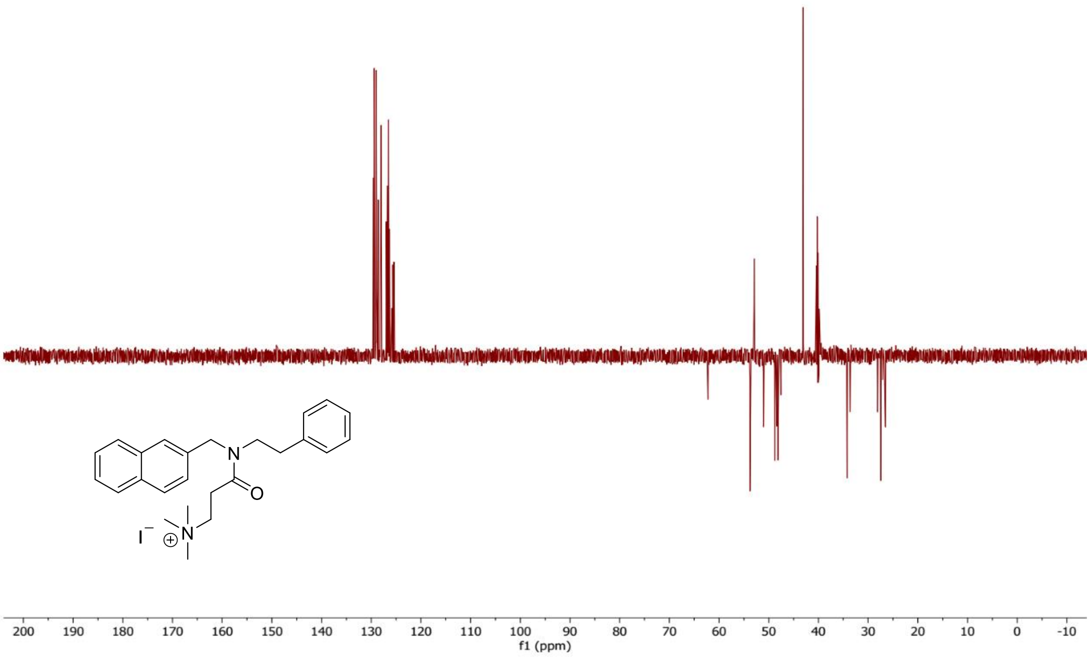

*172. Compound **16c** -HSQC*

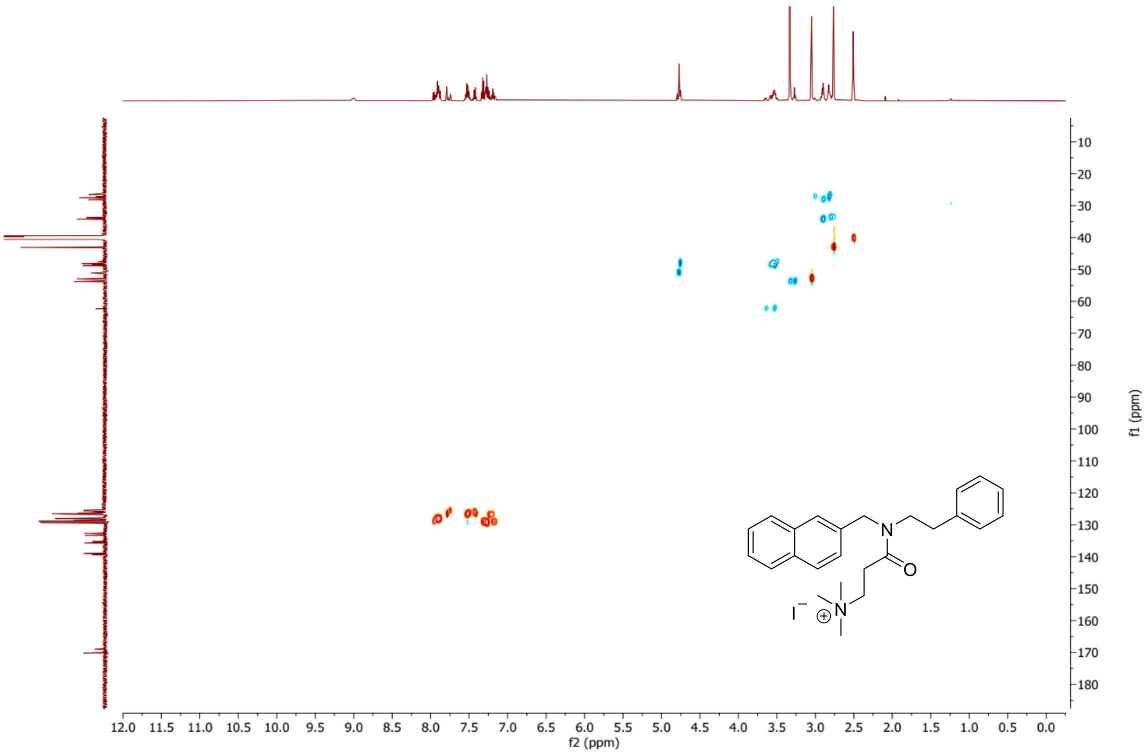

*173. Compound **16d** -^{1}HNMR*

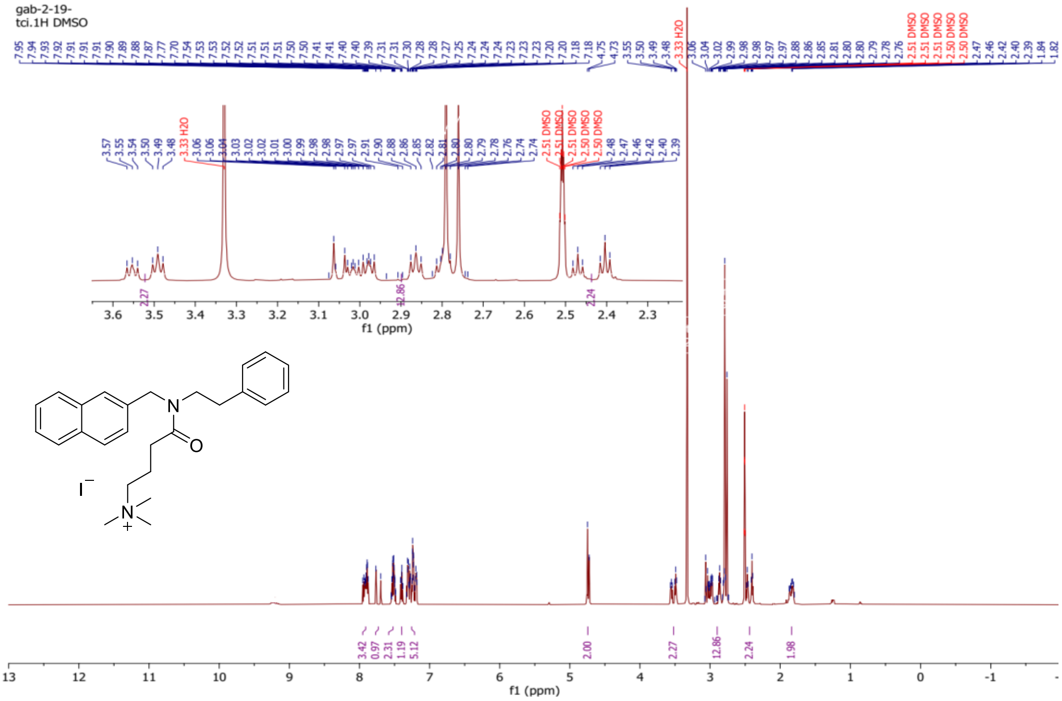

*174. Compound **16d** -^{13}CNMR*

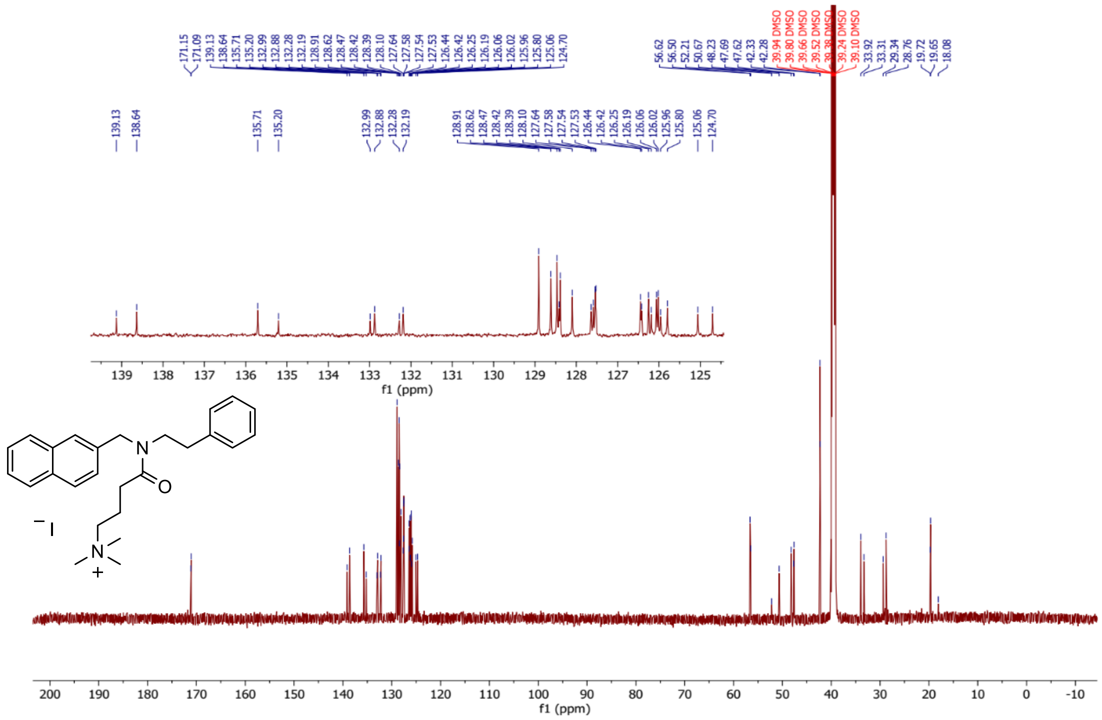

*175. Compound **16d** -^{13}CNMR-dept 135*

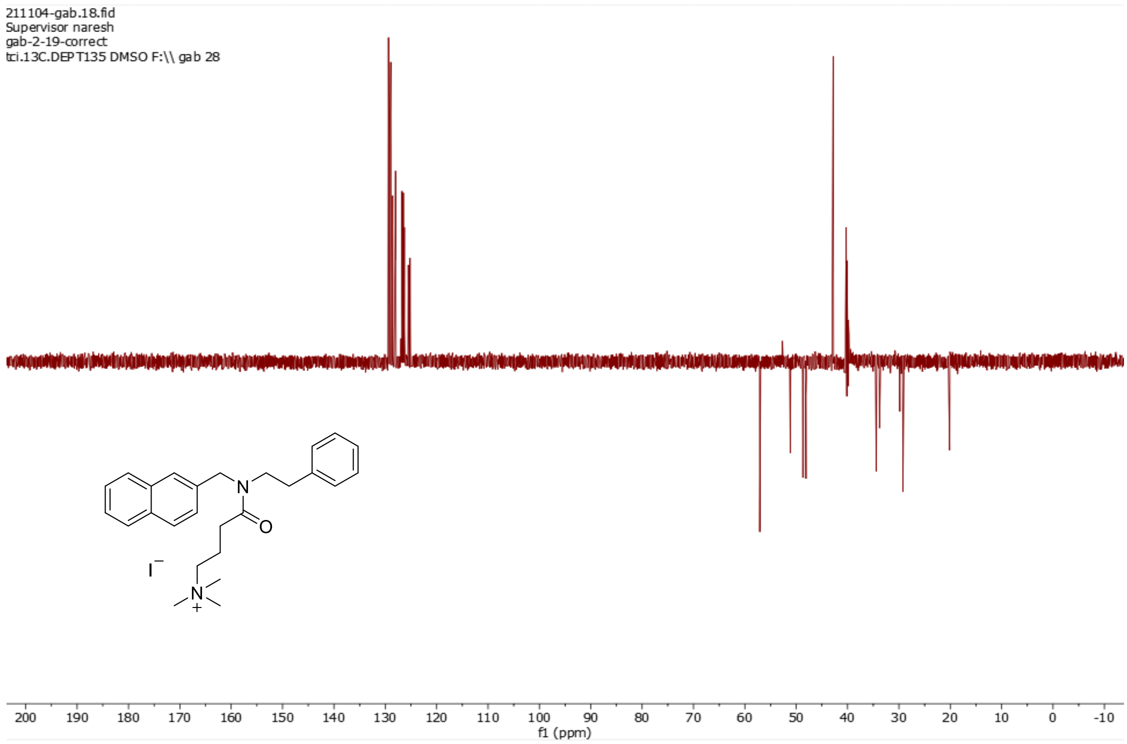

*176. Compound **16d** -HSQC*

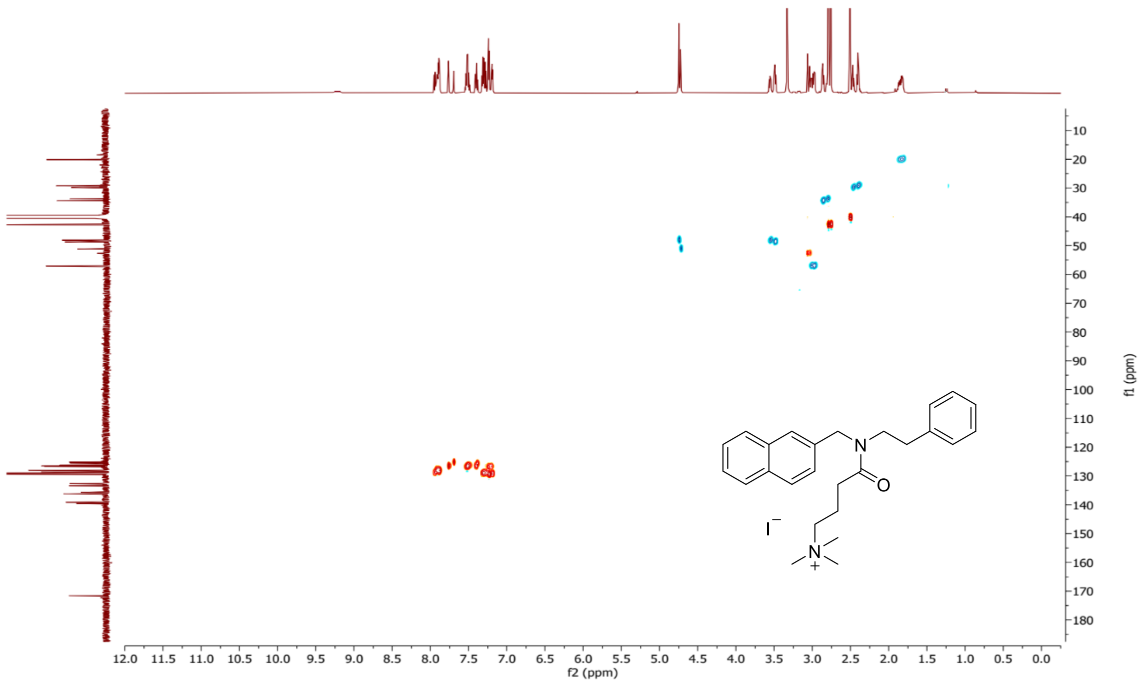

15- Expanded ^{1}HNMR spectra for some regions not clear in the full ^{1}HNMR spectra:

12c

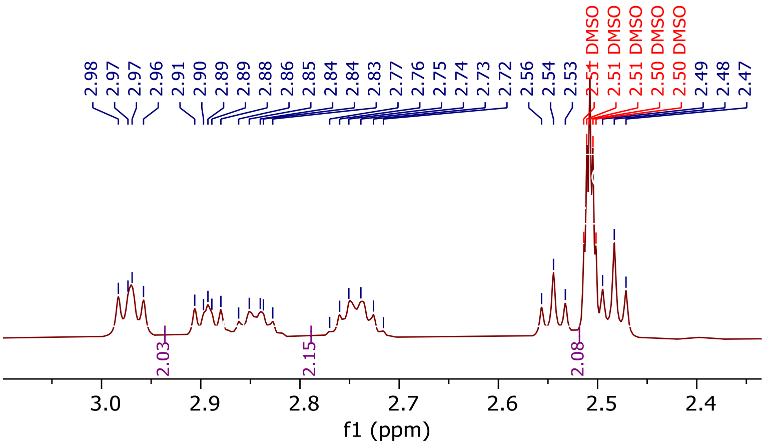

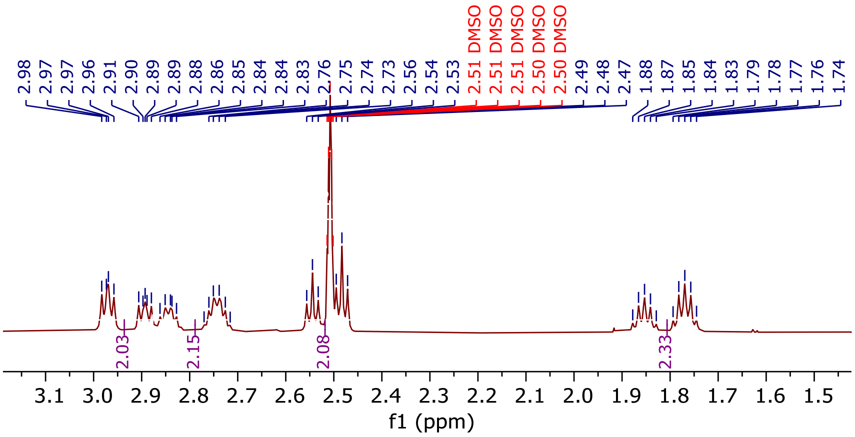

12f

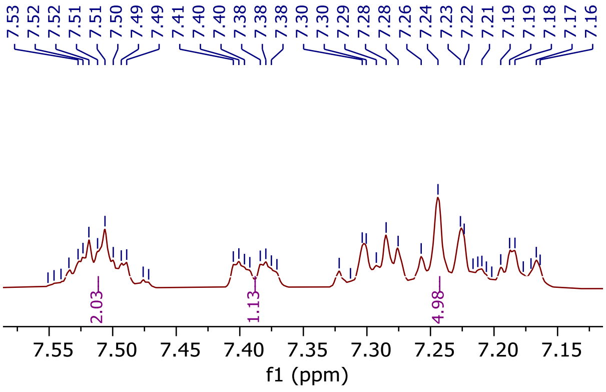

15 a

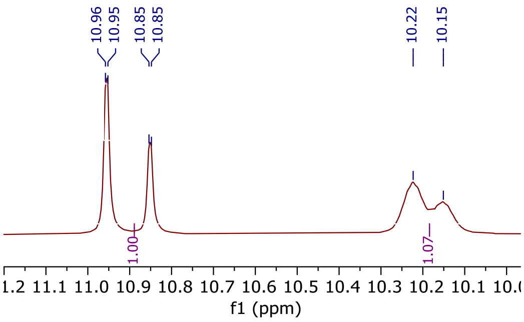
10.96
10.95
10.85
10.85
10.22
10.15
1.00
1.07
f1 (ppm)

15b

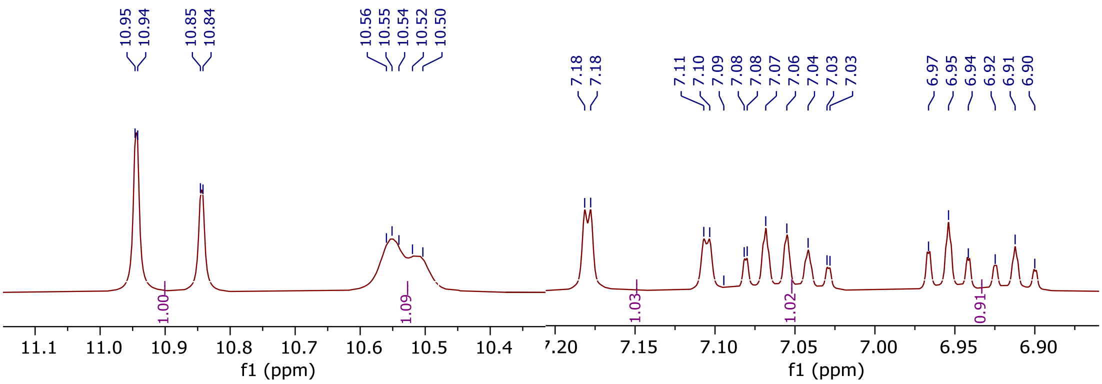
10.95
10.94
10.85
10.84
10.56
10.55
10.54
10.52
10.50
1.00
1.09
f1 (ppm)
7.18
7.18
7.11
7.10
7.09
7.08
7.08
7.07
7.06
7.04
7.03
7.03
6.97
6.95
6.94
6.92
6.91
6.90
1.03
1.02
0.91
f1 (ppm)

15c

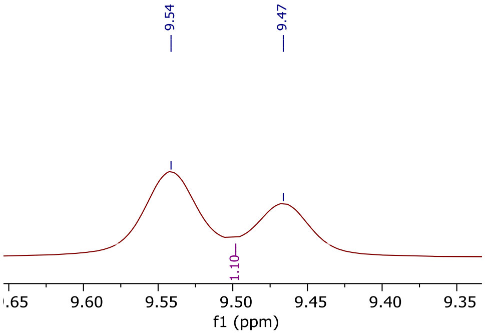
9.54
9.47
1.10
f1 (ppm)

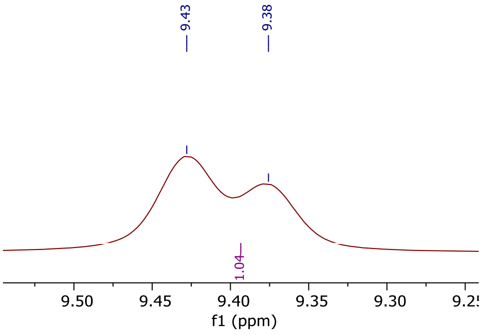
9.43
9.38
1.04
9.50 9.45 9.40 9.35 9.30
f1 (ppm)

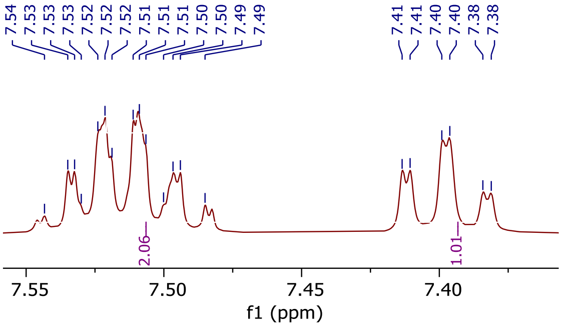
7.54 7.53 7.53 7.53 7.52 7.52 7.52 7.51 7.51 7.51 7.50 7.50 7.49 7.49
7.41 7.41 7.40 7.40 7.38 7.38
2.06
1.01
7.55 7.50 7.45 7.40
f1 (ppm)

16 a

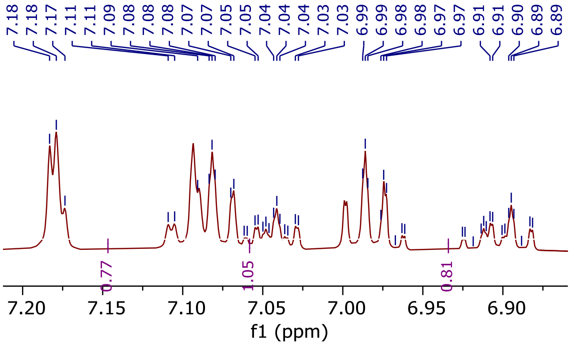
7.18 7.18 7.17 7.11 7.11 7.09 7.08 7.08 7.08 7.07 7.07 7.05 7.05 7.04 7.04 7.04 7.03 7.03 6.99 6.99 6.98 6.98 6.97 6.97 6.91 6.91 6.90 6.89 6.89
0.77
1.05
0.81
7.20 7.15 7.10 7.05 7.00 6.95 6.90
f1 (ppm)

16b

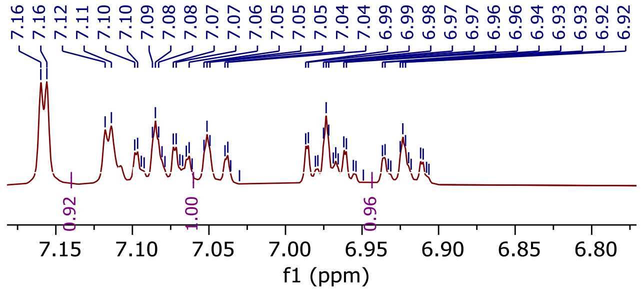

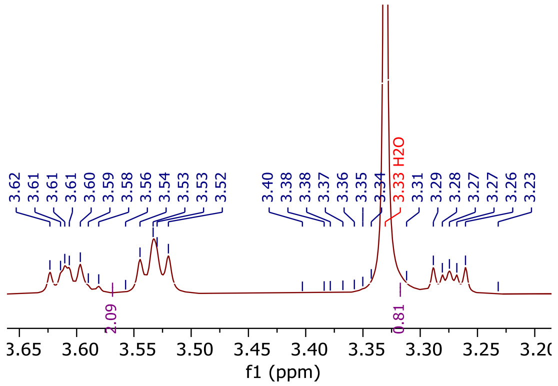

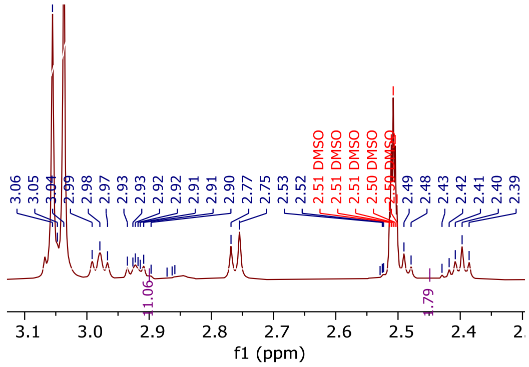

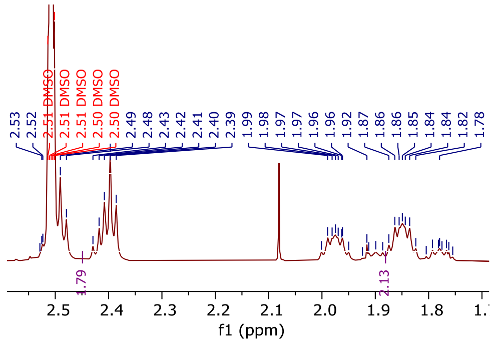

16d

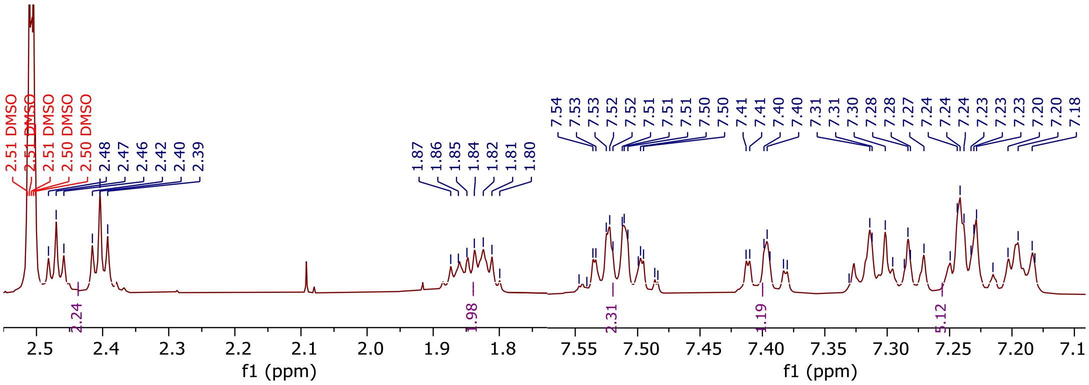
2.51 DMSO
2.51 DMSO
2.51 DMSO
2.50 DMSO
2.50 DMSO
2.48
2.47
2.46
2.42
2.40
2.39
1.87
1.86
1.85
1.84
1.82
1.81
1.80
7.54
7.53
7.53
7.52
7.52
7.51
7.51
7.51
7.50
7.50
7.41
7.41
7.40
7.40
7.31
7.31
7.30
7.28
7.28
7.27
7.24
7.24
7.24
7.23
7.23
7.23
7.20
7.20
7.18
2.24
1.98
2.31
1.19
5.12
f1 (ppm)
f1 (ppm)

18a

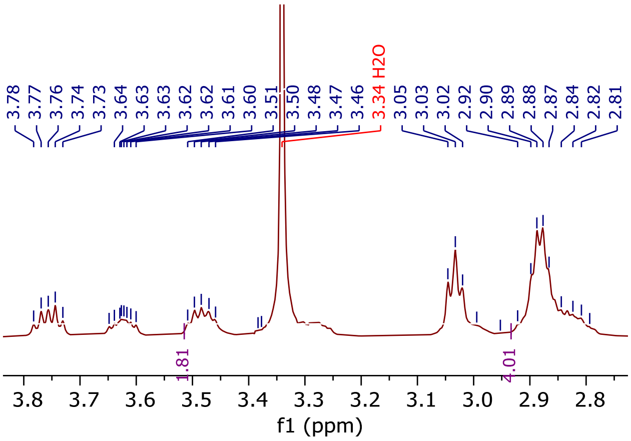
3.78
3.77
3.76
3.74
3.73
3.64
3.63
3.63
3.62
3.62
3.61
3.60
3.51
3.50
3.48
3.47
3.46
3.34 H2O
3.05
3.03
3.02
2.92
2.90
2.89
2.88
2.87
2.84
2.82
2.81
1.81
4.01
f1 (ppm)

18b

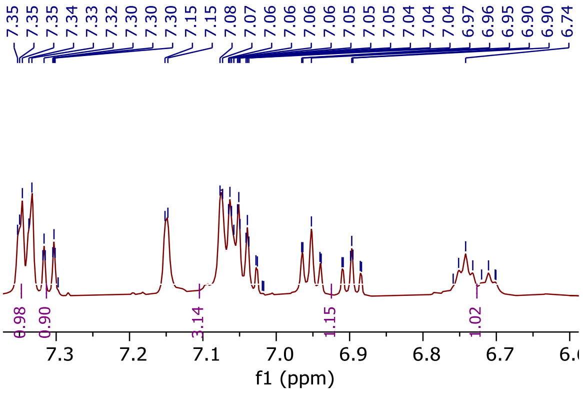
7.35
7.35
7.35
7.34
7.33
7.32
7.30
7.30
7.30
7.15
7.15
7.08
7.07
7.06
7.06
7.06
7.05
7.05
7.05
7.04
7.04
7.04
6.97
6.96
6.95
6.90
6.90
6.74
0.98
0.90
3.14
1.15
1.02
f1 (ppm)

19a

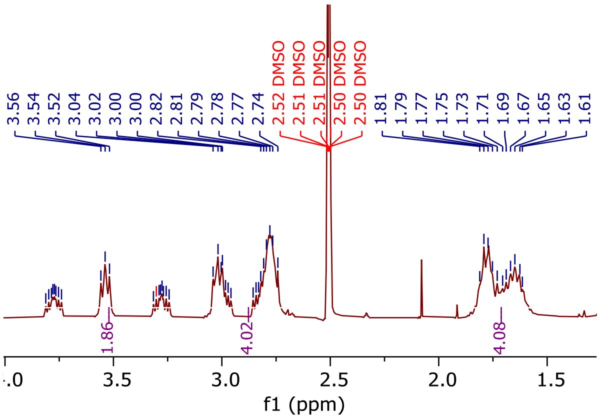

19b

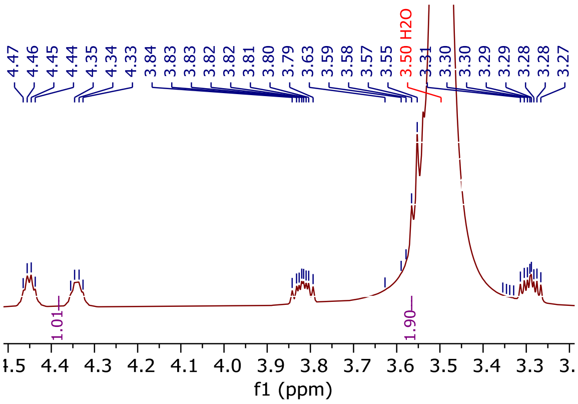

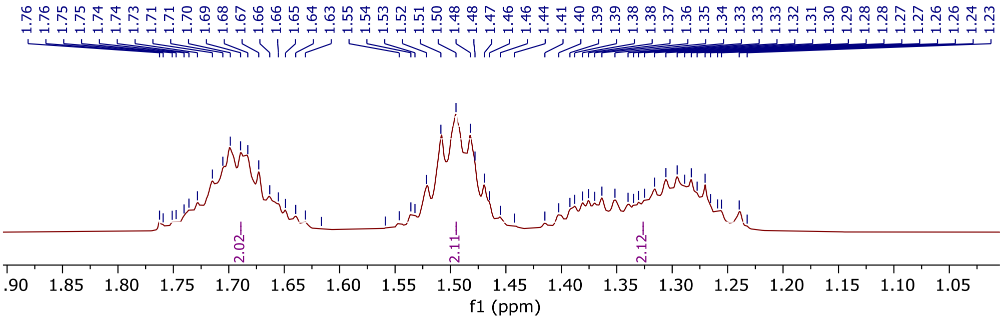

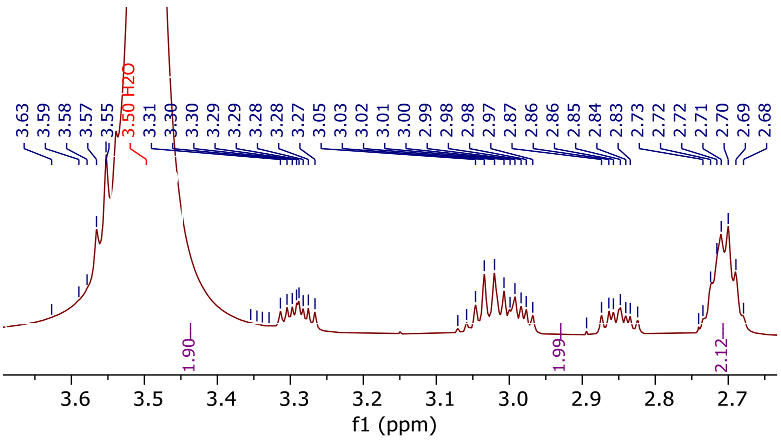

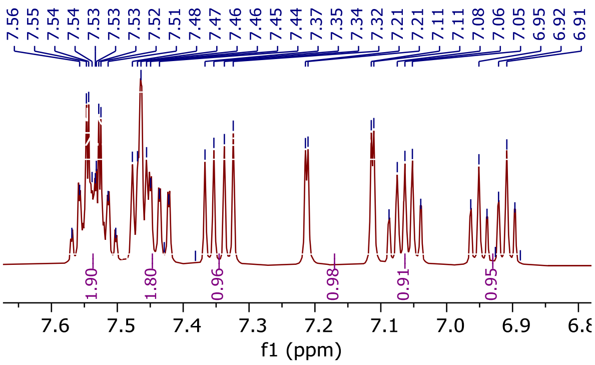

20a

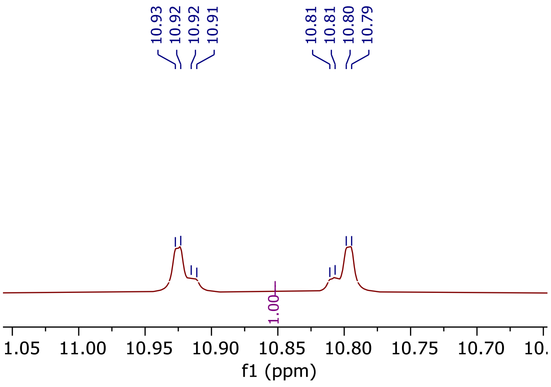

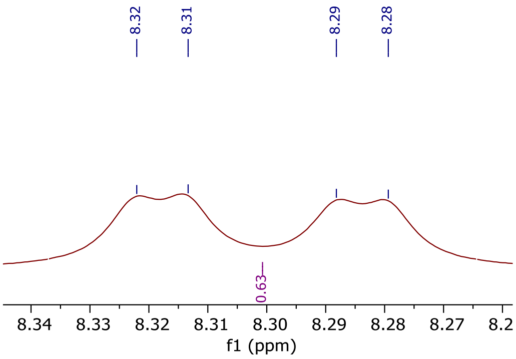

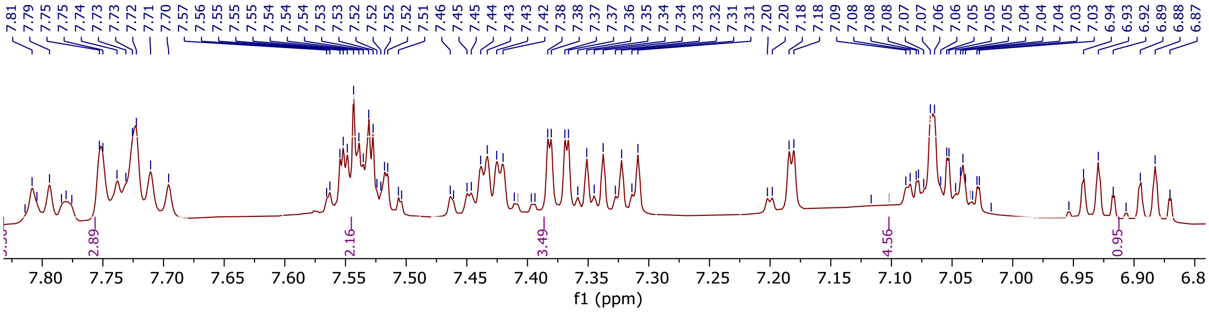

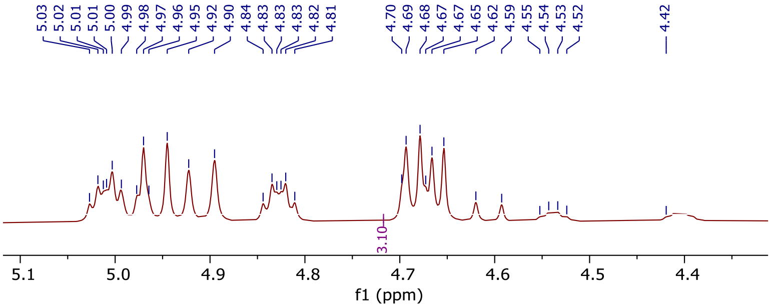

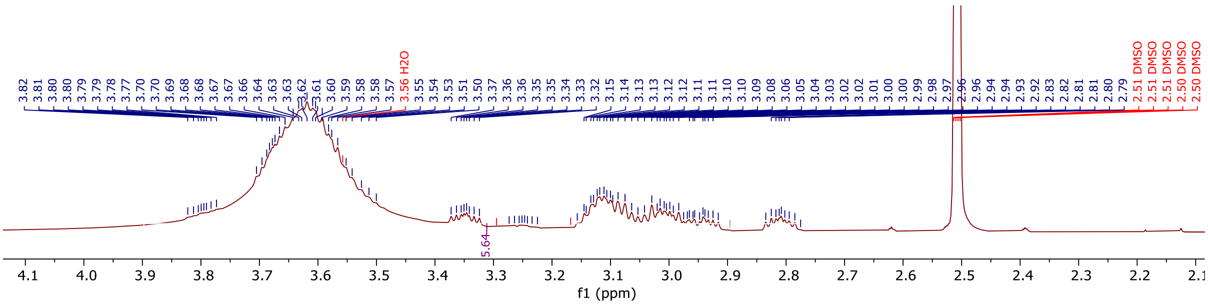

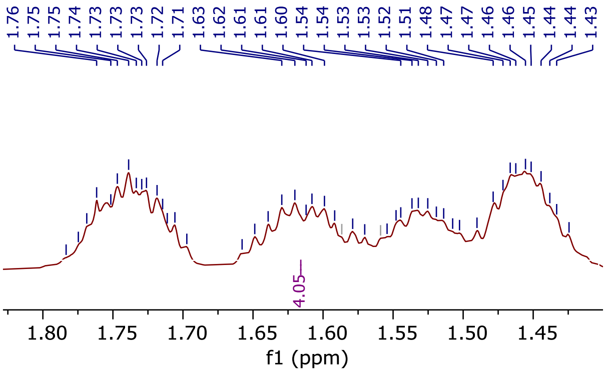

20b

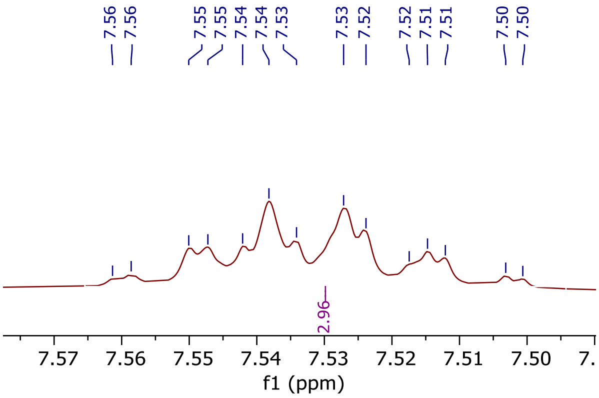
7.56
7.56
7.55
7.55
7.54
7.54
7.53
7.53
7.52
7.52
7.51
7.51
7.50
7.50
2.96
7.57 7.56 7.55 7.54 7.53 7.52 7.51 7.50 7.
f1 (ppm)

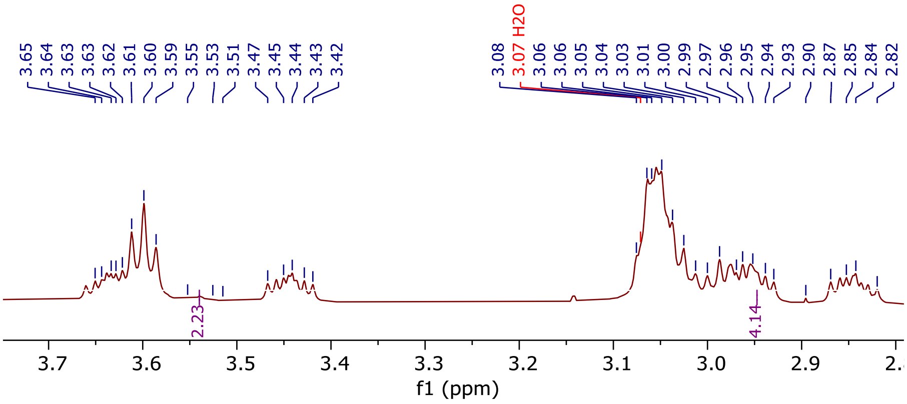
3.65
3.64
3.63
3.63
3.62
3.61
3.60
3.59
3.55
3.53
3.51
3.47
3.45
3.44
3.43
3.42
3.08
3.07 H2O
3.06
3.06
3.05
3.04
3.03
3.01
3.00
2.99
2.97
2.96
2.95
2.94
2.93
2.90
2.87
2.85
2.84
2.82
2.23
4.14
3.7 3.6 3.5 3.4 3.3 3.2 3.1 3.0 2.9 2.
f1 (ppm)

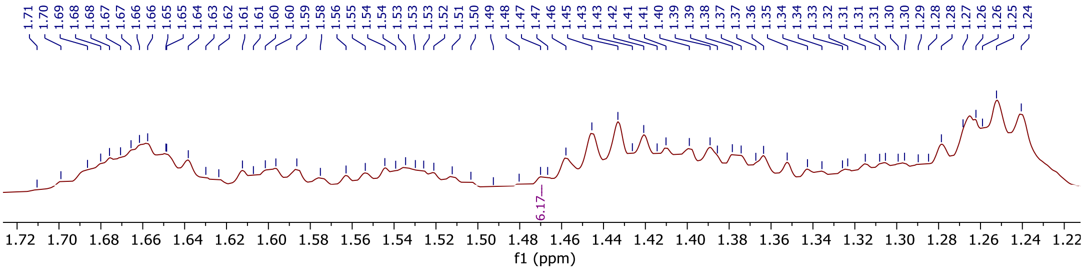
6.17
1.72 1.70 1.68 1.66 1.64 1.62 1.60 1.58 1.56 1.54 1.52 1.50 1.48 1.46 1.44 1.42 1.40 1.38 1.36 1.34 1.32 1.30 1.28 1.26 1.24 1.22
f1 (ppm)

21

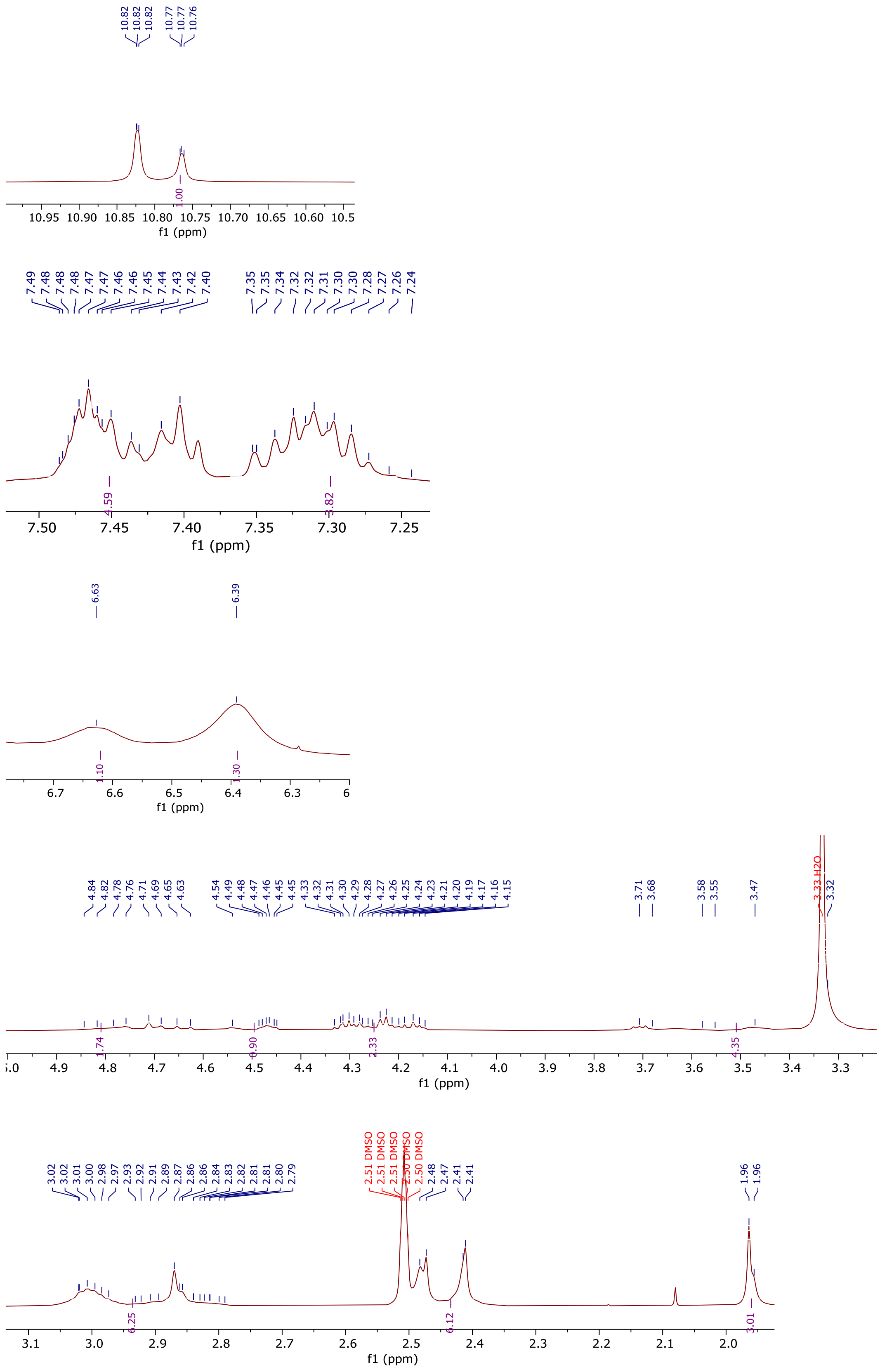

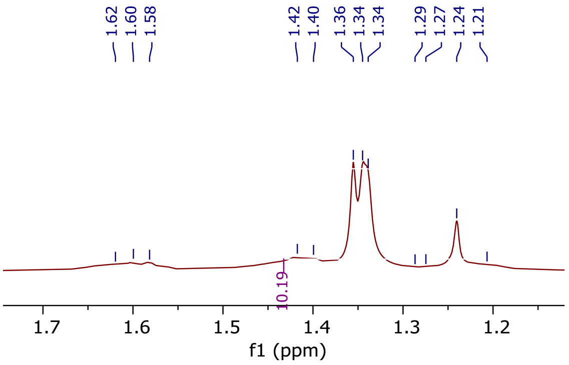
1.62
1.60
1.58
1.42
1.40
1.36
1.34
1.34
1.29
1.27
1.24
1.21
10.19
f1 (ppm)

22

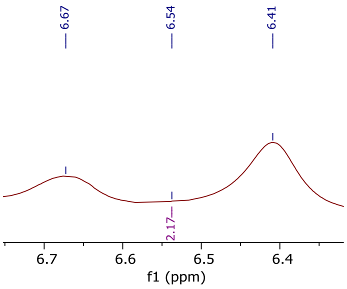
6.67
6.54
6.41
2.17
f1 (ppm)

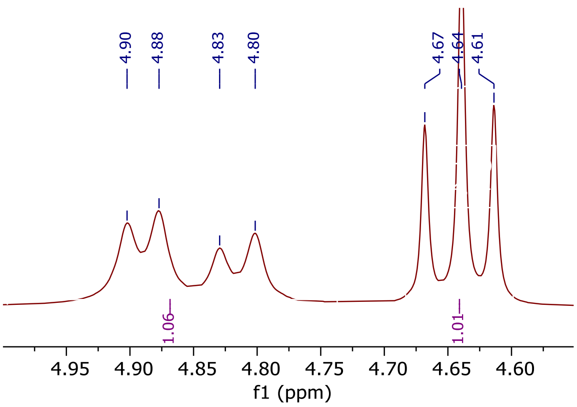
4.90
4.88
4.83
4.80
4.67
4.64
4.61
1.06
1.01
f1 (ppm)

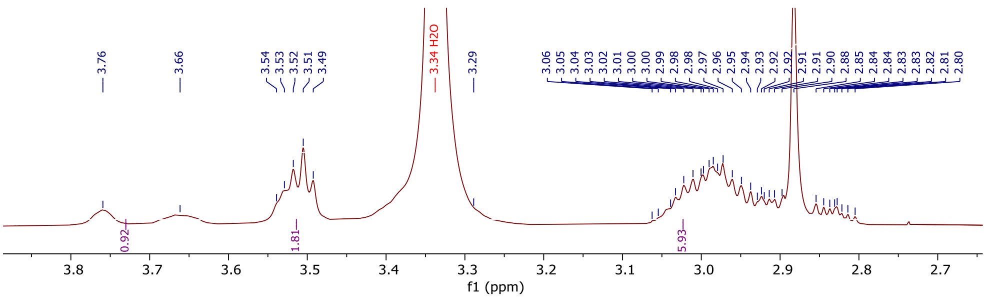
3.76
3.66
3.54
3.53
3.52
3.51
3.49
3.34 H2O
3.29
0.92
1.81
5.93
f1 (ppm)

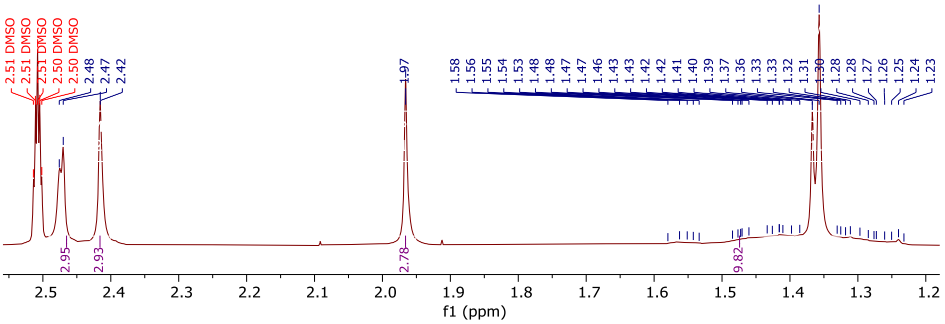

23

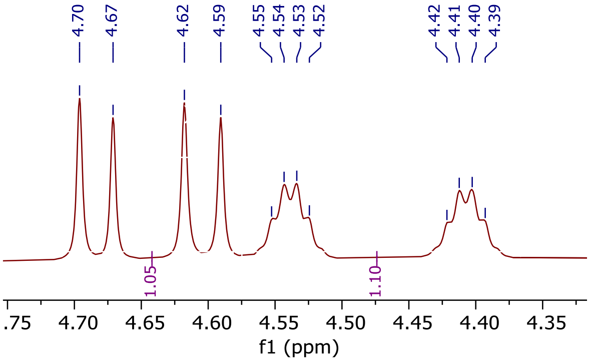

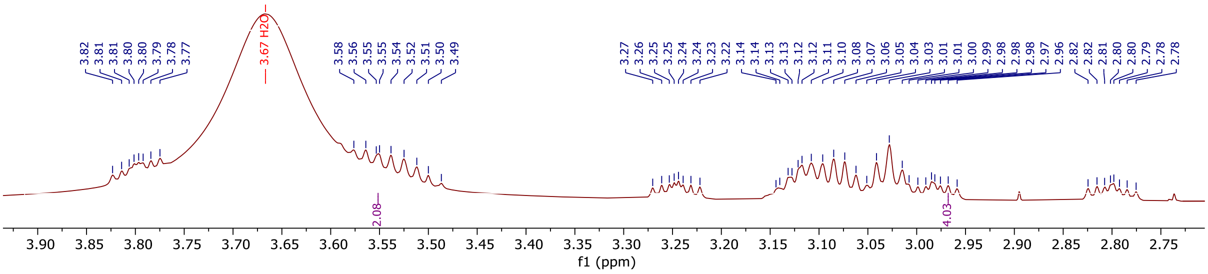

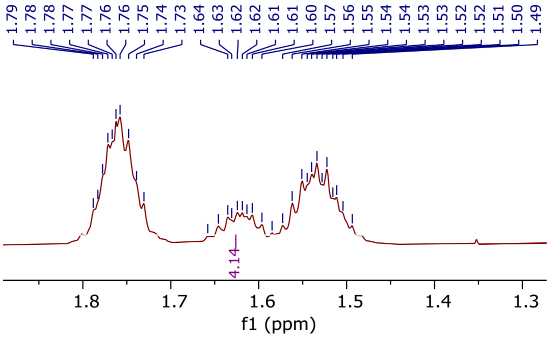

The Supplementary Data for

Short tryptamine based peptoids as potential therapeutics for microbial keratitis: Structure-function correlation studies

Ghayah Bahatheg [1,3], Rajesh Kuppusamy [1,2,*], Muhammad Yasir [2], David StC Black [1], Mark Willcox [2] and Naresh Kumar [1,*]

[1] School of Chemistry, The University of New South Wales, UNSW Sydney, NSW 2052, Australia; g.bahatheg@unsw.edu.au (G.B); d.black@unsw.edu.au (D.S.B)

[2] School of Optometry and Vision Science, The University of New South Wales, UNSW Sydney, NSW 2052, Australia; m.yasir@unsw.edu.au (M.Y); m.willcox@unsw.edu.au (M.W).

[3] Department of Chemistry, Faculty of Science, University of Jeddah, Jeddah, 21589, Saudi Arabia.

* Correspondence: n.kumar@unsw.edu.au (N.K); r.kuppusamy@unsw.edu.au (R.K)

contents

HRMS of 2-(*1H*-indol-3-yl)-*N*-(naphthalen-2-ylmethyl) ethan-1-amine (8) S114

HRMS of *N*-(naphthalen-2-ylmethyl)-2-phenylethan-1-amine (9) S115

HRMS of Tert-butyl(2-((2-(*1H*-indol-3-yl)ethyl)(naphthalen-2-ylmethyl)amino)-2-oxoethyl)carbamate (11a) ... S116

HRMS of Tert-butyl(3-((2-(*1H*-indol-3-yl) ethyl) (naphthalen-2-ylmethyl) amino)-3 oxopropyl) carbamate (11b) S117

HRMS of Tert-butyl(4-((2-(*1H*-indol-3-yl) ethyl) (naphthalen-2-ylmethyl) amino)-4-oxobutyl) carbamate (11c)S118

HRMS of Tert-butyl (2-((naphthalen-2-ylmethyl) (phenethyl)amino)-2-oxoethyl) carbamate (11d)....... S119

HRMS of Tert-butyl (3-((naphthalen-2-ylmethyl) (phenethyl)amino)-3-oxopropyl) carbamate (11e). S120

HRMS of Tert-butyl (4-((naphthalen-2-ylmethyl) (phenethyl)amino)-4-oxobutyl) carbamate (11f) S121

HRMS of N-(2-(*1H*-indol-3-yl) ethyl)-2-amino-*N*-(naphthalen-2-ylmethyl) acetamide (TFA salt) (12a)....... S122

HRMS of *N*-(2-(*1H*-indol-3-yl) ethyl)-3-amino-*N*-(naphthalen-2-ylmethyl) propenamide (TFA salt) (12b) S123

HRMS of *N*-(2-(1H-indol-3-yl) ethyl)-4-amino-*N*-(naphthalen-2-ylmethyl) butanamide (TFA salt) (12c) S123

HRMS of 2-Amino-*N*-(naphthalen-2-ylmethyl)-*N*-phenethylacetamide (TFA salt) (12d)....... S125

HRMS of 3-Amino-*N*-(naphthalen-2-ylmethyl)-*N*-phenethylpropanamide (TFA salt) (12e)....... S125

HRMS of 4-Amino-*N*-(naphthalen-2-ylmethyl)-*N*-phenethyl- butanamide (TFA salt) (12f) S126

HRMS of N-(2-(1H-indol-3-yl) ethyl)-2-guanidino-*N*-(naphthalen-2-ylmethyl) acetamide....... 128

(TFAsalt)(13a)....... S128

HRMS of N-(2-(*1H*-indol-3-yl) ethyl)-3-guanidino-*N*-(naphthalen-2-ylmethyl) propenamide (TFA salt) (13b).. S128

HRMS of *N*-(2-(*1H*-indol-3-yl) ethyl)-4-guanidino-N-(naphthalen-2-ylmethyl) butanamide (TFA salt) (13c) S129

HRMS of 2-Guanidino-*N*-(naphthalen-2-ylmethyl)-*N*-phenethyl acetamide (TFA salt) (13d) S131

HRMS of 3-Guanidino-*N*-(naphthalen-2-ylmethyl)-*N*-phenethyl- propanamide (TFA salt) (13e) S132

HRMS of 4-Guanidino-*N*-(naphthalen-2-ylmethyl)-*N*-phenethyl- butanamide (TFA salt) (13f) S133

HRMS of *N*-(2-(*1H*-indol-3-yl) ethyl)-3-(dimethylamino)-*N*-(naphthalen-2-ylmethyl) propenamide (14a).........S134
HRMS of *N*-(2-(*1H*-indol-3-yl) ethyl)-4-(dimethylamino)-*N*-(naphthalen-2-ylmethyl) butanamide (14b)...........S135
HRMS of 4-(Dimethylamino)-*N*-(naphthalen-2-ylmethyl)-*N*-phenethyl-butanamide (14c) S136
HRMS of 3-(Dimethylamino)-*N*-(naphthalen-2-ylmethyl)-*N*-phenethyl-propanamide (14d) S137
HRMS of *N*-(2-(*1H*-indol-3-yl) ethyl)-3-(dimethylamino)-*N*-(naphthalen-2-ylmethyl) propenamide (15a).........S138
HRMS of *N*-(2-(*1H*-indol-3-yl) ethyl)-4-(dimethylamino)-*N*-(naphthalen-2-ylmethyl) butanamide (15b)...........S139
HRMS of 3-(Dimethylamino)-*N*-(naphthalen-2-ylmethyl)-*N*-phenethyl-propanamide (15c)..............................S140
HRMS of 4-(Dimethylamino)-*N*-(naphthalen-2-ylmethyl)-*N*-phenethyl-butanamide (15d)................................S141
HRMS of 3-((2-(*1H*-indol-3-yl) ethyl) (naphthalen-2-ylmethyl) amino)-*N,N,N*-trimethyl-3-oxopropan-1-aminium (16a) S142
HRMS of 4-((2-(*1H*-indol-3-yl) ethyl) (naphthalen-2-ylmethyl) amino)-*N,N,N*-trimethyl-4-oxobutan-1-aminium (16b) S143
HRMS of *N,N,N*-Trimethyl-3-((naphthalen-2-ylmethyl)(phenethyl) amino)-3-oxopropan-1-aminium (16c)......S144
HRMS of *N,N,N*-Trimethyl-4-((naphthalen-2-ylmethyl)(phenethyl) amino)-4-oxobutan-1-aminium (16d)........S145
HRMS of Di-tert-butyl(5-((2-(*1H*-indol-3-yl) ethyl) (naphthalen-2-ylmethyl) amino)-5-oxopentane-1,4-diyl) (S)-dicarbamate (18a)....................S146
HRMS of Di-tert-butyl(6-((2-(*1H*-indol-3-yl) ethyl) (naphthalen-2-ylmethyl) amino)-6-oxohexane-1,5-diyl) (S)-dicarbamate (18b)....................S147
HRMS of (S)-*N*-(2-(*1H*-indol-3-yl) ethyl)-2,5-diamino-*N*-(naphthalen-2-ylmethyl) pentanamide (TFA salt) (19a) S148
HRMS of (S)-*N*-(2-(*1H*-indol-3-yl) ethyl)-2,6-diamino-*N*-(naphthalen-2-ylmethyl) hexanamide (TFA salt) (19b) S149
HRMS of *N*-(2-(*1H*-indol-3-yl) ethyl)-2,5-diguanidino-*N*-(naphthalen-2-ylmethyl) pentanamide (TFA salt) (20a) S150
HRMS of (S)-*N*-(2-(*1H*-indol-3-yl) ethyl)-2,6-diguanidino-*N*-(naphthalen-2-ylmethyl) hexanamide (TFA salt) (20b) S151
HRMS of Tert-butyl(S)-(1-((2-(*1H*-indol-3-yl) ethyl) (naphthalen-2-ylmethyl) amino)-1-oxo-5-(3-((2,2,4,6,7-pentamethyl-2,3-dihydrobenzofuran-5-yl) sulfonyl)guanidino) pentan-2-yl) carbamate (21) S152
HRMS of (S)-*N*-(2-(*1H*-indol-3-yl) ethyl)-2-amino-*N*-(naphthalen-2-ylmethyl)-5-(3-((2,2,4,6,7-pentamethyl-2,3-dihydrobenzofuran-5-yl) sulfonyl) guanidino)pentanamide (22) S153
HRMS of (S)-*N*-(2-(*1H*-indol-3-yl) ethyl)-2-amino-5-guanidino-N-(naphthalen-2-ylmethyl) pentanamide (TFA salt) (23) S154
HRMS of *N*-benzyl-2-(*1H*-indol-3-yl) ethan-1-amine (24)....................S155
HRMS of Tert-butyl(2-((2-(*1H*-indol-3-yl)ethyl)(benzyl)amino)-2-oxoethyl)carbamate (25) S156
HRMS of *N*-(2-(*1H*-indol-3-yl) ethyl)-2-amino-*N*-benzylacetamide (26) (TFA salt) S157

HRMS of 2-(1H-indol-3-yl)-N-(naphthalen-2-ylmethyl) ethan-1-amine (8)

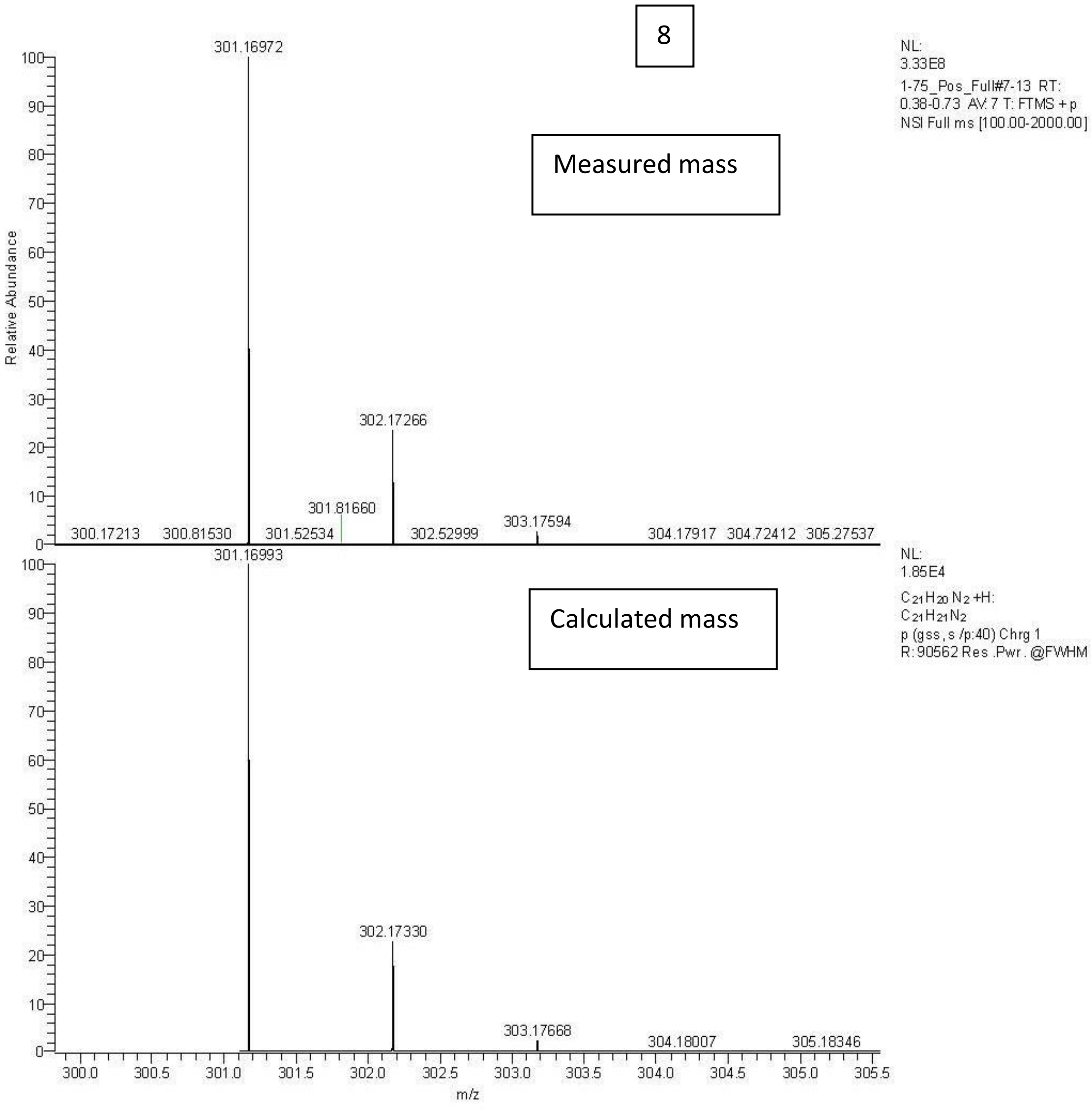

HRMS of N-(naphthalen-2-ylmethyl)-2-phenylethan-1-amine (9)

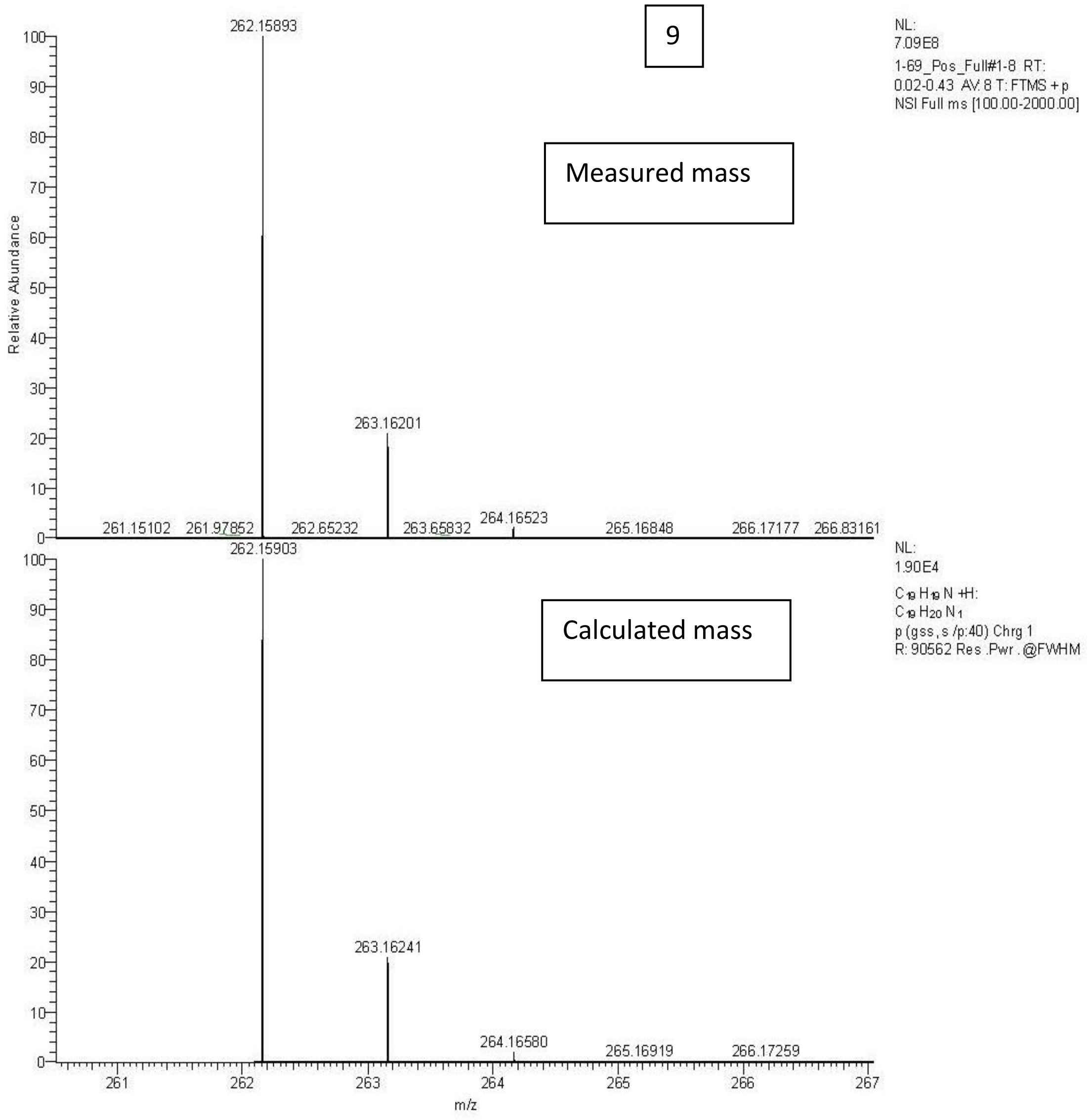

HRMS of Tert-butyl(2-((2-(1H-indol-3-yl)ethyl)(naphthalen-2-ylmethyl)amino)-2-oxoethyl)carbamate (11a)

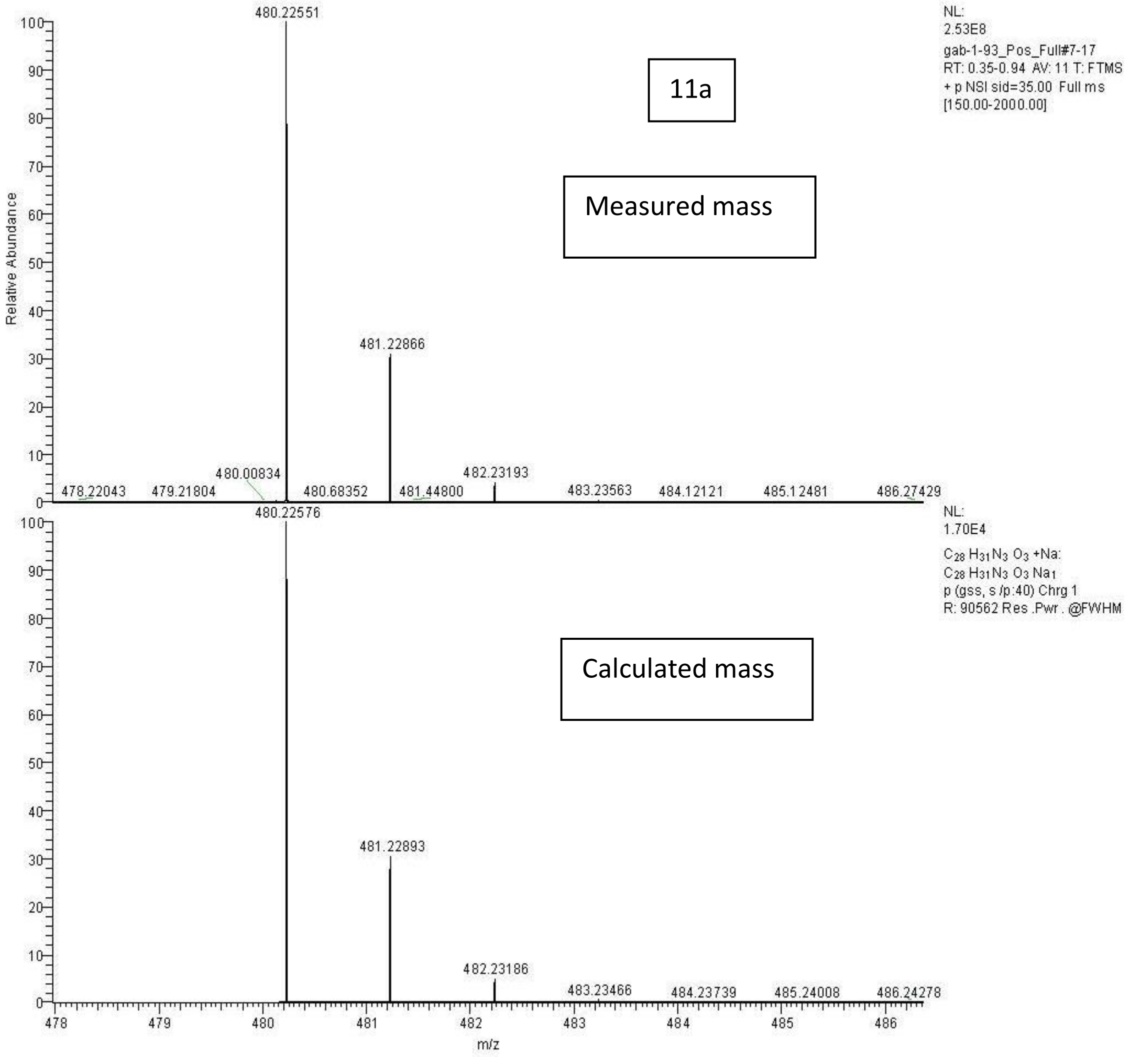

HRMS of Tert-butyl(3-((2-(1H-indol-3-yl) ethyl) (naphthalen-2-ylmethyl) amino)-3 oxopropyl) carbamate (11b)

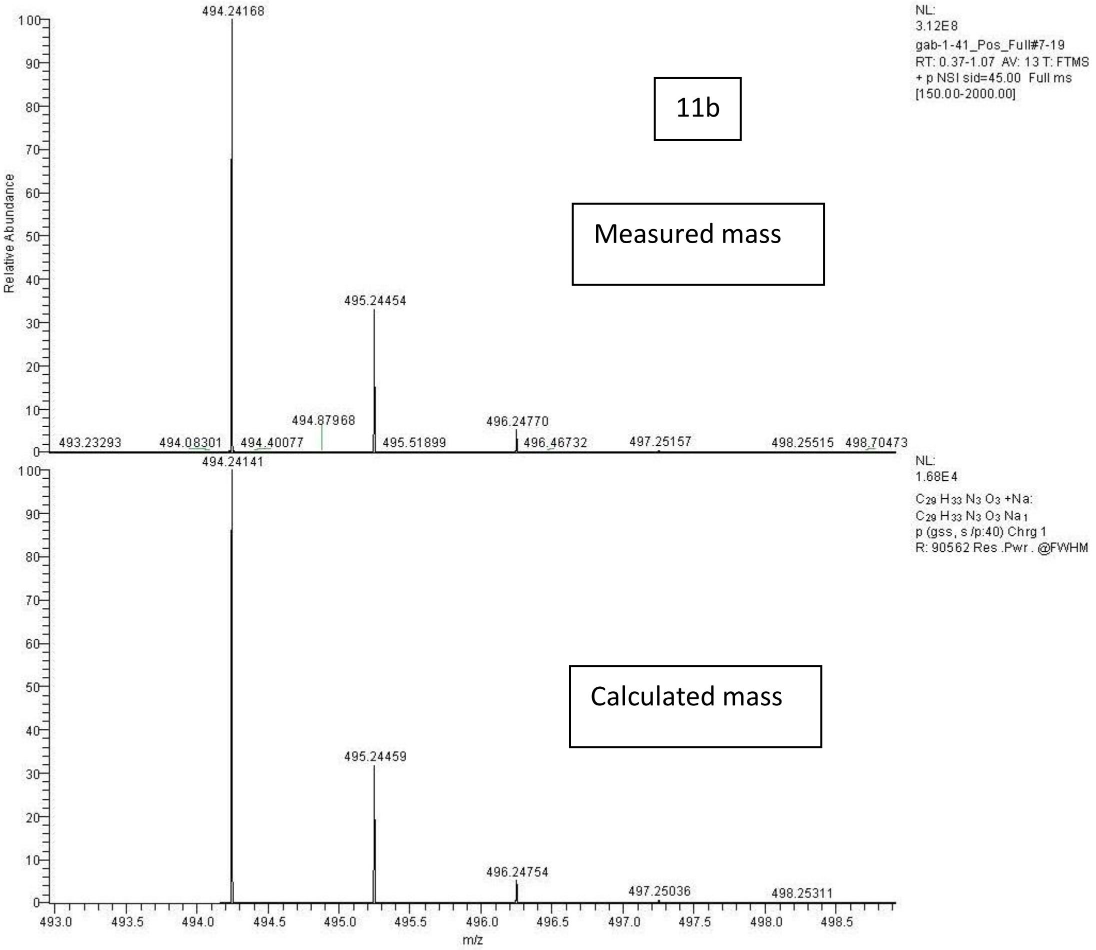

HRMS of Tert-butyl(4-((2-(1H-indol-3-yl) ethyl) (naphthalen-2-ylmethyl) amino)-4-oxobutyl) carbamate (11c)

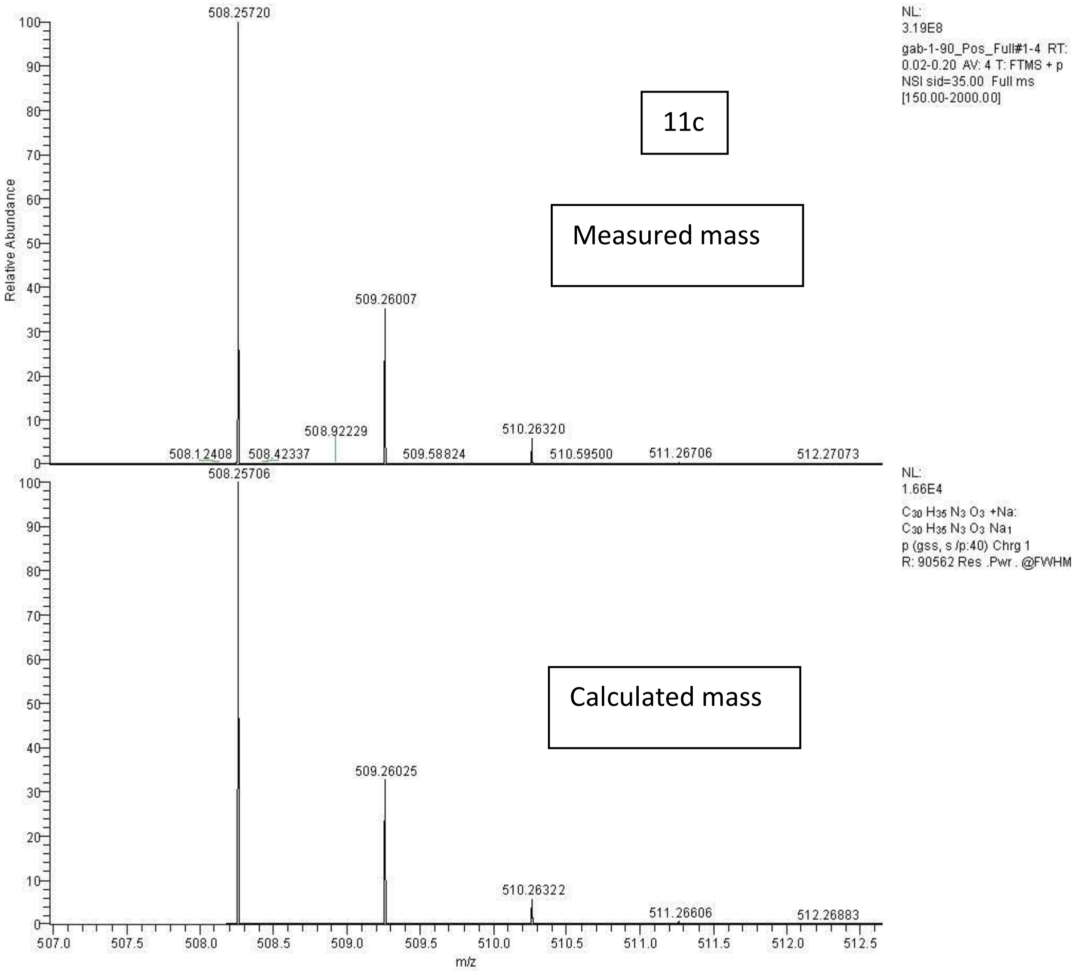

HRMS of Tert-butyl (2-((naphthalen-2-ylmethyl) (phenethyl)amino)-2-oxoethyl) carbamate (11d)

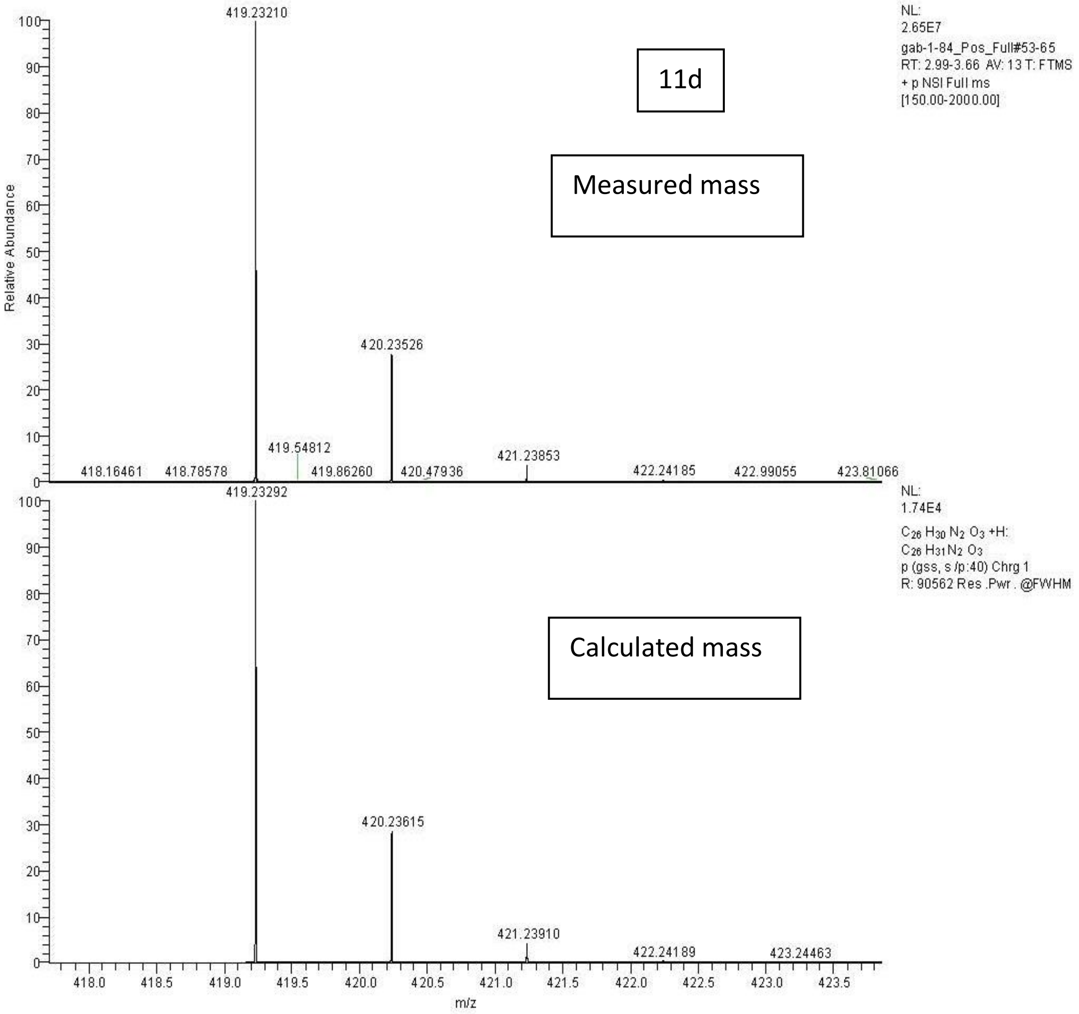

HRMS of Tert-butyl (3-((naphthalen-2-ylmethyl) (phenethyl)amino)-3-oxopropyl) carbamate (11e)

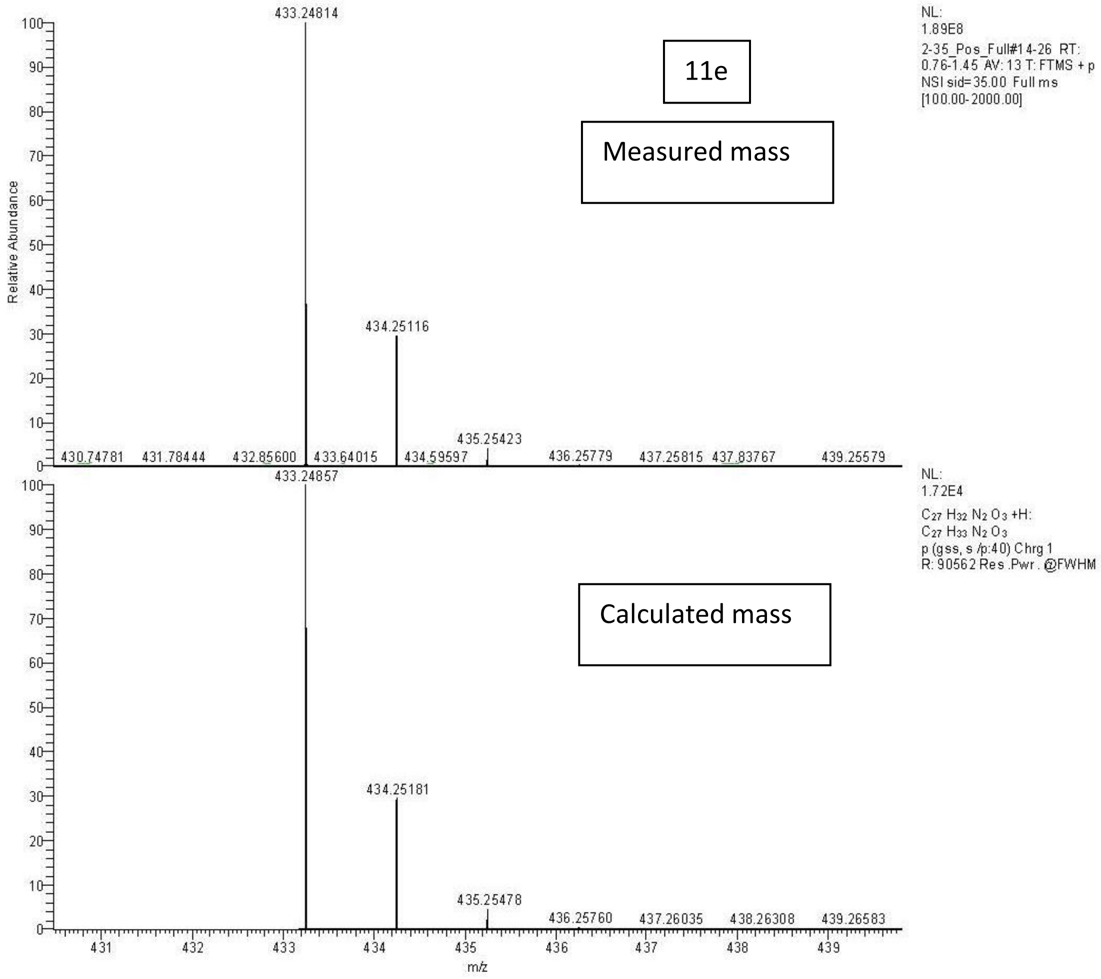

HRMS of Tert-butyl (4-((naphthalen-2-ylmethyl) (phenethyl)amino)-4-oxobutyl) carbamate (11f)

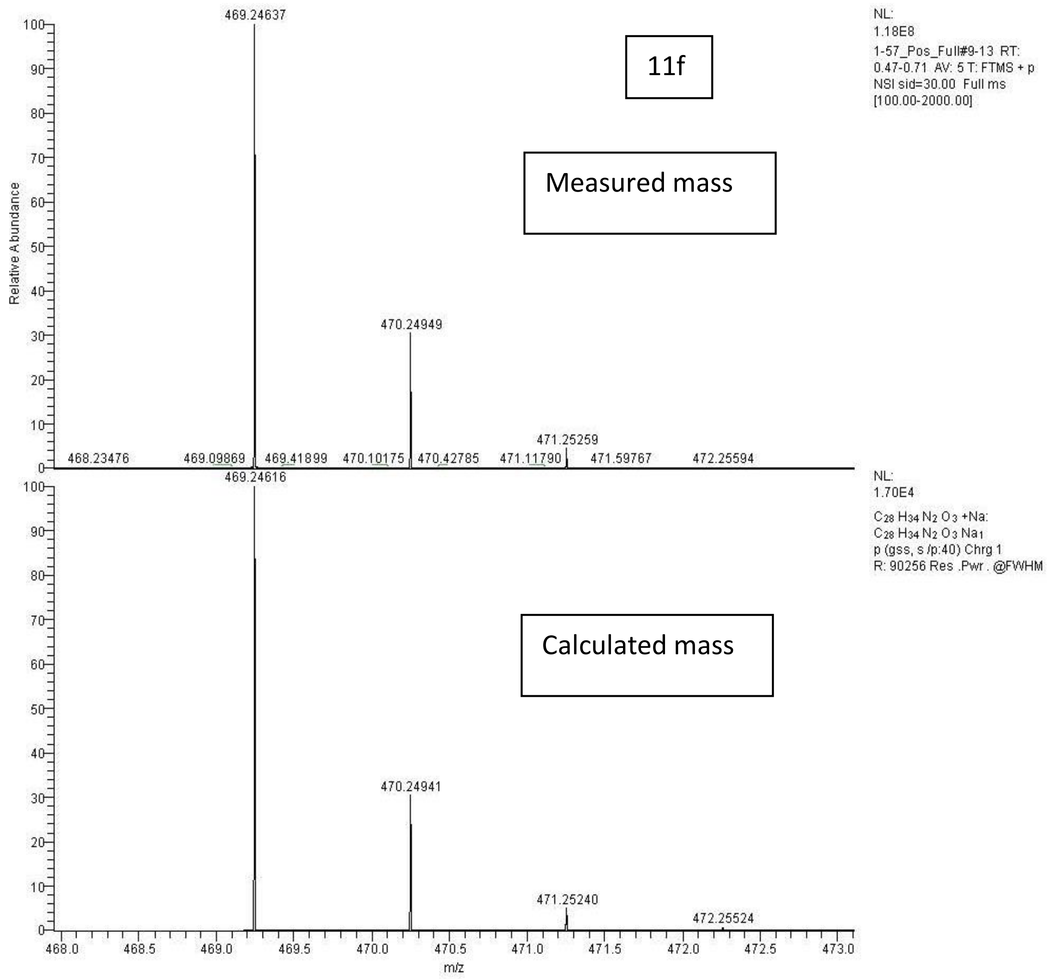

HRMS of N-(2-(1H-indol-3-yl) ethyl)-2-amino-N-(naphthalen-2-ylmethyl) acetamide (TFA salt) (12a)

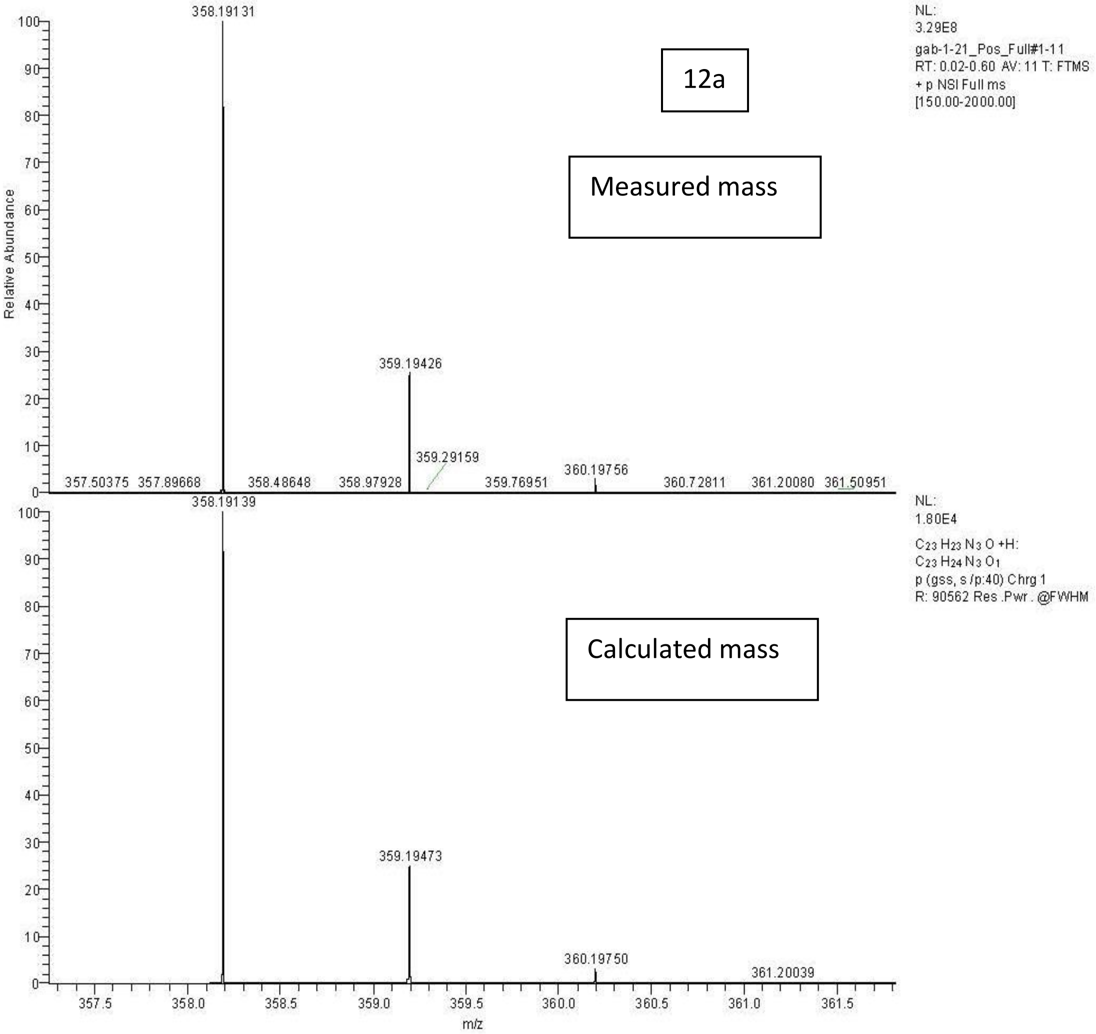

HRMS of N-(2-(1H-indol-3-yl) ethyl)-3-amino-N-(naphthalen-2-ylmethyl) propenamide (TFA salt) (12b)

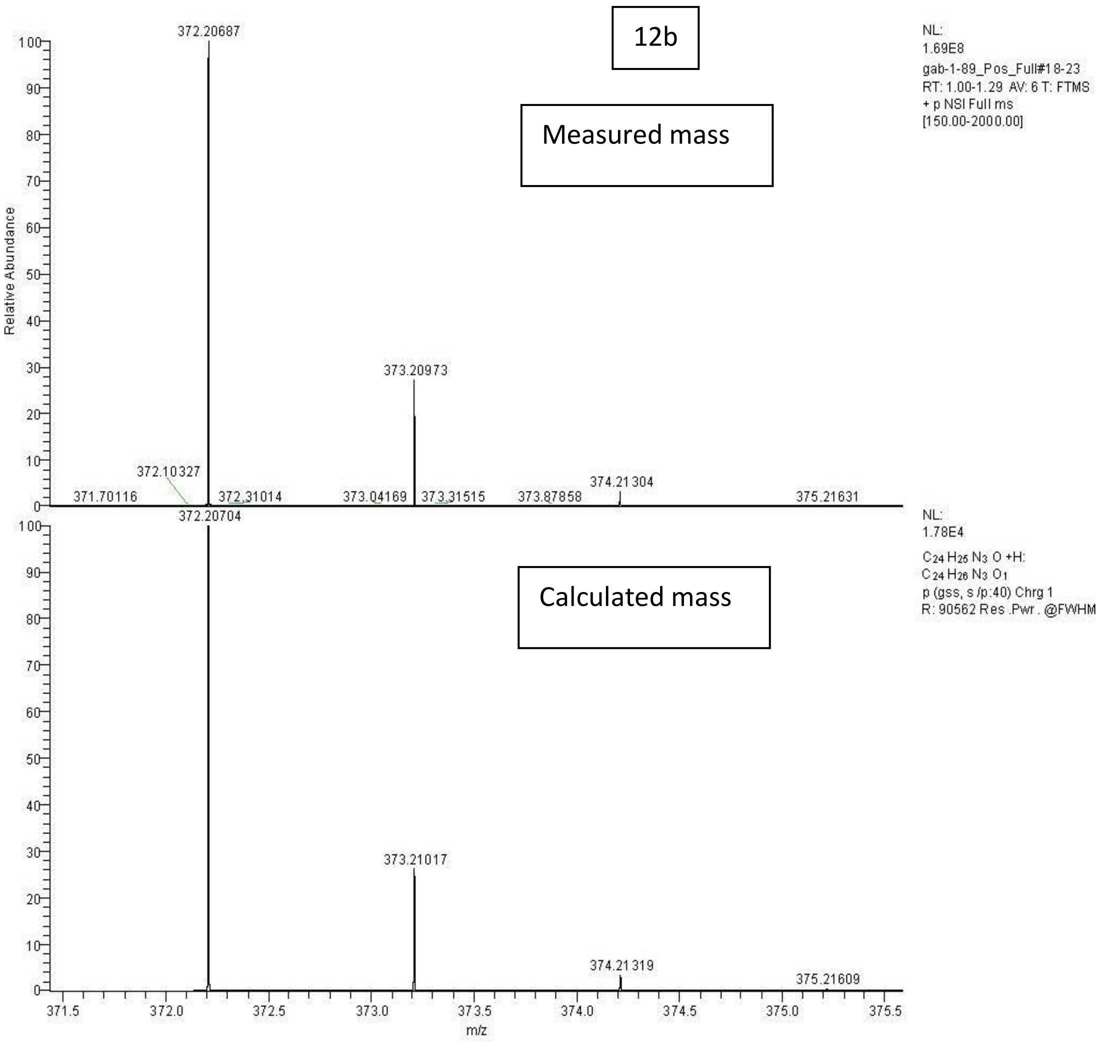

HRMS of N-(2-(1H-indol-3-yl) ethyl)-4-amino-N-(naphthalen-2-ylmethyl) butanamide (TFA salt) (12c)

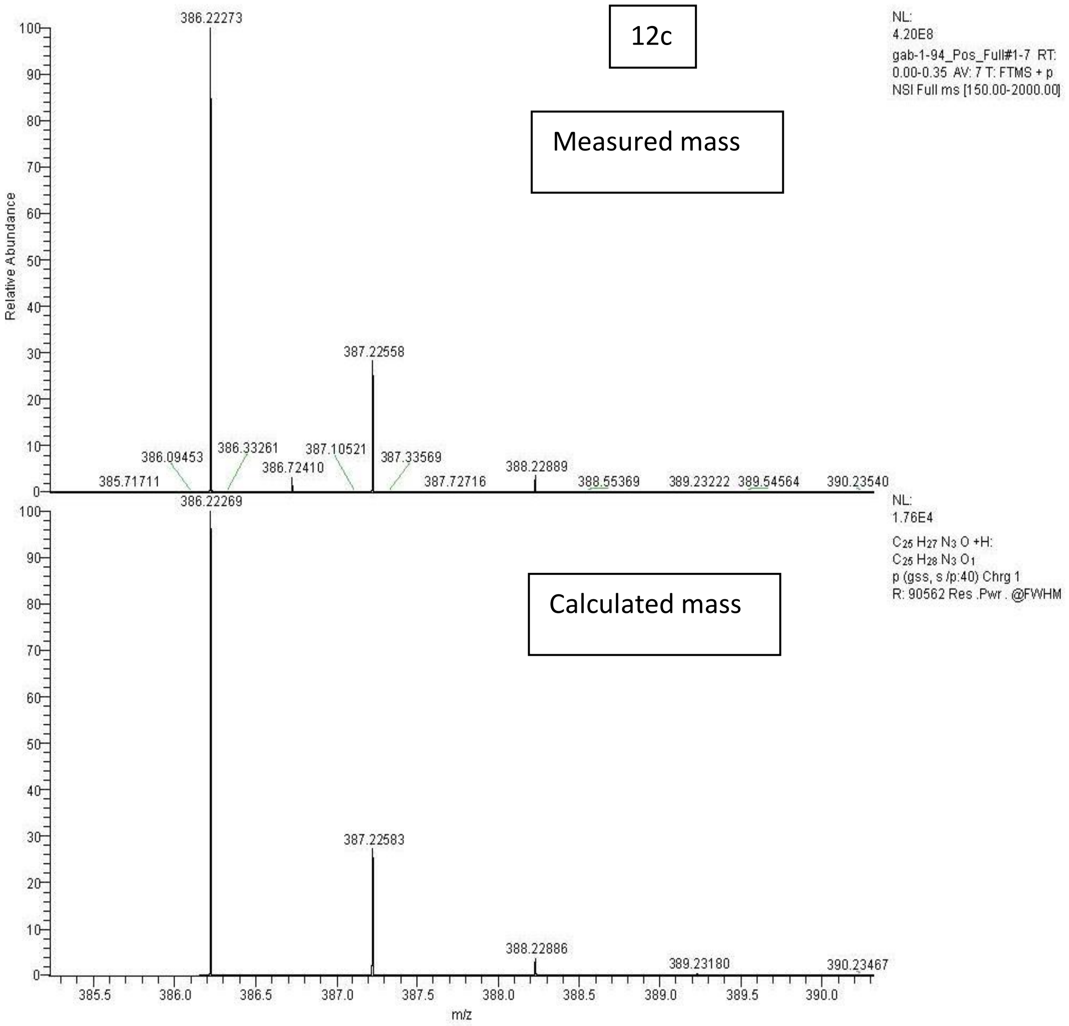

HRMS of 2-Amino-N-(naphthalen-2-ylmethyl)-N-phenethylacetamide (TFA salt) (12d)

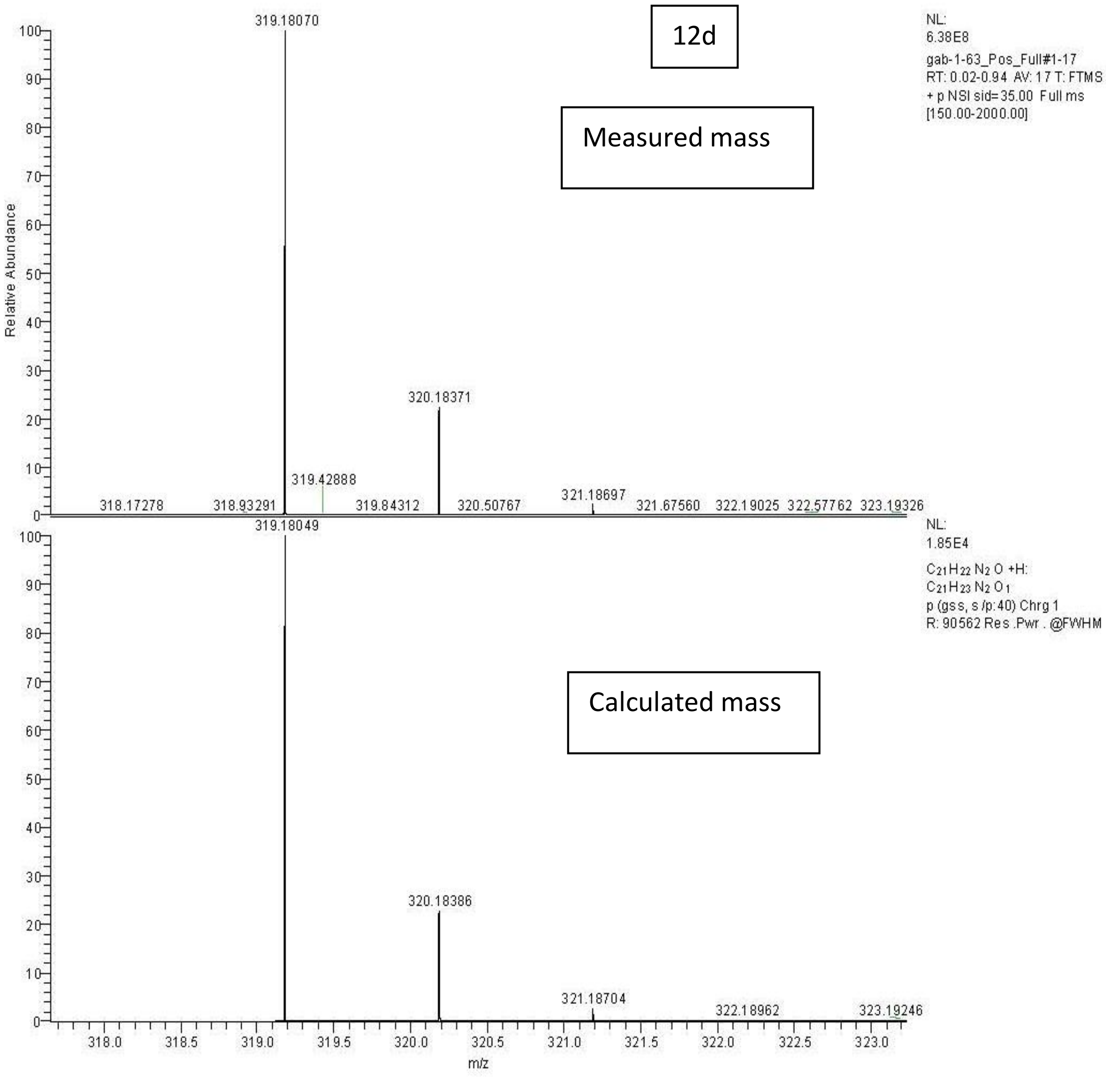

HRMS of 3-Amino-N-(naphthalen-2-ylmethyl)-N-phenethylpropanamide (TFA salt) (12e)

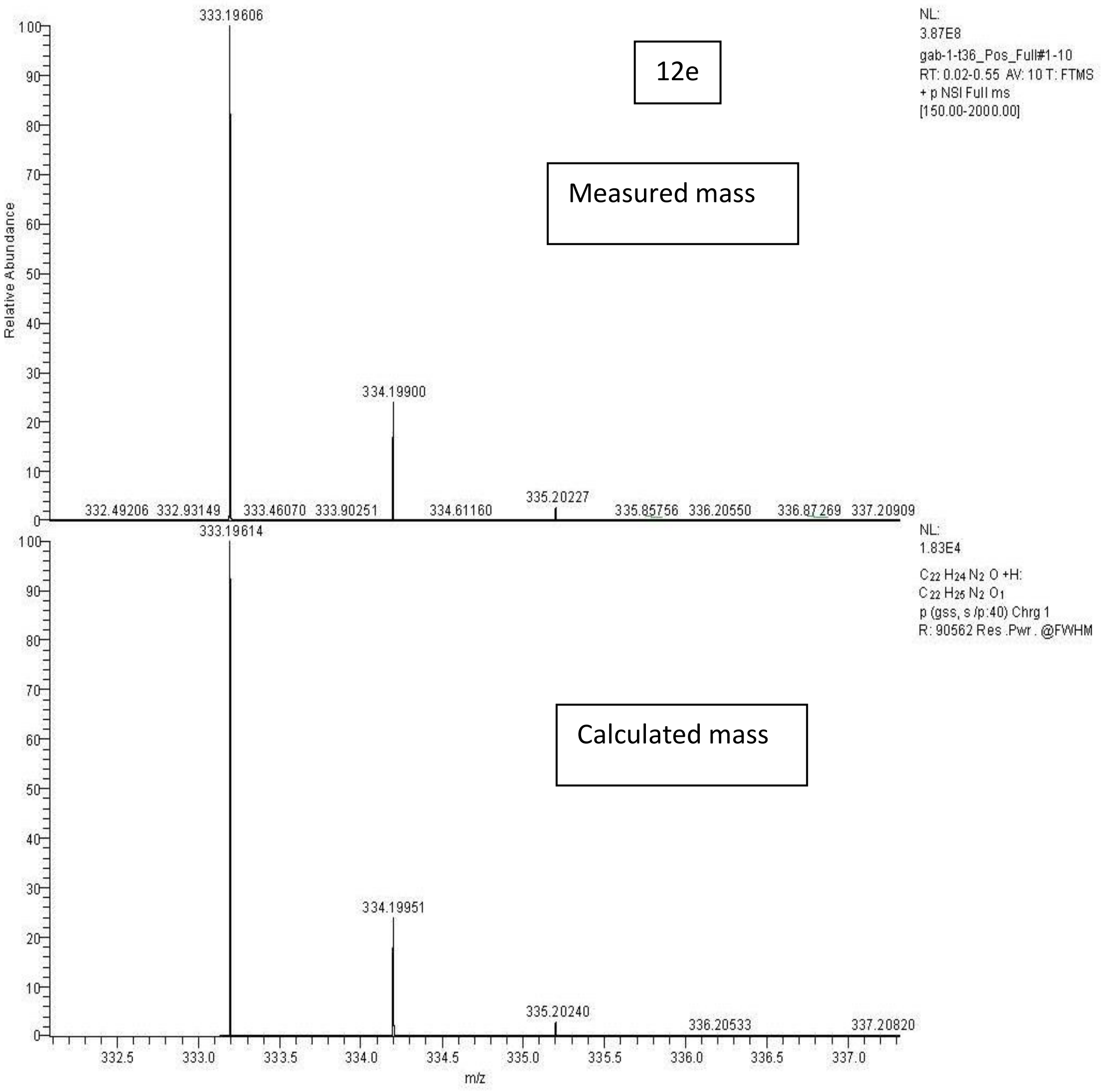

HRMS of 4-Amino-N-(naphthalen-2-ylmethyl)-N-phenethyl- butanamide (TFA salt) (12f)

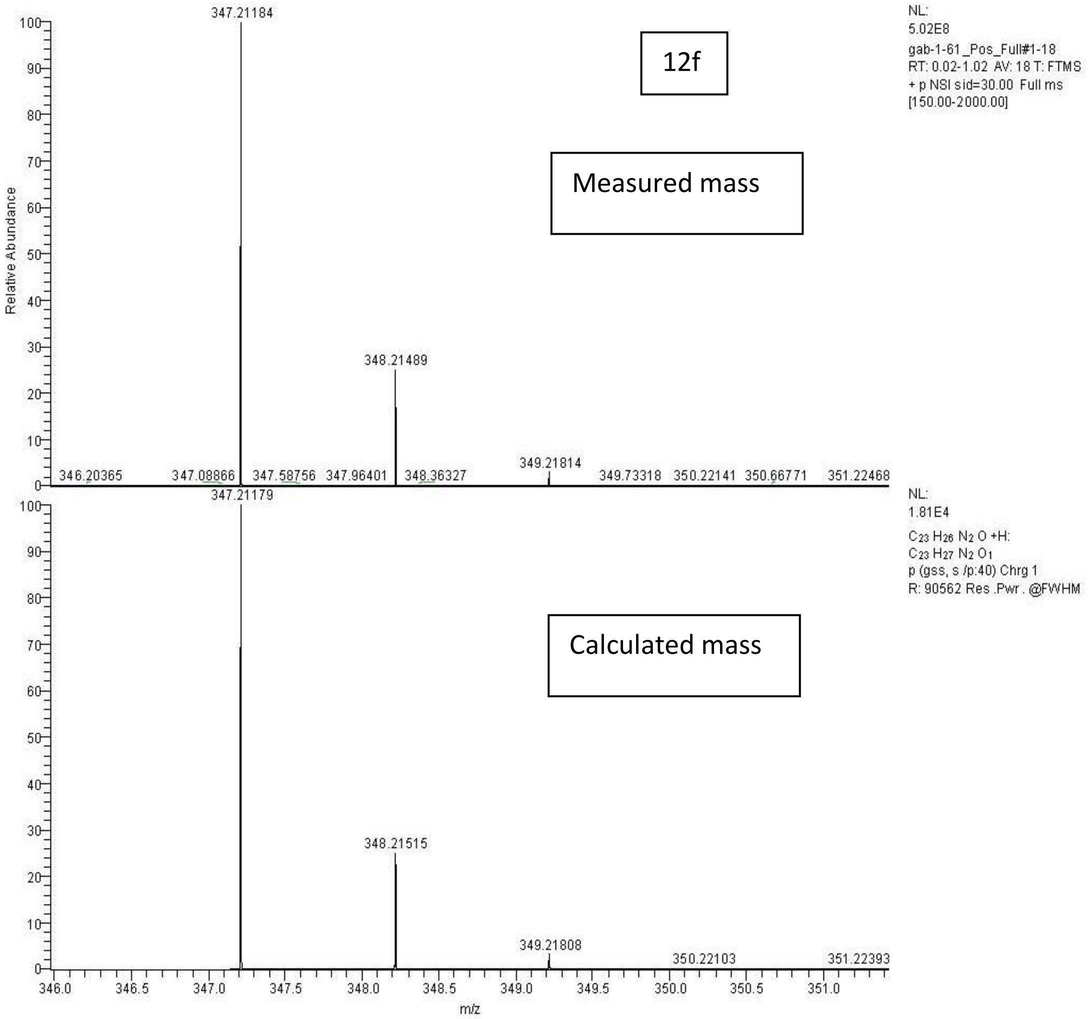

HRMS of N-(2-(1H-indol-3-yl) ethyl)-2-guanidino-N-(naphthalen-2-ylmethyl) acetamide (TFAsalt)(13a)

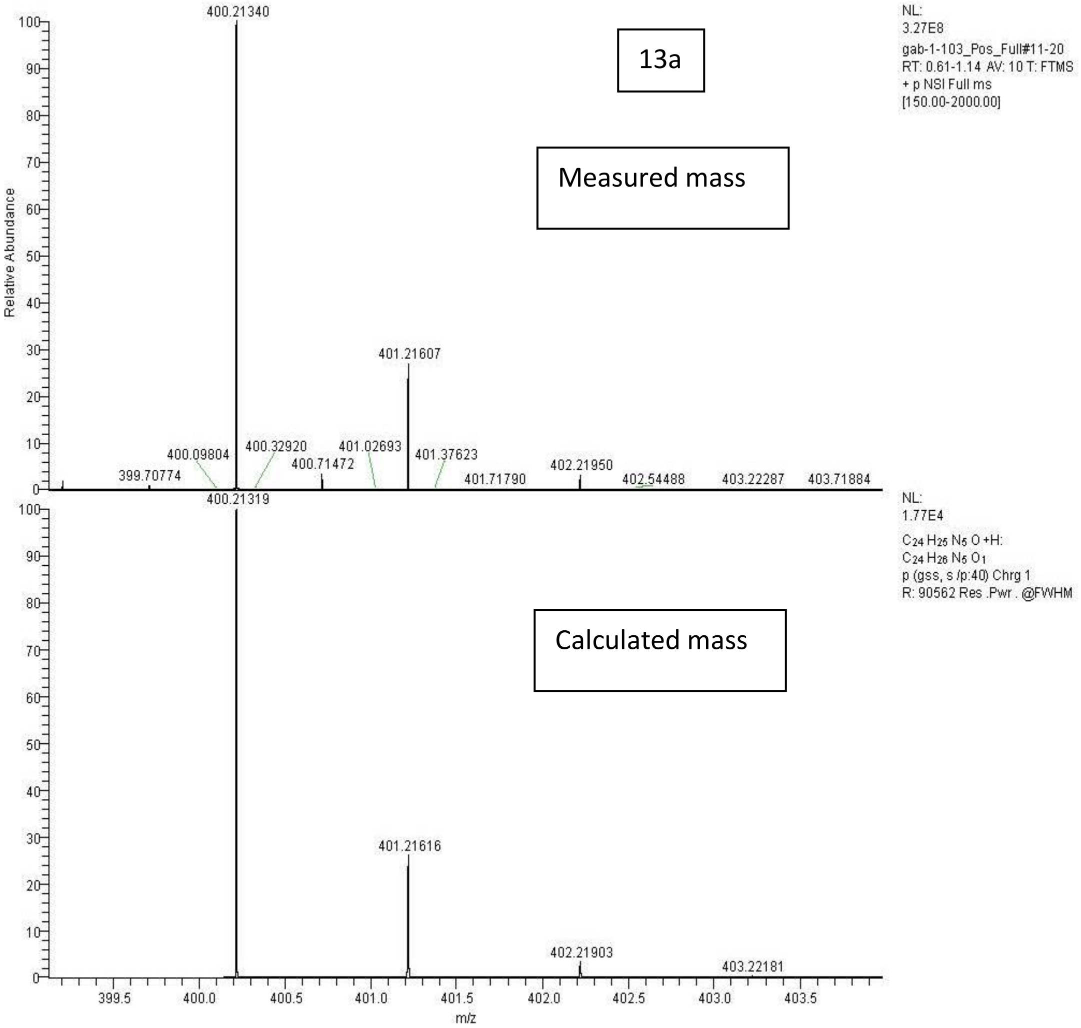

HRMS of N-(2-(1H-indol-3-yl) ethyl)-3-guanidino-N-(naphthalen-2-ylmethyl) propenamide (TFA salt) (13b)

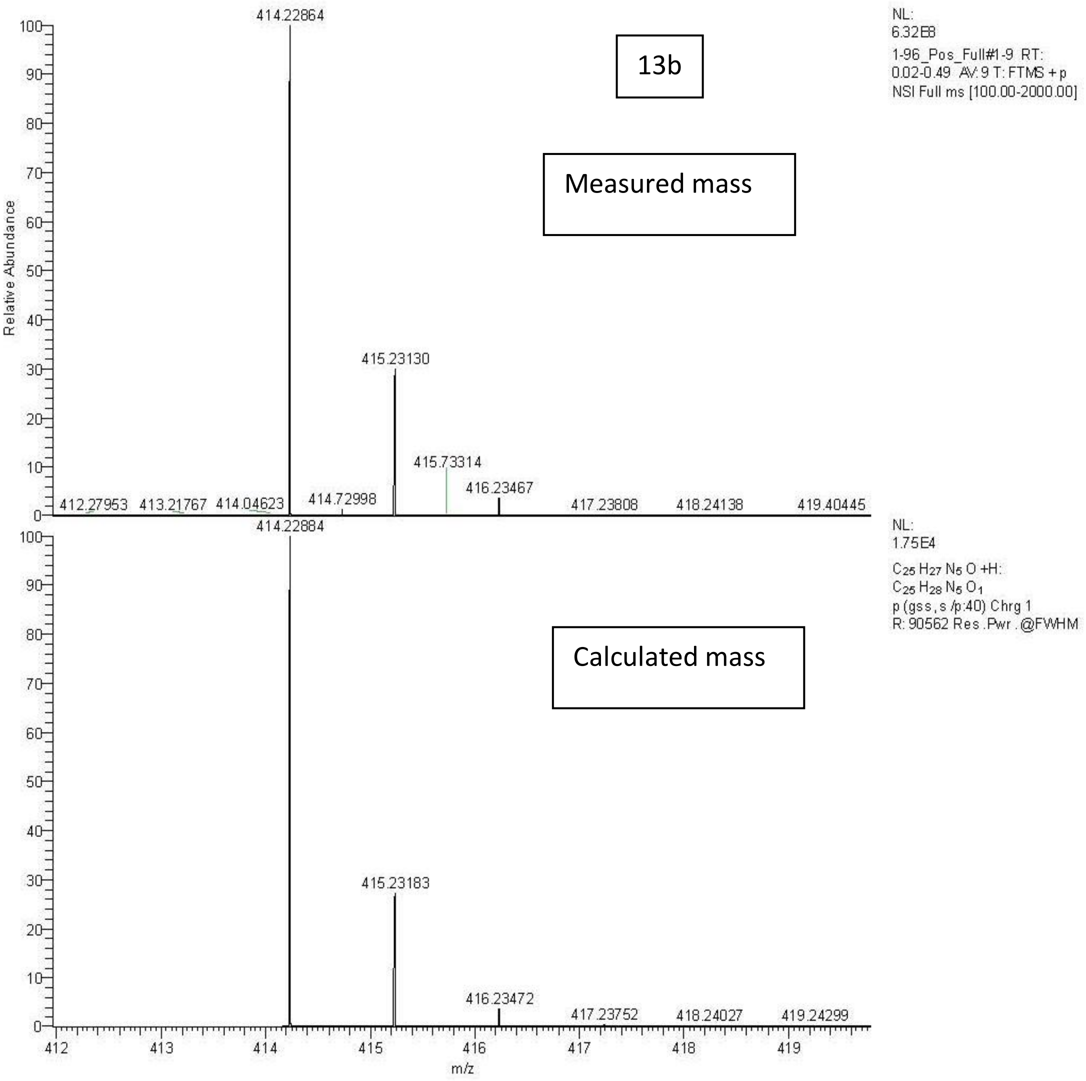

HRMS of N-(2-(1H-indol-3-yl) ethyl)-4-guanidino-N-(naphthalen-2-ylmethyl) butanamide (TFA salt) (13c)

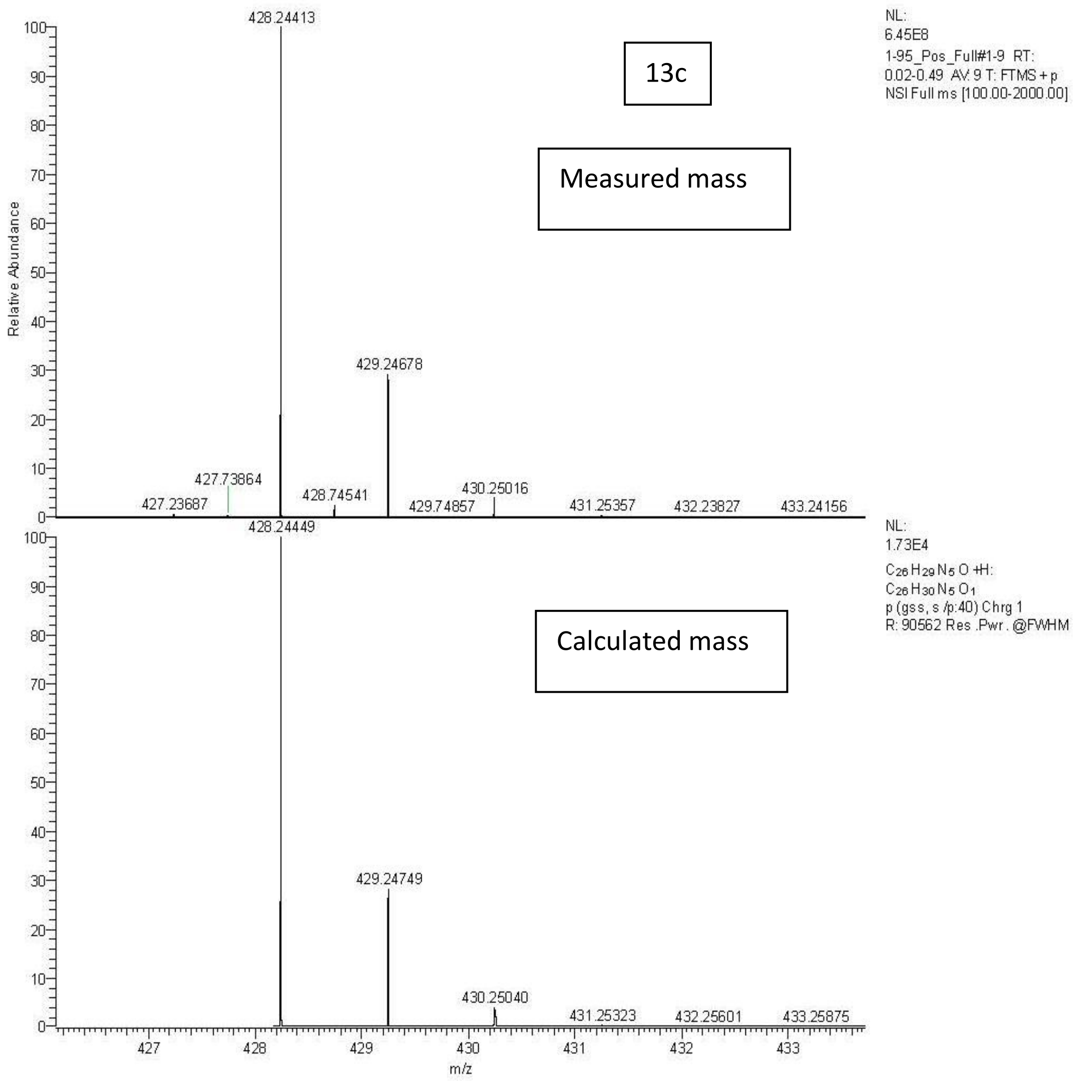

HRMS of 2-Guanidino-N-(naphthalen-2-ylmethyl)-N-phenethyl acetamide (TFA salt) (13d)

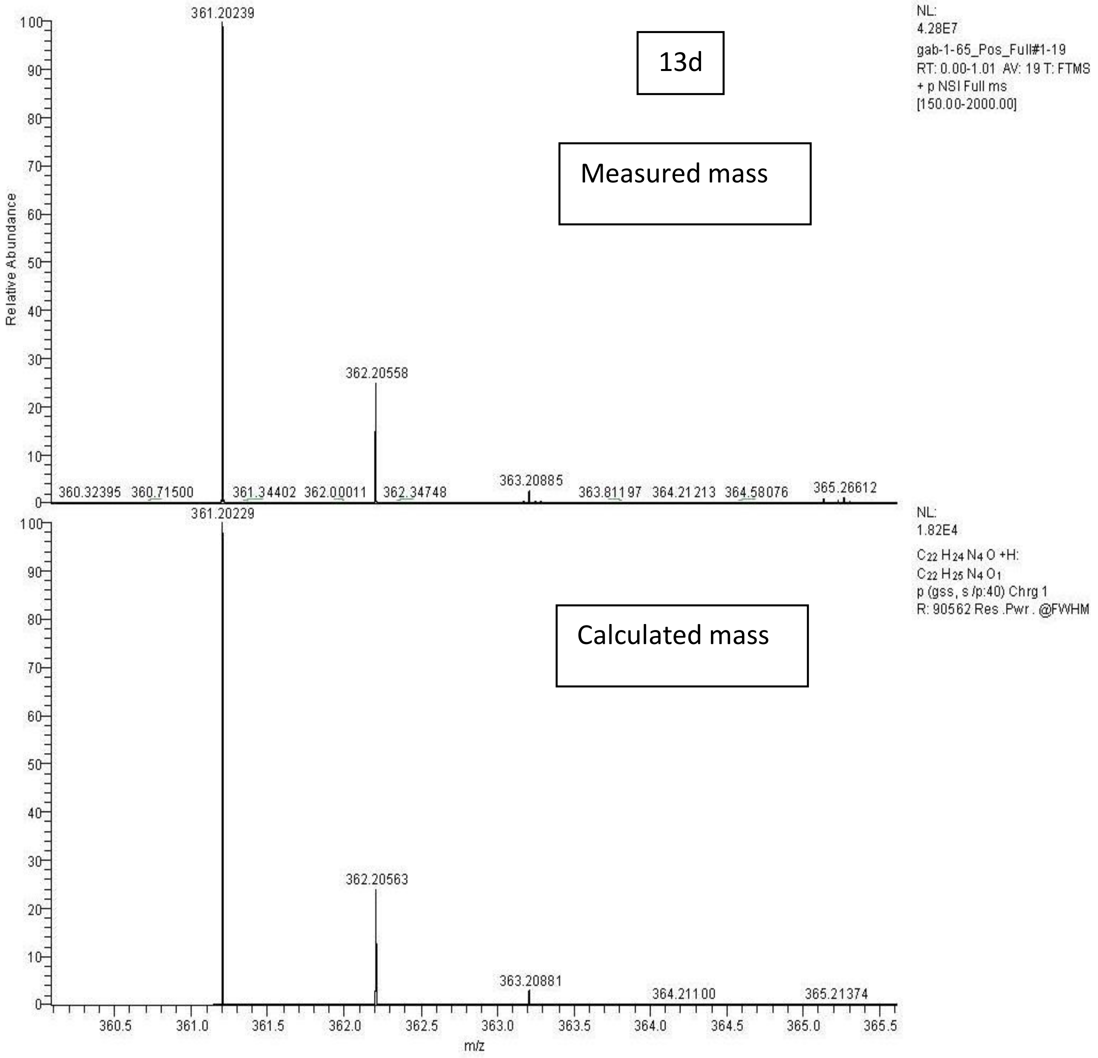

HRMS of 3-Guanidino-N-(naphthalen-2-ylmethyl)-N-phenethyl- propanamide (TFA salt) (13e)

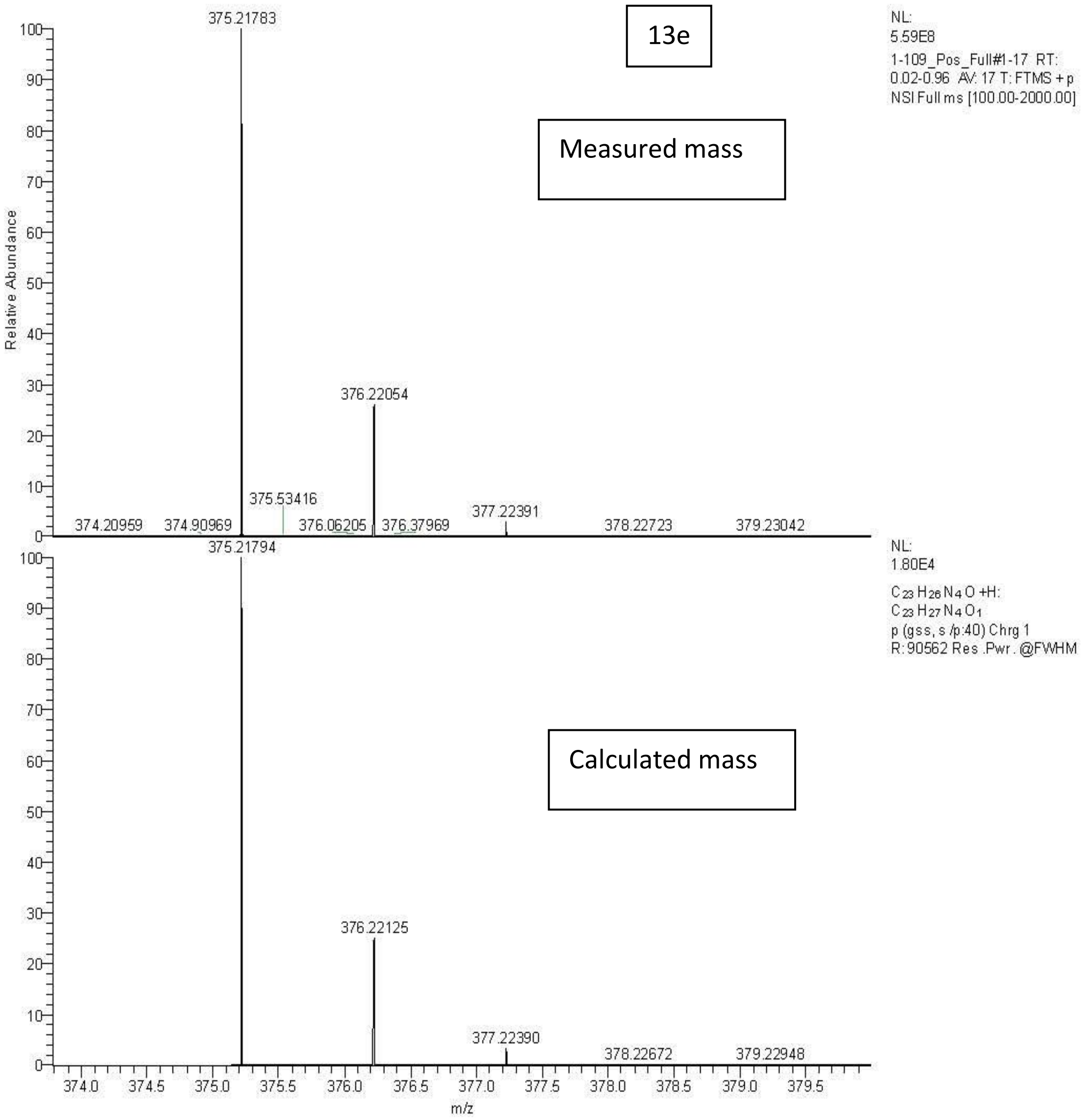

HRMS of 4-Guanidino-N-(naphthalen-2-ylmethyl)-N-phenethyl- butanamide (TFA salt) (13f)

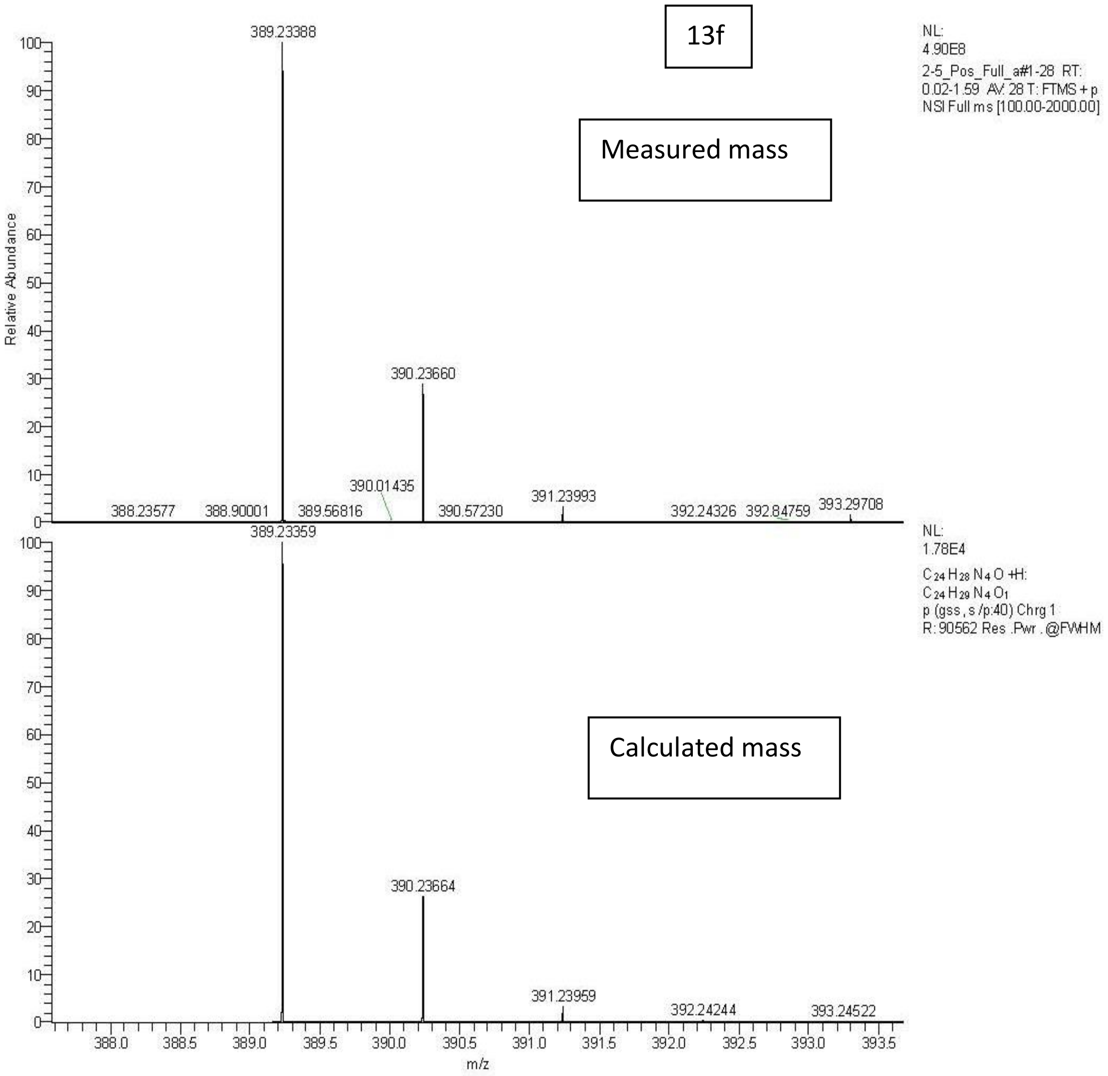

HRMS of N-(2-(1H-indol-3-yl) ethyl)-3-(dimethylamino)-N-(naphthalen-2-ylmethyl) propenamide (14a)

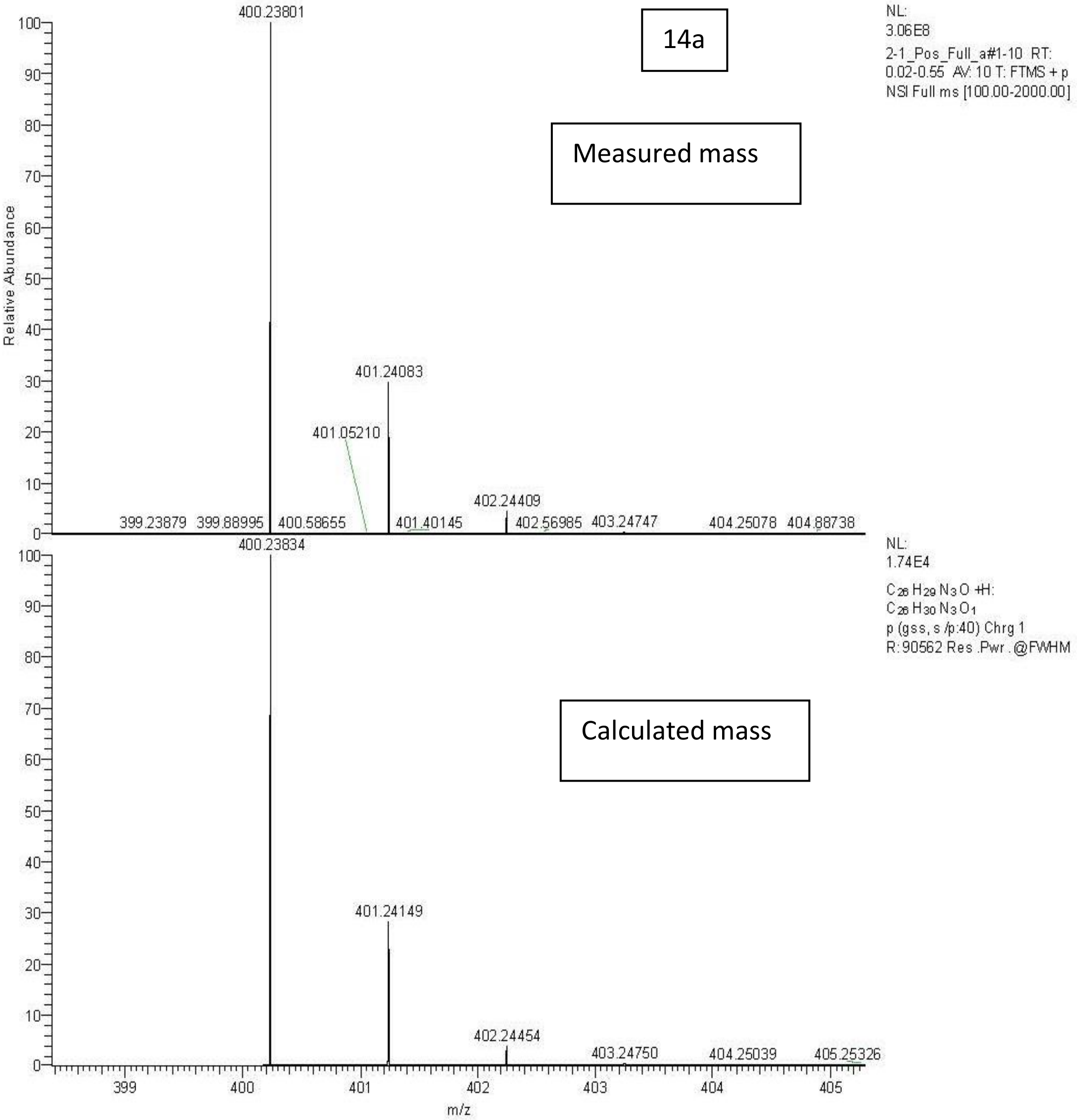

HRMS of N-(2-(1H-indol-3-yl) ethyl)-4-(dimethylamino)-N-(naphthalen-2-ylmethyl) butanamide (14b)

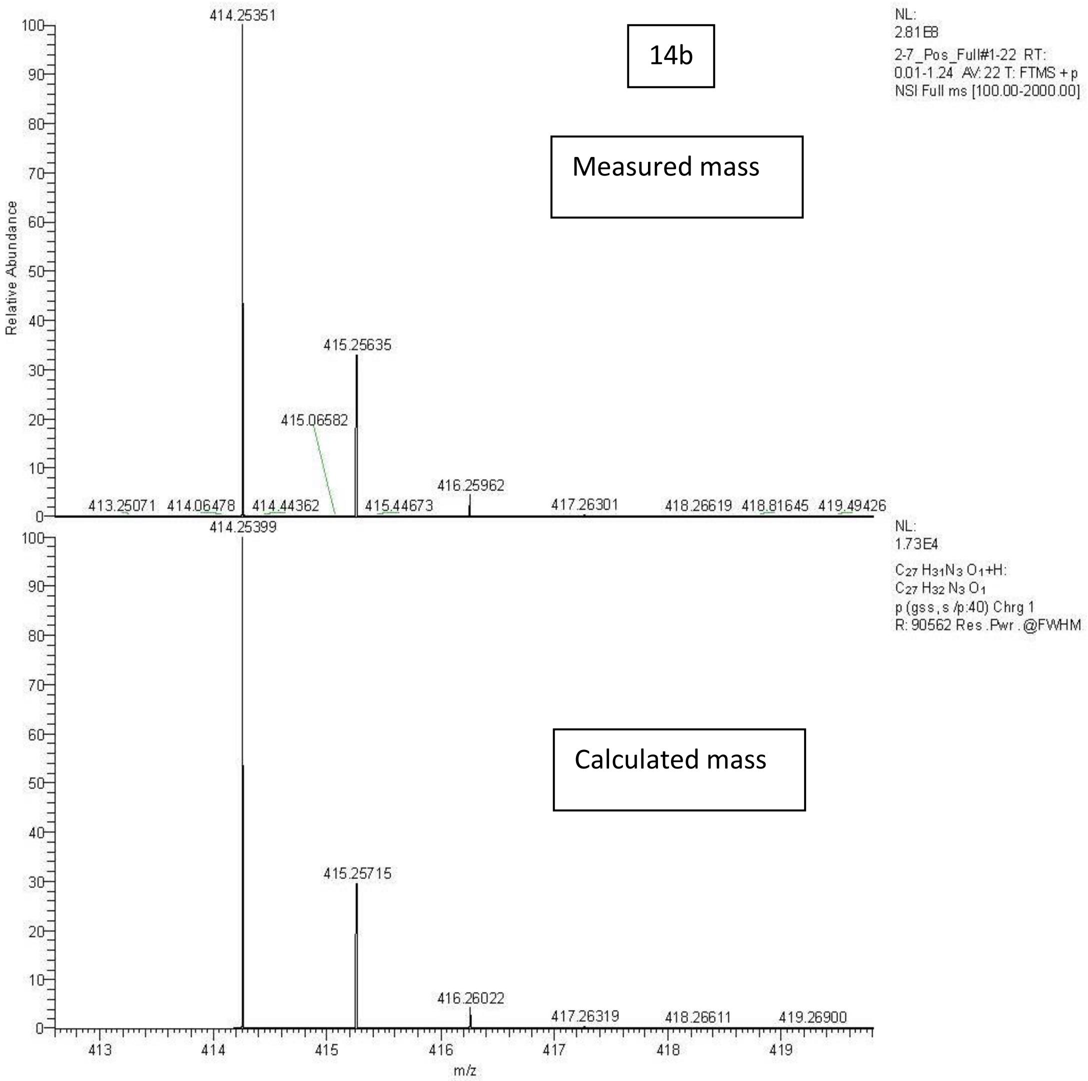

HRMS of 4-(Dimethylamino)-N-(naphthalen-2-ylmethyl)-N-phenethyl-butanamide (14c)

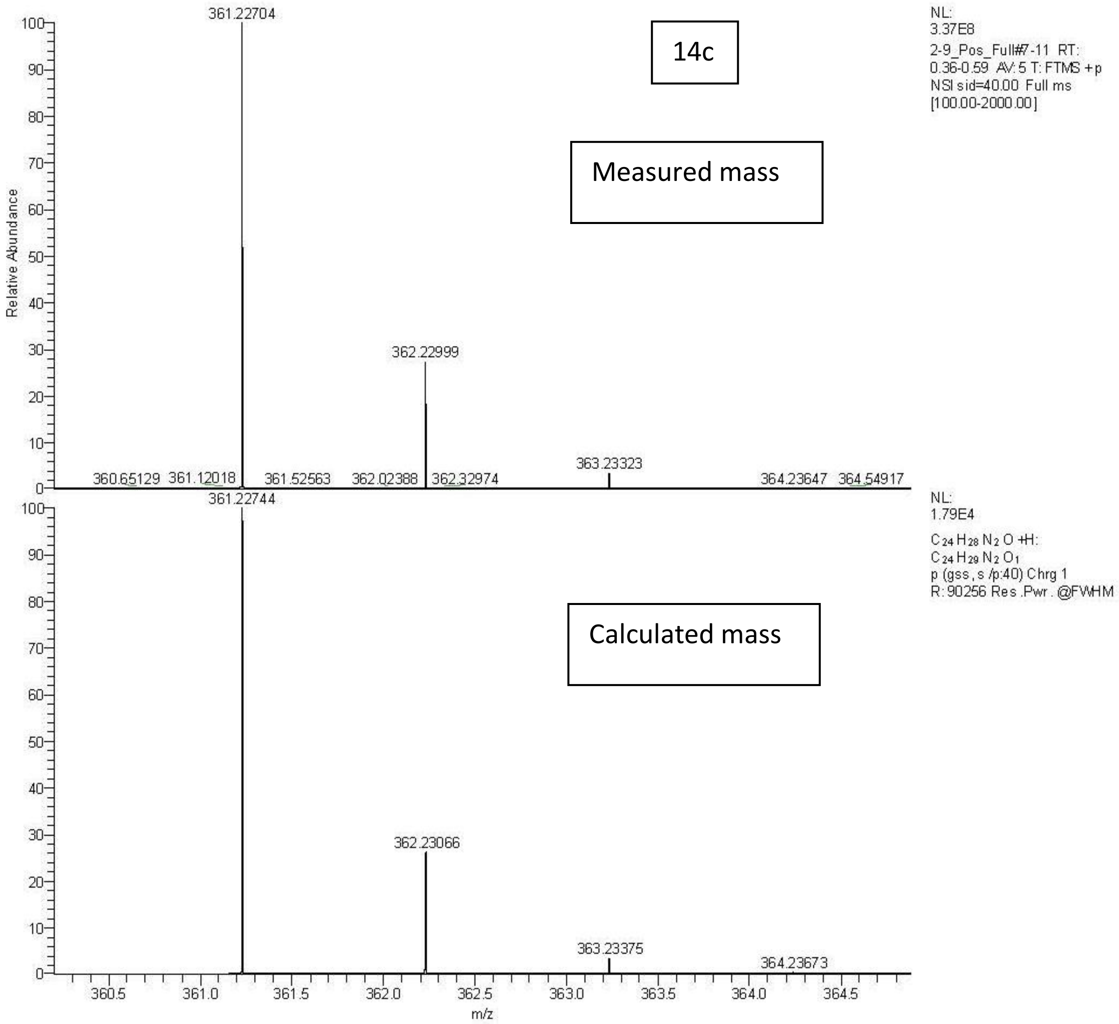

HRMS of 3-(Dimethylamino)-N-(naphthalen-2-ylmethyl)-N-phenethyl-propanamide (14d)

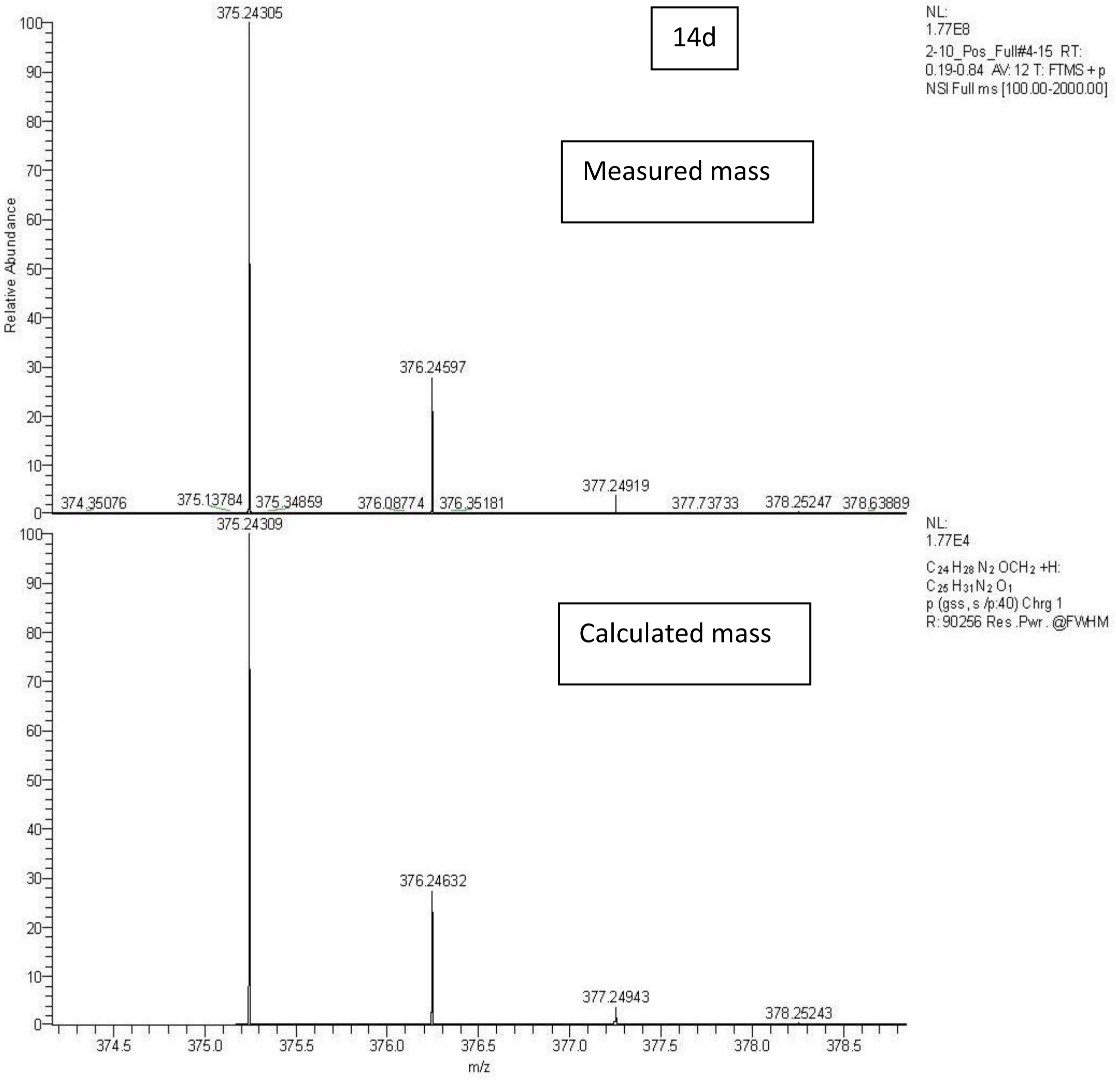

HRMS of N-(2-(1H-indol-3-yl) ethyl)-3-(dimethylamino)-N-(naphthalen-2-ylmethyl) propenamide (15a)

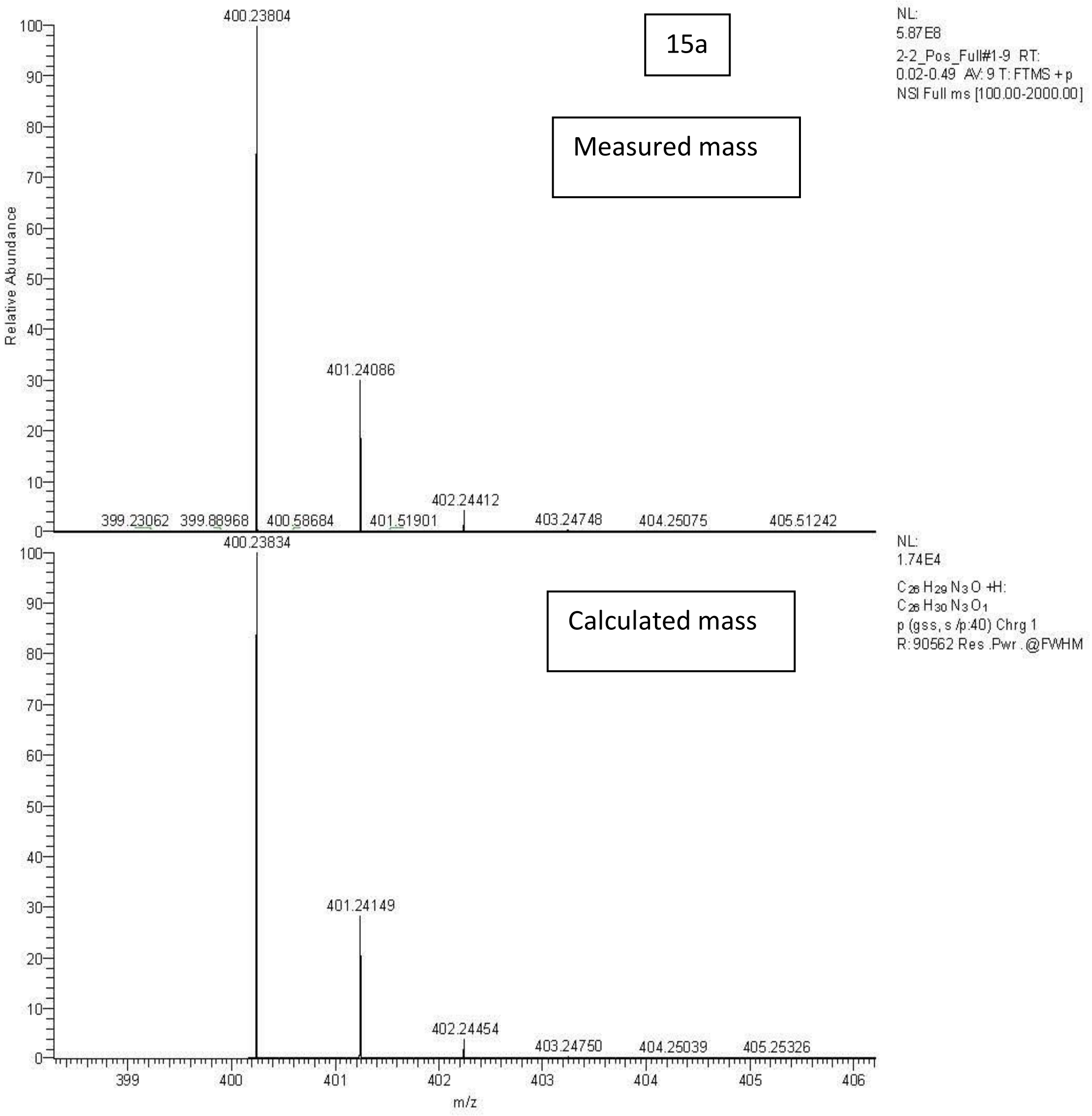

HRMS of N-(2-(1H-indol-3-yl) ethyl)-4-(dimethylamino)-N-(naphthalen-2-ylmethyl) butanamide (15b)

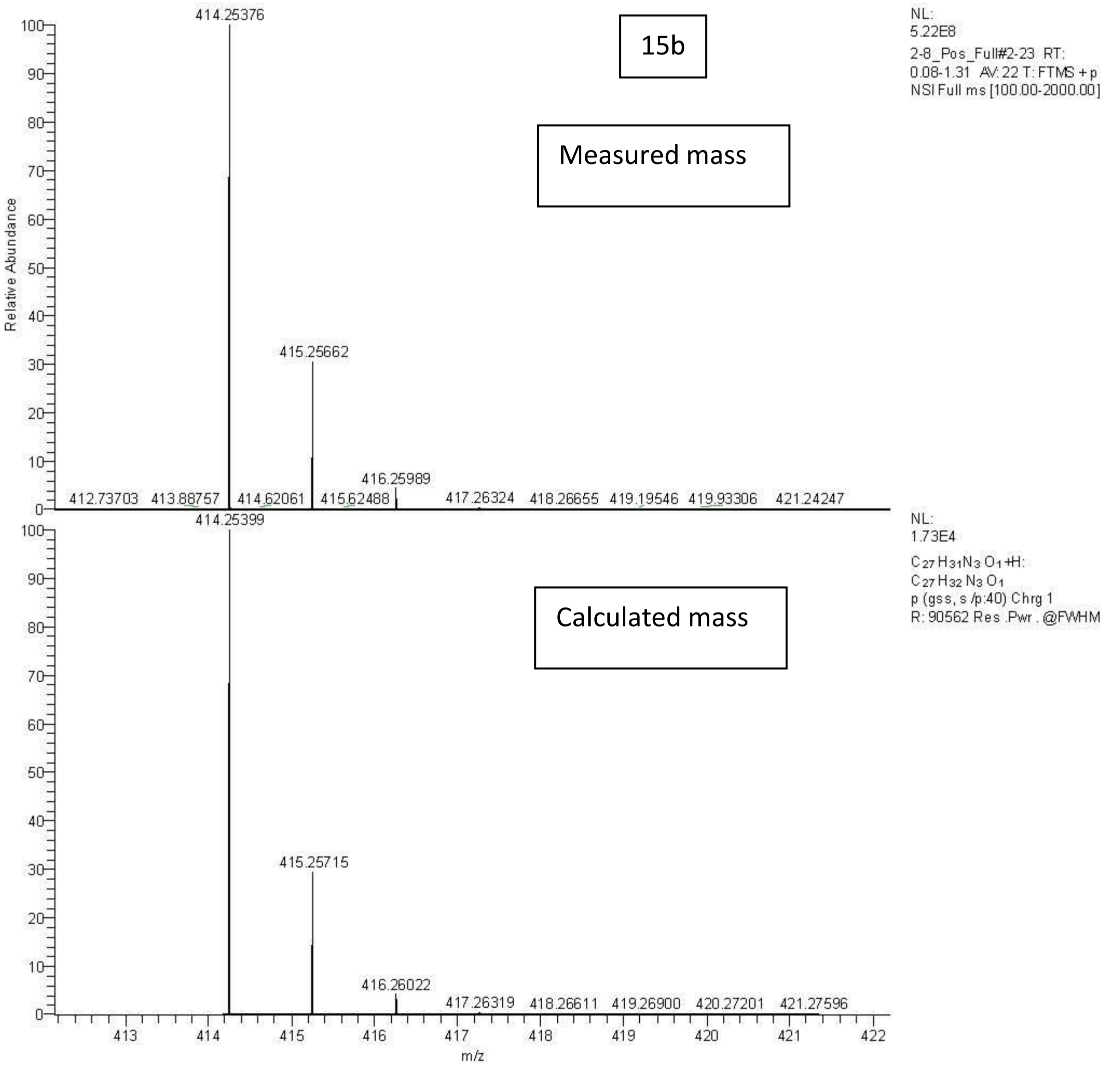

HRMS of 3-(Dimethylamino)-N-(naphthalen-2-ylmethyl)-N-phenethyl-propanamide (15c)

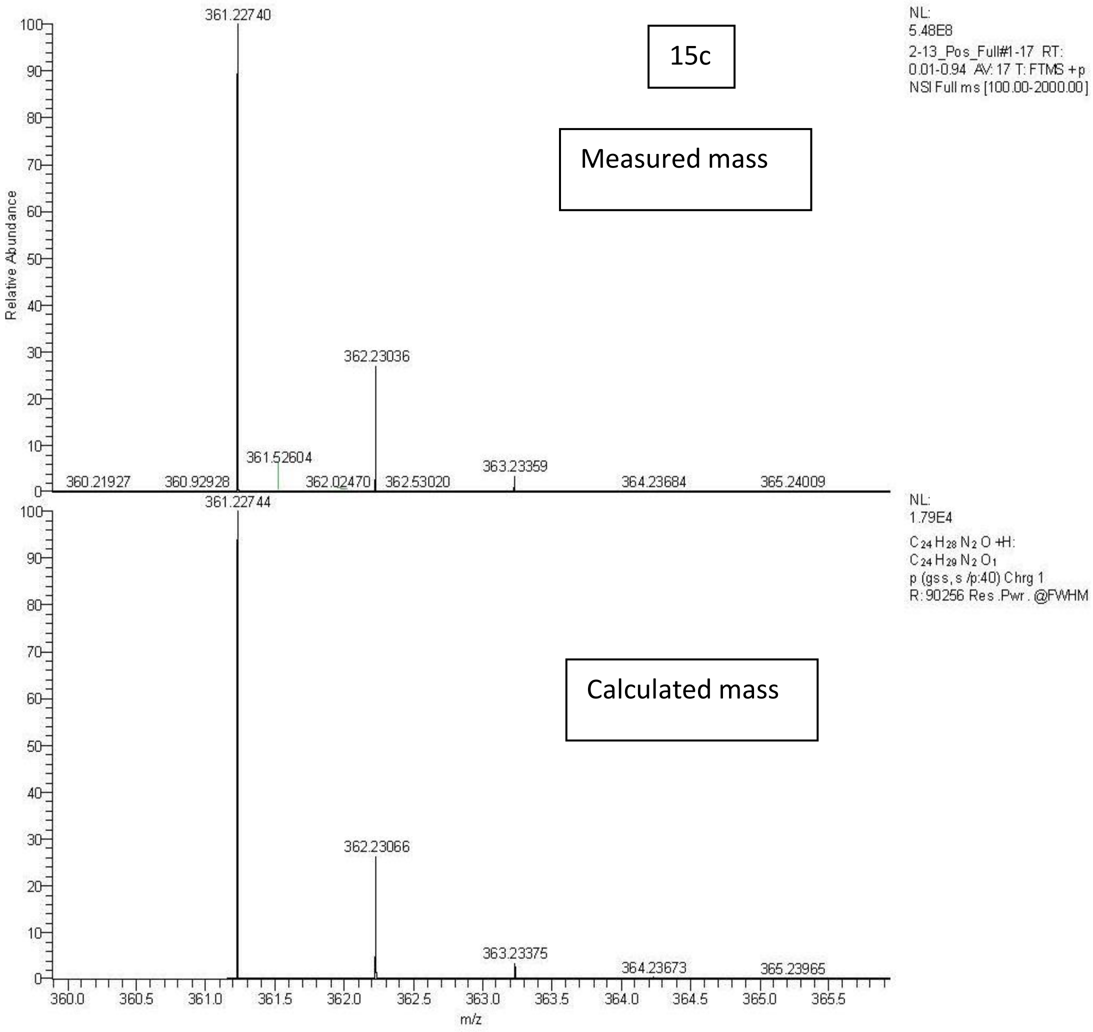

HRMS of 4-(Dimethylamino)-N-(naphthalen-2-ylmethyl)-N-phenethyl-butanamide (15d)

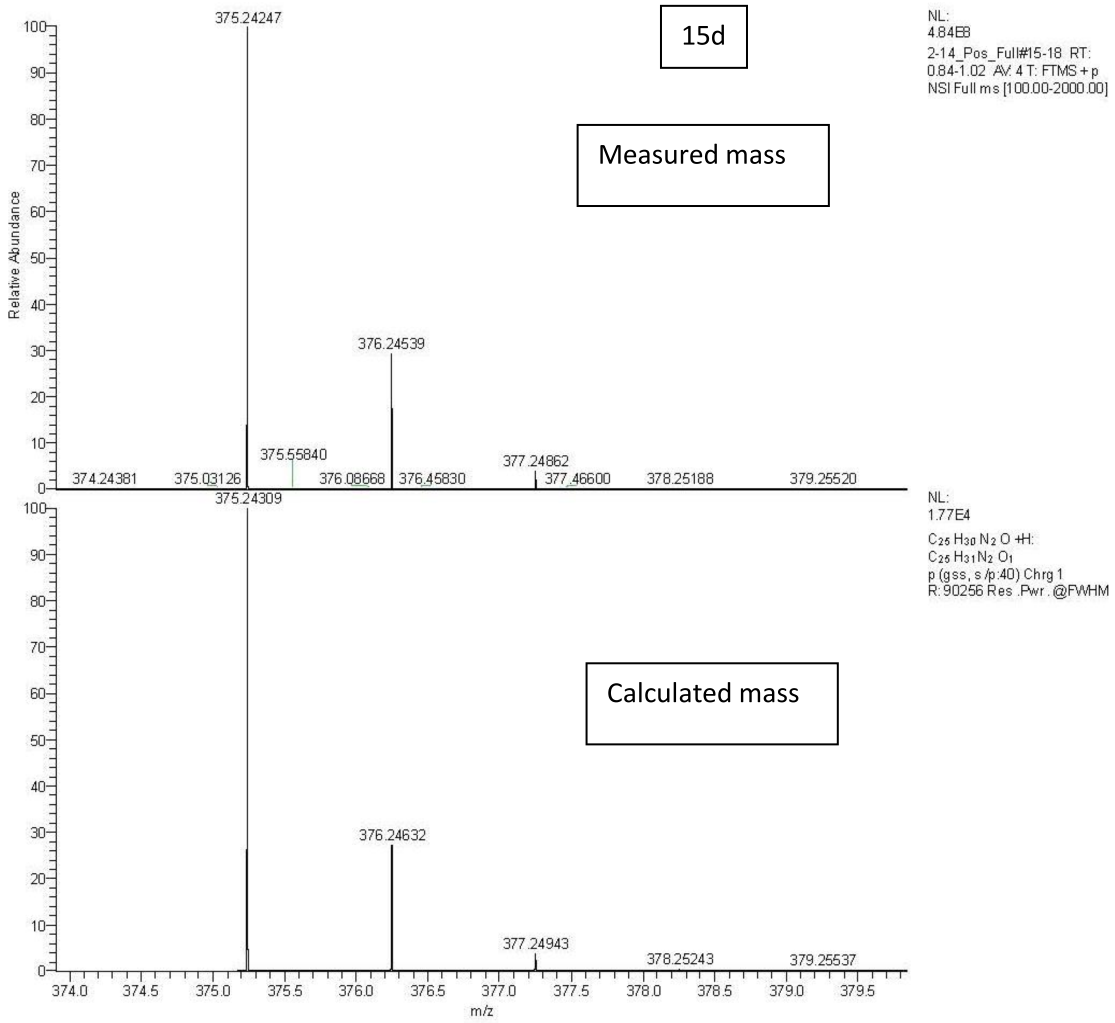

HRMS of 3-((2-(1H-indol-3-yl) ethyl) (naphthalen-2-ylmethyl) amino)-N,N,N-trimethyl-3-oxopropan-1-aminium (16a)

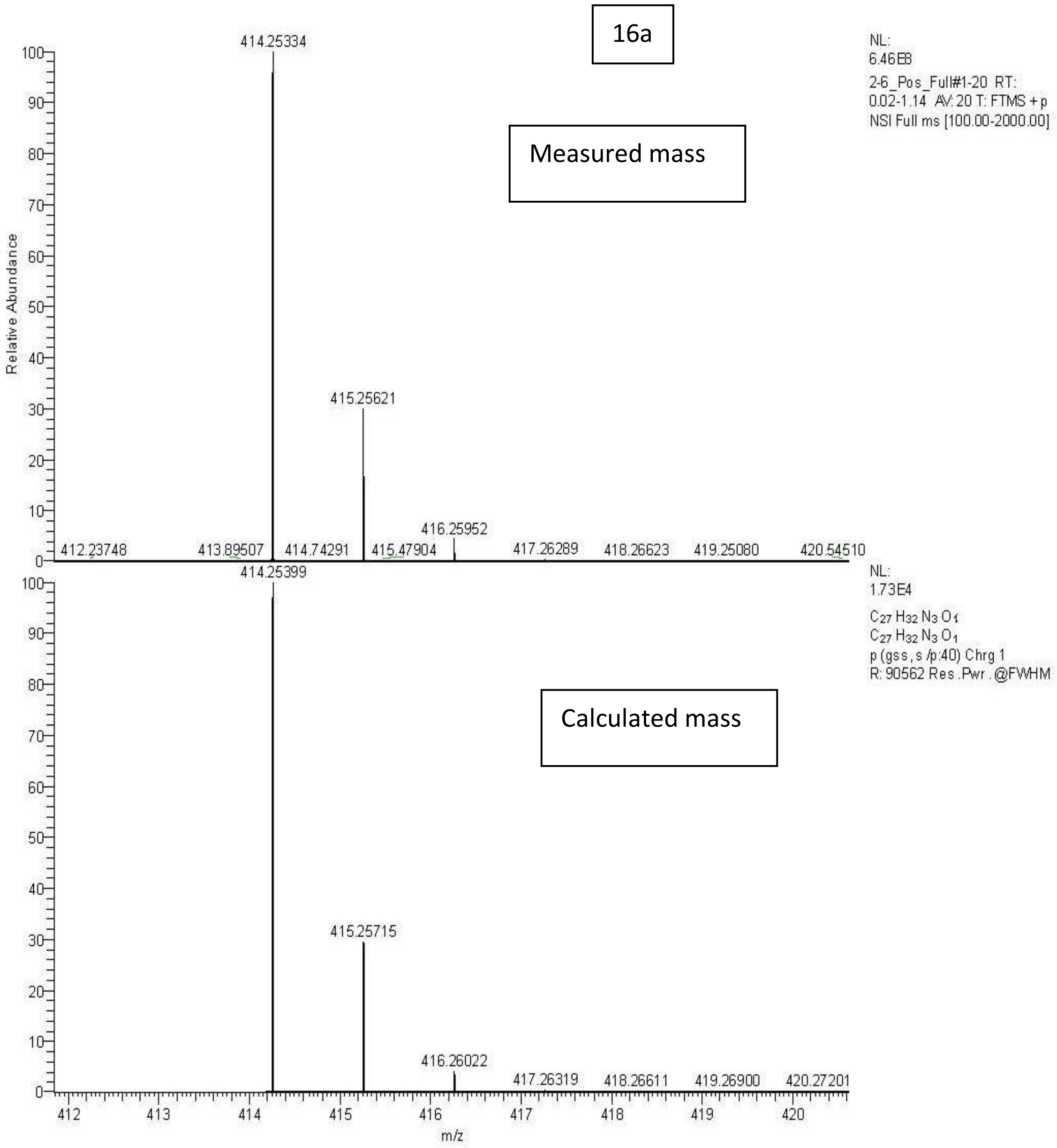

HRMS of 4-((2-(1H-indol-3-yl) ethyl) (naphthalen-2-ylmethyl) amino)-N,N,N-trimethyl-4-oxobutan-1-aminium (16b)

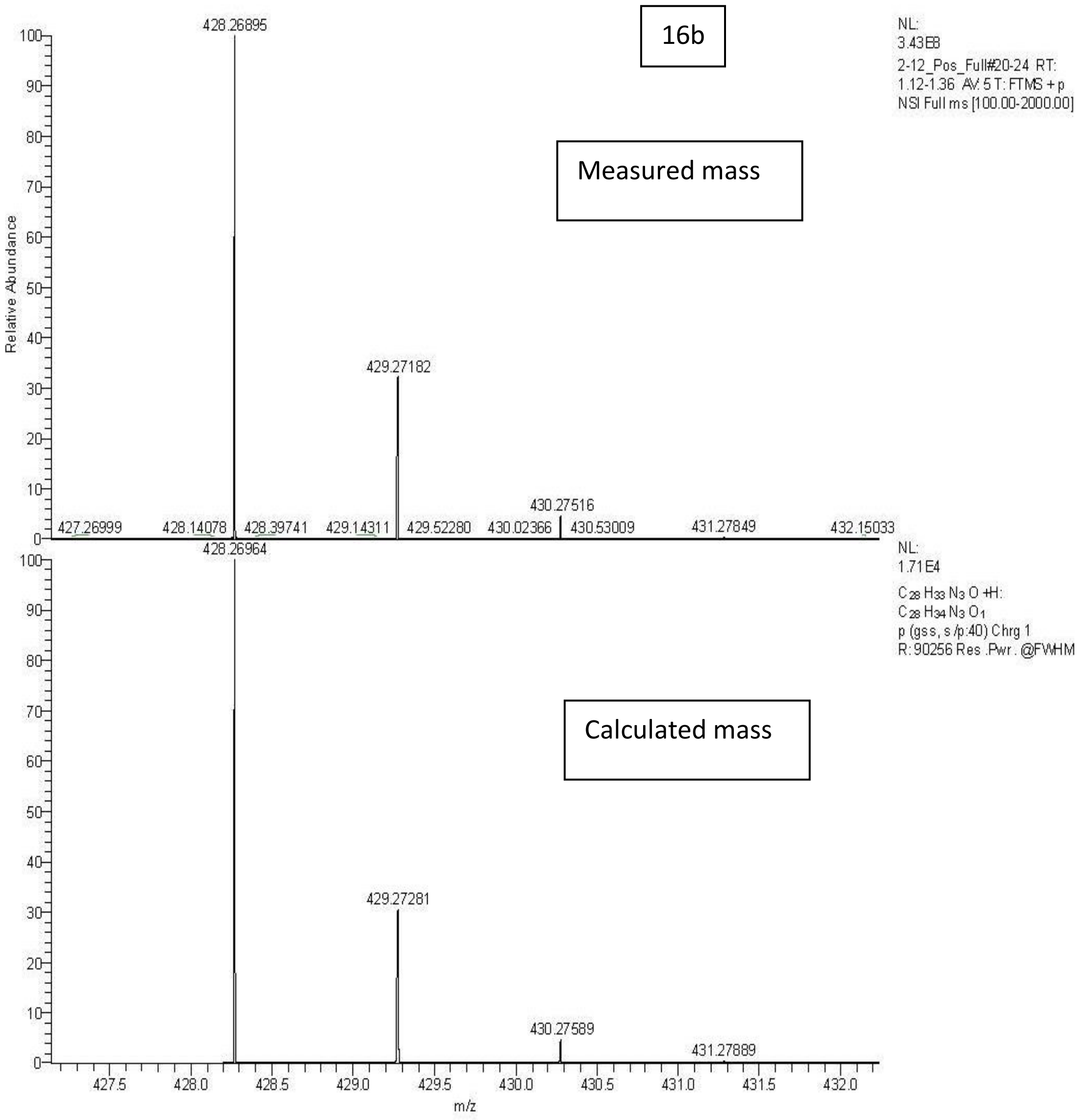

HRMS of N,N,N-Trimethyl-3-((naphthalen-2-ylmethyl)(phenethyl) amino)-3-oxopropan-1-aminium (16c)

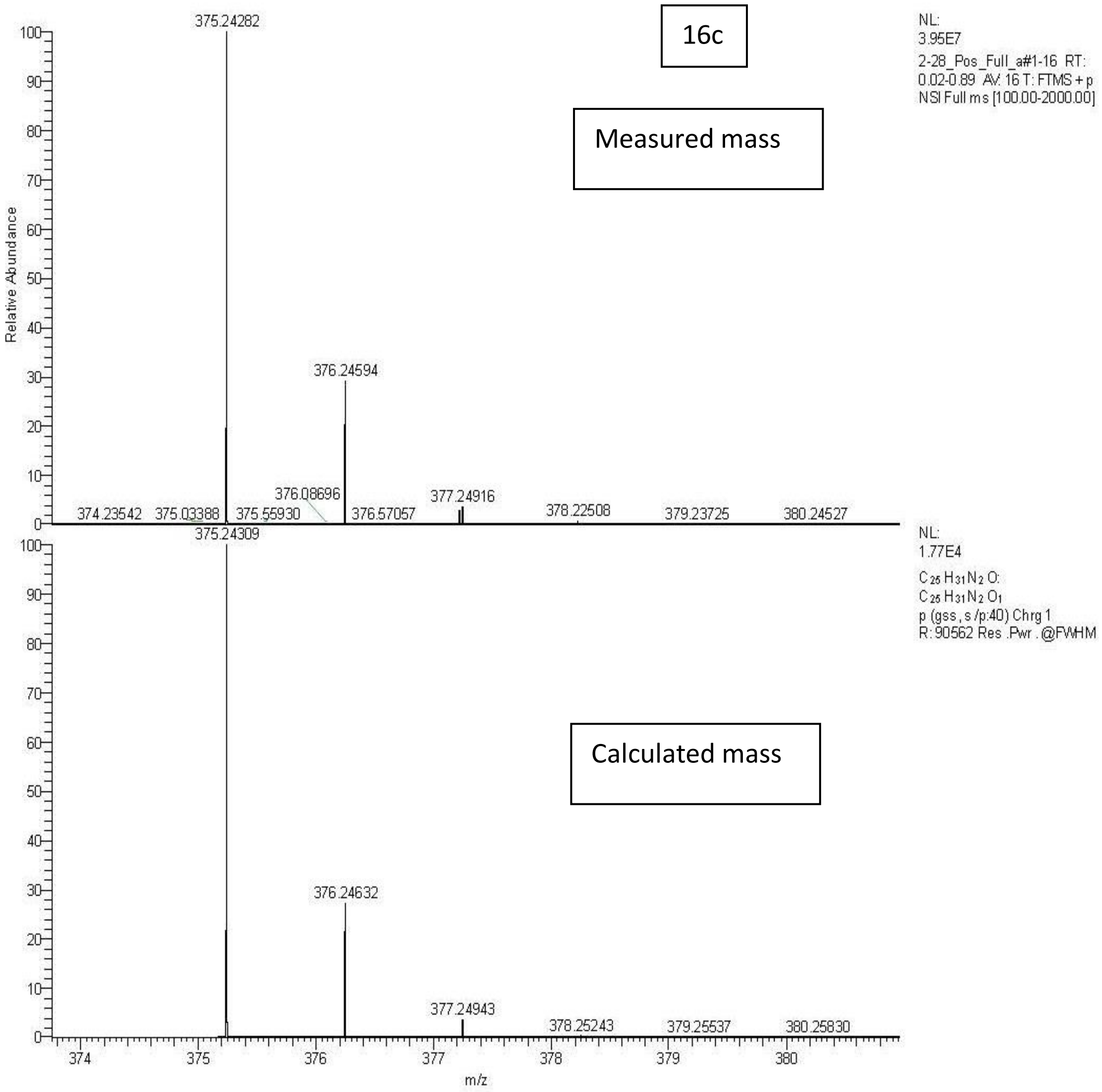

HRMS of N,N,N-Trimethyl-4-((naphthalen-2-ylmethyl)(phenethyl) amino)-4-oxobutan-1-aminium (16d)

16d

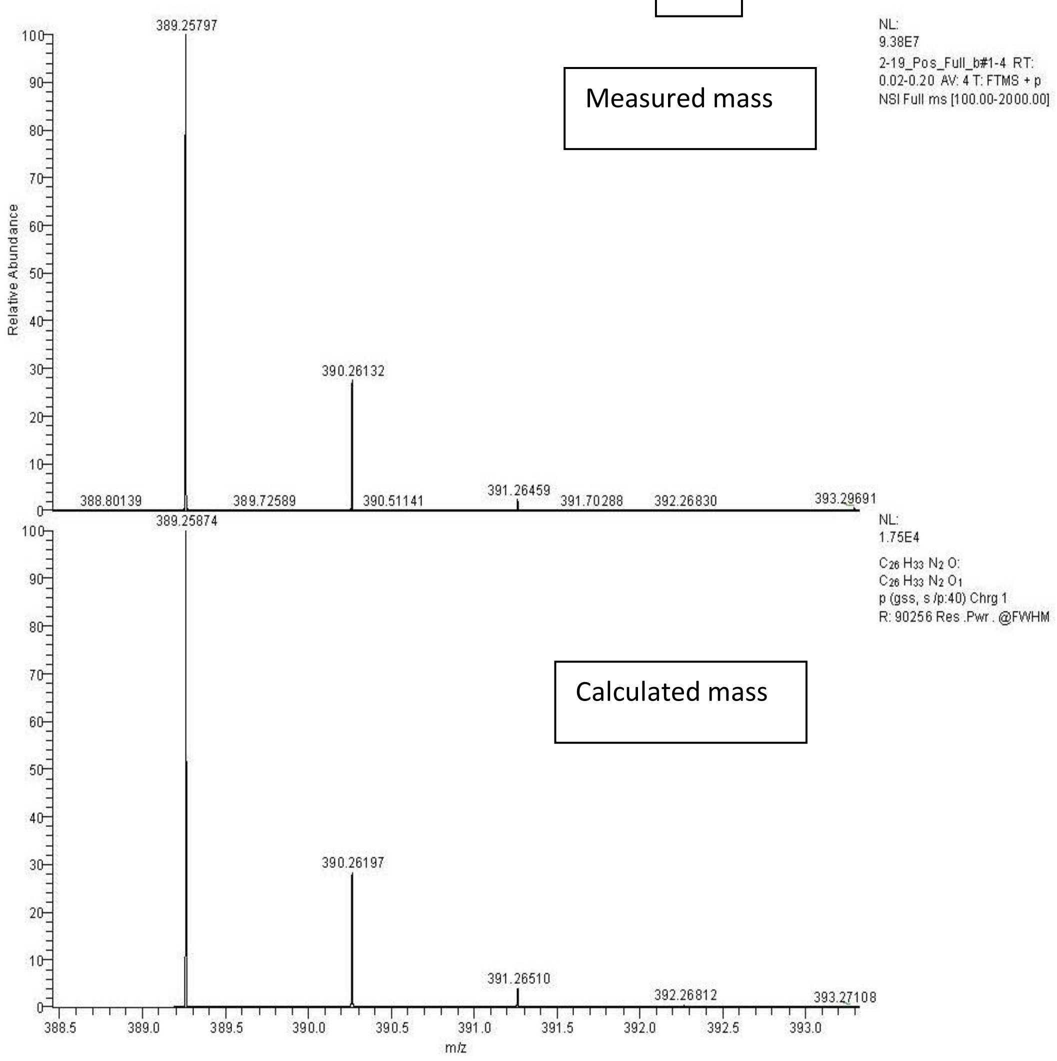

HRMS of Di-tert-butyl(5-((2-(1H-indol-3-yl) ethyl) (naphthalen-2-ylmethyl) amino)-5-oxopentane-1,4-diyl) (S)-dicarbamate (18a)

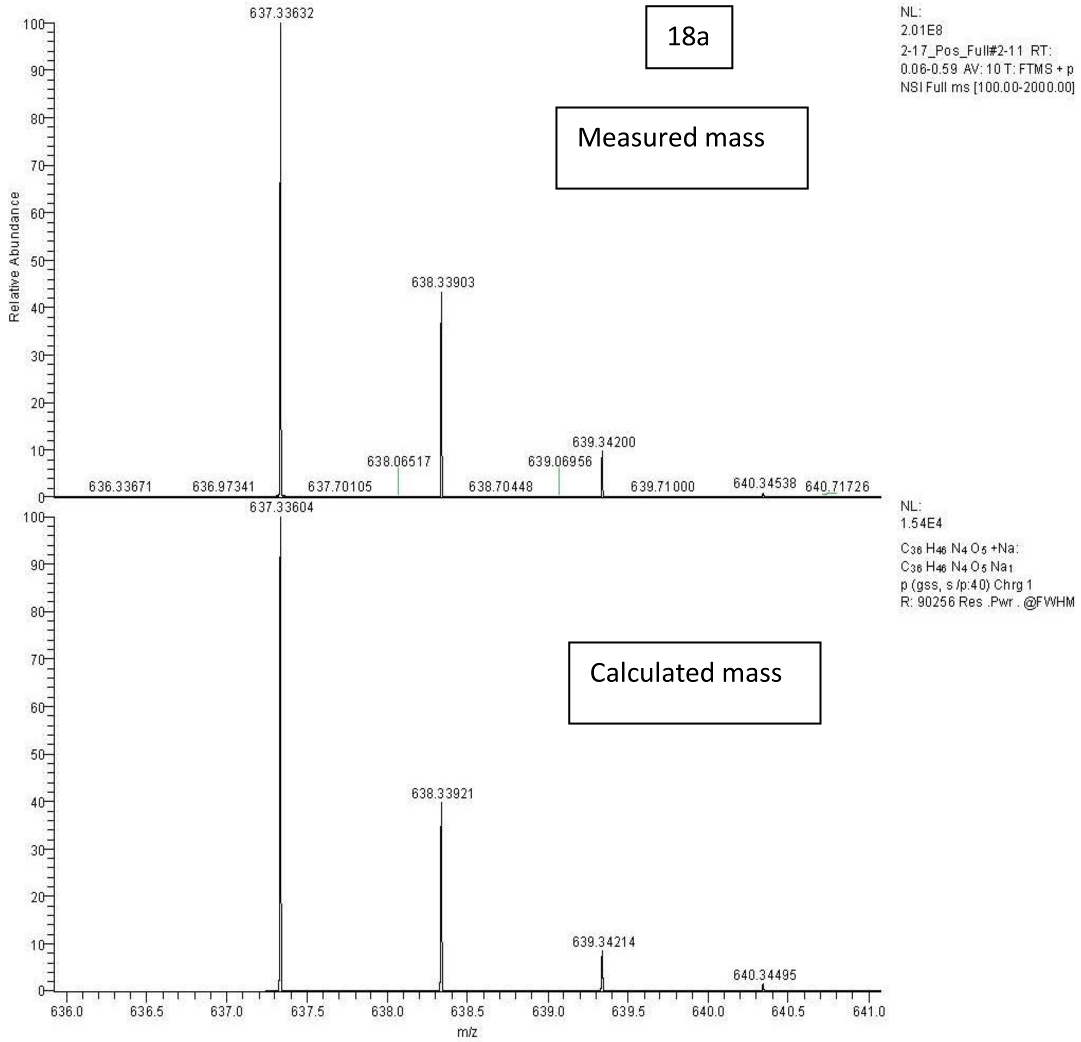

HRMS of Di-tert-butyl(6-((2-(1H-indol-3-yl) ethyl) (naphthalen-2-ylmethyl) amino)-6-oxohexane-1,5-diyl) (S)-dicarbamate (18b)

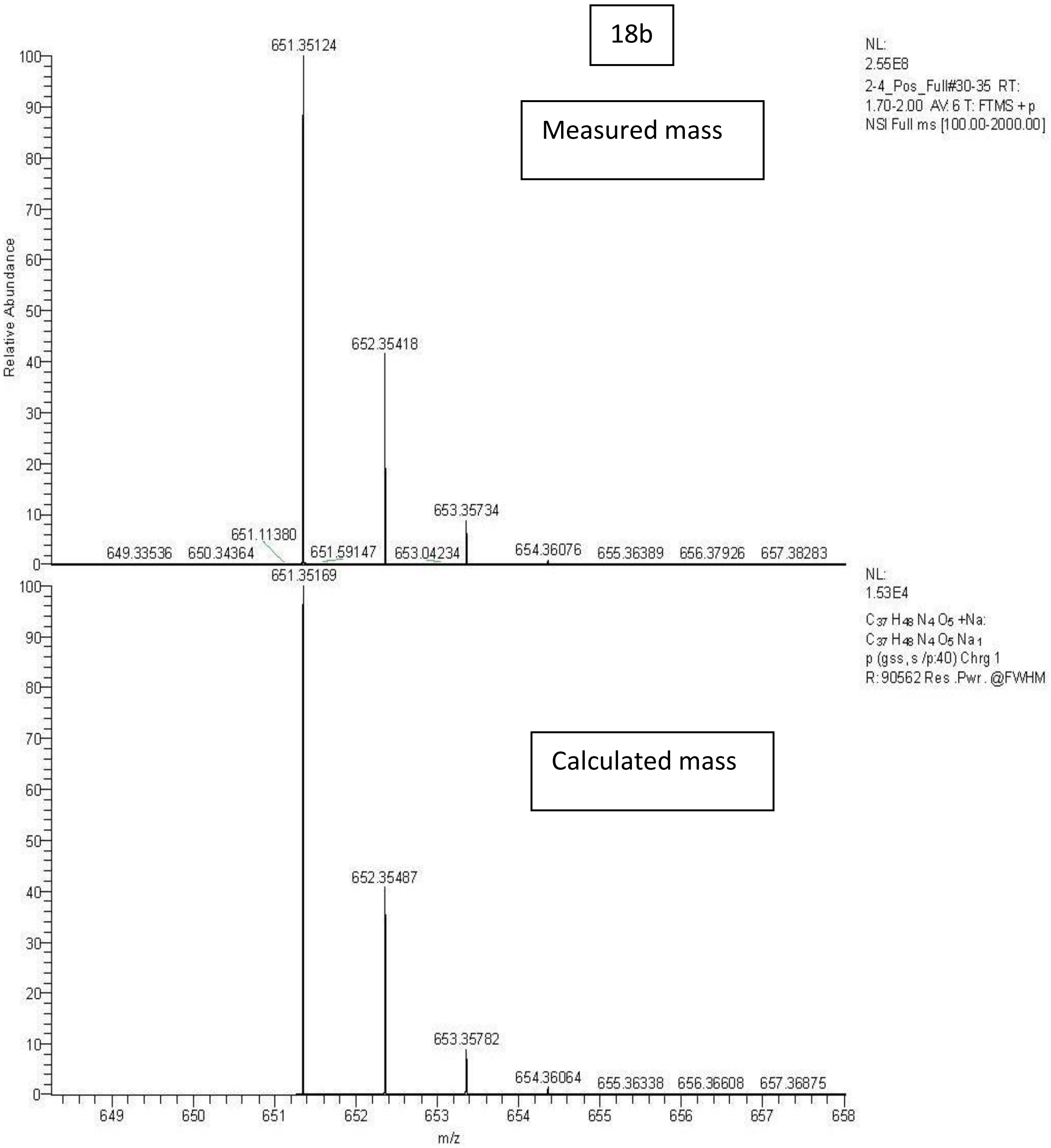

HRMS of (S)-N-(2-(1H-indol-3-yl) ethyl)-2,5-diamino-N-(naphthalen-2-ylmethyl) pentanamide (TFA salt) (19a)

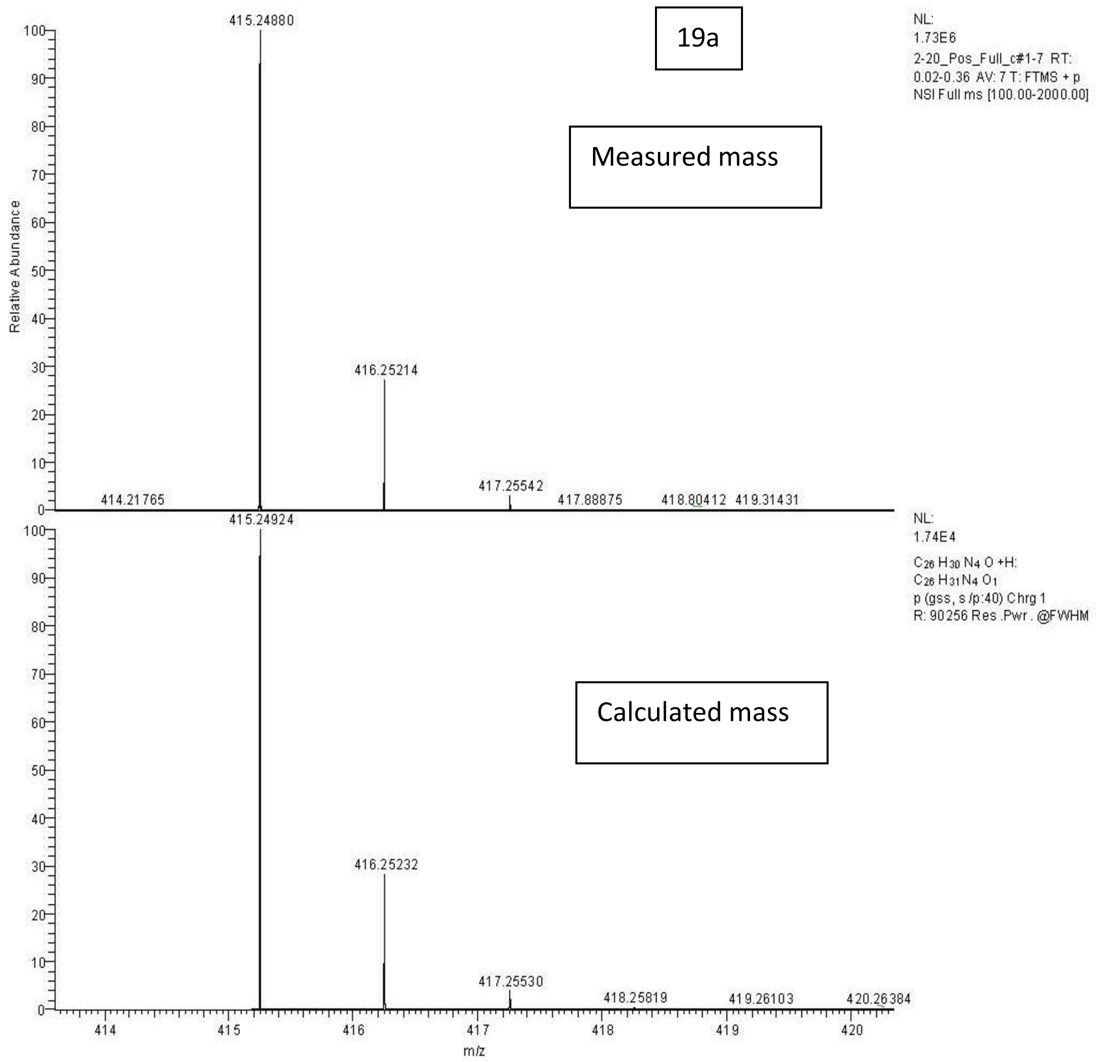

HRMS of (S)-N-(2-(1H-indol-3-yl) ethyl)-2,6-diamino-N-(naphthalen-2-ylmethyl) hexanamide (TFA salt) (19b)

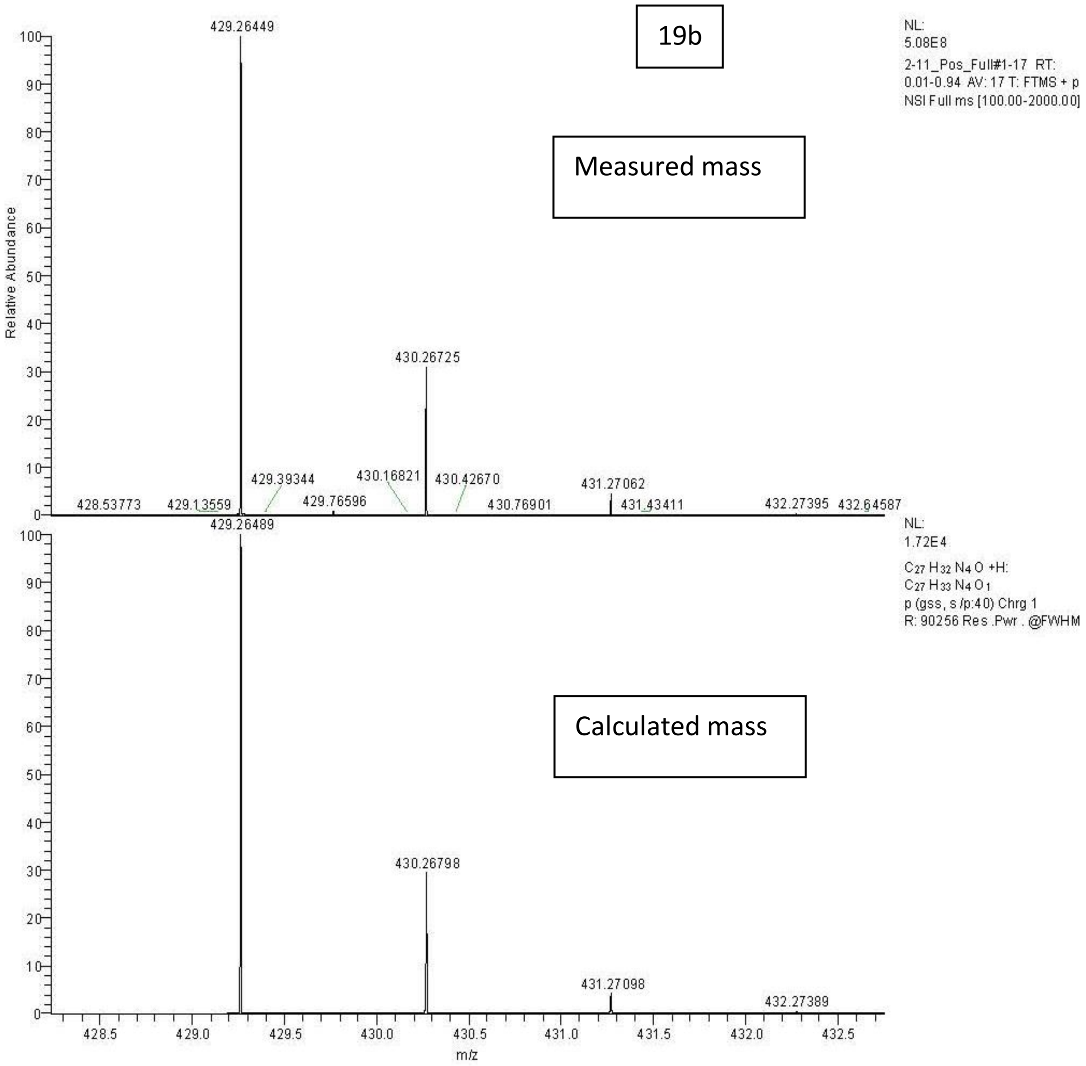

HRMS of N-(2-(1H-indol-3-yl) ethyl)-2,5-diguanidino-N-(naphthalen-2-ylmethyl) pentanamide (TFA salt) (20a)

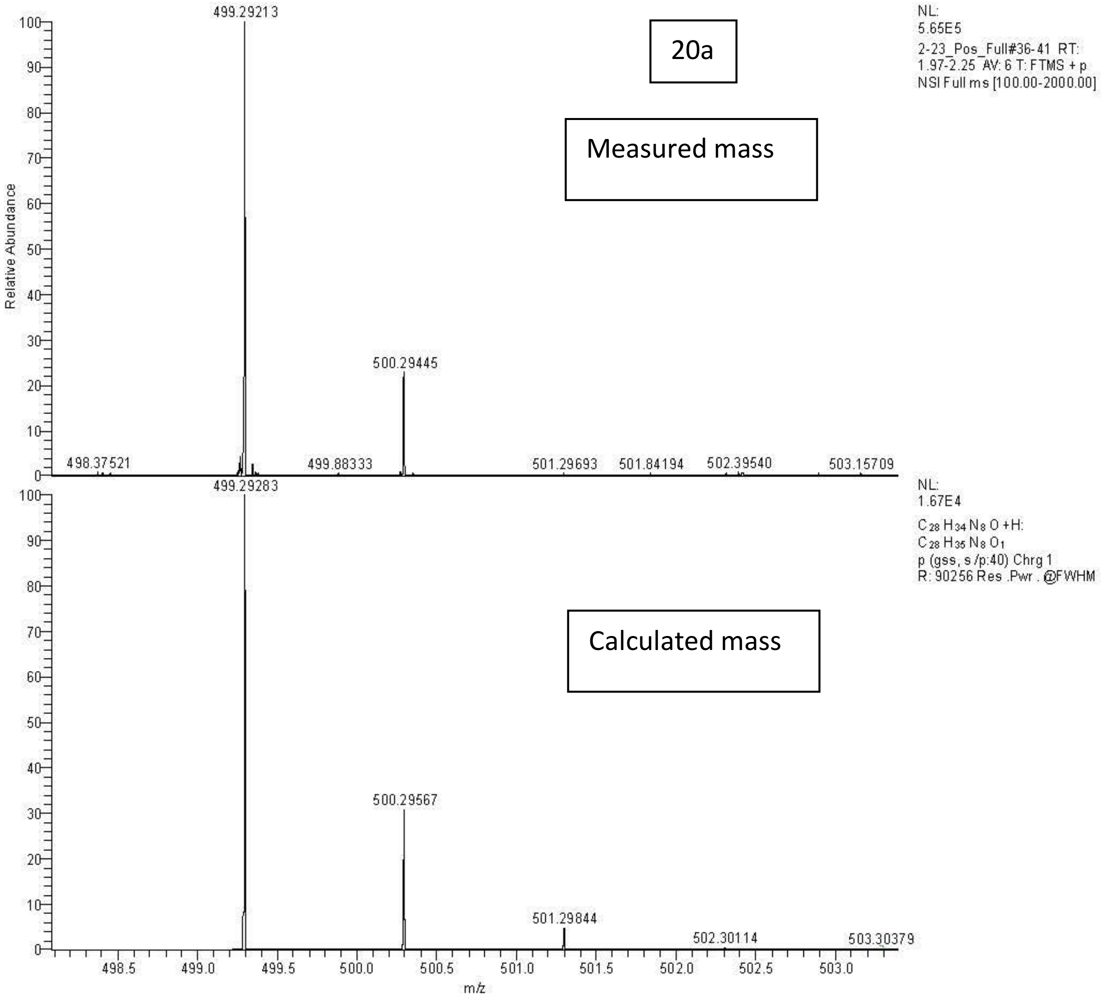

HRMS of (S)-N-(2-(1H-indol-3-yl) ethyl)-2,6-diguanidino-N-(naphthalen-2-ylmethyl) hexanamide (TFA salt) (20b)

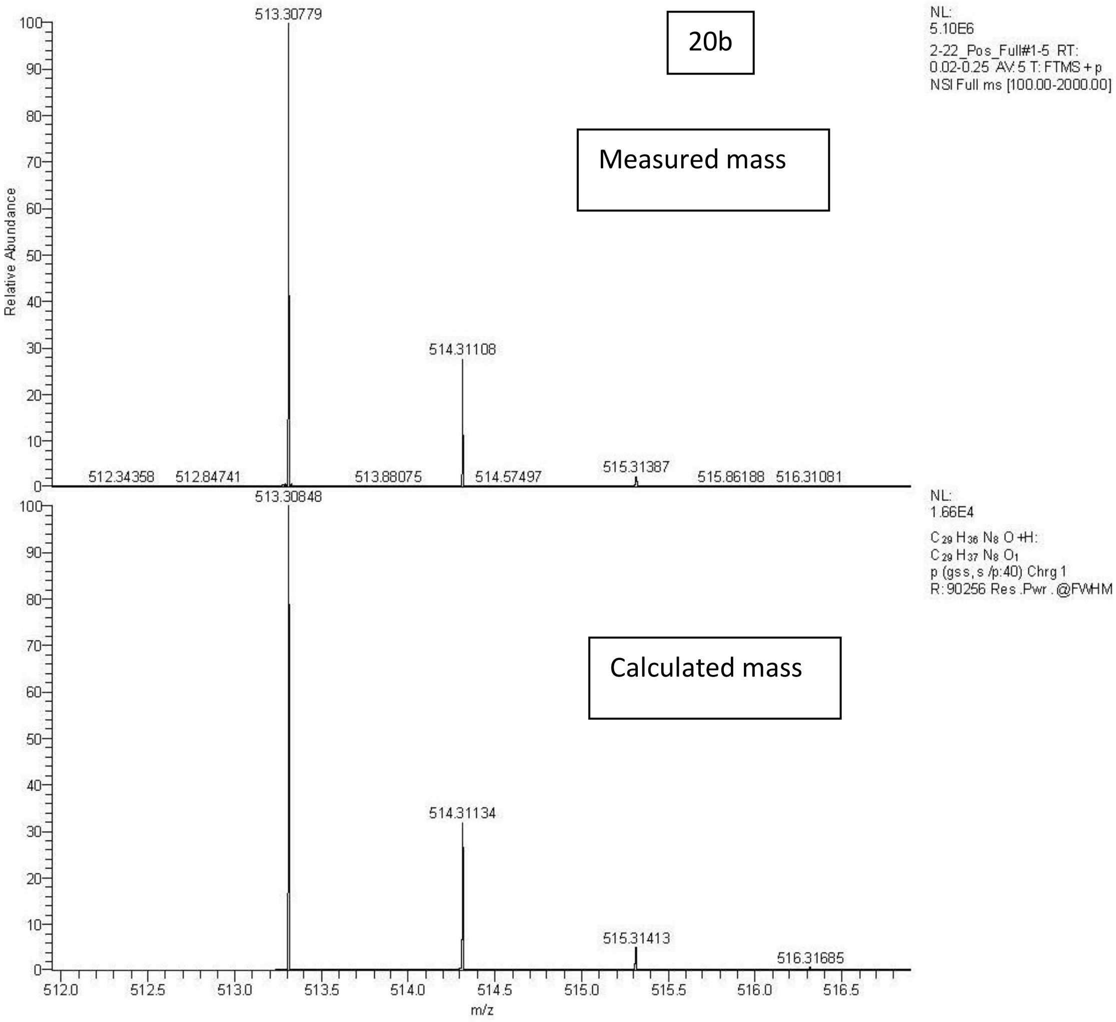

HRMS of Tert-butyl(S)-(1-((2-(1H-indol-3-yl) ethyl) (naphthalen-2-ylmethyl) amino)-1-oxo-5-(3-((2,2,4,6,7-pentamethyl-2,3-dihydrobenzofuran-5-yl) sulfonyl)guanidino) pentan-2-yl) carbamate (21)

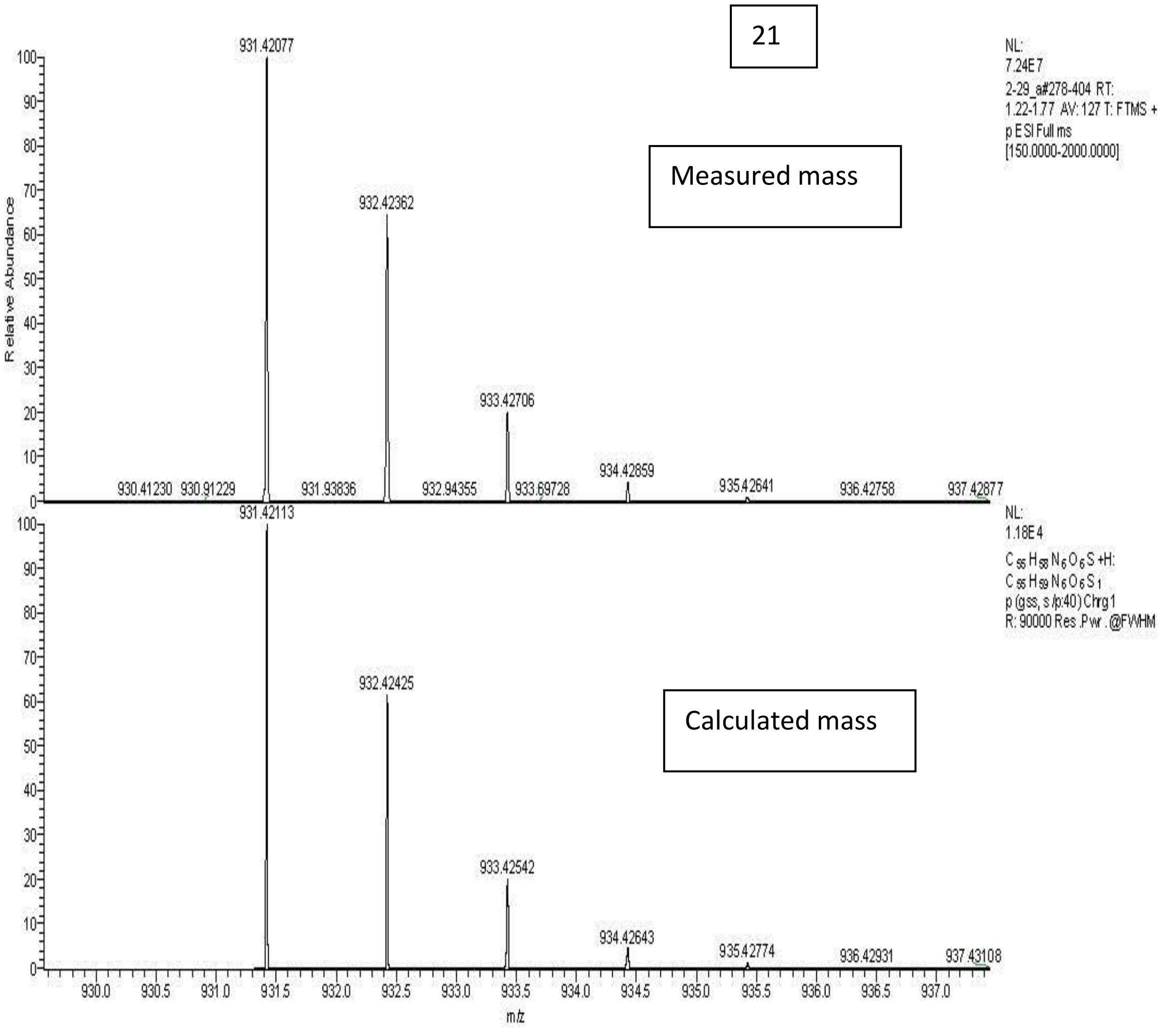

HRMS of (S)-N-(2-(1H-indol-3-yl) ethyl)-2-amino-N-(naphthalen-2-ylmethyl)-5-(3-((2,2,4,6,7-pentamethyl-2,3-dihydrobenzofuran-5-yl) sulfonyl) guanidino)pentanamide (22)

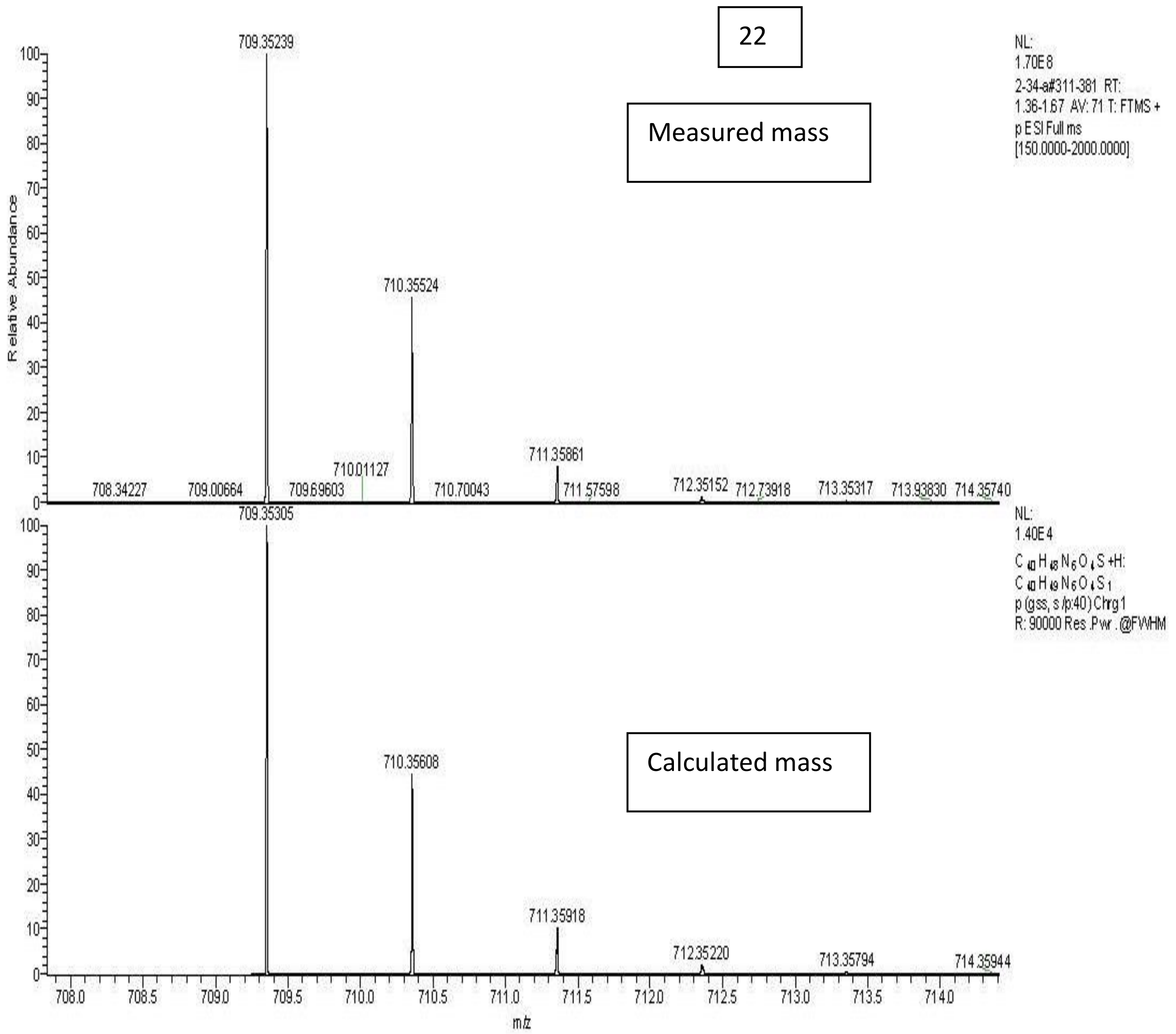

HRMS of (S)-N-(2-(1H-indol-3-yl) ethyl)-2-amino-5-guanidino-N-(naphthalen-2-ylmethyl) pentanamide (TFA salt) (23)

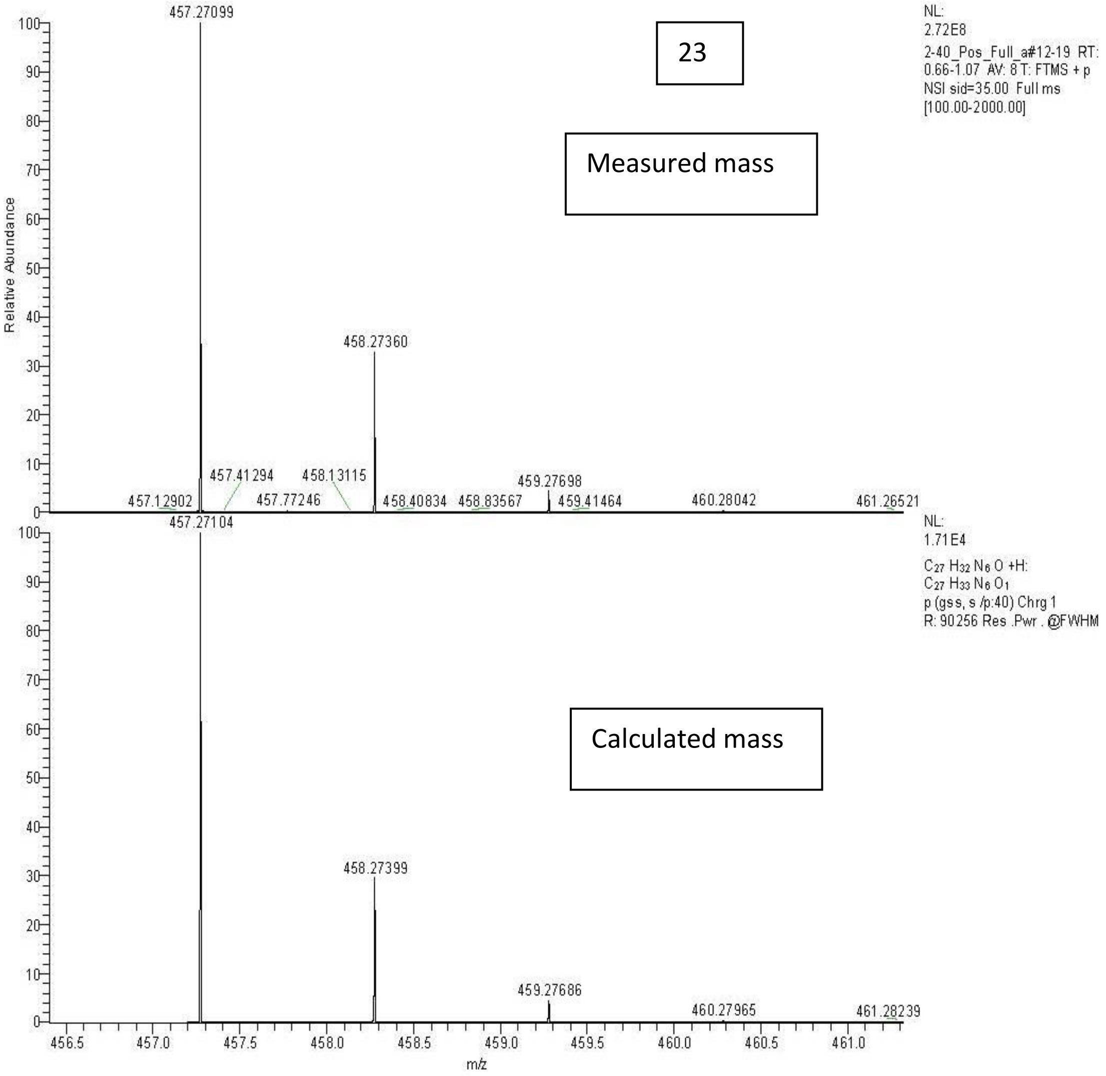

HRMS of N-benzyl-2-(1H-indol-3-yl) ethan-1-amine (24)

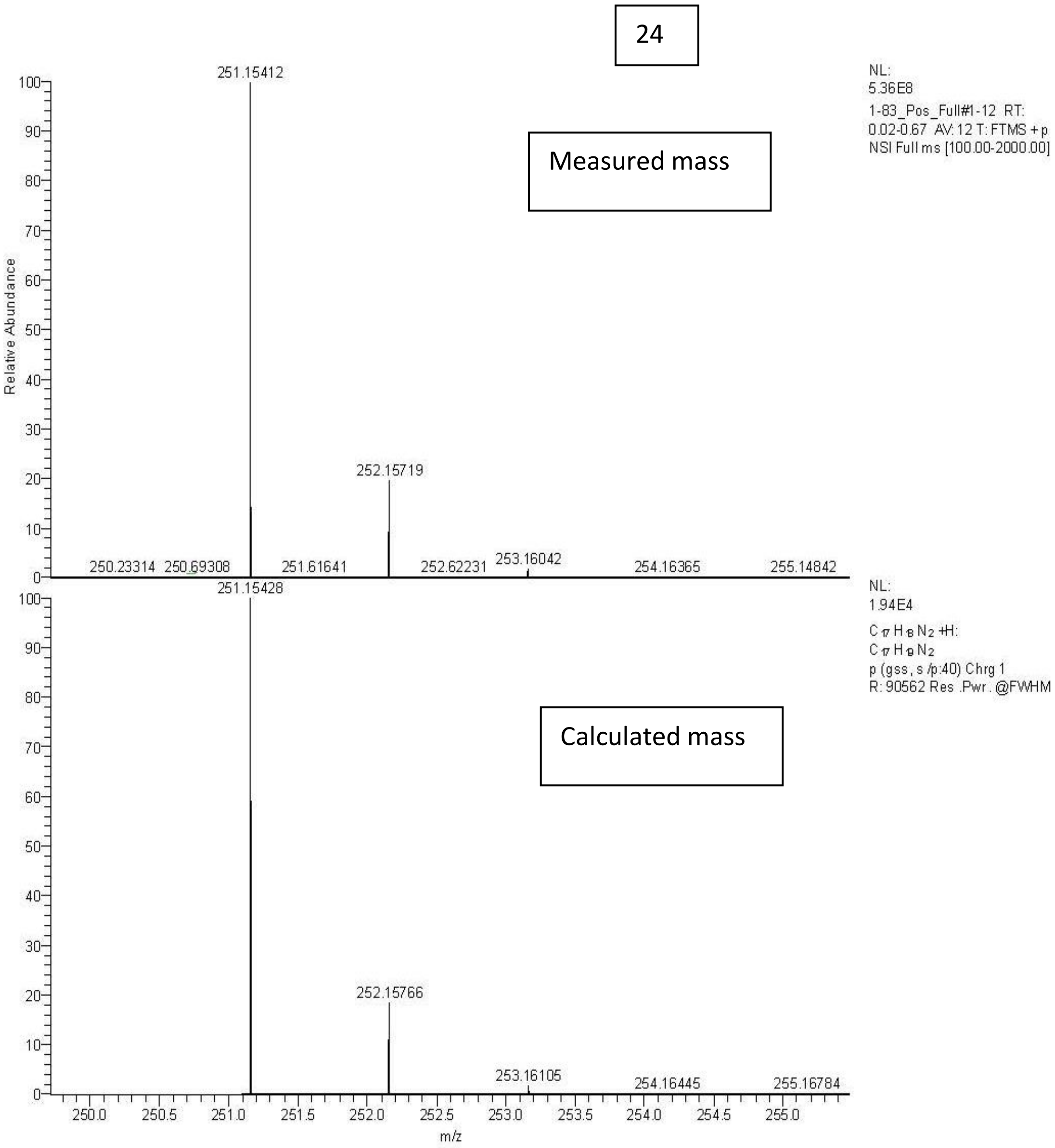

HRMS of Tert-butyl(2-((2-(1H-indol-3-yl)ethyl)(benzyl)amino)-2-oxoethyl)carbamate (25)

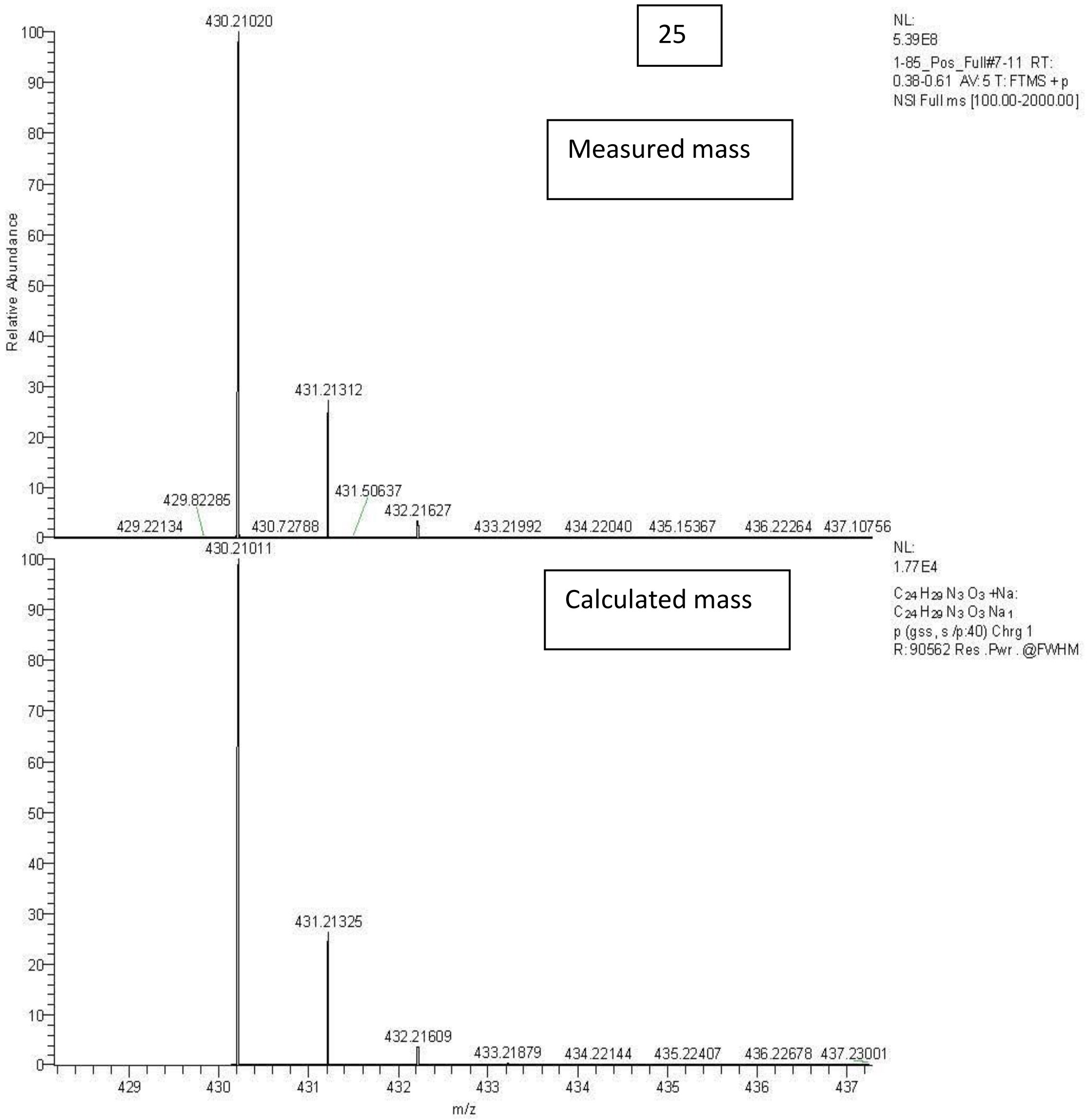

HRMS of N-(2-(1H-indol-3-yl) ethyl)-2-amino-N-benzylacetamide (26) (TFA salt)

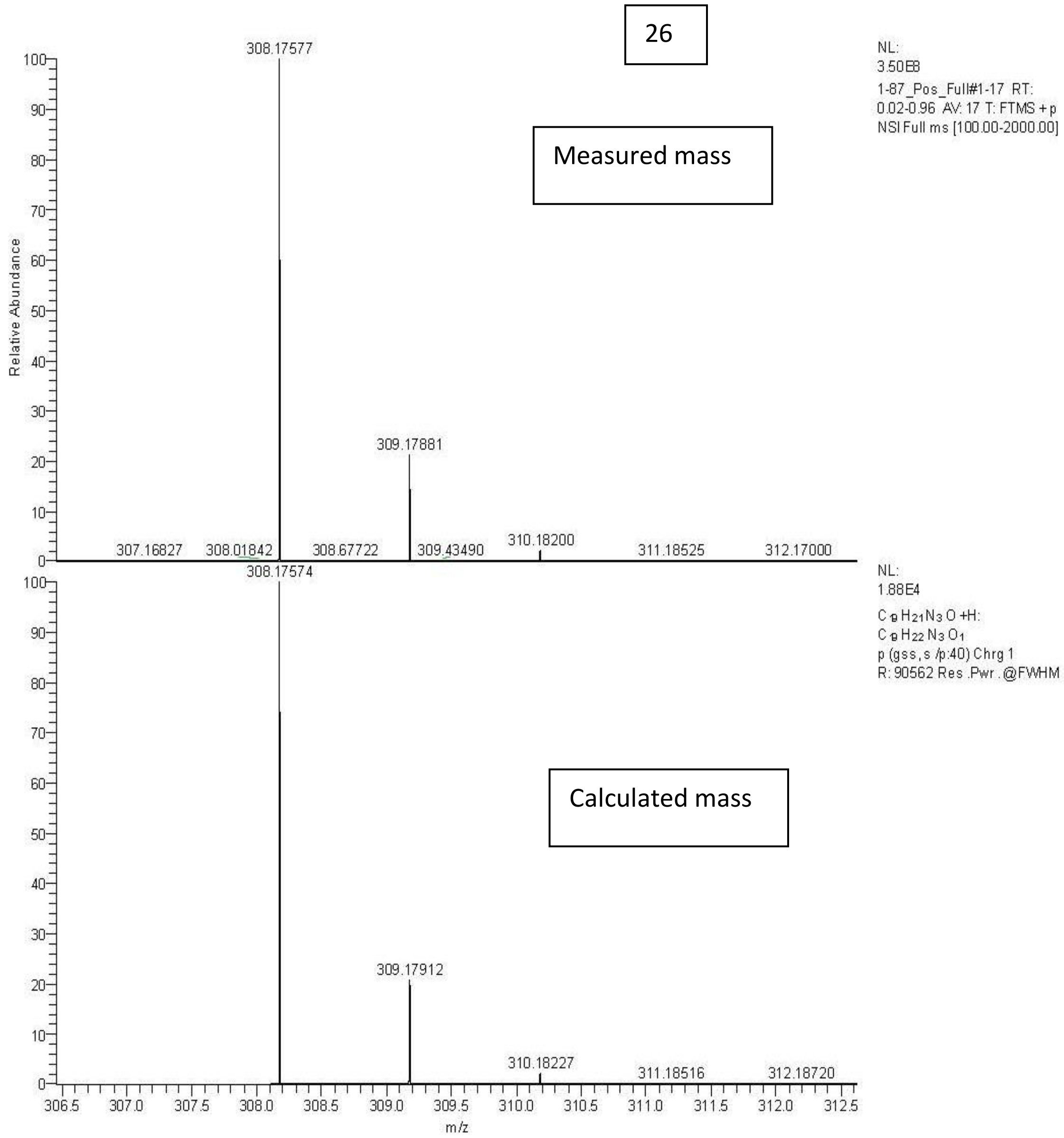

Supplement: Supplementary file 1 [file antibiotics-11-01074-s001.zip › antibiotics-1845495-supplementary.pdf]
